# Saudi Critical Care Society clinical practice guidelines on the prevention of venous thromboembolism in adults with trauma: reviewed for evidence-based integrity and endorsed by the Scandinavian Society of Anaesthesiology and Intensive Care Medicine

**DOI:** 10.1186/s13613-023-01135-8

**Published:** 2023-05-11

**Authors:** Marwa Amer, Mohammed S. Alshahrani, Yaseen M. Arabi, Ahmed Al-jedai, Hassan M. Alshaqaq, Abdulaziz Al-Sharydah, Faisal A. Al-Suwaidan, Hosam Aljehani, Thamer Nouh, Hassan Mashbari, Nehal Tarazan, Saad Alqahtani, Wail Tashkandi, Khalid Maghrabi, Muneerah Albugami, Samaher Hashim, Norah M. Alsubaie, Mohammad Alsenani, Haifa Algethamy, Thamir M. Alshammari, Ali Alaklabi, Nadia Ismail, Esraa S. Altawil, Alyaa Elhazmi, Ahmed Nahhas, Maha Aljuaid, Naif Alsadoon, Yasser Binbraik, Yuhong Yuan, Waleed Alhazzani

**Affiliations:** 1 Medical/Critical Pharmacy Division, King Faisal Specialist Hospital and Research Center, Al Mathar Ash Shamali, Riyadh, 11564 Saudi Arabia; 2 College of Medicine and Pharmacy, Alfaisal University, Riyadh, Saudi Arabia; 3 Department of Emergency and Critical Care, King Fahd Hospital of the University, Imam Abdulrahman Bin Faisal University, Dammam, Saudi Arabia; 4 Intensive Care Department, Ministry of National Guard Health Affairs, King Abdullah International Medical Research Center, King Saud Bin Abdulaziz University for Health Sciences, Riyadh, Saudi Arabia; 5 Therapeutic Affairs, Ministry of Health, Riyadh, Saudi Arabia; 6 Emergency Medicine Department, King Saud Medical City, Riyadh, Saudi Arabia; 7 Diagnostic and Interventional Radiology Department, King Fahd Hospital of the University, Imam Abdulrahman Bin Faisal University, Dammam, Saudi Arabia; 8 Clinical Excellence Administration and King Fahad Medical City, Second Health Cluster in Riyadh, Ministry of Health, Riyadh, Saudi Arabia; 9 Department of Interventional Neuroradiology, Neurosurgery, Neurocritical Care, King Fahd Hospital of the University, Imam Abdulrahman Bin Faisal University, Dammam, Saudi Arabia; 10 Trauma and Acute Care Surgery Unit, King Saud University, Riyadh, Saudi Arabia; 11 Department of Surgery, Jazan University, Jazan, Saudi Arabia; 12 Department of Medicine, McMaster University, Hamilton, Canada; 13 Department of Orthopedic Surgery, King Fahd Hospital of the University, Imam Abdulrahman Bin Faisal University, Dammam, Saudi Arabia; 14 Department of Surgery, King Abdulaziz University, Jeddah, Saudi Arabia; 15 Department of Critical Care, Fakeeh Care Group, Jeddah, Saudi Arabia; 16 Department of Critical Care Medicine, King Faisal Specialist Hospital and Research Center, Riyadh, Saudi Arabia; 17 Department of Internal Medicine, King Faisal Specialist Hospital and Research Center, Riyadh, Saudi Arabia; 18 Pulmonary and Critical Care Department, International Medical Center/First Clinic, Jeddah, Saudi Arabia; 19 Department of Surgery, King Saud University Medical City, Riyadh, Saudi Arabia; 20 Trauma Center, King Saud Medical City, Riyadh, Saudi Arabia; 21 Department of Anesthesia and Critical Care, King Abdulaziz University, Jeddah, Saudi Arabia; 22 College of Applied Medical Sciences, King Saud University, Riyadh, Saudi Arabia; 23 Department of Medicine, King Saud Bin Abdulaziz University for Health Sciences, King Abdullah International Medical Research Center, Riyadh, Saudi Arabia; 24 Department of Pharmacy, Imam Abdulrahman Bin Faisal University, Dammam, Saudi Arabia; 25 Pharmacy Department, Clinical Pharmacy Services, King Saud University Medical City, Riyadh, Saudi Arabia; 26 Dr Sulaiman Al-Habib Medical Group, Critical Care Department, Riyadh, Saudi Arabia; 27 Clinical Nursing Department, King Abdulaziz Medical City, Riyadh, Saudi Arabia; 28 Alshaya International Trading Company, Riyadh, Saudi Arabia; 29 Cardiac Sciences Department, King Saud University, Riyadh, Saudi Arabia; 30 Division of Gastroenterology, Department of Medicine, McMaster University, Hamilton, Canada; 31 Department of Health Research Methods, Evidence, and Impact, McMaster University, Hamilton, Canada; 32 Department of Critical Care, College of Medicine, King Saud University, Riyadh, Saudi Arabia; 33 Scientific Research Center, Directorate General of Armed Forces Medical Services, Riyadh, Saudi Arabia

**Keywords:** Venous thromboembolism, Pharmacologic VTE prophylaxis, Traumatic brain injury, Spinal cord injury, Non-operative solid organ injuries, Low molecular weight heparin, Unfractionated heparin, Adult trauma patient, Practice guidelines, GRADE

## Abstract

**Background:**

To develop evidence-based clinical practice guidelines on venous thromboembolism (VTE) prevention in adults with trauma in inpatient settings.

**Methods:**

The Saudi Critical Care Society (SCCS) sponsored guidelines development and included 22 multidisciplinary panel members who completed conflict-of-interest forms. The panel developed and answered structured guidelines questions. For each question, the literature was searched for relevant studies. To summarize treatment effects, meta-analyses were conducted or updated. Quality of evidence was assessed using the Grading Recommendations, Assessment, Development, and Evaluation (GRADE) approach, then the evidence-to-decision (EtD) framework was used to generate recommendations. Recommendations covered the following prioritized domains: timing of pharmacologic VTE prophylaxis initiation in non-operative blunt solid organ injuries; isolated blunt traumatic brain injury (TBI); isolated blunt spine trauma or fracture and/or spinal cord injury (SCI); type and dose of pharmacologic VTE prophylaxis; mechanical VTE prophylaxis; routine duplex ultrasonography (US) surveillance; and inferior vena cava filters (IVCFs).

**Results:**

The panel issued 12 clinical practice recommendations—one, a strong recommendation, 10 weak, and one with no recommendation due to insufficient evidence. The panel suggests starting early pharmacologic VTE prophylaxis for non-operative blunt solid organ injuries, isolated blunt TBIs, and SCIs. The panel suggests using low molecular weight heparin (LMWH) over unfractionated heparin (UFH) and suggests either intermediate–high dose LMWH or conventional dosing LMWH. For adults with trauma who are not pharmacologic candidates, the panel strongly recommends using mechanical VTE prophylaxis with intermittent pneumatic compression (IPC). The panel suggests using either combined VTE prophylaxis with mechanical and pharmacologic methods or pharmacologic VTE prophylaxis alone. Additionally, the panel suggests routine bilateral lower extremity US in adults with trauma with elevated risk of VTE who are ineligible for pharmacologic VTE prophylaxis and suggests against the routine placement of prophylactic IVCFs. Because of insufficient evidence, the panel did not issue any recommendation on the use of early pharmacologic VTE prophylaxis in adults with isolated blunt TBI requiring neurosurgical intervention.

**Conclusion:**

The SCCS guidelines for VTE prevention in adults with trauma were based on the best available evidence and identified areas for further research. The framework may facilitate adaptation of recommendations by national/international guideline policymakers.

**Supplementary Information:**

The online version contains supplementary material available at 10.1186/s13613-023-01135-8.

## Introduction

Traumatic injuries are a significant threat to public health and the fourth leading cause of mortality worldwide, accounting for 9% of deaths globally and 22.6% of years of potential life lost in Saudi Arabia [1]. Early preventable deaths after injury can be primarily attributed to uncontrolled hemorrhage and hypocoagulability which largely resolves within 24 h, after which hypercoagulability becomes prevalent. As such, pharmacologic VTE prophylaxis is an important preventive strategy after the initial resuscitation phase [2]. Deferring VTE prophylaxis during trauma-induced coagulopathy is associated with an increased VTE rate [2]. Therefore, it is desirable to initiate pharmacologic VTE prophylaxis once a hypocoagulable state is resolved and there are no signs of ongoing bleeding. The Eastern Association for the Surgery of Trauma in 2002 recognized the importance of initiating VTE prophylaxis, however, the ideal timing, agent, dose, and monitoring strategy were controversial [3]. Recently, the American Association for the Surgery of Trauma (AAST) Critical Care Committee and guidelines by Western Trauma Association (WTA) published updated consensus statements [4, 5]. However, current guidelines on this topic did not assess the quality of evidence and statements with limited consideration of other factors such as the balance of desirable and undesirable effects, patients’ values, resource considerations, feasibility, acceptability, and equity [6].

A survey of clinicians and surgeons who assess practice patterns of VTE prophylaxis use in TBI, SCI, and non-operative solid organ injuries in trauma centers across Saudi Arabia was recently published. The results showed variability in practice patterns regarding timing, type, and dosing of pharmacologic VTE prophylaxis, and other preventive strategies [7]. Therefore, the SCCS formulated a multidisciplinary panel of experts to develop trustworthy clinical practice guidelines on inpatient VTE prophylaxis in adults with trauma [8].

## Objectives

To provide evidence-based recommendations and identify knowledge gaps for future research priorities.

### Guidelines scope and target users

The guidelines provide recommendations to key stakeholders who provide care to adults hospitalized with major trauma in inpatient settings. The target users are clinicians (e.g., critical care physicians, surgeons, thrombosis experts, and interventional radiologists), allied health professionals (e.g., clinical pharmacists, nurses, nurse-practitioners, and physiotherapists), and policymakers.

## Methods

### Panel selection

The SCCS Guidelines Chapter selected expert panel members from different trauma-related disciplines. Panel members were selected to obtain a balance of expertise, gender, geographic location, and to address content needs. The panel included 22 panelists with different expertise in critical care, emergency medicine, general surgery, trauma surgery, neurosurgery, orthopedics, clinical pharmacy, nursing, interventional radiology, hematology and thrombosis, and research methodology. The Guidelines in Intensive Care Development and Evaluation (GUIDE) Group provided methodological support, including librarian and statistical support, throughout the guidelines’ development process. We followed best practices for guidelines development recommended by the Institute of Medicine and Guidelines International Network [8] and reported the guidelines following Appraisal of Guidelines for REsearch and Evaluation (AGREE) II reporting checklist [9] (Additional file 1: Appendix 1). Professional society with related interests and expertise was invited to participate as endorser. The guidelines are reviewed for evidence-based integrity and endorsed by the Scandinavian Society of Anaesthesiology and Intensive Care Medicine.

### Management of conflict of interests (COI)

All panel members completed a COI form prior to participation [10]. These included financial, intellectual, and personal COI.

The guidelines chairs reviewed all disclosures and adjudicated any potential conflicts prior to assigning panel members to different subgroups according to guidelines questions. Direct financial and industry-related COI were not permitted. We defined intellectual COI as leading clinical research that is directly relevant to a given recommendation/topic. Panel members with possible intellectual COI were not permitted to vote on corresponding recommendations. All reported/adjudicated COIs were secondary and were managed in accordance with the SCCS COI policy [11].

### Question development and outcome prioritization

The guidelines chairs developed the initial list of questions. Panel members were invited to provide feedback on the initial list and suggest additional questions, when applicable. We structured all actionable guidelines questions in the population, intervention, control, and outcome(s) (PICO) format. The guidelines Steering Committee incorporated the panel’s input and approved the final list of PICO questions (Additional file 2: Appendix 2). The guidelines questions covered the following eight domains: (1) timing of pharmacologic VTE prophylaxis in non-operative blunt solid organ injuries; (2) timing of pharmacologic VTE prophylaxis in isolated blunt TBI; (3) timing of pharmacologic VTE prophylaxis in isolated blunt spine trauma or fracture and/or SCI; (4) type of pharmacologic VTE prophylaxis; (5) dose of pharmacologic VTE prophylaxis; (6) mechanical VTE prophylaxis; (7) routine duplex US surveillance; and (8) prophylactic use of IVCFs.

We used the GRADE approach and prioritized outcomes according to the relative importance of each outcome to patients [12]. Critical outcomes were mortality, VTE, deep vein thrombosis (DVT), pulmonary embolism (PE), and adverse events (major bleeding, and need for surgical intervention).

### Patient engagement

A patient representative participated in dedicated teleconferences with the guidelines chairs. The patient representative provided perspectives on patients’ values and preferences, reviewed evidence summaries, and provided input on recommendations.

### Search strategy and study inclusion

A professional librarian drafted and performed an electronic literature search for each defined question or group of similar questions. The guidelines librarian, with input from the panel, identified pertinent search terms that included, at a minimum, trauma, VTE, DVT, PE combined with appropriate question-specific keywords (Additional file 2: Appendix 2). We restricted searches to capture only articles published in the English language from database inception up to October, 19, 2021. We searched three electronic bibliographic databases (MEDLINE, EMBASE, and Cochrane), and database of clinical trials (www.Clinicaltrials.gov) to identify ongoing or unpublished trials. For some questions, looking for systematic reviews (SRs) in the Epistemonikos database supplemented electronic searches. We relied on direct evidence whenever available for VTE prophylaxis in adults with trauma. Search results were imported into reference management software (EndNote version 20, EndNote, Philadelphia, PA), deduplicated, and imported into Covidence systematic review software (Veritas Health Innovation, Melbourne, Australia) to facilitate the SR process [13]. For each PICO question, two reviewers from the SR team screened the search results for relevant SRs, randomized controlled trials (RCTs), and observational studies (Additional file 2: Appendix 2, Table S1). Any citation identified by either reviewer as potentially relevant underwent full text review. Any disagreements about study inclusion were resolved by discussion with input from a non-conflicted panel member. Additionally, content experts reviewed the final list to identify any missed studies.

### Data abstraction and risk of bias assessment

When de novo or updated meta-analysis was required, the SR team abstracted relevant data from eligible studies using a standardized data abstraction form, and items relevant to risk of bias assessment. We conducted risk of bias assessments for each included study using the Cochrane Collaboration’s risk of bias tool for randomized trials or nonrandomized studies [14, 15].

### Analysis

For a given PICO question, we used meta-analytic techniques to generate pooled estimates across relevant studies, when applicable. All analyses were conducted using Review-Manager software version 5.3 (The Nordic Cochrane Centre, Copenhagen) [16]. In keeping with published guidance and due to methodological differences, we pooled RCTs and observational studies separately [17]. We used a random-effects model to pool weighted effect sizes across studies and used a fixed-effect model only when the number of studies was ≤ 3. Pooled estimates were reported as relative risks (RRs) or odds ratios (ORs) with 95% confidence intervals (CIs) for dichotomous outcomes; and mean differences with 95% CIs for continuous outcomes. We assessed heterogeneity using the Chi^2^ test (*P* < 0.05 indicating substantial heterogeneity) and the heterogeneity statistic *I*^2^ (> 50% indicating substantial heterogeneity), and by inspecting forest plots. For questions with insufficient quantitative data, we narratively summarized the evidence.

### Quality of evidence and grading of recommendations

The guidelines methodologists used the GRADE approach to assess the quality of evidence and summarize confidence in the estimate of the effect to support a recommendation. The quality of evidence was rated as high, moderate, low, or very low. We used Guideline Development Tool online software (Evidence Prime, Hamilton, ON) to generate evidence profiles (evidence summaries).

### Recommendation formulation and voting process

We used the EtD framework to formulate recommendations. Methodologist drafted the preliminary recommendations considering the balance of desirable and undesirable effects, quality of evidence, resource considerations and cost, equity, feasibility, and acceptability. Following the drafting of preliminary recommendations, we used guideline development tool Panel Voice (Evidence Prime, Hamilton, ON) to vote on the strength and direction of the recommendation after reviewing the components of the EtD framework. We assessed whether the desirable effects of an intervention would outweigh the undesirable effects, the strength of a recommendation reflects the panel’s degree of confidence in that balance assessment. Thus, a strong recommendation in favor of an intervention reflects the panel’s opinion that the desirable effects of adhering to a recommendation will clearly outweigh the undesirable effects. A weak recommendation in favor of an intervention indicates the judgment that the desirable effects will likely outweigh the undesirable effects. We used “we recommend” for strong recommendations and “we suggest” for weak recommendations. The implications of different recommendations to key stakeholders are presented in Additional file 2: Appendix 2, Table S2. Together, we generated best practice statements (BPSs) in compliance with the GRADE Working Group criteria [18].

Acceptance of a recommendation required at least 75% of the panel voting. Voters could provide feedback for consideration in revising statements that did not receive consensus in up to three rounds of voting. However, we achieved approval on all recommendations after a single round of voting.

## Results

**Fig. 1:**
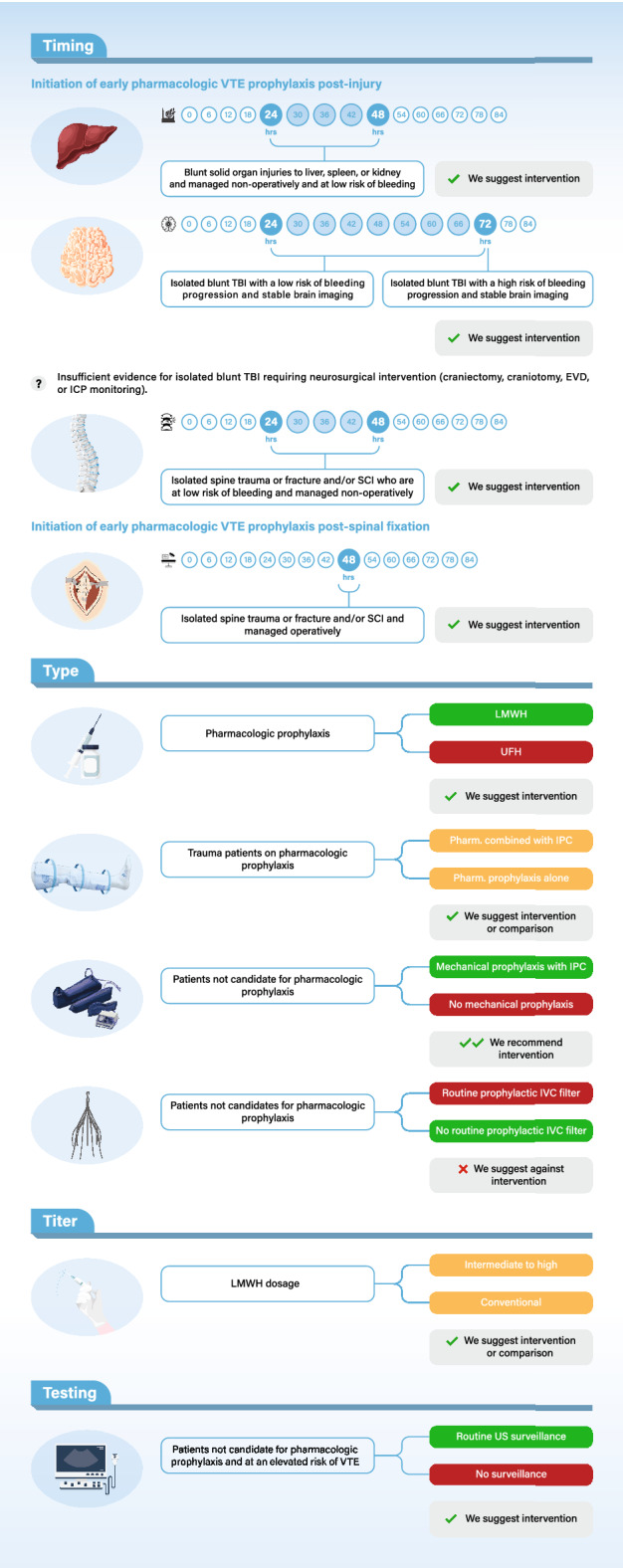
Summary of the recommendations (4Ts acronym: Timing, Type, Titer, Testing). EVD, external ventricular drain; ICP, intracranial pressure; IPC, intermittent pneumatic compression; IVC filter, inferior vena cava filter; LMWH, low molecular weight heparin; SCI, spinal cord injury; TBI, traumatic brain injury; UFH, unfractionated heparin; US, ultrasonography; VTE, venous thromboembolism. The shaded area in the timing reflects the acceptable time range for initiation of early pharmacologic VTE prophylaxis post-injury.

The panel issued 12 recommendations—one, a strong recommendation, 10 weak, and one with no recommendation due to insufficient evidence. Table 1 and Fig. 1 show a summary of the recommendations.

**Table 1:** Summary of recommendations

Recommendation	Strength and quality of evidence	Practical considerations	GRADE evidence profile and EtD framework
*Timing of Pharmacologic VTE prophylaxis in non-operative blunt solid organ injuries*			
Recommendation 1: In adults with blunt solid organ injuries to liver, spleen, or kidney who are managed non-operatively and are at low risk of bleeding, we *suggest* starting pharmacologic VTE prophylaxis early (i.e., within 24–48 h) over delayed initiation of pharmacologic VTE prophylaxis (> 48 h)^a^	Weak, very low	Clinicians should assess risk of bleeding. This recommendation is inapplicable to patients at high risk of major bleeding (e.g., high grade solid organ injuries and large hemoperitoneum) and those with hemodynamic instability	https://guidelines.gradepro.org/profile/FmzyU2rljqs
*Timing of pharmacologic VTE prophylaxis in TBI*			
Recommendation 2: In adults with isolated blunt TBI with a low risk of bleeding progression who had stable repeated brain imaging showing no bleeding progression and stable neurologic examination, we *suggest* early pharmacologic VTE prophylaxis (within 24–72 h post-injury) over delayed pharmacologic VTE prophylaxis (> 72 h)^b^	Weak, very low	This recommendation is inapplicable to patients with high risk of ICH spontaneous progression demonstrated at baseline or repeated brain imaging or patients with worsening of neurologic examination findings that necessitate upgrading care or emergent neurosurgical intervention	https://guidelines.gradepro.org/profile/aXj7XJvkfm8
Recommendation 3: In adults with isolated blunt TBI at a high risk of bleeding progression, we *suggest* starting early pharmacologic VTE prophylaxis 72 h post-injury with stable brain imaging that shows no bleeding progression and stable neurologic examination over delayed pharmacologic VTE prophylaxis (> 72 h). The decision is usually made in conjunction with multidisciplinary teams’ evaluation^b^	Weak, very low	Early pharmacologic VTE prophylaxis should be held until follow-up brain imaging (e.g., brain CT) demonstrates no bleeding progression. If progression is demonstrated, mechanical VTE prophylaxis (if no contradictions) should be continued and prophylactic IVCF and/or US screening to be considered This recommendation is inapplicable for patients with known coagulopathy (INR > 1.5, a partial thromboplastin time > 40 s, a platelet counts of < 100 × 10^9^/l)	https://guidelines.gradepro.org/profile/7y-WPSqYQvw
Statement 4: There is insufficient evidence to issue a recommendation on the use of early pharmacologic VTE prophylaxis in adults with isolated blunt TBI requiring neurosurgical intervention (including craniectomy, craniotomy, EVD, or ICP monitoring)	No recommendation	We agree that best practice includes withholding early pharmacologic VTE prophylaxis until follow-up brain imaging (e.g., brain CT) demonstrates no bleeding progression. If progression is demonstrated, we agree that best practice includes continuation of mechanical VTE prophylaxis (if no contradictions) and prophylactic IVCF and/or US screening to be considered (Best Practice Statement) We agree that best practice includes evaluation of timely initiation of pharmacologic VTE prophylaxis by multidisciplinary teams (trauma, neuro/neurosurgical, critical care, and clinical pharmacist) (Best Practice Statement)	https://guidelines.gradepro.org/profile/v7SWl8qQGJs Additional file 2: Appendix 2, Table S6
*Timing of pharmacologic VTE prophylaxis for spine trauma or fracture and/or SCI*			
Recommendation 5: In adults with isolated spine trauma or fracture and/or SCI who are at low risk of bleeding and are managed non-operatively, we *suggest* initiating pharmacologic VTE prophylaxis within 24–48 h post-injury over delayed pharmacologic VTE prophylaxis (> 48 h)^c^	Weak, very low	The presence of neurological deficit and presence/or expansion of intraspinal hematoma or epidural hematoma demonstrated on radiologic spine images (CT and/or MRI) should prompt discussion among multidisciplinary teams prior to initiating pharmacologic VTE prophylaxis Mechanical VTE prophylaxis (if no contradictions) should be initiated for all SCI patients. If initiation of pharmacologic VTE prophylaxis is anticipated to be delayed or interrupted, US screening and/or prophylactic IVCF may be considered	https://guidelines.gradepro.org/profile/HaApoQ153kU
Recommendation 6: In adults with isolated spine trauma or fracture and/or SCI and managed operatively, we *suggest* initiating early pharmacologic VTE prophylaxis within 48 h post-spinal fixation over delayed pharmacologic VTE prophylaxis (> 48 h)	Weak, very low	The presence of neurological deficit and presence/or expansion of intraspinal hematoma or epidural hematoma demonstrated on radiologic spine images (CT and/or MRI) should prompt discussion among multidisciplinary teams prior to initiating pharmacologic VTE prophylaxis Mechanical VTE prophylaxis (if no contradictions) should be initiated for all SCI patients. If initiation of pharmacologic VTE prophylaxis is anticipated to be delayed or interrupted, US screening and/or prophylactic IVCF may be considered	https://guidelines.gradepro.org/profile/XexGjIWaWJU
*Type of pharmacologic VTE prophylaxis*			
Recommendation 7: In adults with trauma who receive pharmacologic VTE prophylaxis, we *suggest* using LMWH (e.g., enoxaparin, dalteparin) over UFH	Weak, low	UFH is preferred in patients with end-stage renal disease and in those with low creatinine clearance (< 30 ml/min)	https://guidelines.gradepro.org/profile/xphSP0xqeg4
*Dose of pharmacologic VTE prophylaxis*			
Recommendation 8: In adults with trauma and low risk of bleeding who are prescribed LMWH (enoxaparin) for VTE prophylaxis, we *suggest* using either intermediate–high dose LMWH or conventional dosing LMWH	Weak, very low	Most common regimen used was enoxaparin 40 mg subcutaneous every 12 h This recommendation is inapplicable to those at a high risk for bleeding (patients older than 65 year, < 50 kg, have low creatinine clearance, and TBI or SCI patients who are high risk for bleeding)	https://guidelines.gradepro.org/profile/BWf_VYx4hqc
*Mechanical VTE prophylaxis*			
Recommendation 9: In adults with trauma who are not candidates for pharmacologic VTE prophylaxis, we *recommend* using mechanical VTE prophylaxis with IPC over no mechanical VTE prophylaxis when not contraindicated by lower extremity injury	Strong, very low		https://guidelines.gradepro.org/profile/8om8BWmPK-s
Recommendation 10: In adults with trauma taking pharmacologic VTE prophylaxis, we *suggest either* using adjunct mechanical VTE prophylaxis or pharmacologic VTE prophylaxis alone	Weak, very low		https://guidelines.gradepro.org/profile/ICFXyvu8Og=
*Routine duplex US surveillance*			
Recommendation 11: In adults with trauma who are at an elevated risk of VTE and are not candidates for pharmacologic VTE prophylaxis, we *suggest* routine bilateral lower extremity US to screen for asymptomatic DVT over no routine screening^d^	Weak, very low	This recommendation is inapplicable to trauma patients who are ambulating, those at low VTE risk, and patients with signs or symptoms of DVT in whom diagnostic imaging is indicated	https://guidelines.gradepro.org/profile/b1Vj16rY8u0
*Prophylactic IVCFs*			
Recommendation 12: In adults with trauma who are not candidates for pharmacologic VTE prophylaxis, we *suggest against* the routine placement of prophylactic IVCFs	Weak, very low	Clinicians may consider using temporary retrievable IVCF in patients who are expected to be off pharmacologic VTE prophylaxis for > 7 days (e.g., severely injured patients with an ongoing bleeding risk)	https://guidelines.gradepro.org/profile/BHbLqHtu0Gc

EVD, external ventricular drain; DVT, deep vein thrombosis; ICH, intracranial hemorrhage; ICP, intracranial pressure; IPC, intermittent pneumatic compression; IVCF, inferior vena cava filters; LMWH, low molecular weight heparin; SCI, spinal cord injury; TBI, traumatic brain injury; UFH, unfractionated heparin; US, Ultrasonography; VTE, venous thromboembolism

^a^Low risk of bleeding likely represent those with injury grade < 3 and hemodynamic stability ( please refer to Additional file 2: Appendix 2, Table S4 for more details)

^b^Low and high risk for spontaneous bleeding progression in TBI is defined as per Parkland protocol

^c^There is no standardized definition of a low risk of bleeding in spine trauma or fracture and/or SCI. Some studies defined this category as the absence of the following: paraspinal or epidural hematoma on advanced images, ICH progression (in patients with concurrent TBI), unstable clinically significant extracranial bleeding, neurological deficit, and medical contraindication to pharmacologic VTE prophylaxis

^d^There was no consensus on what factors define a high-risk trauma patient. Greenfield risk assessment profile (RAP score) has been suggested to identify trauma patients at high risk for VTE

### Timing of pharmacologic VTE prophylaxis in non-operative blunt solid organ injuries

Question: *In adults with blunt solid organ injuries to liver, spleen, or kidney managed non-operatively with low risk of bleeding, should we use early pharmacologic VTE prophylaxis (24–48 h) vs. delayed pharmacologic VTE prophylaxis (*> *48 h)?*

#### Recommendation 1

In adults with blunt solid organ injuries to liver, spleen, or kidney who are managed non-operatively and are at low risk of bleeding, we ***suggest*** starting pharmacologic VTE prophylaxis early (i.e., within 24–48 h) over delayed initiation of pharmacologic VTE prophylaxis (> 48 h) (weak recommendation, very low quality evidence).

#### Remark

Clinicians should assess risk of bleeding in all trauma patients who are considered for VTE prophylaxis. This recommendation is inapplicable to patients at elevated risk of major bleeding (e.g., high grade solid organ injuries and large hemoperitoneum) and those with hemodynamic instability.

#### Rationale

The decision to initiate early pharmacologic VTE prophylaxis in patients with blunt solid organ injuries requires weighing the risk of VTE against the risk of bleeding. Fortunately, most of these injuries are managed non-operatively or using minimally invasive techniques (e.g., angioembolization), especially in patients without hemodynamic compromise [19]. The definition of early versus late initiation of pharmacologic VTE prophylaxis is controversial. The WTA defined the timing of early prophylaxis as 12 to 24 h of admission, while the AAST used 48 h as the upper limit [4, 5]. A retrospective study measured thrombo-elastography in 304 patients with blunt solid organ injury and found that 13.8% of patients converted to a hypercoagulable state within 48 h [20].

We identified a meta-analysis of ten observational studies (*n* = 14,675) [21]. Five studies (*n* = 13,809) examined the association between timing of pharmacologic VTE prophylaxis and VTE outcome. The results showed a significant reduction in VTE with early, compared to late, pharmacologic VTE prophylaxis (OR 0.49, 95% CI 0.41–0.60; low quality). The largest study included 13,027 patients and found no clear effect on mortality in early pharmacologic VTE prophylaxis compared to late (RR 0.67, 95% CI 0.67–1.11; very low quality) [22]. Three studies (*n* = 13,261) reported on post-prophylaxis blood transfusion. The pooled estimate across studies was imprecise and failed to show a clear effect (OR 0.94, 95% CI 0.59–1.50; very low quality). Similarly, the effect on failure of non-operative management was unclear, yet the criteria of failure were not standardized among included studies (OR 1.10, 95% CI 0.92–1.30; very low quality). Notably, the majority of severe injuries (i.e., grade ≥ 3) were allocated to late pharmacologic VTE prophylaxis raising the concern of selection bias. We also assessed two recently published retrospective studies [23, 24]. The results were consistent with those of the meta-analysis, therefore, we decided not to update the meta-analysis (Additional file 2: Tables S13, S14).

Very low quality evidence showed that moderate benefit (reduction of VTE risk) of early pharmacologic VTE prophylaxis outweighed the possible harm (increased risk of bleeding or failure of non-operative management) in patients at low risk of bleeding and likely represent those with injury grade < 3 and hemodynamic stability (Additional file 2: Appendix 2, Table S4). We judged that early pharmacologic VTE prophylaxis is probably acceptable, feasible, cost-effective, and would have little to no impact on equity.

Given the above, we suggest starting early pharmacologic VTE prophylaxis within 24–48 h in patients with solid organ injuries who are managed non-operatively and are at low risk of bleeding.

### Timing of pharmacologic VTE prophylaxis in TBI

Question: *In adults with isolated blunt TBI who are at low risk of bleeding progression, should we recommend early pharmacologic VTE prophylaxis (within 24–72 h post-injury with stable brain imaging showing no bleeding progression) versus delayed pharmacologic VTE prophylaxis (*> *72 h)?*

#### Recommendation 2

In adults with isolated blunt TBI with a low risk of bleeding progression who had stable repeated brain imaging showing no bleeding progression and stable neurologic examination, we ***suggest*** early pharmacologic VTE prophylaxis (within 24–72 h post-injury) over delayed pharmacologic VTE prophylaxis (> 72 h) (weak recommendation, very low quality of evidence).

#### Remark

This recommendation is inapplicable to patients with elevated risk of intracranial hemorrhage (ICH) spontaneous progression demonstrated at baseline or repeated brain imaging (refer to PICO 3) or patients with worsening of neurologic examination findings that necessitate upgrading care or emergent neurosurgical intervention (refer to PICO 4).

#### Rationale

Parkland protocol defined low risk for spontaneous progression of traumatic ICH as those with subdural or epidural hemorrhage < 8 mm, brain contusion ≤ 2 cm, < 8 mm intraparenchymal hemorrhage, localized subarachnoid hemorrhage, and no more than a single parenchymal contusion per lobe [25, 26]. We used these variables to define low-risk population and our search identified one RCT (*n* = 62) and nine observational studies (*n* = 2012) [27–36].

The pooled estimates from five observational studies (*n* = 1361) showed no difference between early and late pharmacologic VTE prophylaxis in mortality (RR 0.86; 95% CI 0.50–1.46; very low quality) [30–34]. These results were limited by serious indirectness because of the inclusion of polytrauma patients (not isolated blunt TBI) and imprecision. The pooled estimates from five observational studies (*n* = 1172) demonstrated that early pharmacologic VTE prophylaxis was associated with lower risk of DVT (RR 0.55; 95% CI 0.33–0.93; very low quality) [29–32, 35]. Additionally, the pooled estimates from five observational studies (*n* = 1172) demonstrated a possible reduction in PE in patients receiving early pharmacologic VTE prophylaxis, however, the 95% CI was imprecise (RR 0.83; 95% CI 0.31–2.20; very low quality) [29–32, 35]. These results were also limited by serious risk of bias and serious indirectness because of the inclusion of polytrauma patients, mixed TBI (blunt and penetrating). Pooled estimates of eight observational studies (*n* = 1919) reveled no significant reduction in VTE associated with early pharmacologic VTE prophylaxis (RR 1.08; 95% CI 0.64–1.81) [28–34, 36]. The pilot RCT (DEEP I pilot RCT; *n* = 62) suggests that early pharmacologic VTE prophylaxis reduced the risk of VTE and DVT by 2.6%. Evidence was rated down for imprecision due to the low event rate, target sample size not met, and wide 95% CI leading to very serious imprecision (RR 0.28, 95% CI 0.01–6.53, very low quality) [27].

Concerns about hemorrhagic complications have been the main reason to delay pharmacologic VTE prophylaxis. However, even in the absence of anticoagulants, the baseline rate of radiographic progression of traumatic ICH ranges between 3 and 19%, indicating that a substantial percentage of TBI progressions are likely related to the natural evolution of the injury rather than a consequence of pharmacologic prophylaxis [37–40].

Meta-analysis of eight observational studies (*n* = 2383) showed no association between early pharmacologic VTE prophylaxis and the risk of ICH progression (RR 0.84; 95% CI 0.58–1.21; very low quality) [28–35]. Similarly, pooled estimates of five observational studies (*n* = 1361) demonstrated no association of early initiation of pharmacologic VTE prophylaxis and the rate of acute neurosurgical intervention required for ICH progression (RR 0.92; 95% CI 0.55–1.53; very low quality) [29–33]. Likewise, DEEP I pilot RCT demonstrated nonsignificant radiographic ICH progression with early initiation of pharmacologic VTE prophylaxis (5.9% compared to 3.6% in placebo) [27]. Nevertheless, none of these progressions were clinically significant (i.e., no worsening of neurological status nor acute neurosurgical intervention were required). Of note, two studies reported an incidence of extracranial hemorrhagic complications (e.g., hematuria) which was deemed insignificant neither statistically nor clinically [30, 31].

Therefore, the desirable consequences of early pharmacologic VTE prophylaxis outweigh the small undesirable effects. The intervention was judged to probably be cost-effective and feasible to implement. It likely has no impact on health equity, and most likely is acceptable to key stakeholders.

Considering the very low quality of evidence, we issued a weak recommendation suggesting early pharmacologic VTE prophylaxis (within 24–72 h post-injury with stable repeated brain imaging showing no bleeding progression and stable neurologic examination) over delayed pharmacologic VTE prophylaxis (> 72 h). Our recommendation is similar to those of the Neurocritical Care Society (NCS), AAST, and WTA’s guidelines [4, 5, 41]. Yet, the Brain Trauma Foundation guidelines did not indicate sufficient evidence to support the timing of pharmacologic VTE prophylaxis in TBI patients [42]. Thus far, there is a high demand for high-quality RCTs. We suggest utilizing a standard protocol in future research to enable objective, and consistent assessment of the TBI radiographic findings.

Question: *In adults with isolated blunt TBI who are at high risk for bleeding progression, should we recommend early pharmacologic VTE prophylaxis (within 72 h post-injury with stable brain imaging that showed no bleeding progression prior to pharmacologic VTE prophylaxis commencement) versus delayed pharmacologic VTE prophylaxis (*> *72 h)?*

#### Recommendation 3

In adults with isolated blunt TBI at an elevated risk of bleeding progression, we ***suggest*** starting early pharmacologic VTE prophylaxis 72 h post-injury with stable brain imaging that shows no bleeding progression and stable neurologic examination over delayed pharmacologic VTE prophylaxis (> 72 h). The decision is usually made in conjunction with multidisciplinary teams’ evaluation (trauma, neuro/neurosurgical, critical care, and clinical pharmacist) (weak recommendation, very low quality of evidence).

#### Remarks

- Early pharmacologic VTE prophylaxis should be held until follow-up brain imaging (e.g., brain CT) demonstrates no progression. If progression is demonstrated, mechanical VTE prophylaxis (if no contradictions) should be continued and prophylactic IVCF and/or US screening to be considered.
- This recommendation is inapplicable for patients with known coagulopathy (INR > 1.5, a partial thromboplastin time > 40 s, a platelet counts of < 100 × 10^9^/l).

#### Rationale

The Parkland protocol defined high risk for spontaneous progression of traumatic ICH as those with subdural or epidural hemorrhage ≥ 8 mm, contusion or intraventricular hemorrhage > 2 cm, and > 1 contusion per brain lobe [25, 26]. Furthermore, the original Parkland protocol considered patients who required emergent neurosurgical interventions at high risk for bleeding progression. However, current literature lacks consistent criteria to classify the risk of hemorrhagic progression. Therefore, we used these variables to identify relevant studies for this question. We identified 12 relevant observational studies (*n* = 4393) that fit this question [28, 31, 33–36, 43–48]. There were no relevant RCTs on this topic. Meta-analysis of six observational studies demonstrated that early pharmacologic VTE prophylaxis was associated with lower risk of DVT (*n* = 3010; RR, 0.57; 95% CI 0.42–0.78; very low quality) and the risk of PE (*n* = 3010; RR, 0.54; 95% CI 0.30–0.98; very low quality) compared to delayed one [31, 35, 43–46]. However, the results of DVT and PE were limited by indirectness attributable to the inclusion of polytrauma patients in two studies. In addition, estimates of the effect on PE were serious for imprecision due to the low number of events. Despite the reduction in DVT and PE risk, there was no associated mortality benefit with early pharmacologic VTE prophylaxis (RR, 1.09; 95% CI 0.87–1.37; very low quality) [31, 33, 34, 43–46]. Estimates of eight observational studies (*n* = 1393) showed no significant association between early pharmacologic VTE prophylaxis and increased risk of ICH progression (RR, 0.89; 95% CI 0.58–1.37; very low quality) [28, 31, 33–35, 44, 45, 47]. Furthermore, none of the included studies reported clinically significant extracranial bleeding. Moreover, pooled estimates across five studies (*n* = 3146) showed no difference among early and late pharmacologic VTE prophylaxis in the rate of acute neurosurgical interventions required for hemorrhage progression (RR, 1.19; 95% CI 0.69–2.07; very low quality) [31, 33, 43, 45, 46]. We acknowledged the publication of one recent study after completing our meta-analysis [49]. The results were assessed and deemed consistent with the recommendation, and we did not update the meta-analysis (Additional file 2: Tables S13, S14).

In light of the very low quality in the evidence, we issued a weak recommendation suggesting early pharmacologic VTE prophylaxis (72 h post-injury) over delayed pharmacologic VTE prophylaxis (> 72 h). Moreover, ICH stability was considered a prerequisite for starting early pharmacologic VTE prophylaxis, consistent with the WTA guidelines [5]. Although other guidelines have not distinguished TBI patients with low versus high risk for bleeding, the NCS issued a weak recommendation toward earlier pharmacologic VTE prophylaxis in TBI patients (24–48 h of hospital presentation), irrespective of the risk of bleeding [41]. Similarly, the AAST supported initiating prophylaxis within 24–72 h following admission with a prerequisite of stable ICH [4]. Moreover, the AAST panel suggested that the timing of prophylaxis initiation should be individualized based on TBI severity, which is consistent with our advocacy for multidisciplinary team evaluation. On the other hand, the Brain Trauma Foundation guidelines concluded that the evidence is insufficient to make recommendations about the timing of pharmacologic VTE prophylaxis [42]. To date, the efficacy and safety of early VTE prophylaxis among TBI patients with an elevated risk of bleeding are uncertain. The lack of RCTs combined with several flaws in the observational studies challenges the quality of evidence; thus, this question was considered a research priority and needed high-quality RCTs with adequate power. We suggest using standard criteria, like the Parkland protocol, to consistently assess the TBI radiographic findings and associated risk of bleeding. In addition, the definitions of early pharmacologic VTE prophylaxis are inconsistent among studies, ranging from 24–72 h post-injury. Therefore, future studies need to consider following a unified timeframe to decrease clinical practice and research variability.

Question: *In adults with TBI requiring intracranial pressure (ICP) monitoring or external ventricular drain (EVD) or craniotomy or craniectomy, should we recommend early pharmacologic VTE prophylaxis (24 h from the procedure and follow-up stable brain imaging) versus delayed pharmacologic VTE prophylaxis (*> *24 h)?*

#### Statement 4

There is insufficient evidence to issue a recommendation on the use of early pharmacologic VTE prophylaxis in adults with isolated blunt TBI requiring neurosurgical intervention (including craniectomy, craniotomy, EVD, or ICP monitoring).

We agree that best practice includes withholding early pharmacologic VTE prophylaxis until follow-up brain imaging (e.g., brain CT) demonstrates no progression. If progression is demonstrated, we agree that best practice includes continuation of mechanical VTE prophylaxis (if no contradictions) and prophylactic IVCF and/or US screening to be considered (BPS).

We agree that best practice includes evaluation of timely initiation of pharmacologic VTE prophylaxis by multidisciplinary teams (trauma, neuro/neurosurgical, critical care, and clinical pharmacist) (BPS).

#### Rationale

Patients with TBI who undergo acute neurosurgical interventions are at risk of ICH progression [50]. A recent observational study showed that early pharmacologic VTE prophylaxis reduces the risk of VTE; but at the expense of an increased risk of repeated neurosurgical intervention [51]. Therefore, the optimal timing of initiating pharmacologic VTE prophylaxis in this population is unclear. We identified 11 relevant observational studies [29–31, 43–48, 51, 52]. Pooled estimates from eight studies (*n* = 3779) showed a 3.4% DVT risk reduction associated with early pharmacologic VTE prophylaxis (RR, 0.58; 95% CI 0.44–0.76; very low quality) [29–31, 43–46, 52]. Similarly, it demonstrated a 0.9% reduction in PE (RR, 0.58; 95% CI 0.35–0.97; very low quality) [29–31, 43–46, 52]. Nevertheless, the quality of both outcomes was downgraded due to serious indirectness because of the inclusion of polytrauma patients in four studies and imprecision. Pooled estimates across five studies (*n* = 5202) demonstrated a possible reduction in VTE in patients receiving early pharmacologic VTE prophylaxis, however, the 95% CI was imprecise (RR, 0.83; 95% CI 0.69–1; very low quality) [29–31, 44, 51].

On contrary, pooled estimates from seven studies (*n* = 2135) showed no difference between early and late pharmacologic VTE prophylaxis in ICH progression (RR, 1.06; 95% CI 0.75–1.51; very low quality) [29–31, 44, 45, 47, 52]. Additionally, the use of early pharmacologic VTE prophylaxis was probably associated with an increased risk of acute neurosurgical intervention; however, the 95% CI could not exclude any difference (*n* = 7949; RR, 1.57; 95% CI 0.90–2.73; very low quality) [29–31, 43, 45, 46, 51]. The risk of repeated neurosurgical interventions with early pharmacologic VTE prophylaxis appears to be the highest particularly during the first 3 days after the index procedure. Ultimately, early pharmacologic VTE prophylaxis was associated with significantly higher mortality (*n* = 7023; RR, 1.23; 95% CI 1.06–1.42; very low quality) [30, 31, 43–46, 51]. The subgroup analysis by Byrne et al. demonstrated that TBI patients who underwent ICP monitoring or drain insertion and received early pharmacologic VTE prophylaxis were associated with higher mortality [51]. Nonetheless, this association was not observed in those who underwent craniotomy or craniectomy. It should be noted that none of the included studies examined the clinical neurological deterioration after the commencement of pharmacologic VTE prophylaxis, and none reported clinically significant extracranial hemorrhage.

Accordingly, we judged the desirable and undesirable effects as moderate. In addition, we could not determine the direction of the balance of effects. The overall quality of the evidence of effects is very low. Data on required resources were not available. Furthermore, there is likely an important variability in patients’ values. Due to the very low quality in the evidence and lack of clarity about the risk to benefit ratio, we judged the current body of evidence to be insufficient to support a recommendation for or against early VTE prophylaxis in this population. Although we did not make a recommendation on optimal timing of VTE prophylaxis, we encourage clinicians to assess ICH stability using CT imaging prior to commencing pharmacologic VTE prophylaxis and to subsequently monitor patient closely for signs of bleeding. The decision should be individualized, weighing the benefits and risks with input from relevant healthcare disciplines.

The AAST did not make a distinct recommendation for TBI patients requiring neurosurgical intervention [4]. However, the NCS issued a weak recommendation for early pharmacologic VTE prophylaxis in TBI patients (24 h after craniotomy) [41]. We identified this area as a research gap that requires further study.

### Timing of pharmacologic VTE prophylaxis for spine trauma or fracture and/or SCI

Question: *In adults with isolated spine trauma or fracture and/or SCI with low risk of bleeding and who are managed non-operatively, should we rec*ommend *early pharmacologic VTE prophylaxis (within 24–48 h post-injury) versus delayed pharmacologic VTE prophylaxis (*> *48 h)?*

#### Recommendation 5

In adults with isolated spine trauma or fracture and/or SCI who are at low risk of bleeding and are managed non-operatively, we ***suggest*** initiating pharmacologic VTE prophylaxis within 24–48 h post-injury over delayed pharmacologic VTE prophylaxis (> 48 h) (weak recommendation, very low quality of evidence).

#### Remarks

- The presence of neurological deficit and presence/or expansion of intraspinal hematoma or epidural hematoma demonstrated on radiologic spine images (CT and/or MRI) should prompt discussion among multidisciplinary teams (trauma, neuro/neurosurgical, orthopedic trauma, critical care, and clinical pharmacist) in collaboration with the spine surgery team prior to initiating pharmacologic VTE prophylaxis.
- Mechanical VTE prophylaxis (if no contradictions) should be initiated for all SCI patients (refer to PICO 9, 10). If initiation of pharmacologic VTE prophylaxis is anticipated to be delayed or interrupted, US screening and/or prophylactic IVCF may be considered.

#### Rationale

Patients with a spine injury are at substantial risk of developing VTE complications due to either immobilization or trauma [53]. Several studies have found a greater incidence of VTE in paraplegia than in tetraplegia (16.7% versus 3.3%) [54]. The highest incidence of VTE occurs among patients with thoracic segment SCI [55]. Traumatic intraspinal hematoma is poorly described in the literature with reported incidence of 0.5% to 7.5% [53]. We found two observational studies that addressed this question [56, 57]. The first study (*n* = 275) showed an association between early pharmacologic VTE prophylaxis and lower VTE risk (RR 0.08, 95% CI 0.02–0.31; very low quality) [56]. Similarly, these two studies (*n* = 8827) showed that early pharmacologic VTE prophylaxis was associated with reduced DVT (RR 0.16, 95% CI 0.07–0.41; moderate quality) [56, 57]. The pooled estimates from these two studies (*n* = 8827) showed a reduction in PE risk with early pharmacologic VTE prophylaxis (RR 0.39, 95% CI 0.27–0.57; moderate quality) [56, 57]. One study assessed adverse effects (*n* = 8552) [57]. The need of post-VTE prophylaxis decompressive laminectomy was not different among those receiving early pharmacologic VTE prophylaxis (RR 0.66; 95% CI 0.40–1.09; very low quality). Likewise, mortality and post-prophylaxis blood transfusion were not significantly different among those receiving early pharmacologic VTE prophylaxis (RR 1.24, 95% CI 0.81–1.89; very low quality), (RR 1.09, 95% CI 0.72–1.65; very low quality), respectively [57]. The results were limited by serious indirectness of outcome (need of post-VTE prophylaxis decompressive laminectomy was used as surrogate marker for intraspinal hematoma), and very serious imprecision. No studies reported the risk of intraspinal hematoma, epidural hematoma, worsening of neuro or motor examination, and clinically significant extracranial bleeding.

The period of acute hospitalization post-SCI, particularly during the first 2–3 weeks, is associated with the highest risk of VTE. Based on the available evidence, it is likely that the benefits of early pharmacologic VTE prophylaxis within 24–48 h post-injury outweigh the minimal risks. This is because the risk of VTE is greater in the acute-care phase of SCI than in the chronic phase. It would probably have no impact on equity, possibly cost-effective, and likely acceptable and feasible. Moreover, baseline risk of VTE in SCI is perceived to be higher than hemorrhagic risk. Uncertainty remains due to incomplete reporting of other important outcomes related to adverse effects. The overall quality of evidence was very low. Therefore, we suggest starting early pharmacologic VTE prophylaxis within 24–48 h post-injury. Our recommendation is consistent with recommendations by other professional societies [5, 58]. We also highlighted that this area is understudied, and high-quality studies are needed.

Question: *In adults with spine trauma or fracture and/or SCI managed operatively, should we recommend early pharmacologic VTE prophylaxis (within 48 h post-spinal fixation) versus delayed pharmacologic VTE prophylaxis (*> *48 h)?*

#### Recommendation 6

In adults with isolated spine trauma or fracture and/or SCI and managed operatively, we ***suggest*** initiating early pharmacologic VTE prophylaxis within 48 h post-spinal fixation over delayed pharmacologic VTE prophylaxis (> 48 h) (weak recommendation, very low quality of evidence).

#### Remarks:

- The presence of neurological deficit and presence/or expansion of intraspinal hematoma or epidural hematoma demonstrated on radiologic spine images (CT and/or MRI) should prompt discussion among multidisciplinary teams (trauma, neuro/neurosurgical, orthopedic trauma, critical care, and clinical pharmacist) in collaboration with the spine surgery team prior to initiating pharmacologic VTE prophylaxis.
- Mechanical VTE prophylaxis (if no contradictions) should be initiated for all SCI patients (refer to PICO 9). If initiation of pharmacologic VTE prophylaxis is anticipated to be delayed or interrupted, US screening and/or prophylactic IVCF may be considered.

#### Rationale

DVT and PE rates were the highest among SCI managed operatively for vertebral fractures involving more than one level of the spine, followed by isolated lumbar spine injury and thoracic spine injury [55, 59]. We identified four observational studies (*n* = 4330) that addressed this question [59–62]. The pooled estimates of three observational studies (*n* = 786) showed that early pharmacologic VTE prophylaxis associated with reduced VTE (RR 0.41, 95% CI 0.23–0.72; very low quality) [60–62]. These results were limited by serious imprecision due to small sample and/or effect sizes which leads to uncertainty. Similarly, four observational studies (*n* = 4330) showed that early pharmacologic VTE prophylaxis associated with reduced DVT (RR 0.2, 95% CI 0.15–0.28; moderate quality) and reduced PE prophylaxis (RR 0.61, 95% CI 0.38–0.97; very low quality) [59–62]. Regarding the adverse effects, one study (*n* = 3544) reported the need of post-VTE prophylaxis repeated decompressive laminectomy and was not different among those receiving early pharmacologic VTE prophylaxis (RR 0.62; 95% CI 0.33–1.14; very low quality) [59]. Likewise, all-cause mortality and post-prophylaxis blood transfusion were not different among those receiving early pharmacologic VTE prophylaxis (RR 0.79, 95% CI 0.54–1.15; very low quality) and (RR 1.10, 95% CI 0.61–1.97; very low quality), respectively [59–61]. The results were limited by serious indirectness of outcome (need of post-VTE prophylaxis repeated decompressive laminectomy was used as a surrogate marker for intraspinal hematoma), and very serious imprecision. Two studies reported the risk of intraspinal hematoma and epidural hematoma development or expansion after starting early pharmacologic VTE prophylaxis. Kim et al. (*n* = 206) showed no epidural hematoma reported in early (0/48) versus late (0/158) [60]. Chang et al. (*n* = 501) showed no association between early VTE prophylaxis and risk of intraspinal hematoma expansion (HR, 1.90; 95% CI 0.32–11.41) [61]. No study reported clinically significant extracranial bleeding. We also assessed one recently published study which was deemed consistent with the recommendation, and we did not update the meta-analysis [63] (Additional file 2: Tables S13, S14).

Based on the available evidence, it is likely that the benefits of early pharmacologic VTE prophylaxis (moderate reduction of DVT, VTE, and PE) outweigh the small undesirable effects. It likely has no impact on equity, is possibly cost-effective and likely feasible. Moreover, baseline risk of VTE in SCI is perceived to be higher than hemorrhagic risk. Uncertainty remains due to limited reported data of other important outcomes related to adverse effects. The overall quality of evidence was very low. Therefore, we issued a weak recommendation for early pharmacologic VTE prophylaxis within 48 h post-spinal fixation over delayed pharmacologic VTE prophylaxis (> 48 h). Our recommendation is consistent with recommendations by other professional societies [5, 58]. We also highlighted that this area is understudied, and that high-quality RCTs are needed.

### Type of pharmacologic VTE prophylaxis

Question: *In adults with trauma who are prescribed pharmacologic VTE prophylaxis, should we recommend LMWH over UFH?*

#### Recommendation 7

In adults with trauma who receive pharmacologic VTE prophylaxis, we ***suggest*** using LMWH (e.g., enoxaparin, dalteparin) over UFH (weak recommendation, low quality of evidence).

#### Remark

UFH is preferred in patients with end-stage renal disease and in those with low creatinine clearance (< 30 ml/min).

#### Rationale

We reviewed a meta-analysis of eight observational studies (*n* = 30,674) and four RCTs compared LMWH (e.g., enoxaparin, dalteparin) versus UFH for VTE prophylaxis [64]. The pooled estimates of four RCTs (*n* = 785) showed a significant reduction in DVT with LMWH compared to UFH (RR 0.67, 95% CI 0.50–0.88; moderate quality). The pooled estimates of three observational studies showed that LMWH was associated with lower DVT (adjusted odds ratio (aOR) 0.62, 95% CI 0.57–0.66; low quality). Only one RCT reported on PE outcome, the results of which was inconclusive (RR 0.34, 95% CI 0.01–8.29; low quality). The pooled estimates of two observational studies showed that LMWH reduced PE (aOR 0.56, 95% CI 0.50–0.62; low quality). Similarly, the pooled estimates of four RCTs (*n* = 785) and six observational studies showed reduced VTE risk with LMWH (RR 0.68, 95% CI 0.51–0.90; moderate quality) and (aOR 0.71, 95% CI 0.63–0.81; low quality), respectively. LMWH may reduce mortality based on pooled estimates of three observational data (aOR 0.54, 95% CI 0.45–0.65, low quality). RCT data were unclear regarding mortality outcome due to very serious imprecision (RR 0.51, 95% CI 0.05–5.58, low quality).

On the other hand, three RCTs (*n* = 767) reported on major bleeding. The pooled estimate was imprecise and failed to show a clear effect (RR 1.42, 95% CI 0.62–3.24; very low quality). Furthermore, LMWH did not increase the risk adverse events compared to UFH (RR 0.80, 95% CI 0.48 –1.33; low quality). There was an uncertain effect of LMWH compared to UFH on unexpected return to OR (pooled observational data, aOR 0.96, 95% CI 0.80–1.16, very low quality). We also assessed recently published studies (in TBI and orthopedic trauma patients) which were deemed consistent with the recommendation, and we did not update the meta-analysis [65] (Additional file 2: Tables S13, S14).

In summary, the desirable consequences of LMWH probably outweighs the trivial to small undesirable consequences. The use of LMWH probably has no impact on equity and mostly acceptable, feasible, and possibly cost-effective. Altogether, we suggest using LMWH over UFH in adult trauma patients with low risk of bleeding. The WTA recommended enoxaparin for most trauma patients while the AAST recommended using either UFH or LMWH for pharmacologic VTE prophylaxis in patients with TBI [4, 5]. In solid organ injuries, AAST recommended LMWH.

The use of LMWH may be impacted by its renal clearance and concerns about bioaccumulation and potential for increased bleeding. However, previous studies have shown that this is not the case with dalteparin and enoxaparin prophylaxis dosing for critically ill patients [66]. The RCTs included in Tran et al. review excluded patients with renal insufficiency, and some cohort studies did not account for renal dysfunction during confounding adjustment [64]. It is worth noting that the risk of bioaccumulation is not uniform across all LMWHs and varies depending on the patient and the preparation used. A previous study of critically ill patients with severe renal insufficiency receiving dalteparin prophylaxis found that the efficacy of VTE prevention and bleeding risk was related to patient factors rather than drug accumulation. However, for enoxaparin, previous literature has shown that bioaccumulation and bleeding can occur in the setting of severe renal insufficiency [64, 66]. The risks may be minimized by reducing enoxaparin dose and monitoring anti-Xa activity. Alternatively, in patients with renal insufficiency or those on renal replacement therapy, the use of UFH may be a suitable alternative.

### Dose of pharmacologic VTE prophylaxis

Question: *In adults with trauma who are prescribed LMWH (enoxaparin), should using intermediate–high dose versus conventional dosing be recommended?*

#### Recommendation 8

In adults with trauma and low risk of bleeding who are prescribed LMWH (enoxaparin) for VTE prophylaxis, we ***suggest*** using ***either*** intermediate–high dose LMWH or conventional dosing LMWH (weak recommendation, very low quality of evidence).

#### Remarks:

- Most common regimen used was enoxaparin 40 mg subcutaneous every 12 h.
- This recommendation is inapplicable to those at a high risk for bleeding (patients older than 65 year, < 50 kg, have low creatinine clearance, and TBI or SCI patients who are high risk for bleeding) [5, 67].

#### Rationale

In the absence of a standard definition, any dose greater than the standard dose of LMWH prophylaxis (30 mg every 12 h or 40 mg every 24 h) and less than the therapeutic dose was considered as intermediate–high dose. Accordingly, we identified three strategies in the literature; fixed higher initial dosing regimen (40 mg every 12 h), dosing based on anti-Xa level adjustments with dose escalation for subtherapeutic anti-Xa level and weight-adjusted dosing (weight-based and weight-stratified). Our search identified one pilot RCT (*n* = 234) using weight-based dosing enoxaparin versus conventional dosing [67]. Due to the small number of events in this pilot RCT, the results were imprecise for most outcomes. There was a reduction in VTE; however, this was not statistically significant (RR 0.38, 95% CI 0.12–1.13; low quality). In addition, the risk of DVT and PE was non-significantly reduced (RR 0.41, 95% CI 0.13–1.25; low quality) and (RR 0.38, 95% CI 0.02–9.12; low quality), respectively. Similarly, the results for mortality were inconclusive (RR 0.38, 95% CI 0.02–9.12; low quality).

Additionally, we identified four observational studies (*n* = 5180), that examined the use of intermediate–high enoxaparin dose (40 mg every 12 h) versus conventional dose (30 mg every 12 h) [68–71]. The pooled estimates showed that intermediate–high enoxaparin dose was associated with reduced VTE (RR 0.64, 95% CI 0.42–0.97; very low quality) and PE (RR 0.32, 95% CI 0.14–0.76; very low quality). However, the reduction in DVT risk was statistically nonsignificant (RR 0.65, 95% CI 0.37–1.14; very low quality). Three observational studies (*n* = 5111) reported on mortality outcome with unclear benefit (RR 1.14, 95% CI 0.93–1.40; very low quality) [68, 69, 71].

Furthermore, we found two observational studies (*n* = 421) that used anti-Xa level LMWH dosing versus conventional dosing [72, 73]. The effect of using LMWH doses based on anti-Xa levels on VTE and DVT risk is uncertain, as the available evidence suggests a possible reduction or increase in VTE risk (RR 0.53, 95% CI 0.05–5.71; very low quality) and DVT risk (RR 0.33, 95% CI 0.07–1.55; very low quality). However, these results are limited by very serious imprecision, and the 95% CI encompasses a wide range of possible differences, making it difficult to draw definitive conclusions about the direction of effect.

Regarding the risk of bleeding, the results were unclear from pilot RCT result comparing weight-based dosing enoxaparin versus conventional dosing (RR 0.38, 95% CI 0.02–9.12; low quality) and two observational studies (*n* = 292) using intermediate–high dosing (RR 0.84, 95% CI 0.33–2.13; very low quality) [67, 70, 71].

We acknowledged the publication of recent SRMAs after completing our meta-analysis (Additional file 2: Tables S13, S14) [74]. The results were assessed and deemed consistent with the recommendation. Conflicting data regarding anti-Xa-based dosing of LMWH and VTE rates may be due to difficulty in obtaining appropriately timed anti-Xa levels [74].

In summary, the existing evidence on intermediate–high dose LMWH is of very low quality. The costs of intermediate–high dosing are higher than those of conventional dosing. Intermediate–high dosing probably has no impact on equity and is probably acceptable to key stakeholders. However, feasibility varies according to availability of an anti-Xa assay which is likely not available in low resource settings (anti-Xa assay use is debatable and the lack of it does not preclude the use of intermediate dose). Therefore, we issued a weak recommendation to use either intermediate–high dose or conventional dosing LMWH. Future studies focusing on patient-centered outcomes such as VTE, mortality, and major bleeding are warranted. The WTA guidelines suggested using enoxaparin 40 mg every 12 h for most trauma patients. However, for patients with spine and brain trauma, they suggested 30 mg every 12 h and to adjust the dose according to anti-Xa levels [5].

### Mechanical VTE prophylaxis

Question: *In adults with trauma who are not candidates for pharmacologic VTE prophylaxis, should we recommend mechanical VTE prophylaxis with IPC versus no mechanical VTE prophylaxis?*

#### Recommendation 9

In adults with trauma who are not candidates for pharmacologic VTE prophylaxis, we ***recommend*** using mechanical VTE prophylaxis with IPC over no mechanical VTE prophylaxis when not contraindicated by lower extremity injury (strong recommendation, very low quality evidence).

#### Rationale

Mechanical VTE prophylaxis is a form of thromboprophylaxis and acts to prevent venous stagnation in the lower limbs by promoting venous outflow. Mechanical VTE prophylaxis includes graduated compression stockings, IPC devices/sequential compression devices and A–V foot pumps [2]. Unlike pharmacologic VTE prophylaxis, mechanical VTE prophylaxis is not associated with bleeding. Antiembolism stockings (thrombo-embolus deterrent stockings or compression stockings) are not as effective as IPC devices [75–77]. Accordingly, we only addressed IPC in our guidelines.

We identified three relevant RCTs (*n* = 860) and two observational studies (*n* = 272) [78–80]. A meta-analysis of three RCTs demonstrated no difference in mortality among patients who received mechanical VTE prophylaxis or not (RR 0.80, 95% CI 0.06–10.34, very low quality) [78–80]. Results were limited by serious inconsistency supported by differences in point estimates and high *I*^2^ values (57%) and very serious imprecision.

One observational study (*n* = 240) showed that mechanical VTE prophylaxis is associated with lower risk of VTE (RR 0.34, 95% CI 0.19–0.60, very low quality) [81]. Moreover, the pooled estimates of two observational studies (*n* = 272) showed lower risk of DVT with mechanical prophylaxis (RR 0.39, 95% CI 0.20–0.77, very low quality) [81, 82]. Results were limited by serious risk of bias and serious imprecision. The pooled estimates of three RCTs (*n* = 860) showed reduced risk of DVT with mechanical VTE prophylaxis as compared to no mechanical VTE prophylaxis (RR 0.46, 95% CI 0.23–0.9, low quality) [78–80]. Results were limited by serious inconsistency supported by differences in point estimates and high *I*^2^ value (63%), and serious imprecision.

The pooled estimates across three RCTs (*n* = 860) showed lower risk of PE with mechanical VTE prophylaxis, however, the 95% CI could not exclude increased risk (RR 0.71, 95% CI 0.30–1.67, low quality) [78–80]. Similarly, the pooled estimate across two observational studies (*n* = 272) did not show an association between mechanical VTE prophylaxis and lower PE (RR 0.73, 95% CI 0.07–8.03, very low quality) [81, 82]. Results were limited by serious risk of bias, inconsistency supported by differences in point estimates and high *I*^2^ value (84%), and very serious imprecision.

In terms of adverse effects, two RCTs (*n* = 556) and one observational study (*n* = 240) reported zero bleeding events in both groups [78, 79, 81]. No other adverse events were reported in these studies. However, based on indirect comparison (combined mechanical VTE prophylaxis and pharmacologic VTE prophylaxis versus pharmacologic VTE prophylaxis alone), mechanical VTE prophylaxis was associated with a small nonsignificant increase in leg skin injury [83].

The balance between desirable and undesirable effects probably favors mechanical VTE prophylaxis over no mechanical VTE prophylaxis for patients who are not candidate for pharmacologic VTE prophylaxis. The use of mechanical VTE prophylaxis likely has no impact on equity, is probably cost-effective, and is probably acceptable to stakeholders and feasible to implement. It should be recognized that many trauma patients considered at moderate-to-high risk for VTE would receive mechanical VTE prophylaxis especially in case of contraindication to pharmacologic one.

Our recommendation is consistent with other professional societies’ recommendations for major trauma patients with contraindication to pharmacologic VTE prophylaxis due to active bleeding [4, 5, 84].

Question: *In adults with trauma on pharmacologic VTE prophylaxis, should we recommend adding mechanical VTE prophylaxis (IPC) versus pharmacologic VTE prophylaxis alone?*

#### Recommendation 10

In adults with trauma taking pharmacologic VTE prophylaxis, we ***suggest either*** using adjunct mechanical VTE prophylaxis or pharmacologic VTE prophylaxis alone (weak recommendation, very low quality evidence).

#### Rationale

Our search identified five RCTs, and one observational study [76, 83, 85–89]. Pooled estimates of the five RCTs (*n* = 2984) showed no clear mortality benefit from combined mechanical and pharmacologic VTE prophylaxis compared to pharmacological prophylaxis alone (RR 0.88, 95% CI 0.72–1.08, low quality) [83, 85–88]. Results were limited by serious indirectness and imprecision.

Pooled estimates of two RCTs (*n* = 2184) and one observational study (*n* = 618) showed no difference in VTE outcome between combined mechanical and pharmacologic VTE prophylaxis compared to pharmacologic VTE prophylaxis alone (RR 1.13, 95% CI 0.88–1.45, low quality from 2 RCTs and RR 0.67, 95% CI 0.34–1.31, very low quality from one observational study) [76, 83, 85]. Both results were limited by serious indirectness and imprecision.

Pooled estimates of five RCTs (*n* = 2617) showed no difference in DVT outcome between combined mechanical and pharmacologic VTE prophylaxis compared to pharmacologic VTE prophylaxis alone, however, the 95% CI could not exclude possible reduction in DVT (RR 0.65, 95% CI 0.37–1.14, very low quality) [83, 85–88]. A meta-analysis of five RCTs (*n* = 2691) showed no difference in PE risk (RR 1.02, 95% CI 0.40–2.62, very low quality) [83, 85–88].

In terms of side effects, one RCT (*n* = 2003) reported a similar risk of lower extremity skin injury between the two groups; 2.9% in combined mechanical and pharmacologic VTE prophylaxis compared to 2.8% in pharmacologic VTE prophylaxis alone (RR 1.06, 95% CI 0.63–1.76, low quality) [83, 89]. Based on pooled estimates of two RCTs (*n* = 676), both groups had similar risks of major and minor bleeding [85, 86]. We also assessed one recently published retrospective study and SR in patients undergoing high-risk procedures (including trauma) and the authors concluded that combined mechanical and pharmacological VTE prophylaxis reduced DVT (OR 0.38, 95% CI 0.21–0.70, high quality) and PE risks (OR 0.46, 95% CI 0.3–0.71, low quality) [90, 91] (Additional file 2: Tables S13, S14).

There is uncertainty about the balance between the desirable and undesirable effects of both approaches. Most of the included studies comprised a mixed population and the PREVENT study (largest RCT) was performed in a wide variety of critically ill patients (medical and surgical). Trauma patients accounted only for about 8% in both group [83]. The use of mechanical VTE prophylaxis likely has no impact on equity, is probably cost-effective, and is probably acceptable and feasible. We recognized that trauma patients are at high risk for VTE and combined prophylaxis is probably favored over either mechanical or pharmacologic VTE prophylaxis alone. The 2020 WTA guidelines encouraged combining mechanical with pharmacologic VTE prophylaxis for moderate-to-high VTE risk trauma patients [5].

### Routine duplex US surveillance

Question: *In adults with trauma who are not candidates for pharmacologic VTE prophylaxis, should we recommend routine VTE US screening versus no routine screening?*

#### Recommendation 11

In adults with trauma who are at an elevated risk of VTE and are not candidates for pharmacologic VTE prophylaxis, we ***suggest*** routine bilateral lower extremity US to screen for asymptomatic DVT over no routine screening (weak recommendation, very low quality evidence).

#### Remark

This recommendation is inapplicable to trauma patients who are ambulating, those at low VTE risk, and patients with signs or symptoms of DVT in whom diagnostic imaging is indicated.

#### Rationale

The data for screening of asymptomatic patients for DVT are conflicting and these practices vary widely among trauma centers. Pooled estimates across observational studies suggest higher odds of DVT and lower odds of PE with routine US surveillance compared to no surveillance [92–95]. The PREVENT sub-study showed an association between US surveillance and lower 90-day mortality (HR 0.75; 95% CI 0.57–0.99; very low quality) [95]. These results were limited by indirectness as trauma patients accounted for 8% in both groups.

Given the concerns of residual confounding in observational data, we assessed one available RCT (*n* = 1989), in which routine US surveillance group had higher risks of distal DVT (RR 15.48, 95% CI 7.62–31.48; low quality) and proximal DVT (RR 2.37, 95% CI 1.04–5.39; very low quality) [96]. There was significantly fewer in-hospital PE (RR 0.11, 95% CI 0.01–0.87; very low quality) and no difference in 90-day and in-hospital mortality (RR 0.83, 95% CI 0.59–1.18; low quality and RR 0.73 95% CI 0.44–1.22; very low quality), respectively [96]. There was no difference in major bleeding rate for those who received anticoagulation for treatment of DVT [96].

Detection of more DVTs in US screening group may allow early diagnosis, and prevention of DVT propagation and embolization [96]. Additionally, US screening could be justified based on the high baseline prevalence of symptomatic and asymptomatic DVT in trauma patients (approximately 58% without pharmacologic VTE prophylaxis and 28% with mechanical and pharmacologic VTE prophylaxis) [2]; therefore, the benefits of screening likely outweigh the downsides related to overdiagnosis or overtreatment. In addition, US is a non-invasive test and is widely available, likely has no impact on equity, is probably acceptable, and is possibly cost-effective and feasible. Considering the very low quality evidence, along with the resource implications, we issued a weak favoring routine US screening in this population. Our recommendation is consistent with recommendations given by other professional societies [5, 58]. The frequency of screening is resource dependent, but a reasonable frequency once or twice weekly. We also highlighted that this area is understudied, and that high-quality RCTs are needed.

### Prophylactic IVCFs

Question: *In adults with trauma who are not candidates for pharmacologic VTE prophylaxis, should we recommend using prophylactic IVCF versus no prophylactic IVCF?*

#### Recommendation 12

In adults with trauma who are not candidates for pharmacologic VTE prophylaxis, we ***suggest against*** the routine placement of prophylactic IVCFs (weak recommendation, very low quality evidence).

#### Remark

Clinicians may consider using temporary retrievable IVCF in patients who are expected to be off pharmacologic VTE prophylaxis for ≥ 7 days (e.g., severely injured patients with an ongoing bleeding risk).

#### Rationale

IVCFs have been used in patients at high risk for VTE and concurrent contraindication to pharmacologic VTE prophylaxis, mainly to prevent PE. Prophylactic IVCF are placed in patients who have no evidence of VTE. Nevertheless, the efficacy and safety of this approach in trauma patients remain unclear. The pooled estimate from RCTs demonstrated no significant difference between the prophylactic IVCF group and the control group in mortality (RR 1.44; 95% CI 0.86–2.43; low quality), PE (RR 0.27; 95% CI 0.06–1.28; low quality), and DVT (RR 1.18; 95% CI 0.58–2.40; low quality) [97–99]. Similarly, pooled estimates from observational studies demonstrated no clear association between the use of prophylactic IVCFs and the risks of mortality (RR 0.63; 95% CI, 0.3–1.31; very low quality) or DVT (RR 1.65; 95% CI 0.85–3.2; very low quality) [100–103]. However, the use of IVCF was associated with lower risks of PE (RR 0.25; 95% CI 0.12–0.55; very low quality) and fatal PE (RR 0.09; 95% CI 0.01–0.81; very low quality) when compared to not using IVCFs [100–106].

The largest RCT (*n* = 240) showed no clear effect on a composite outcome of PE or death at 90 days (HR 0.99; 95% CI 0.51–1.94) [97]. Nonetheless, among subgroup of patients who did not receive pharmacologic VTE prophylaxis in the first 7 days, IVCF use reduced the risk of symptomatic PE (RR 0; 95% CI 0.00–0.55) [97].

Inserting IVCFs maybe limited by technical challenges (e.g., angulation/tilting and filter migration) and maybe associated with post-procedural complications (e.g., penetration, infection, and thrombosis). Retrieval and follow-up care of IVCFs are crucial as early as when pharmacologic VTE prophylaxis starts since delayed removal increases time-related complications (e.g., inferior vena cava perforation, IVCF thrombus, and migration). Furthermore, the routine use of IVCFs may increase the healthcare system’s economic burden and reduce health equity due to the associated-cost and required resources.

Considering the low-quality evidence, lack of clear effect on mortality, and potential complications, we issued a weak recommendation against the routine use of prophylactic IVCFs in this population. Our recommendation is consistent with that of the Society of Interventional Radiology Guidelines [107]. Moreover, in patients undergoing major surgery for trauma, the American Society of Hematology (ASH) issued a similar recommendation [77]. Our panel felt that retrievable IVCFs should be restricted to a select group of patients and should take into consideration the desirable and undesirable effects when making individualized decisions on a case-by-case basis. We also highlighted that this area is understudied, and that high-quality RCTs are needed.

## Discussion

The panel acknowledges that some patients, depending on specific characteristics and clinical circumstances, may require individualized approaches and that this warrants deviation from the recommendations; hence these recommendations cannot completely replace expert bedside clinical judgment.

The strengths of these guidelines are the inclusion of diverse panel members, rigorous GRADE methodology adherence, consistent use of rapid SRs, inclusion of a public panel member to provide a patient’s perspective, and the use of a formal EtD framework for every recommendation which took into consideration factors such as clinical effects, quality of evidence, resource use, variation in patient and clinician values, and the acceptability and feasibility of implementation. Thereby, enhanced transparency regarding the judgments made.

The guidelines, however, are not without limitations. One major challenge we encountered was the ambiguity surrounding the definition of “early” versus “delayed” initiation of VTE prophylaxis. This lack of a nuanced definition was due in part to limited prospective evidence on the topic. Furthermore, the definition of “early” or “late” VTE prophylaxis may vary depending on the injury type. In cases where there is an ongoing risk of hemorrhage or the injury occurs in a confined space, clinicians must consider the potential consequences of bleeding or hematoma expansion when determining the timing of early VTE prophylaxis [20]. While the existing literature supports early initiation of pharmacologic VTE prophylaxis for severely injured patients, further prospective studies are needed. For non-operatively managed blunt solid organ injuries and isolated spine trauma with or without SCI, early initiation is most frequently defined as 24–48 h from initial trauma. This time frame is supported by basic science studies that indicate patients transition from hypocoagulable to hypercoagulable state approximately 48 h after injury. However, the existing data on TBIs and the optimal time for VTE prophylaxis initiation are relatively sparse, and clinical equipoise persists. In cases of TBI, a time cut-off of 72 h is most frequently used in literature [20]. Another limitation of our work is that our SRs were not registered a priori, as we adopted a rapid SR approach, commonly used in guidelines methodology [108]. Additionally, the evidence supporting VTE prophylaxis in adults with trauma does not provide high quality of effects for many critical and important outcomes, and based upon confounded observational evidence, we identified limited number of RCTs. Some studies evaluated critically ill patients and trauma patients accounted for 8% of included population, resulting in gaps in areas where the panel extrapolated from indirect evidence to develop a general recommendation [83, 91]. The panel also recognized that many studies for VTE prophylaxis in trauma were old and may have included outdated means of VTE diagnosis (e.g., venography). Moreover, the clinical practice has evolved over time with more emphasis in early mobilization for trauma with minor injuries whenever possible. It is uncertain whether such changes in practice impact the relative effectiveness of various prophylactic measures. There is an urgent need for high-quality evidence to guide clinicians involved in the care of adults with trauma [109–113]. Table 2 shows a summary of identified research priorities.

**Table 2:** Research priorities identified by the panel

Section	Research priorities	Ongoing studies
Timing of pharmacologic VTE prophylaxis in non-operative blunt solid organ injuries	• Further high-quality RCTs or prospective multi-center studies are important to provide greater certainty comparing early pharmacological VTE prophylaxis versus late prophylaxis in patients with solid organ injuries • Future studies shall consider reporting direct clinical outcome (bleeding including major and minor such as hematoma requiring evacuation or retroperitoneal bleeding) rather than reporting post-prophylaxis blood transfusion as surrogate markers of bleeding complications	None identified
Timing of pharmacologic VTE prophylaxis in TBI [102]	Isolated blunt TBI with low risk of bleeding progression: • Further high-quality RCTs with adequate power are important to provide greater certainty comparing early pharmacological VTE prophylaxis versus late prophylaxis in patients with TBI and low risk of ICH progression. There is a plan for a definitive follow-up RCT in the low-risk Parkland arm (DEEP II) • Future research could focus on clarifying what characteristics of TBI low risk of ICH progression utilizing Brain Injury Guideline criteria and Parkland criteria and using more practical cut-off for definition of early pharmacological VTE prophylaxis • Future research could focus on correlating radiographic TBI progression with clinical neurologic status as dichotomizing TBI radiographically as either having “progressed” or “not progressed” may fail to quantify the degree of expansion and association with clinical sequelae • Future research is needed on cost-effectiveness studies in Saudi Arabia	There is currently a prospective RCT completed recruitment which is looking at the timing of pharmacological VTE prophylaxis in TBI comparing early (36–48 h) versus late (> 96 h) initiation of pharmacological VTE prophylaxis (OPTTICH trial) ClinicalTrials.gov Identifier: NCT01589393
	Isolated blunt TBI with high risk of bleeding progression: • Further high-quality RCTs with adequate power are important to provide greater certainty comparing early pharmacological VTE prophylaxis versus late prophylaxis in patients with TBI and high risk of ICH progression	There is a plan for pilot RCT on the medium Parkland arm known as delayed Versus Early Enoxaparin Prophylaxis III study (DEEP III)
	TBI requiring neurosurgical intervention: • Further high-quality RCTs with adequate power are important to provide greater certainty comparing early pharmacological VTE prophylaxis versus late prophylaxis in patients with TBI requiring ICP monitoring or EVD or craniotomy or craniectomy	None identified
Timing of pharmacologic VTE prophylaxis for spine trauma or fracture and/or SCI [102]	• Further high-quality RCTs with adequate power are important to provide greater certainty comparing early pharmacological VTE prophylaxis versus late prophylaxis in patients with SCI and managed non-operatively and operatively • Future studies shall consider reporting direct outcome (intraspinal hematoma, epidural hematoma development or expansion after starting VTE prophylaxis) rather than using surrogate outcomes • More studies are needed to evaluate the subsets of spine-injured patients who require more or less aggressive approaches to early VTE prophylaxis initiation and the impact by spine segment involved (cervical, thoracic, or lumbar), or completeness of injury (complete versus incomplete SCI) • Pharmacoeconomic studies are needed to compare the cost-effectiveness between the two arms • More studies are needed to evaluate the need for routine follow-up imaging to check for hematoma expansion after initiation of VTE prophylaxis and MRI role in the diagnosis or exclusion of epidural hematoma prior to VTE prophylaxis	None identified
Type of pharmacologic VTE prophylaxis	• Bleeding complications in trauma patients is limited by inconsistent and non-validated bleeding definitions. Clinically meaningful outcomes are required for future research • The commonly used enoxaparin 30 mg subcutaneously twice a day regimen which is considered to be an underdosing (new WTA guideline suggests enoxaparin 40 mg subcutaneously twice a day for VTE prophylaxis [5]). Therefore, true comparative studies are needed with this new dosing regimen regarding efficacy for VTE prevention and safety	None identified
Dose of pharmacologic VTE prophylaxis	• More studies are needed focusing on patient-centered outcomes (VTE, mortality) as primary outcome rather than using surrogate marker (on-target anti-Xa level) as primary outcome and differentiate between major and minor bleeding complications when reporting results • Further investigations are needed to determine specific patient subgroups most likely to benefit from intermediate dose prophylaxisCost-effectiveness analysis studies are also needed • Implications of ATIII activity in VTE formation	There is an ongoing study investigating weight-based enoxaparin dosing in trauma patients would provide valuable information once completed ClinicalTrials.gov Identifier: NCT01916707
Mechanical VTE prophylaxis	• Further high-quality comparative studies in trauma patients using appropriate clinical outcomes would be of value to add more certainty to recommendation • Studies enabling identification of baseline risk would be valuable to identify patients particularly likely to benefit from combined prophylaxis strategies • The duration of compression (hours per day) needed for VTE prevention with IPC; device standardization • Need for economics study to assess the cost-effectiveness of these interventions	There is an ongoing study investigating sequential compression device versus combined sequential compression device and dalteparin in TBI patients ClinicalTrials.gov Identifier: NCT03559114
Routine duplex ultrasonography (US) surveillance [103]	• More RCTs with possible adaptive study design and Bayesian statistical methods are needed for comparing effectiveness of surveillance US versus prophylactic inferior vena cava filter versus combination approach to prevent fatal PE as clinically important VTE outcomes • More study is needed to define the frequency of surveillance US • Further investigation is needed to further characterize the method of VTE risk assessment in determining which subpopulations will benefit most from routine surveillance • More studies are needed for the utility of upper extremities and neck US screening	There is currently an ongoing RCT (Diagnosing Deep-vein Thrombosis Early in Critically ill Patients “DETECT” trial) comparing surveillance US for lower limb DVT in high-risk medical-surgical ICU patients to a clinician-directed approach ClinicalTrials.gov Identifier: NCT05112705
Prophylactic IVCFs	• Cost-effectiveness analysis is probably needed in Saudi Arabia to determine if costs of using prophylactic filter offset by the savings from having a lower incidence of symptomatic PE and its complications • Newly developed, absorbable IVC filters, which are absorbed over a 32-week period, have demonstrated an ability to prevent PE for at least 5 weeks after placement in a swine mode. RCT in humans is needed • RCT with adequate power is probably also needed for evaluating the effect of retrievable IVC filter in reducing PE-related death rather than composite outcome of PE or death or reporting all-cause of mortality	None identified
Other areas [104–106]	• In trauma patients, PE frequently occurs in the absence of DVT (are not embolic) and is thought to originate de novo in the lungs (pulmonary thrombosis) as a result of inflammation, endothelial injury, and the hypercoagulable state caused by the injury itself. More studies are needed to evaluate the impact of available VTE prophylaxis strategies in prevention of this distinct clinical entity	None identified

AT III, antithrombin III; EVD, external ventricular drain; DVT, deep vein thrombosis; ICH, intracranial hemorrhage; ICP, intracranial pressure; IPC, intermittent pneumatic compression; IVCF, inferior vena cava filters; LMWH, low molecular weight heparin; MRI, magnetic resonance imaging; PE, pulmonary embolism; RCT, randomized controlled trials; SCI, spinal cord injury; TBI, traumatic brain injury; UFH, unfractionated heparin; US, Ultrasonography; VTE, venous thromboembolism

### Results from the most recent guidelines

The ASH 2019 guidelines in surgical hospitalized patients were limited to two recommendations for VTE prophylaxis in major trauma [77]. The ASH guidelines suggest using pharmacologic prophylaxis over no pharmacologic prophylaxis for patients experiencing major trauma and who are at low-to-moderate risk for bleeding. The ASH guidelines suggest using LMWH or UFH in patients experiencing major trauma in whom pharmacologic VTE prophylaxis is used.

A widely used high-quality guidelines are the WTA 2020 guidelines [5], and the 2022 clinical protocol developed by the AAST and the American College of Surgeons—Committee on Trauma [58] which places an emphasis on patients’ VTE risk scores, e.g., patient with an Injury Severity Score (ISS) of ≥ 10 suggests that pharmacologic VTE prophylaxis should be initiated as soon as possible, whereas patients with an ISS of < 10 are at a lower risk of VTE and may not require pharmacologic prophylaxis. Because ISS is not calculated in real time, the Greenfield Risk Assessment Profile or the Trauma Embolic Scoring System can assist with calculating VTE risk [114–116]. While scoring systems are helpful for stratifying risk, most trauma patients with major injuries that require hospitalization are at increased risk of VTE. Therefore, AAST and the American College of Surgeons—Committee on Trauma recommended that pharmacologic VTE prophylaxis should be initiated promptly without the need for formal risk scoring, unless the patient is ambulatory and has an expected length of stay < 24 h [58].

#### Plan for guidelines adaptation and updating

The SCCS will determine the need for future updates based on emerging evidence and changing priorities. We will consider addressing the role of direct oral anticoagulants and low-dose aspirin for VTE prophylaxis in isolated orthopedic injuries, thrombo-elastography with platelet mapping guided VTE prophylaxis dosing, and VTE prophylaxis in a special trauma population (pregnant patients). Management of pharmacologic VTE prophylaxis in trauma with epidural catheter should follow the general guidance from regional anesthesia guidelines [117]. The EtD framework may also serve as the basis for adaptation of these recommendations in different context by a local, regional, or international guidelines panel.

## Conclusion

The SCCS guidelines provide guidance for clinicians involved in the care of hospitalized adults with trauma. The panel members generated 12 clinical practice recommendations related to VTE prophylaxis in adults with trauma (1 strong recommendation, 10 weak recommendations, and identified one PICO question with insufficient evidence to make a recommendation) and identified areas where further research is needed.

## Supplementary Information

**Additional file 1:** Appendix 1. AGREE Reporting Checklist

**Additional file 2:** Appendix 2.** Table S1**. Selection and Organization of Committee Members. Search Strategy.** Table S2.** Implications of different recommendations to key stakeholders.** Tables S3,** S5, S7-S12. PICO questions.** Table S4.** Definition of bleeding risk in blunt solid organ injuries.** Table S6.** Evidence to Decision Framework Recommendation 4: TBI requiring neurosurgicalintervention. Evidence Profiles and Evidence to Decision Frameworks for each PICO. Meta-analyses for each PICO.** Table S13. **Results for studies identified after conclusion of SR and guidelines panel recommendations.** Table S14.** Summary findings for studies identified after conclusion of SR and guidelines panelrecommendations.

**Additional file 3:** Appendix 3. Management of conflict of interests

## Data Availability

The datasets are available from the corresponding author on reasonable request.

## Abbreviations

AAST: American Association for the Surgery of Trauma
aOR: Adjusted odds ratio
ASH: American Society of Hematology
BPS: Best practice statement
CI: Confidence intervals
COI: Conflict of interests
DEEP I: Delayed Versus Early Enoxaparin Prophylaxis I study
DVT: Deep-vein thrombosis
EtD: Evidence-to-decision
EVD: External ventricular drain
GRADE: Grading Recommendations, Assessment, Development, and Evaluation
GUIDE: Guidelines in Intensive Care Development and Evaluation
ICH: Intracranial hemorrhage
ICP: Intracranial pressure
IPC: Intermittent pneumatic compression
ISS: Injury Severity Score
IVCF: Inferior vena cava filters
LMWH: Low molecular weight heparin
NCS: Neurocritical Care Society
OR: Odds ratios
PE: Pulmonary embolism
PICO: Population, intervention, comparison, and outcome(s)
PREVENT: Pneumatic Compression for Preventing Venous Thromboembolism trial
RCT: Randomized controlled trials
RR: Relative risk
SCCS: Saudi Critical Care Society
SCI: Spinal cord injury
SR: Systematic review
TBI: Traumatic brain injury
UFH: Unfractionated heparin
US: Ultrasonography
VTE: Venous thromboembolism
WTA: Western Trauma Association

## References

[1] World Health Organization (2018). Global health estimates 2016: deaths by cause, age, sex, by country and by region, 2000–2016.

[2] Moore EE, Moore HB, Kornblith LZ, Neal MD, Hoffman M, Mutch NJ (2021). Trauma-induced coagulopathy. Nat Rev Dis Primers.

[3] Rogers FB, Cipolle MD, Velmahos G, Rozycki G, Luchette FA (2002). Practice management guidelines for the prevention of venous thromboembolism in trauma patients: the EAST practice management guidelines work group. J Trauma.

[4] Rappold JF, Sheppard FR, Carmichael SP, Cuschieri J, Ley E, Rangel E (2021). Venous thromboembolism prophylaxis in the trauma intensive care unit: an American Association for the Surgery of Trauma Critical Care Committee Clinical Consensus Document. Trauma Surg Acute Care Open.

[5] Ley EJ, Brown CV, Moore EE, Sava JA, Peck KA, Ciesla DJ (2020). Updated guidelines to reduce venous thromboembolism in trauma patients: a Western Trauma Association critical decisions algorithm J. Trauma Acute Care Surg.

[6] Guyatt GH, Oxman AD, Vist GE, Kunz R, Falck-Ytter Y, Alonso-Coello P, Schunemann HJ, Group GW (2008). GRADE: an emerging consensus on rating quality of evidence and strength of recommendations. BMJ.

[7] Amer M, Bawazeer M, Maghrabi K, Amin R, De Vol E, Hijazi M (2020). Timing and dose of pharmacological thromboprophylaxis in adult trauma patients: perceptions, barriers, and experience of Saudi Arabia practicing physicians. Saudi Surg J.

[8] Schunemann HJ, Wiercioch W, Etxeandia I, Falavigna M, Santesso N, Mustafa R (2014). Guidelines 2.0: systematic development of a comprehensive checklist for a successful guideline enterprise. CMAJ.

[9] Brouwers MC, Kerkvliet K, Spithoff K, AGREE Next Steps Consortium (2016). The AGREE Reporting Checklist: a tool to improve reporting of clinical practice guidelines. BMJ.

[10] Alhazzani W, Lewis K, Jaeschke R, Rochwerg B, Moller MH, Evans L (2018). Conflicts of interest disclosure forms and management in critical care clinical practice guidelines. Intensive Care Med.

[11] Alhazzani W, Alshahrani M, Alshamsi F, Aljuhani O, Eljaaly K, Hashim S (2022). The Saudi Critical Care Society practice guidelines on the management of COVID-19 in the ICU: therapy section. J Infect Public Health.

[12] Guyatt GH, Oxman AD, Kunz R, Atkins D, Brozek J, Vist G (2011). GRADE guidelines: 2. Framing the question and deciding on important outcomes. J Clin Epidemiol.

[13] Covidence systematic review software, Veritas Health Innovation, Melbourne, Australia. www.covidence.org. Accessed 22 Aug 2022.

[14] Higgins JP, Thomas J, Chandler J, Cumpston M, Li T, Page MJ, Welch VA (2019). Cochrane handbook for systematic reviews of interventions.

[15] Guyatt GH, Oxman AD, Vist G, Kunz R, Brozek J, Alonso-Coello P (2011). GRADE guidelines: 4. Rating the quality of evidence–study limitations (risk of bias). J Clin Epidemiol.

[16] Manager R. Book version 5.4. The Nordic Cochrane Centre, The Cochrane Collaboration, Copenhagen. 2014.

[17] Cumpston M, Li T, Page MJ, Chandler J, Welch VA, Higgins JP (2019). Updated guidance for trusted systematic reviews: a new edition of the Cochrane Handbook for Systematic Reviews of Interventions. Cochrane Database Syst Rev.

[18] Guyatt GH, Schunemann HJ, Djulbegovic B, Akl EA (2015). Guideline panels should not GRADE good practice statements. J Clin Epidemiol.

[19] Cimbanassi S, Chiara O, Leppaniemi A, Henry S, Scalea TM, Shanmuganathan K (2018). Nonoperative management of abdominal solid-organ injuries following blunt trauma in adults: results from an International Consensus Conference. J Trauma Acute Care Surg.

[20] Schellenberg M, Costantini T, Joseph B, Price MA, Bernard AC, Haut ER (2023). Timing of venous thromboembolism prophylaxis initiation after injury: findings from the consensus conference to implement optimal VTE prophylaxis in trauma. J Trauma Acute Care Surg.

[21] Murphy PB, de Moya M, Karam B, Menard L, Holder E, Inaba K (2022). Optimal timing of venous thromboembolic chemoprophylaxis initiation following blunt solid organ injury: meta-analysis and systematic review. Eur J Trauma Emerg Surg.

[22] Skarupa D, Hanna K, Zeeshan M, Madbak F, Hamidi M, Haddadin Z (2019). Is early chemical thromboprophylaxis in patients with solid organ injury a solid decision?. J Trauma Acute Care Surg.

[23] Gaitanidis A, Breen KA, Nederpelt C, Parks J, Saillant N, Kaafarani HMA (2021). Timing of thromboprophylaxis in patients with blunt abdominal solid organ injuries undergoing nonoperative management. J Trauma Acute Care Surg.

[24] Moore K, Barton CA, Wang Y, Ran R, Che A, Rowell S (2022). Early initiation of thromboembolic prophylaxis in critically ill trauma patients with high-grade blunt liver and splenic lacerations is not associated with increased rates of failure of non-operative management. Trauma.

[25] Pastorek RA, Cripps MW, Bernstein IH, Scott WW, Madden CJ, Rickert KL (2014). The Parkland Protocol’s modified Berne-Norwood criteria predict two tiers of risk for traumatic brain injury progression. J Neurotrauma.

[26] Joseph B, Friese RS, Sadoun M, Aziz H, Kulvatunyou N, Pandid V (2014). The BIG (brain injury guidelines) project: defining the management of traumatic brain injury by acute care surgeons. J Trauma Acute Care Surg.

[27] Phelan HA, Wolf SE, Norwood SH (2012). A randomized, double-blinded, placebo-controlled pilot trial of anticoagulation in low-risk traumatic brain injury. J Trauma Acute Care Surg.

[28] Salottolo K, Offner P, Levy AS, Mains CW, Slone DS, Bar-Or D (2011). interrupted pharmocologic thromboprophylaxis increases venous thromboembolism in traumatic brain injury. J Trauma Inj Infect Crit Care.

[29] Depew AJ, Hu CK, Nguyen AC, Driessen N (2008). Thromboembolic prophylaxis in blunt traumatic intracranial hemorrhage: a retrospective review. Am Surg.

[30] Koehler DM, Shipman J, Davidson MA, Guillamondegui O (2011). Is early venous thromboembolism prophylaxis safe in trauma patients with intracranial hemorrhage. J Trauma Inj Infect Crit Care.

[31] Kim J, Gearhart MM, Zurick A, Zuccarello M, James L, Luchette FA (2002). Preliminary report on the safety of heparin for deep venous thrombosis prophylaxis after severe head injury. J Trauma Inj Infect Crit Care.

[32] Rivas L, Vella M, Ju T, Fernandez-Moure JS, Sparks A, Seamon MJ (2022). Early chemoprophylaxis against venous thromboembolism in patients with traumatic brain injury. Am Surg.

[33] Frisoli FA, Shinseki M, Nwabuobi L, Zeng XL, Adrados M, Kanter C (2017). Early venous thromboembolism chemoprophylaxis after traumatic intracranial hemorrhage. Neurosurgery.

[34] Saadi R, Brandt K, Madlinger R, Nerenberg SF (2021). Assessment of the use of pharmacologic venous thromboembolism prophylaxis in post-traumatic brain injury patients. J Pharm Pract.

[35] Saadeh Y, Gohil K, Bill C, Smith C, Morrison C, Mosher B (2012). Chemical venous thromboembolic prophylaxis is safe and effective for patients with traumatic brain injury when started 24 hours after the absence of hemorrhage progression on head CT. J Trauma Acute Care Surg.

[36] Levy AS, Salottolo K, Bar-Or R (2010). Pharmacologic thromboprophylaxis is a risk factor for hemorrhage progression in a subset of patients with traumatic brain injury. J Trauma.

[37] Norwood SH, Berne JD, Rowe SA, Villarreal DH, Ledlie JT (2008). Early venous thromboembolism prophylaxis with enoxaparin in patients with blunt traumatic brain injury. J Trauma.

[38] Scudday T, Brasel K, Webb T, Codner P, Somberg L, Weigelt J (2011). Safety and efficacy of prophylactic anticoagulation in patients with traumatic brain injury. J Am Coll Surg.

[39] Norwood SH, McAuley CE, Berne JD, Vallina VL, Kerns DB, Grahm TW (2002). Prospective evaluation of the safety of enoxaparin prophylaxis for venous thromboembolism in patients with intracranial hemorrhagic injuries. Arch Surg.

[40] Phelan HA, Eastman AL, Madden CJ, Aldy K, Berne JD, Norwood SH (2012). TBI risk stratification at presentation: a prospective study of the incidence and timing of radiographic worsening in the Parkland Protocol. J Trauma Acute Care Surg.

[41] Nyquist P, Bautista C, Jichici D, Burns J, Chhangani S, DeFilippis M (2016). Prophylaxis of venous thrombosis in neurocritical care patients: an evidence-based guideline: a statement for healthcare professionals from the Neurocritical Care Society. Neurocrit Care.

[42] Carney N, Totten AM, Oeilly C, Ullman JS, Hawryluk GW, Bell MJ (2017). Guidelines for the management of severe traumatic brain injury, fourth edition. Neurosurgery.

[43] Byrne JP, Mason SA, Gomez D, Hoeft C, Subacius H, Xiong W (2016). Timing of pharmacologic venous thromboembolism prophylaxis in severe traumatic brain injury: a propensity-matched cohort study. J Am Coll Surg.

[44] Hachem LD, Mansouri A, Scales DC, Geerts W, Pirouzmand F (2018). Anticoagulant prophylaxis against venous thromboembolism following severe traumatic brain injury: a prospective observational study and systematic review of the literature. Clin Neurol Neurosurg.

[45] Störmann P, Osinloye W, Freiman TM, Seifert V, Marzi I, Lustenberger T (2019). Early chemical thromboprophylaxis does not increase the risk of intracranial hematoma progression in patients with isolated severe traumatic brain injury. World J Surg.

[46] Kim L, Schuster J, Holena DN, Sims CA, Levine J, Pascual JL (2014). Early initiation of prophylactic heparin in severe traumatic brain injury is associated with accelerated improvement on brain imaging. J Emerg Trauma Shock.

[47] Kwiatt ME, Patel MS, Ross SE, Lachant MT, MacNew HG, Ochsner MG (2012). Is low-molecular-weight heparin safe for venous thromboembolism prophylaxis in patients with traumatic brain injury? A Western Trauma Association multicenter study. J Trauma Acute Care Surg.

[48] Farooqui A, Hiser B, Barnes SL, Litofsky NS (2013). Safety and efficacy of early thromboembolism chemoprophylaxis after intracranial hemorrhage from traumatic brain injury. J Neurosurg.

[49] Dorzi HM, Al-Yami G, Al-Daker F, Alqirnas MQ, Alhamadh MS, Khan R (2022). The association of timing of pharmacological prophylaxis and venous thromboembolism in patients with moderate-to-severe traumatic brain injury: a retrospective cohort study. Ann Thorac Med.

[50] Phelan HA, Eastman AL, Madden CJ, Aldy K, Berne JD, Norwood SH (2012). TBI risk stratification at presentation. J Trauma Acute Care Surg.

[51] Byrne JP, Witiw CD, Schuster JM, Pascual JL, Cannon JW, Martin ND (2022). Association of venous thromboembolism prophylaxis after neurosurgical intervention for traumatic brain injury with thromboembolic complications, repeated neurosurgery, and mortality. JAMA Surg.

[52] Tanweer O, Boah A, Huang PP (2013). Risks for hemorrhagic complications after placement of external ventricular drains with early chemical prophylaxis against venous thromboembolisms. J Neurosurg.

[53] Raksin PB, Harrop JS, Anderson PA, Arnold PM, Chi JH, Dailey AT (2019). Congress of Neurological Surgeons systematic review and evidence-based guidelines on the evaluation and treatment of patients with thoracolumbar spine trauma: prophylaxis and treatment of thromboembolic events. Neurosurgery.

[54] Jones T, Ugalde V, Franks P, Zhou H, White RH (2005). Venous thromboembolism after spinal cord injury: incidence, time course, and associated risk factors in 16,240 adults and children. Arch Phys Med Rehabil.

[55] Maung AA, Schuster KM, Kaplan LJ, Maerz LL, Davis KA (2011). Risk of venous thromboembolism after spinal cord injury: not all levels are the same. J Trauma.

[56] Aito S, Pieri A, D'Andrea M, Marcelli F, Cominelli E (2002). Primary prevention of deep venous thrombosis and pulmonary embolism in acute spinal cord injured patients. Spinal Cord.

[57] Khan M, Jehan F, O'Keeffe T, Hamidi M, Truitt M, Zeeshan M (2018). Optimal timing of initiation of thromboprophylaxis after nonoperative blunt spinal trauma: a propensity-matched analysis. J Am Coll Surg.

[58] Yorkgitis BK, Berndtson AE, Cross A, Kennedy R, Kochuba MP, Tignanelli C (2022). American Association for the Surgery of Trauma/American College of Surgeons-Committee on Trauma Clinical Protocol for inpatient venous thromboembolism prophylaxis after trauma. J Trauma Acute Care Surg.

[59] Zeeshan M, Khan M, O'Keeffe T, Pollack N, Hamidi M, Kulvatunyou N (2018). Optimal timing of initiation of thromboprophylaxis in spine trauma managed operatively: a nationwide propensity-matched analysis of trauma quality improvement program. J Trauma Acute Care Surg.

[60] Kim DY, Kobayashi L, Chang D, Fortlage D, Coimbra R (2015). Early pharmacological venous thromboembolism prophylaxis is safe after operative fixation of traumatic spine fractures. Spine (Phila Pa 1976).

[61] Chang R, Scerbo MH, Schmitt KM, Adams SD, Choi TJ, Wade EW (2017). Early chemoprophylaxis is associated with decreased venous thromboembolism risk without concomitant increase in intraspinal hematoma expansion after traumatic spinal cord injury. J Trauma Acute Care Surg.

[62] Ahlquist S, Park HY, Kelley B, Holly L, Shamie AN, Park DY (2020). Venous thromboembolism chemoprophylaxis within 24 hours of surgery for spinal cord injury: is it safe and effective?. Neurospine.

[63] Taghlabi K, Carlson BB, Bunch J, Jackson RS, Winfield R, Burton DC (2022). Chemoprophylactic anticoagulation 72 hours after spinal fracture surgical treatment decreases venous thromboembolic events without increasing surgical complications. N Am Spine Soc J..

[64] Tran A, Fernando SM, Carrier M, Siegal DM, Inaba K, Vogt K (2022). Efficacy and safety of low molecular weight heparin versus unfractionated heparin for prevention of venous thromboembolism in trauma patients: a systematic review and meta-analysis. Ann Surg.

[65] Maragkos GA, Cho LD, Legome E, Wedderburn R, Margetis K (2022). Delayed cranial decompression rates after initiation of unfractionated heparin versus low-molecular-weight heparin in traumatic brain injury. World Neurosurg.

[66] Cook D, Meade M, Guyatt G, PROTECT Investigators for the Canadian Critical Care Trials Group and the Australian and New Zealand Intensive Care Society Clinical Trials Group (2011). Dalteparin versus unfractionated heparin in critically ill patients. N Engl J Med.

[67] Kay AB, Majercik S, Sorensen J, Woller SC, Stevens SM, White TW (2018). Weight-based enoxaparin dosing and deep vein thrombosis in hospitalized trauma patients: a double-blind, randomized, pilot study. Surgery.

[68] Gates RS, Lollar DI, Collier BR, Smith J, Faulks ER, Gillen JR (2022). Enoxaparin titrated by anti-Xa levels reduces venous thromboembolism in trauma patients. J Trauma Acute Care Surg.

[69] Dhillon NK, Barmparas G, Lin TL, Linaval NT, Yang AR, Sekhon HK (2021). A systems-based approach to reduce deep venous thrombosis and pulmonary embolism in trauma patients. World J Surg.

[70] Kopelman TR, O'Neill PJ, Pieri PG, Salomone JP, Hall ST, Quan A (2013). Alternative dosing of prophylactic enoxaparin in the trauma patient: is more the answer?. Am J Surg.

[71] Bigos R, Solomon E, Dorfman JD, Ha M (2021). A Weight- and anti-Xa-guided enoxaparin dosing protocol for venous thromboembolism prophylaxis in intensive care unit trauma patients. J Surg Res.

[72] Karcutskie CA, Dharmaraja A, Patel J, Eidelson SA, Padiadpu AB, Martin AG (2018). Association of anti-factor Xa-guided dosing of enoxaparin with venous thromboembolism after trauma. JAMA Surg.

[73] Ko A, Harada MY, Barmparas G, Chung K, Mason R, Yim DA (2016). Association between enoxaparin dosage adjusted by anti-factor Xa trough level and clinically evident venous thromboembolism after trauma. JAMA Surg.

[74] Teichman AL, Cotton BA, Byrne J, Dhillon NK, Berndtson AE, Price MA (2023). Approaches for optimizing venous thromboembolism prevention in injured patients: findings from the consensus conference to implement optimal venous thromboembolism prophylaxis in trauma. J Trauma Acute Care Surg.

[75] Dennis M, Sandercock PA, Reid J, Graham C, Murray G, CLOTS Trials Collaboration (2009). Effectiveness of thigh-length graduated compression stockings to reduce the risk of deep vein thrombosis after stroke (CLOTS trial 1): a multicentre, randomised controlled trial. Lancet.

[76] Arabi YM, Khedr M, Dara SI, Dhar GS (2013). Use of intermittent pneumatic compression and not graduated compression stockings is associated with lower incident VTE in critically ill patients: a multiple propensity scores adjusted analysis. Chest.

[77] Anderson DR, Morgano GP, Bennett C, Dentali F, Francis CW, Garcia DA (2019). American Society of Hematology 2019 guidelines for management of venous thromboembolism: prevention of venous thromboembolism in surgical hospitalized patients. Blood Adv.

[78] Knudson MM, Lewis FR, Clinton A, Atkinson K, Megerman J (1994). Prevention of venous thromboembolism in trauma patients. J Trauma.

[79] Dennis JW, Menawat S, Von Thron J, Fallon WF, Vinsant GO, Laneve LM (1993). Efficacy of deep venous thrombosis prophylaxis in trauma patients and identification of high-risk groups. J Trauma.

[80] Fisher CG, Blachut PA, Salvian AJ, Meek RN, O'Brien PJ (1995). Effectiveness of pneumatic leg compression devices for the prevention of thromboembolic disease in orthopaedic trauma patients: a prospective, randomized study of compression alone versus no prophylaxis. J Orthop Trauma.

[81] Wan B, Fu H-Y, Yin J-T, Ren G-Q (2015). Low-molecular-weight heparin and intermittent pneumatic compression for thromboprophylaxis in critical patients. Exp Ther Med.

[82] Gersin K, Grindlinger GA, Lee V, Dennis RC, Wedel SK, Cachecho R (1994). The efficacy of sequential compression devices in multiple trauma patients with severe head injury. J Trauma.

[83] Arabi YM, Al-Hameed F, Burns KEA, Mehta S, Alsolamy SJ, Alshahrani MA (2019). Adjunctive intermittent pneumatic compression for venous thromboprophylaxis. N Engl J Med.

[84] Gould MK, Garcia DA, Wren SM, Karanicolas PJ, Arcelus JI, Heit JA (2012). Prevention of VTE in nonorthopedic surgical patients: antithrombotic therapy and prevention of thrombosis, 9th ed: American College of Chest Physicians Evidence-Based Clinical Practice Guidelines. Chest.

[85] Investigators Spinal Cord Injury Thromboprophylaxis (2003). Prevention of venous thromboembolism in the acute treatment phase after spinal cord injury: a randomized, multicenter trial comparing low-dose heparin plus intermittent pneumatic compression with enoxaparin. J Trauma.

[86] Stannard JP, Lopez-Ben RR, Volgas DA, Anderson ER, Busbee M, Karr DK (2006). Prophylaxis against deep-vein thrombosis following trauma: a prospective, randomized comparison of mechanical and pharmacologic prophylaxis. J Bone Joint Surg Am.

[87] Fuchs S, Heyse T, Rudofsky G, Gosheger G, Chylarecki C (2005). Continuous passive motion in the prevention of deep-vein thrombosis: a randomised comparison in trauma patients. J Bone Joint Surg Br.

[88] Yanar H, Kurtoglu M, Taviloglu K, Guloglu R, Ertekin C (2007). Is intermittent pneumatic compression make low molecular weight heparin more efficient in the prophylaxis of venous thromboembolism in trauma patients. Eur J Trauma Emerg Surg.

[89] Chibbaro S, Cebula H, Todeschi J, Fricia M, Vigouroux D, Abid H (2018). Evolution of prophylaxis protocols for venous thromboembolism in neurosurgery: results from a prospective comparative study on low-molecular-weight heparin, elastic stockings, and intermittent pneumatic compression devices. World Neurosurg.

[90] Kakkos S, Caprini JA, Geroulakos G, Nicolaides AN, Stansby G, Reddy DJ (2022). Combined intermittent pneumatic leg compression and pharmacological prophylaxis for prevention of venous thromboembolism. Cochrane Database Syst Rev.

[91] Guo PC, Li N, Zhong HM, Zhao GF (2022). Clinical effectiveness of a pneumatic compression device combined with low-molecular-weight heparin for the prevention of deep vein thrombosis in trauma patients: a single-center retrospective cohort study. World J Emerg Med.

[92] Shackford SR, Cipolle MD, Badiee J, Mosby DL, Knudson MM, Lewis PR (2016). Determining the magnitude of surveillance bias in the assessment of lower extremity deep venous thrombosis: a prospective observational study of two centers. J Trauma Acute Care Surg.

[93] Allen CJ, Murray CR, Meizoso JP, Ginzburg E, Schulman CI, Lineen EB (2016). Surveillance and early management of deep vein thrombosis decreases rate of pulmonary embolism in high-risk trauma patients. J Am Coll Surg.

[94] Haut ER, Noll K, Efron DT, Berenholz SM, Haider A, Cornwell EE (2007). Can increased incidence of deep vein thrombosis (DVT) be used as a marker of quality of care in the absence of standardized screening? The potential effect of surveillance bias on reported DVT rates after trauma. J Trauma Acute Care Surg.

[95] Arabi YM, Burns KE, Alsolamy SJ, Alshahrani MS, Al-Hameed FM, Arshad Z (2020). Surveillance or no surveillance ultrasonography for deep vein thrombosis and outcomes of critically ill patients: a pre-planned sub-study of the PREVENT trial. Intensive Care Med.

[96] Kay AB, Morris DS, Woller SC, Stevens SM, Bledsoe JR, Lloyd JF (2021). Trauma patients at risk for venous thromboembolism who undergo routine duplex ultrasound screening experience fewer pulmonary emboli: a prospective randomized trial. J Trauma Acute Care Surg.

[97] Ho KM, Rao S, Honeybul S, Zellweger R, Wibrow B, Lipman J (2019). A multicenter trial of vena cava filters in severely injured patients. N Engl J Med.

[98] Rajasekhar A, Lottenberg L, Lottenberg R, Feezor RJ, Armen SB, Liu H (2011). A pilot study on the randomization of inferior vena cava filter placement for venous thromboembolism prophylaxis in high-risk trauma patients. J Trauma.

[99] Alshaqaq HM, Al-Sharydah AM, Alshahrani MS, Alqahtani SM, Amer M (2023). Prophylactic inferior vena cava filters for venous thromboembolism in adults with trauma: an updated systematic review and meta-analysis. J Intensive Care Med.

[100] Khansarinia S, Dennis JW, Veldenz HC, Butcher JL, Hartland L (1995). Prophylactic Greenfield filter placement in selected high-risk trauma patients. J Vasc Surg.

[101] Rodriguez JL, Lopez JM, Proctor MC, Conley JL, Gerndt SJ, Marx MV (1996). Early placement of prophylactic vena caval filters in injured patients at high risk for pulmonary embolism. J Trauma.

[102] Gorman PH, Qadri SF, Rao-Patel A (2009). Prophylactic inferior vena cava (IVC) filter placement may increase the relative risk of deep venous thrombosis after acute spinal cord injury. J Trauma.

[103] Hemmila MR, Osborne NH, Henke PK, Kepros JP, Patel SG, Cain-Nielsen AH (2015). Prophylactic inferior vena cava filter placement does not result in a survival benefit for trauma patients. Ann Surg.

[104] Wilson JT, Rogers FB, Wald SL, Shackford SR, Ricci MA (1994). Prophylactic vena cava filter insertion in patients with traumatic spinal cord injury: preliminary results. Neurosurgery.

[105] Gosin JS, Graham AM, Ciocca RG, Hammond JS (1997). Efficacy of prophylactic vena cava filters in high-risk trauma patients. Ann Vasc Surg.

[106] Batty LM, Lyon SM, Dowrick AS, Bailey M, Mahar PD, Liew SM (2012). Pulmonary embolism and the use of vena cava filters after major trauma. ANZ J Surg.

[107] Kaufman JA, Barnes GD, Chaer RA, Cuschieri J, Eberhardt RT, Johnson MS (2020). Society of Interventional Radiology Clinical Practice Guideline for Inferior Vena Cava Filters in the Treatment of Patients with Venous Thromboembolic Disease: developed in collaboration with the American College of Cardiology, American College of Chest Physicians, American College of Surgeons Committee on Trauma, American Heart Association, Society for Vascular Surgery, and Society for Vascular Medicine. J Vasc Interv Radiol.

[108] Ganann R, Ciliska D, Thomas H (2010). Expediting systematic reviews: methods and implications of rapid reviews. Implement Sci.

[109] Stein DM, Braverman MA, Phuong J, Shipper E, Price MA, Bixby PJ, The NTRAP Neurotrauma Panel Group (2022). Developing a National Trauma Research Action Plan: results from the Neurotrauma Research Panel Delphi Survey. J Trauma Acute Care Surg.

[110] Granholm A, Alhazzani W, Derde LPG, Angus DC, Zampieri FG, Hammond NE (2021). Randomised clinical trials in critical care: past, present and future. Intensive Care Med.

[111] Knudson MM, Moore EE, Kornblith LZ, Shui AM, Brakenridge S, Bruns BR (2022). Challenging traditional paradigms in posttraumatic pulmonary thromboembolism. JAMA Surg.

[112] Costantini TW, Galante JM, Braverman MA, Phuong J, Price MA, Cuschieri J, NTRAP Acute Resuscitation Panel (2022). Developing a National Trauma Research Action Plan (NTRAP): results from the acute resuscitation, initial patient evaluation, imaging, and management research gap Delphi survey. J Trauma Acute Care Surg.

[113] Schulman S, Angerås U, Bergqvist D, Eriksson B, Lassen MR, Fisher W, Subcommittee on Control of Anticoagulation of the Scientific and Standardization Committee of the International Society on Thrombosis and Haemostasis (2010). Definition of major bleeding in clinical investigations of antihemostatic medicinal products in surgical patients. J Thromb Haemost.

[114] Rogers FB, Shackford SR, Horst MA, Miller JA, Wu D, Bradburn E (2012). Determining venous thromboembolic risk assessment for patients with trauma: The Trauma Embolic Scoring System. J Trauma Acute Care Surg.

[115] Meizoso JP, Karcutskie CA, Ray JJ, Ruiz X, Ginzburg E, Namias N (2017). A simplified stratification system for venous thromboembolism risk in severely injured trauma patients. J Surg Res.

[116] Greenfield LJ, Proctor MC, Rodriguez JL, Luchette FA, Cipolle MD, Cho J (1997). Posttrauma thromboembolism prophylaxis. J Trauma.

[117] Horlocker TT, Vandermeuelen E, Kopp SL, Gogarten W, Leffert LR, Benzon HT (2018). Regional anesthesia in the patient receiving antithrombotic or thrombolytic therapy: American Society of Regional Anesthesia and Pain Medicine Evidence-Based Guidelines (fourth edition). Reg Anesth Pain Med.

