# Supplementary material for: Saudi Critical Care Society clinical practice guidelines on the prevention of venous thromboembolism in adults with trauma: reviewed for evidence-based integrity and endorsed by the Scandinavian Society of Anaesthesiology and Intensive Care Medicine
Source: Ann Intensive Care. 2023 May 11;13:41. doi: 10.1186/s13613-023-01135-8 (PMC10172441; doi:10.1186/s13613-023-01135-8)
Supplement: Supplementary file 1 — Additional file 1: Appendix 1. AGREE Reporting Checklist [file 13613_2023_1135_MOESM1_ESM.docx]

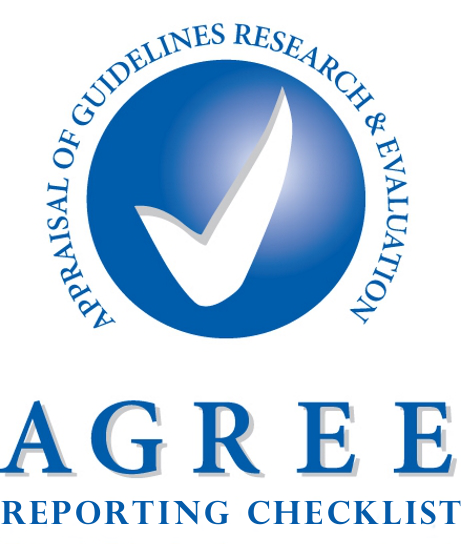
 **Additional appendix 1: AGREE Reporting Checklist**

2016

*This checklist is intended to guide the reporting of clinical practice guidelines.*

| **CHECKLIST ITEM AND DESCRIPTION** | **REPORTING CRITERIA** | **Page #** |
| --- | --- | --- |
| ***DOMAIN 1: SCOPE AND PURPOSE*** | | |
| **1. OBJECTIVES**  *Report the overall objective(s) of the guideline. The expected health benefits from the guideline are to be specific to the clinical problem or health topic.* | Health intent(s) (i.e., prevention, screening, diagnosis, treatment, etc.)  Expected benefit(s) or outcome(s)  Target(s) (e.g., patient population, society) | Stated in page 9 and page 11 |
| **2. QUESTIONS**  *Report the health question(s) covered by the guideline, particularly for the key recommendations.* | Target population  Intervention(s) or exposure(s)  Comparisons (if appropriate)  Outcome(s)  Health care setting or context | Stated in page 9 and Supplementary Appendix 2 (include structured 12 PICO questions) |
| **3. POPULATION**  *Describe the population (i.e., patients, public, etc.) to whom the guideline is meant to apply.* | Target population, sex and age  Clinical condition (if relevant)  Severity/stage of disease (if relevant)  Comorbidities (if relevant)  Excluded populations (if relevant): | Stated in Page 9  The guidelines intended for adults with trauma (mentioned in title and objective of guidelines). Moreover, it focused on patients with blunt injuries ( non-operative blunt solid organ injuries; isolated blunt TBI; isolated blunt spine trauma or fracture and/or SCI) Therefore, it excluded patients with penetrating injuries |
| ***DOMAIN 2: STAKEHOLDER INVOLVEMENT*** | | |
| **4. GROUP MEMBERSHIP**  *Report all individuals who were involved in the development process. This may include members of the steering group, the research team involved in selecting and reviewing/rating the evidence and individuals involved in formulating the final recommendations.* | Name of participant  Discipline/content expertise (e.g., neurosurgeon, methodologist)  Institution (e.g., St. Peter’s hospital)  Geographical location (e.g., Seattle, WA)  A description of the member’s role in the guideline development group | Stated in title page  Page 9-10 , page 45-46, and Supplementary Appendix 2, Table S1 |
| **5. TARGET POPULATION PREFERENCES AND VIEWS**  *Report how the views and preferences of the target population were sought/considered and what the resulting outcomes were.* | Statement of type of strategy used to capture patients’/publics’ views and preferences (e.g., participation in the guideline development group, literature review of values and preferences)  Methods by which preferences and views were sought (e.g., evidence from literature, surveys, focus groups)  Outcomes/information gathered on patient/public information  How the information gathered was used to inform the guideline development process and/or formation of the recommendations | Stated in page 11 “patient engagement section” and included under each EtD frameworks |
| **6. TARGET USERS**  *Report the target (or intended) users of the guideline.* | The intended guideline audience (e.g. specialists, family physicians, patients, clinical or institutional leaders/administrators)  How the guideline may be used by its target audience (e.g., to inform clinical decisions, to inform policy, to inform standards of care) | Page 9-10 |
| ***DOMAIN 3: RIGOUR OF DEVELOPMENT*** | | |
| **7. SEARCH METHODS**  *Report details of the strategy used to search for evidence.* | Named electronic database(s) or evidence source(s) where the search was performed (e.g., MEDLINE, EMBASE, PsychINFO, CINAHL)  Time periods searched (e.g., January 1, 2004 to March 31, 2008)  Search terms used (e.g., text words, indexing terms, subheadings)  Full search strategy included (e.g., possibly located in appendix) | Stated in page 11-12 and search strategy included in Supplementary Appendix 2 (page 8-22) |
| **8. EVIDENCE SELECTION CRITERIA**  *Report the criteria used to select (i.e., include and exclude) the evidence. Provide rationale, where appropriate.* | Target population (patient, public, etc.) characteristics  Study design  Comparisons (if relevant)  Outcomes  Language (if relevant)  Context (if relevant) | Stated in page 9 and Supplementary Appendix 2 (include structured 12 PICO questions) |
| **9****. STRENGTHS & LIMITATIONS OF THE EVIDENCE**  *Describe the strengths and limitations of the evidence. Consider from the perspective of the individual studies and the body of evidence aggregated across all the studies. Tools exist that can facilitate the reporting of this concept.* | Study design(s) included in body of evidence  Study methodology limitations (sampling, blinding, allocation concealment, analytical methods)  Appropriateness/relevance of primary and secondary outcomes considered  Consistency of results across studies  Direction of results across studies  Magnitude of benefit versus magnitude of harm  Applicability to practice context | Stated in page 12 risk of bias assessment and page 13 Certainty of evidence and Grading of Recommendations  Applicability to practice context stated under strength and limitation in discussion section page 42-43 |
| **10. FORMULATION OF RECOMMENDATIONS**  *Describe the methods used to formulate the recommendations and how final decisions were reached. Specify any areas of disagreement and the methods used to resolve them.* | Recommendation development process (e.g., steps used in modified Delphi technique, voting procedures that were considered)  Outcomes of the recommendation development process (e.g., extent to which consensus was reached using modified Delphi technique, outcome of voting procedures)  How the process influenced the recommendations (e.g., results of Delphi technique influence final recommendation, alignment with recommendations and the final vote) | Stated in page 13-14 under Recommendation formulation and voting process |
| **11. CONSIDERATION OF BENEFITS AND HARMS**  *Report the health benefits, side effects, and risks that were considered when formulating the recommendations.* | Supporting data and report of benefits  Supporting data and report of harms/side effects/risks  Reporting of the balance/trade-off between benefits and harms/side effects/risks  Recommendations reflect considerations of both benefits and harms/side effects/risks | included under each EtD frameworks |
| **12. LINK BETWEEN RECOMMENDATIONS AND EVIDENCE**  *Describe the explicit link between the recommendations and the evidence on which they are based.* | How the guideline development group linked and used the evidence to inform recommendations  Link between each recommendation and key evidence (text description and/or reference list)  Link between recommendations and evidence summaries and/or evidence tables in the results section of the guideline | included under each EtD frameworks (under justification of recommendation)  described in rational section of each PICO.  References included in each grade evidence profiles and EtD frameworks |
| **13. EXTERNAL REVIEW**  *Report the methodology used to conduct the external review.* | Purpose and intent of the external review (e.g., to improve quality, gather feedback on draft recommendations, assess applicability and feasibility, disseminate evidence)  Methods taken to undertake the external review (e.g., rating scale, open-ended questions)  Description of the external reviewers (e.g., number, type of reviewers, affiliations)  Outcomes/information gathered from the external review (e.g., summary of key findings)  How the information gathered was used to inform the guideline development process and/or formation of the recommendations (e.g., guideline panel considered results of review in forming final recommendations) | The guidelines manuscript will be submitted to peer-review publication and reviewed and endorsed by the Saudi Critical Care Society. Professional societies with related interests and expertise were invited to participate as endorsers. The guidelines are reviewed for evidence-based integrity and endorsed by the Scandinavian Society of Anaesthesiology and Intensive Care Medicine. The guidelines are under endorsement consideration by Society of Critical Care Medicine  We plan to pilot testing guideline with target end users (members of target audience, stakeholder who participated in guideline development). We plan to distribute survey and collect the feedback and summary of key findings from users to assess applicability and feasibility, and improve implementation in subsequent guidelines versions |
| **14. UPDATING PROCEDURE**  *Describe the procedure for updating the guideline.* | A statement that the guideline will be updated  Explicit time interval or explicit criteria to guide decisions about when an update will occur  Methodology for the updating procedure | Stated in page 44 under plan for guideline adaptation and updating  We will use methodology described in CheckUp checklist <https://www.agreetrust.org/resource-centre/checkup/>  to help reporting update decisions |
| ***DOMAIN 4: CLARITY OF PRESENTATION*** | | |
| **1****5. SPECIFIC AND UNAMBIGUOUS RECOMMENDATIONS**  *Describe which options are appropriate in which situations and in which population groups, as informed by the body of evidence.* | A statement of the recommended action  Intent or purpose of the recommended action (e.g., to improve quality of life, to decrease side effects)  Relevant population (e.g., patients, public)  Caveats or qualifying statements, if relevant (e.g., patients or conditions for whom the recommendations would not apply)  If there is uncertainty about the best care option(s), the uncertainty should be stated in the guideline | Stated under each PICO recommendations and remarks. Good practice statements were used for PICO 4 |
| **16. MANAGEMENT OPTIONS**  *Describe the different options for managing the condition or health issue.* | Description of management options  Population or clinical situation most appropriate to each option | Stated under each PICO recommendations, remarks, and rational section |
| **17. IDENTIFIABLE KEY RECOMMENDATIONS**  *Present the key recommendations so that they are easy to identify.* | Recommendations in a summarized box, typed in bold, underlined, or presented as flow charts or algorithms  Specific recommendations grouped together in one section | Table 1 included summary of recommendation . |
| ***DOMAIN 5: APPLICABILITY*** | | |
| **1****8. FACILITATORS AND BARRIERS TO APPLICATION**  *Describe the facilitators and barriers to the guideline’s application.* | Types of facilitators and barriers that were considered  Methods by which information regarding the facilitators and barriers to implementing recommendations were sought (e.g., feedback from key stakeholders, pilot testing of guidelines before widespread implementation)  Information/description of the types of facilitators and barriers that emerged from the inquiry (e.g., practitioners have the skills to deliver the recommended care, sufficient equipment is not available to ensure all eligible members of the population receive mammography)  How the information influenced the guideline development process and/or formation of the recommendations | included under each EtD frameworks |
| **19. IMPLEMENTATION ADVICE/TOOLS**  *Provide advice and/or tools on how the recommendations can be applied in practice.* | Additional materials to support the implementation of the guideline in practice.  For example:   - Guideline summary documents - Links to check lists, algorithms - Links to how-to manuals - Solutions linked to barrier analysis (see Item 18) - Tools to capitalize on guideline facilitators (see Item 18) - Outcome of pilot test and lessons learned | links to guidelines GRADE evidence profiles and EtD frameworks at GRADEpro online database are included in Table 1 summary of recommendation and Supplementary Appendix 2 |
| **20. RESOURCE IMPLICATIONS**  *Describe any potential resource implications of applying the recommendations.* | Types of cost information that were considered (e.g., economic evaluations, drug acquisition costs)  Methods by which the cost information was sought (e.g., a health economist was part of the guideline development panel, use of health technology assessments for specific drugs, etc.)  Information/description of the cost information that emerged from the inquiry (e.g., specific drug acquisition costs per treatment course)  How the information gathered was used to inform the guideline development process and/or formation of the recommendations | included under each EtD frameworks- resources and cost-effectiveness sections |
| **21. MONITORING/ AUDITING CRITERIA**  *Provide monitoring and/or auditing criteria to measure the application of guideline recommendations.* | Criteria to assess guideline implementation or adherence to recommendations  Criteria for assessing impact of implementing the recommendations  Advice on the frequency and interval of measurement  Operational definitions of how the criteria should be measured | We plan to pilot testing guideline with target end users (members of target audience, stakeholder who participated in guideline development). We will distribute survey and collect the feedback and summary of key findings from users to assess applicability and feasibility, and improve implementation in subsequent guidelines versions |
| ***DOMAIN 6: EDITORIAL INDEPENDENCE*** | | |
| **2****2. FUNDING BODY**  *Report the funding body’s influence on the content of the guideline.* | The name of the funding body or source of funding (or explicit statement of no funding)  A statement that the funding body did not influence the content of the guideline | Stated in page 45 |
| **23. COMPETING INTERESTS**  *Provide an explicit statement that all group members have declared whether they have any competing interests.* | Types of competing interests considered  Methods by which potential competing interests were sought  A description of the competing interests  How the competing interests influenced the guideline process and development of recommendations | Stated in page 10 under management of conflict of Interests and page 45 |

From: Brouwers MC, Kerkvliet K, Spithoff K, on behalf of the AGREE Next Steps Consortium. The AGREE Reporting Checklist: a tool to improve reporting of clinical practice guidelines. *BMJ* 2016;352:i1152. doi: 10.1136/bmj.i1152.

For more information about the AGREE Reporting Checklist, please visit the AGREE Enterprise website at <http://www.agreetrust.org>.
