# Supplementary material for: Saudi Critical Care Society clinical practice guidelines on the prevention of venous thromboembolism in adults with trauma: reviewed for evidence-based integrity and endorsed by the Scandinavian Society of Anaesthesiology and Intensive Care Medicine
Source: Ann Intensive Care. 2023 May 11;13:41. doi: 10.1186/s13613-023-01135-8 (PMC10172441; doi:10.1186/s13613-023-01135-8)
Supplement: Supplementary file 2 — Additional file 2: Appendix 2. Table S1. Selection and Organization of Committee Members. Search Strategy. Table S2. Implications of different recommendations to key stakeholders. Tables S3, S5, S7-S12. PICO questions. Table S4. Definition of bleeding risk in blunt solid organ injuries. Table S6. Evidence to Decision Framework Recommendation 4: TBI requiring neurosurgicalintervention. Evidence Profiles and Evidence to Decision Frameworks for each PICO. Meta-analyses for each PICO. Table S13. Results for studies identified after conclusion of SR and guidelines panel recommendations. Table S14. Summary findings for studies identified after conclusion of SR and guidelines panelrecommendations. [file 13613_2023_1135_MOESM2_ESM.docx]

**Additional Appendix 2**

**Saudi Critical Care Society clinical practice guidelines on the prevention of venous thromboembolism in adults with trauma**

Reviewed for evidence-based integrity and endorsed by the Scandinavian Society of Anaesthesiology and Intensive Care Medicine

**Running title:** Venous thromboembolism prophylaxis in adults with trauma guidelines

Marwa Amer^1,2 †#^, Mohammed S. Alshahrani^3 #^, Yaseen M. Arabi^4^, Ahmed Al-jedai^2,5^, Hassan M. Alshaqaq^6^, Abdulaziz Al-Sharydah^7^, Faisal A. Al-Suwaidan^8^, Hosam Aljehani^9^, Thamer Nouh^10^, Hassan Mashbari^11^, Nehal Tarazan^12^, Saad Alqahtani ^13^, Wail Tashkandi^14,15^, Khalid Maghrabi^16^, Muneerah Albugami^17^, Samaher Hashim^18^, Norah M. Alsubaie^19^, Mohammad Alsenani^20^, Haifa Algethamy^21^, Thamir M Alshammari^22^, Ali Alaklabi^23^, Nadia Ismail^24^, Esraa S. Altawil^25^, Alyaa Elhazmi^26^, Ahmed Nahhas^16^, Maha Aljuaid^27^, Naif Alsadoon^28^, Yasser Binbraik^12,29^, Yuhong Yuan^30^, Waleed Alhazzani ^12,31,32,33^

**^#^**Contributed equally

^1^ Medical/Critical Pharmacy Division, King Faisal Specialist Hospital and Research Center, Riyadh, Saudi Arabia

^2^ College of Medicine and Pharmacy, Alfaisal University, Riyadh, Saudi Arabia

^3^ Department of Emergency and Critical Care, King Fahd Hospital of the University, Imam Abdulrahman Bin Faisal University, Dammam, Saudi Arabia

^4^ Intensive Care Department, Ministry of National Guard Health Affairs, King Abdullah International Medical Research Center, King Saud Bin Abdulaziz University for Health Sciences, Riyadh, Saudi Arabia

^5^ Therapeutic Affairs, Ministry of Health, Riyadh, Saudi Arabia

^6^ Emergency Medicine Department, King Saud Medical City, Riyadh, Saudi Arabia

^7^ Diagnostic and Interventional Radiology Department, King Fahd Hospital of the University, Imam Abdulrahman Bin Faisal University, Dammam, Saudi Arabia

^8^ Clinical Excellence Administration and King Fahad Medical City, Second Health Cluster in

Riyadh, Ministry of Health, Saudi Arabia

^9^ Department of interventional Neuroradiology, Neurosurgery, Neurocritical care, King Fahd Hospital of the University, Imam Abdulrahman Bin Faisal University, Dammam, Saudi Arabia

^10^ Trauma and Acute Care Surgery Unit, King Saud University, Riyadh, Saudi Arabia

^11^ Department of Surgery, Jazan University, Jazan, Saudi Arabia

^12^ Department of Medicine, McMaster University, Hamilton, Canada

^13^ Department of Orthopedic Surgery, King Fahd Hospital of the University, Imam Abdulrahman Bin Faisal University, Dammam, Saudi Arabia

^14^ Department of Surgery, King Abdulaziz University, Jeddah, Saudi Arabia

^15^ Department of Critical Care, Fakeeh Care Group, Jeddah, Saudi Arabia

^16^ Department of Critical Care Medicine, King Faisal Specialist Hospital and Research Center, Riyadh, Saudi Arabia

^17^ Department of Internal Medicine, King Faisal Specialist Hospital and Research Center, Riyadh, Saudi Arabia

^18^ Pulmonary and Critical Care Department, International Medical Center/First Clinic, Jeddah, Saudi Arabia

^19^ Department of surgery, King Saud university medical city, Riyadh, Saudi Arabia

^20^ Trauma Center, King Saud Medical City, Riyadh, Saudi Arabia

^21^ Department of Anesthesia and Critical Care, King Abdulaziz University, Jeddah, Saudi Arabia

^22^ College of Applied Medical Sciences, King Saud University

^23^ Department of medicine, King Saud Bin Abdulaziz University for Health Sciences, King Abdullah International Medical Research Center, Riyadh, Saudi Arabia

^24^ Department of Pharmacy, Imam Abdulrahman Bin Faisal University, Dammam, Saudi Arabia

^25^ Pharmacy Department, Clinical Pharmacy Services, King Saud University Medical City, Riyadh, Saudi Arabia

^26^ Dr Sulaiman Al-Habib Medical Group, Critical Care Department, Riyadh, Saudi Arabia

^27^ King Abdulaziz Medical City, Clinical Nursing Department, Riyadh, Saudi Arabia

^28^ Alshaya International Trading Company, Riyadh, Saudi Arabia

^29^ Cardiac Sciences Department, King Saud University, Riyadh, Saudi Arabia

^30^ Division of Gastroenterology, Department of Medicine, McMaster University, Hamilton, Canada

^31^ Department of Health Research Methods, Evidence, and Impact, McMaster University, Hamilton, Canada

^32^ Department of Critical Care, College of Medicine, King Saud University, Riyadh, Saudi Arabia

^33^ Scientific Research Center, Directorate General of Armed Forces Medical Services, Riyadh, Saudi Arabia

†**Corresponding author:** Marwa Amer, Critical Care Clinical Pharmacy Consultant, King Faisal Specialist Hospital & Research Center, Al Mathar Ash Shamali, Riyadh 11564, Saudi Arabia

Adjunct Assistant Professor- Alfaisal University- College of Medicine

Tel: +966114647272 Ext 70836 or 48030

Email address: [mamer@kfshrc.edu.sa](mailto:mamer@kfshrc.edu.sa) and [mra02834@sjfc.edu](mailto:mra02834@sjfc.edu)

**Table of Contents**

| **Item** | **Page #** |
| --- | --- |
| Table S1. Selection and Organization of Committee Members | 6-7 |
| Search Strategy | 8-22 |
| Table S2. Implications of different recommendations to key stakeholders | 23 |
| Table S3. PICO question: Recommendation 1: blunt solid organ injuries managed non-operatively | 24 |
| Table S4. Definition of bleeding risk in blunt solid organ injuries | 25 |
| Evidence Profile and Evidence to Decision Framework Recommendation 1: blunt solid organ injuries managed non-operatively | 26 |
| Meta-analysis for blunt solid organ injury managed non-operatively | 27-28 |
| Table S5. PICO question: Recommendation 2-4: traumatic brain injury | 29 |
| Evidence Profile and Evidence to Decision Framework Recommendation 2: isolated blunt TBI with a ***low risk of bleeding progression*** | 30 |
| Meta-analysis for isolated blunt TBI with a ***low risk of bleeding progression*** | 31-36 |
| Evidence Profile and Evidence to Decision Framework Recommendation 3: isolated blunt TBI with a ***high risk of bleeding progression*** | 37 |
| Meta-analysis for isolated blunt TBI with a ***high risk of bleeding progression*** | 38-43 |
| Evidence Profile Recommendation 4: TBI ***requiring neurosurgical intervention*** [intracranial pressure (ICP) monitoring or external ventricular drain (EVD) or craniotomy or craniectomy] | 44 |
| Meta-analysis for TBI ***requiring neurosurgical intervention*** [ intracranial pressure (ICP) monitoring or external ventricular drain (EVD) or craniotomy or craniectomy] | 45-53 |
| Table S6. Evidence to Decision Framework Recommendation 4: TBI ***requiring neurosurgical intervention*** [ intracranial pressure (ICP) monitoring or external ventricular drain (EVD) or craniotomy or craniectomy] | 54-64 |
| Table S7. PICO question: Recommendation 5-6: spine trauma or fracture and/or SCI | 65 |
| Evidence Profile and Evidence to Decision Framework Recommendation 5: isolated spine trauma or fracture and/or SCI and ***managed non-operatively*** | 66 |
| Evidence Profile and Evidence to Decision Framework Recommendation 6: isolated spine trauma or fracture and/or SCI ***managed operatively*** | 66 |
| Meta-analysis for isolated spinal cord injury/Spine trauma and ***managed non-operatively*** | 67-70 |
| Meta-analysis for isolated spinal cord injury/Spine trauma and ***managed operatively*** | 71-74 |
| Table S8. PICO question: Recommendation 7: agent | 75 |
| Evidence Profile and Evidence to Decision Framework Recommendation 7: agent | 76 |
| Table S9. PICO question: Recommendation 8: Dose | 77 |
| Evidence Profile and Evidence to Decision Framework Recommendation 8: Dose | 78 |
| Meta-analysis for LMWH (enoxaparin) dose in trauma | 79-82 |
| Table S10. PICO question: Recommendation 9-10: Mechanical VTE prophylaxis | 83 |
| Evidence Profile and Evidence to Decision Framework Recommendation 9: Mechanical VTE prophylaxis vs. no mechanical prophylaxis | 84 |
| Evidence Profile and Evidence to Decision Framework Recommendation 10: adding mechanical VTE prophylaxis with IPC compared to pharmacologic prophylaxis alone | 84 |
| Meta-analyses for Mechanical VTE prophylaxis vs. no mechanical prophylaxis | 85-87 |
| Meta-analyses for adding mechanical VTE prophylaxis with IPC compared to pharmacologic prophylaxis | 88-90 |
| Table S11. PICO question: Recommendation 11: Ultrasound Surveillance | 91 |
| Evidence Profile and Evidence to Decision Framework Recommendation 11: Ultrasound Surveillance | 92 |
| Meta-analyses for Ultrasound Surveillance | 93-97 |
| Table S12. PICO question: Recommendation 12: Prophylactic IVC filter | 98 |
| Evidence Profile and Evidence to Decision Framework Recommendation 12: Prophylactic IVC filter | 99 |
| Meta-analyses for Prophylactic IVC filter | 100-102 |
| Table S13. Results for studies identified after conclusion of SRMAs and guidelines panel recommendations | 103 |
| Table S14. Summary findings for studies identified after conclusion of SRMAs and guidelines panel recommendations | 104-111 |

**Table S1**. **Selection and Organization of Committee Members**

The guideline chairs brought together a panel that combined committee members with expertise in specific aspects of trauma, and 1 member of the public who was able to provide the patient’s perspectives into the process. Panel members were selected to obtain a balance of expertise, gender, geographic location, and economic region, and to balance continuity and provide new perspectives with the previous committees’ membership as well as to address content needs.

The panel, included 22 academic specialists in critical care, emergency medicine, general surgery, trauma surgery, neurosurgery, orthopedics, clinical pharmacy, nursing, interventional radiology, hematology and thrombosis, and experts in research methodology. The initial list of guideline questions was developed by the guideline chairs. Panel members were invited to comment on the initial list and submit additional questions. All actionable guideline questions were structured in the Population, Intervention, Control, and Outcome(s) (PICO) format. Final PICO questions list was the responsibility of the chair and co-chair with input from PICO questions committee.

| Guideline chairs and steering committee | Dr. Marwa Amer (MA)  Prof. Waleed Alhazzani (WA)  Prof. Mohammed S Alshahrani (MSA) |
| --- | --- |
| PICO questions committee | Dr. Marwa Amer (MA)  Prof. Waleed Alhazzani (WA)  Prof. Mohammed S Alshahrani (MSA)  Prof. Yaseen M. Arabi (YA)  Dr. Hosam Aljehani (HAJ)  Dr. Thamer Nouh (TN) |
| Writing Committee | Dr. Marwa Amer (MA)  Prof. Waleed Alhazzani (WA)  Prof. Mohammed S Alshahrani (MSA)  Prof. Yaseen M. Arabi (YA)  Prof. Ahmed Al-jedai (AAJ) |
| Systematic review and meta-analysis team | Dr. Marwa Amer (MA)  Dr. Hassan M. Alshaqaq (HMA)  Dr. Ahmed Nahhas (AN)  Dr. Nehal Tarazan (NT)  Dr. Abdulaziz Alsharydah (AA)  Dr. Nadia Ismail (NI)  Dr. Hassan Mashbari (HM)  Dr. Yuhong (Cathy) Yuan (YY)  Dr. Yasser Binbraik (YB) |
| Clinical Content Experts | Dr. Marwa Amer (MA)  Prof. Waleed Alhazzani (WA)  Prof. Mohammed S Alshahrani (MSA)  Prof. Yaseen M. Arabi (YA)  Prof. Ahmed Al-jedai (AAJ)  Dr. Saad AlQahtani (SA)  Dr. Hosam aljehani (HAJ)  Dr. Thamer Nouh (NT)  Dr. Wail Tashkandi (WT)  Dr. Khalid Maghrabi (KM)  Dr. Muneerah Albugami (MAB)  Dr. Samaher Hashim (SH)  Dr. Nora Alsubaie (NA)  Dr. Hassan Mashbari (HM)  Dr. Faisal AlSuwaidan (FA)  Dr. Mohammed Alsenani (MAS)  Dr. Haifa Algethamy (HAG)  Ms. Maha Aljuaid (MJ)  Dr. Thamer Alshammary (TA)  Dr. Ali Alaklabi (AAK)  Dr. Abdulaziz Alsharydah (AA)  Dr. Nadia Ismail (NI)  Dr. Esraa altaweel (EA)  Dr. Alyaa Elhazmi (AE) |
| Patient representative | Mr. Naif Alsadoon (NAS) provided perspectives on patients’ values and preferences |

**Search Strategy**

October,19, 2021

**Databases**: Ovid Medline [ppezv], Embase [oemezd]; Cochrane Library Controlled Clinical

Trials Registry (CENTRAL)

**Filters:** RCTs and Systematic Reviews (modified CADTH), Studies/reports of humans

**Search Methods:** *“Electronic Search Strategy”*

The literature search was performed by an information specialist following PRISMA-S guidance (reference: Rethlefsen ML, et al.

PRISMA-S: an extension to the PRISMA Statement for Reporting Literature Searches in Systematic Reviews. Syst Rev. 2021 Jan

26;10(1):39. https://doi.org/10.1186/s13643-020-01542-z.) and using a peer-reviewed search strategy (below). The search

strategy was reviewed according to the methods described in McGowan, 2016 (reference: Jessie McGowan, et al. PRESS Peer

Review of Electronic Search Strategies: 2015 Guideline Statement. J Clin Epi. 2016(75):40-46.

<https://doi.org/10.1016/j.jclinepi.2016.01.021>.). Published literature was identified by searching the following bibliographic

databases on October 19, 2021: MEDLINE (1946–) with in-process records and daily updates via Ovid; Embase (1974–) via Ovid;

The Cochrane Library via Wiley. The search strategy consisted of both controlled vocabulary, such as the National Library of

Medicine’s MeSH (Medical Subject Headings), and keywords. The main search concepts were timing of traumatic brain

injury/trauma/spinal cord injury and venous thromboembolism. A methodological filter was applied to limit the retrieval to

reports of randomized controlled trials and systematic reviews. Retrieval was not limited by publication date or language.

Duplicate records were removed between Medline and Embase using Ovid default duplicate detection, with any additional

duplicates identified and removed in EndNote and Covidence.”

**Embase <1974 to 2021 October 19>, and OVID Medline Epub Ahead of Print, In-Process & Other Non-Indexed Citations, Ovid MEDLINE(R) Daily and Ovid MEDLINE(R) 1946 to Present**

**PICO 2, 3, and 4 traumatic brain injury**

**Embase <1974 to 2021 October 19>**

**OVID Medline Epub Ahead of Print, In-Process & Other Non-Indexed Citations, Ovid MEDLINE(R) Daily and Ovid MEDLINE(R) 1946 to Present**

1 exp Brain injuries, Traumatic/ 75096

2 ((head or crani* or cerebr* or capitis or brain* or forebrain* or fore-brain* or skull* or hemisphere or intra?cran* or inter?cran* or intracran* or intercran*) adj3 (injur* or trauma* or damag* or wound$ or fracture$ or contusion$ or concus*)).ti,ab,kw. 383435

3 (tbi or tbis).ti,ab,kw. 72760

4 traumatic encephalopath*.ti,ab,kw. 2274

5 exp Craniocerebral Trauma/ use ppez 167169

6 exp Head Trauma/ use oemezd 307973

7 exp Cerebrovascular Trauma/ use ppez 7623

8 or/1-7 662078

9 exp Venous Thrombosis/ use ppez 57099

10 exp Vein Thrombosis/ use oemezd 139295

11 exp Pulmonary Embolism/ use ppez 41002

12 exp Lung Embolism/ use oemezd 102069

13 ((vein* or ven*) and (thrombo* or embol*)).ti,ab,kw. 355855

14 ((blood or lung or lungs or pulmonary) adj3 (clot* or thrombo* or embol*)).ti,ab,kw. 169668

15 (VTE or DVT or PE or VTEs or DVTs or PEs).ti,ab,kw. 194862

16 (pulmonary and (embolism or embolisms or thromboembolism or thrombo-embolism or thromboembolisms or thrombo-embolisms)).ti,ab,kw. 112579

17 (thrombus* or thrombotic* or thrombolic* or thromboemboli* or thrombos* or embol*).ti,ab,kw. 896109

18 exp Embolism/ 216375

19 exp Thromboembolism/ 580485

20 exp Thrombosis/ 480059

21 Thrombophlebitis/ 33552

22 or/9-21 1313682

23 exp Time/ 2050577

24 early medical intervention/ use ppez 3347

25 early intervention/ use oemezd 28020

26 Therapy Delay/ use oemezd 15475

27 Time-to-treatment/ use ppez 8950

28 Time to treatment/ use oemezd 20727

29 (immediate or immediately or earlier or early or soon or sooner or "fast-track" or emergen* or expedit* or urgenc* or urgent* or after* or extend* or nonearlie* or non-earlie* or nonearly* or non-early* or post-pon* or postpon* or defer or deferred or delay or delayed or postpone or postponed or late or later or time or timing).ti,ab,kw. 23134870

30 or/23-29 23941404

31 8 and 22 and 30 12681

32 Thrombosis/pc 21073

33 venous thromboembolism/pc use ppez 5008

34 exp vein thrombosis/pc use oemezd 11886

35 pulmonary embolism/pc use ppez 5070

36 exp lung embolism/pc use oemezd 5961

37 post-exposure prophylaxis/ 4981

38 (chemoprevention or chemo-prevention or chemoprophylaxis or chemo-prophylaxis or thromboprophylaxis or thrombo-prophylaxis).ti,ab,kw. 57149

39 or/32-38 102063

40 8 and 39 856

41 31 or 40 13021

42 meta-analysis.pt. 143993

43 meta-analysis/ or systematic review/ or meta-analysis as topic/ or "meta analysis (topic)"/ or "systematic review (topic)"/ or exp technology assessment, biomedical/ 763541

44 ((systematic* adj3 (review* or overview*)) or (methodologic* adj3 (review* or overview*))).ti,ab,kf,kw. 545532

45 ((quantitative adj3 (review* or overview* or synthes*)) or (research adj3 (integrati* or overview*))).ti,ab,kf,kw. 27991

46 ((integrative adj3 (review* or overview*)) or (collaborative adj3 (review* or overview*)) or (pool* adj3 analy*)).ti,ab,kf,kw. 77640

47 (data synthes* or data extraction* or data abstraction*).ti,ab,kf,kw. 72374

48 (handsearch* or hand search*).ti,ab,kf,kw. 22399

49 (mantel haenszel or peto or der simonian or dersimonian or fixed effect* or latin square*).ti,ab,kf,kw. 70264

50 (met analy* or metanaly* or technology assessment* or HTA or HTAs or technology overview* or technology appraisal*).ti,ab,kf,kw. 27626

51 (meta regression* or metaregression*).ti,ab,kf,kw. 25322

52 (meta-analy* or metaanaly* or systematic review* or biomedical technology assessment* or bio-medical technology assessment*).mp,hw. 964927

53 (medline or cochrane or pubmed or medlars or embase or cinahl).ti,ab,hw. 626294

54 (cochrane or (health adj2 technology assessment) or evidence report).jw. 49101

55 (meta-analysis or systematic review).mp. 905482

56 (comparative adj3 (efficacy or effectiveness)).ti,ab,kf,kw. 37493

57 (outcomes research or relative effectiveness).ti,ab,kf,kw. 24961

58 ((indirect or indirect treatment or mixed-treatment) adj comparison*).ti,ab,kf,kw. 7127

59 or/42-58 1367445

60 (Randomized Controlled Trial or Controlled Clinical Trial or Pragmatic Clinical Trial or Equivalence Trial or Clinical Trial, Phase III).pt. 640429

61 Randomized Controlled Trial/ 1226869

62 exp Randomized Controlled Trials as Topic/ 365770

63 "Randomized Controlled Trial (topic)"/ 212918

64 Controlled Clinical Trial/ 558649

65 exp Controlled Clinical Trials as Topic/ 379650

66 "Controlled Clinical Trial (topic)"/ 11939

67 Randomization/ 198029

68 Random Allocation/ 194206

69 Double-Blind Method/ 331995

70 Double Blind Procedure/ 188756

71 Double-Blind Studies/ 315344

72 Single-Blind Method/ 73067

73 Single Blind Procedure/ 44067

74 Single-Blind Studies/ 75079

75 Placebos/ 352241

76 Placebo/ 372427

77 Control Groups/ 111513

78 Control Group/ 111513

79 (random* or sham or placebo*).ti,ab,hw,kf,kw. 3886466

80 ((singl* or doubl*) adj (blind* or dumm* or mask*)).ti,ab,hw,kf,kw. 579660

81 ((tripl* or trebl*) adj (blind* or dumm* or mask*)).ti,ab,hw,kf,kw. 2914

82 (control* adj3 (study or studies or trial* or group*)).ti,ab,kf,kw. 2600474

83 (Nonrandom* or non random* or non-random* or quasi-random* or quasirandom*).ti,ab,hw,kf,kw. 109922

84 allocated.ti,ab,hw. 168473

85 ((open label or open-label) adj5 (study or studies or trial*)).ti,ab,hw,kf,kw. 112051

86 ((equivalence or superiority or non-inferiority or noninferiority) adj3 (study or studies or trial*)).ti,ab,hw,kf,kw. 24517

87 (pragmatic study or pragmatic studies).ti,ab,hw,kf,kw. 1219

88 ((pragmatic or practical) adj3 trial*).ti,ab,hw,kf,kw. 13050

89 ((quasiexperimental or quasi-experimental) adj3 (study or studies or trial*)).ti,ab,hw,kf,kw. 24980

90 (phase adj3 (III or "3") adj3 (study or studies or trial*)).ti,hw,kf,kw. 137715

91 or/60-90 5692416

92 59 or 91 6534712

93 41 and 92 1986

94 exp animals/ 52379076

95 exp animal experimentation/ or exp animal experiment/ 2763853

96 exp models animal/ 2135776

97 nonhuman/ 6690940

98 exp vertebrate/ or exp vertebrates/ 50991954

99 94 or 95 or 96 or 97 or 98 54307434

100 exp humans/ 42629983

101 exp human experimentation/ or exp human experiment/ 569961

102 100 or 101 42632622

103 99 not 102 11676550

104 93 not 103 1486

105 104 use ppez 412

106 104 use oemezd 1074

107 remove duplicates from 104 1219

**PICO 5 and 6- Spinal Cord Injury**

**Embase <1974 to 2021 October 19>**

**OVID Medline Epub Ahead of Print, In-Process & Other Non-Indexed Citations, Ovid MEDLINE(R) Daily and Ovid MEDLINE(R) 1946 to Present**

1 exp Venous Thrombosis/ use ppez 57099

2 exp Vein Thrombosis/ use oemezd 139295

3 exp Pulmonary Embolism/ use ppez 41002

4 exp Lung Embolism/ use oemezd 102069

5 ((vein* or ven*) and (thrombo* or embol*)).ti,ab,kw. 355855

6 ((blood or lung or lungs or pulmonary) adj3 (clot* or thrombo* or embol*)).ti,ab,kw. 169668

7 (VTE or DVT or PE or VTEs or DVTs or PEs).ti,ab,kw. 194862

8 (pulmonary and (embolism or embolisms or thromboembolism or thrombo-embolism or thromboembolisms or thrombo-embolisms)).ti,ab,kw. 112579

9 (thrombus* or thrombotic* or thrombolic* or thromboemboli* or thrombos* or embol*).ti,ab,kw. 896109

10 exp Embolism/ 216375

11 exp Thromboembolism/ 580485

12 exp Thrombosis/ 480059

13 Thrombophlebitis/ 33552

14 or/1-13 1313682

15 exp Time/ 2050577

16 early medical intervention/ use ppez 3347

17 early intervention/ use oemezd 28020

18 Therapy Delay/ use oemezd 15475

19 Time-to-Treatment/ use ppez 8950

20 Time to treatment/ use oemezd 20727

21 (immediate or immediately or earlier or early or soon or sooner or "fast-track" or emergen* or expedit* or urgenc* or urgent* or after* or extend* or nonearlie* or non-earlie* or nonearly* or non-early* or post-pon* or postpon* or defer or deferred or delay or delayed or postpone or postponed or late or later or time or timing).ti,ab,kw. 23134870

22 (within adj2 (h or hours or min or minutes)).ti,ab. 389033

23 or/15-22 24032769

24 exp Spinal Cord Injuries/ use ppez 51450

25 exp Spinal Cord Injury/ use oemezd 81336

26 exp Spinal Cord/ 210660

27 exp Central Cord Syndrome/ use ppez 109

28 Spine/su 6614

29 ((spinal or spine) adj2 (contusion* or damag* or fracture* or injury or injuries or laceration* or transection* or trauma* or wound*)).ti,ab,kw. 130815

30 ((myelopathy or myelopathies) adj (traumatic or post?traumatic)).ti,ab,kw. 12

31 ("central cord injury syndrome" or "central cord syndrome" or "central spinal cord syndrome").ti,ab,kw. 685

32 ((coccygeal or lumbar or sacral or spinal or thoracic) adj cord*).ti,ab,kw. 334952

33 ((conus or spinali or spinalis) adj (medulla or medullari or medullaris or terminali or terminalis)).ti,ab,kw. 3575

34 (myelon or myelons).ti,ab,kw. 300

35 (contusion* or damag* or fracture* or injury or injuries or laceration* or transection* or trauma* or wound*).ti,ab,kw. 4579446

36 (32 or 33 or 34) and 35 140135

37 24 or 25 or 26 or 27 or 28 or 29 or 30 or 31 or 36 393858

38 Thrombosis/pc 21073

39 venous thromboembolism/pc use ppez 5008

40 exp vein thrombosis/pc use oemezd 11886

41 pulmonary embolism/pc use ppez 5070

42 exp lung embolism/pc use oemezd 5961

43 post-exposure prophylaxis/ 4981

44 (chemoprevention or chemo-prevention or chemoprophylaxis or chemo-prophylaxis or thromboprophylaxis or thrombo-prophylaxis).ti,ab,kw. 57149

45 or/38-44 102063

46 (14 or 45) and 23 and 37 5205

47 (Randomized Controlled Trial or Controlled Clinical Trial or Pragmatic Clinical Trial or Equivalence Trial or Clinical Trial, Phase III).pt. 640429

48 Randomized Controlled Trial/ 1226869

49 exp Randomized Controlled Trials as Topic/ 365770

50 "Randomized Controlled Trial (topic)"/ 212918

51 Controlled Clinical Trial/ 558649

52 exp Controlled Clinical Trials as Topic/ 379650

53 "Controlled Clinical Trial (topic)"/ 11939

54 Randomization/ 198029

55 Random Allocation/ 194206

56 Double-Blind Method/ 331995

57 Double Blind Procedure/ 188756

58 Double-Blind Studies/ 315344

59 Single-Blind Method/ 73067

60 Single Blind Procedure/ 44067

61 Single-Blind Studies/ 75079

62 Placebos/ 352241

63 Placebo/ 372427

64 Control Groups/ 111513

65 Control Group/ 111513

66 (random* or sham or placebo*).ti,ab,hw,kf,kw. 3886466

67 ((singl* or doubl*) adj (blind* or dumm* or mask*)).ti,ab,hw,kf,kw. 579660

68 ((tripl* or trebl*) adj (blind* or dumm* or mask*)).ti,ab,hw,kf,kw. 2914

69 (control* adj3 (study or studies or trial* or group*)).ti,ab,kf,kw. 2600474

70 (Nonrandom* or non random* or non-random* or quasi-random* or quasirandom*).ti,ab,hw,kf,kw. 109922

71 allocated.ti,ab,hw. 168473

72 ((open label or open-label) adj5 (study or studies or trial*)).ti,ab,hw,kf,kw. 112051

73 ((equivalence or superiority or non-inferiority or noninferiority) adj3 (study or studies or trial*)).ti,ab,hw,kf,kw. 24517

74 (pragmatic study or pragmatic studies).ti,ab,hw,kf,kw. 1219

75 ((pragmatic or practical) adj3 trial*).ti,ab,hw,kf,kw. 13050

76 ((quasiexperimental or quasi-experimental) adj3 (study or studies or trial*)).ti,ab,hw,kf,kw. 24980

77 (phase adj3 (III or "3") adj3 (study or studies or trial*)).ti,hw,kf,kw. 137715

78 or/47-77 5692416

79 meta-analysis.pt. 143993

80 meta-analysis/ or systematic review/ or meta-analysis as topic/ or "meta analysis (topic)"/ or "systematic review (topic)"/ or exp technology assessment, biomedical/ 763541

81 ((systematic* adj3 (review* or overview*)) or (methodologic* adj3 (review* or overview*))).ti,ab,kf,kw. 545532

82 ((quantitative adj3 (review* or overview* or synthes*)) or (research adj3 (integrati* or overview*))).ti,ab,kf,kw. 27991

83 ((integrative adj3 (review* or overview*)) or (collaborative adj3 (review* or overview*)) or (pool* adj3 analy*)).ti,ab,kf,kw. 77640

84 (data synthes* or data extraction* or data abstraction*).ti,ab,kf,kw. 72374

85 (handsearch* or hand search*).ti,ab,kf,kw. 22399

86 (mantel haenszel or peto or der simonian or dersimonian or fixed effect* or latin square*).ti,ab,kf,kw. 70264

87 (met analy* or metanaly* or technology assessment* or HTA or HTAs or technology overview* or technology appraisal*).ti,ab,kf,kw. 27626

88 (meta regression* or metaregression*).ti,ab,kf,kw. 25322

89 (meta-analy* or metaanaly* or systematic review* or biomedical technology assessment* or bio-medical technology assessment*).mp,hw. 964927

90 (medline or cochrane or pubmed or medlars or embase or cinahl).ti,ab,hw. 626294

91 (cochrane or (health adj2 technology assessment) or evidence report).jw. 49101

92 (meta-analysis or systematic review).mp. 905482

93 (comparative adj3 (efficacy or effectiveness)).ti,ab,kf,kw. 37493

94 (outcomes research or relative effectiveness).ti,ab,kf,kw. 24961

95 ((indirect or indirect treatment or mixed-treatment) adj comparison*).ti,ab,kf,kw. 7127

96 or/79-95 1367445

97 78 or 96 6534712

98 46 and 97 650

99 exp animals/ 52379076

100 exp animal experimentation/ or exp animal experiment/ 2763853

101 exp models animal/ 2135776

102 nonhuman/ 6690940

103 exp vertebrate/ or exp vertebrates/ 50991954

104 99 or 100 or 101 or 102 or 103 54307434

105 exp humans/ 42629983

106 exp human experimentation/ or exp human experiment/ 569961

107 105 or 106 42632622

108 104 not 107 11676550

109 98 not 108 568

110 remove duplicates from 109 463

111 109 use ppez 168

112 109 use oemezd 400

**PICO 8- Dose**

**Embase <1974 to 2021 October 19>**

**OVID Medline Epub Ahead of Print, In-Process & Other Non-Indexed Citations, Ovid MEDLINE(R) Daily and Ovid MEDLINE(R) 1946 to Present**

1 exp Brain injuries, Traumatic/ 75096

2 ((head or crani* or cerebr* or capitis or brain* or forebrain* or fore-brain* or skull* or hemisphere or intra?cran* or inter?cran* or intracran* or intercran*) adj3 (injur* or trauma* or damag* or wound$ or fracture$ or contusion$ or concus*)).ti,ab,kw. 383435

3 (tbi or tbis).ti,ab,kw. 72760

4 traumatic encephalopath*.ti,ab,kw. 2274

5 exp Craniocerebral Trauma/ use ppez 167169

6 exp Head Trauma/ use oemezd 307973

7 exp Cerebrovascular Trauma/ use ppez 7623

8 or/1-7 662078

9 exp "Wounds and Injuries"/ use ppez 950785

10 exp Injury/ use oemezd 2341382

11 ((abdominal or abdomen or thorax or thoracic) adj3 (injur* or trauma* or perforat* or penetrat*)).ti,ab,kw. 48470

12 ((splenic or spleen) adj3 rupture*).ti,ab,kw. 7869

13 ((stomach or gastric) adj3 (rupture or perforation or injur* or burst*)).ti,ab,kw. 13760

14 ((stab* or gunshot or shot or penetrat* wound* or bullet?) adj3 (abdomen* or abdominal or stomach or splenic or spleen or thorax or thoracic)).ti,ab,kw. 4747

15 (asphyxia or burn* or drown or drowning or fracture* or frostbite or injur* or lacerat* or perforation or rupture or stab* or shot or shoot* or trauma* or wound*).ti,ab,kw. 6576386

16 or/9-15 7897678

17 exp Heparin, Low-Molecular-Weight/ 83875

18 (LMWH or low?molecular?weight?heparin or ardeparin or adomiparin or ardeparin or arteven or bemiparin or certoparin or clexane or clivarin or clivarine or Danaparoid or dalteparin or Depo?Heparin or enoxaparin or Eparina or Fluxum or Fragamin or Fraxiparin or Hed?Heparin or Heparina or Heparinate or Heparine or "heparinic acid" or heparinum or Hepathrom or Lip?hepin or liquaemin or liquemin or multiparin or nadroparin or nadroparine or novoheparin or octaparin or pabyrin or parnaparin or pularin or reviparin or sandoparin or semuloparin or subeparin or sublingula or thromboliquine or tinzaparin or triofiban or vetren or vitrum).ti,ab,kw. 34151

19 17 or 18 91387

20 Drug dosage calculations/ use ppez 2631

21 Dose calculation/ use oemezd 20381

22 ((intermediate or high or higher or increased or adjusted or "goal directed" or "weight-based" or titrated or titrate or titrated) adj5 (dose or dosing or dosage)).mp. 529309

23 (dose adjustment* or "alternative dosing").mp. 22320

24 20 or 21 or 22 or 23 567568

25 (Randomized Controlled Trial or Controlled Clinical Trial or Pragmatic Clinical Trial or Equivalence Trial or Clinical Trial, Phase III).pt. 640429

26 Randomized Controlled Trial/ 1226869

27 exp Randomized Controlled Trials as Topic/ 365770

28 "Randomized Controlled Trial (topic)"/ 212918

29 Controlled Clinical Trial/ 558649

30 exp Controlled Clinical Trials as Topic/ 379650

31 "Controlled Clinical Trial (topic)"/ 11939

32 Randomization/ 198029

33 Random Allocation/ 194206

34 Double-Blind Method/ 331995

35 Double Blind Procedure/ 188756

36 Double-Blind Studies/ 315344

37 Single-Blind Method/ 73067

38 Single Blind Procedure/ 44067

39 Single-Blind Studies/ 75079

40 Placebos/ 352241

41 Placebo/ 372427

42 Control Groups/ 111513

43 Control Group/ 111513

44 (random* or sham or placebo*).ti,ab,hw,kf,kw. 3886466

45 ((singl* or doubl*) adj (blind* or dumm* or mask*)).ti,ab,hw,kf,kw. 579660

46 ((tripl* or trebl*) adj (blind* or dumm* or mask*)).ti,ab,hw,kf,kw. 2914

47 (control* adj3 (study or studies or trial* or group*)).ti,ab,kf,kw. 2600474

48 (Nonrandom* or non random* or non-random* or quasi-random* or quasirandom*).ti,ab,hw,kf,kw. 109922

49 allocated.ti,ab,hw. 168473

50 ((open label or open-label) adj5 (study or studies or trial*)).ti,ab,hw,kf,kw. 112051

51 ((equivalence or superiority or non-inferiority or noninferiority) adj3 (study or studies or trial*)).ti,ab,hw,kf,kw. 24517

52 (pragmatic study or pragmatic studies).ti,ab,hw,kf,kw. 1219

53 ((pragmatic or practical) adj3 trial*).ti,ab,hw,kf,kw. 13050

54 ((quasiexperimental or quasi-experimental) adj3 (study or studies or trial*)).ti,ab,hw,kf,kw. 24980

55 (phase adj3 (III or "3") adj3 (study or studies or trial*)).ti,hw,kf,kw. 137715

56 or/25-55 5692416

57 meta-analysis.pt. 143993

58 meta-analysis/ or systematic review/ or meta-analysis as topic/ or "meta analysis (topic)"/ or "systematic review (topic)"/ or exp technology assessment, biomedical/ 763541

59 ((systematic* adj3 (review* or overview*)) or (methodologic* adj3 (review* or overview*))).ti,ab,kf,kw. 545532

60 ((quantitative adj3 (review* or overview* or synthes*)) or (research adj3 (integrati* or overview*))).ti,ab,kf,kw. 27991

61 ((integrative adj3 (review* or overview*)) or (collaborative adj3 (review* or overview*)) or (pool* adj3 analy*)).ti,ab,kf,kw. 77640

62 (data synthes* or data extraction* or data abstraction*).ti,ab,kf,kw. 72374

63 (handsearch* or hand search*).ti,ab,kf,kw. 22399

64 (mantel haenszel or peto or der simonian or dersimonian or fixed effect* or latin square*).ti,ab,kf,kw. 70264

65 (met analy* or metanaly* or technology assessment* or HTA or HTAs or technology overview* or technology appraisal*).ti,ab,kf,kw. 27626

66 (meta regression* or metaregression*).ti,ab,kf,kw. 25322

67 (meta-analy* or metaanaly* or systematic review* or biomedical technology assessment* or bio-medical technology assessment*).mp,hw. 964927

68 (medline or cochrane or pubmed or medlars or embase or cinahl).ti,ab,hw. 626294

69 (cochrane or (health adj2 technology assessment) or evidence report).jw. 49101

70 (meta-analysis or systematic review).mp. 905482

71 (comparative adj3 (efficacy or effectiveness)).ti,ab,kf,kw. 37493

72 (outcomes research or relative effectiveness).ti,ab,kf,kw. 24961

73 ((indirect or indirect treatment or mixed-treatment) adj comparison*).ti,ab,kf,kw. 7127

74 or/57-73 1367445

75 (8 or 16) and 19 and 24 721

76 75 and (56 or 74) 228

77 exp animals/ 52379076

78 exp animal experimentation/ or exp animal experiment/ 2763853

79 exp models animal/ 2135776

80 nonhuman/ 6690940

81 exp vertebrate/ or exp vertebrates/ 50991954

82 77 or 78 or 79 or 80 or 81 54307434

83 exp humans/ 42629983

84 exp human experimentation/ or exp human experiment/ 569961

85 83 or 84 42632622

86 82 not 85 11676550

87 76 not 86 216

88 remove duplicates from 87 173

89 88 use ppez 11

90 88 use oemezd 162

**PICO 9 and 10- Mechanical prophylaxis**

**Embase <1974 to 2021 October 19>**

**OVID Medline Epub Ahead of Print, In-Process & Other Non-Indexed Citations, Ovid MEDLINE(R) Daily and Ovid MEDLINE(R) 1946 to Present**

1 exp Brain injuries, Traumatic/ 75096

2 ((head or crani* or cerebr* or capitis or brain* or forebrain* or skull* or hemisphere or intra?cran* or inter?cran* or intracran* or intercran*) adj3 (injur* or trauma$ or damag* or wound$ or fracture$ or contusion$ or concus*)).ti,ab,kw. 383435

3 (tbi or tbis).ti,ab,kw. 72760

4 traumatic encephalopath*.ti,ab,kw. 2274

5 exp Craniocerebral Trauma/ use ppez 167169

6 exp Head Trauma/ use oemezd 307973

7 exp Cerebrovascular Trauma/ use ppez 7623

8 or/1-7 662078

9 exp Venous Thrombosis/ use ppez 57099

10 exp Vein Thrombosis/ use oemezd 139295

11 exp Pulmonary Embolism/ use ppez 41002

12 exp Lung Embolism/ use oemezd 102069

13 ((vein* or ven*) and (thrombo* or embol*)).ti,ab,kw. 355855

14 ((blood or lung or lungs or pulmonary) adj3 (clot* or thrombo* or embol*)).ti,ab,kw. 169668

15 (VTE or DVT or PE or VTEs or DVTs or PEs).ti,ab,kw. 194862

16 (pulmonary and (embolism or embolisms or thromboembolism or thrombo-embolism or thromboembolisms or thrombo-embolisms)).ti,ab,kw. 112579

17 (thrombus* or thrombotic* or thrombolic* or thromboemboli* or thrombos* or embol*).ti,ab,kw. 896109

18 exp Embolism/ 216375

19 exp Thromboembolism/ 580485

20 exp Thrombosis/ 480059

21 Thrombophlebitis/ 33552

22 or/9-21 1313682

23 Thrombosis/pc 21073

24 Venous Thromboembolism/pc use ppez 5008

25 exp vein thrombosis/pc use oemezd 11886

26 Pulmonary embolism/pc use ppez 5070

27 exp lung embolism/pc use oemezd 5961

28 post-exposure prophylaxis/ 4981

29 (chemoprevention or chemo-prevention or chemoprophylaxis or chemo-prophylaxis or thromboprophylaxis or thrombo-prophylaxis).ti,ab,kw. 57149

30 or/23-29 102063

31 exp "Wounds and Injuries"/ use ppez 950785

32 exp Injury/ use oemezd 2341382

33 ((abdominal or abdomen or thorax or thoracic) adj3 (injur* or trauma* or perforat* or penetrat*)).ti,ab,kw. 48470

34 ((splenic or spleen) adj3 rupture*).ti,ab,kw. 7869

35 ((stomach or gastric) adj3 (rupture or perforation or injur* or burst*)).ti,ab,kw. 13760

36 ((stab* or gunshot or shot or penetrat* wound* or bullet?) adj3 (abdomen* or abdominal or stomach or splenic or spleen or thorax or thoracic)).ti,ab,kw. 4747

37 (asphyxia or burn* or drown or drowning or fracture* or frostbite or injur* or lacerat* or perforation or rupture or stab* or shot or shoot* or trauma* or wound*).ti,ab,kw. 6576386

38 31 or 32 or 33 or 34 or 35 or 36 or 37 7897678

39 Intermittent pneumatic compression devices/ use ppez 761

40 Intermittent pneumatic compression device/ use oemezd 1266

41 ("pneumatic compression" adj (device* or hose or stocking*)).ti,ab,kw. 890

42 ("intermittent pneumatic" adj2 compression).ti,ab,kw. 2046

43 ("pneumatic hose" or "pneumatic intermittent impulse device" or "pneumatic leg compression").ti,ab,kw. 61

44 39 or 40 or 41 or 42 or 43 3698

45 (8 or 38 or (22 or 30)) and 44 2866

46 (Randomized Controlled Trial or Controlled Clinical Trial or Pragmatic Clinical Trial or Equivalence Trial or Clinical Trial, Phase III).pt. 640429

47 Randomized Controlled Trial/ 1226869

48 exp Randomized Controlled Trials as Topic/ 365770

49 "Randomized Controlled Trial (topic)"/ 212918

50 Controlled Clinical Trial/ 558649

51 exp Controlled Clinical Trials as Topic/ 379650

52 "Controlled Clinical Trial (topic)"/ 11939

53 Randomization/ 198029

54 Random Allocation/ 194206

55 Double-Blind Method/ 331995

56 Double Blind Procedure/ 188756

57 Double-Blind Studies/ 315344

58 Single-Blind Method/ 73067

59 Single Blind Procedure/ 44067

60 Single-Blind Studies/ 75079

61 Placebos/ 352241

62 Placebo/ 372427

63 Control Groups/ 111513

64 Control Group/ 111513

65 (random* or sham or placebo*).ti,ab,hw,kf,kw. 3886466

66 ((singl* or doubl*) adj (blind* or dumm* or mask*)).ti,ab,hw,kf,kw. 579660

67 ((tripl* or trebl*) adj (blind* or dumm* or mask*)).ti,ab,hw,kf,kw. 2914

68 (control* adj3 (study or studies or trial* or group*)).ti,ab,kf,kw. 2600474

69 (Nonrandom* or non random* or non-random* or quasi-random* or quasirandom*).ti,ab,hw,kf,kw. 109922

70 allocated.ti,ab,hw. 168473

71 ((open label or open-label) adj5 (study or studies or trial*)).ti,ab,hw,kf,kw. 112051

72 ((equivalence or superiority or non-inferiority or noninferiority) adj3 (study or studies or trial*)).ti,ab,hw,kf,kw. 24517

73 (pragmatic study or pragmatic studies).ti,ab,hw,kf,kw. 1219

74 ((pragmatic or practical) adj3 trial*).ti,ab,hw,kf,kw. 13050

75 ((quasiexperimental or quasi-experimental) adj3 (study or studies or trial*)).ti,ab,hw,kf,kw. 24980

76 (phase adj3 (III or "3") adj3 (study or studies or trial*)).ti,hw,kf,kw. 137715

77 or/46-76 5692416

78 meta-analysis.pt. 143993

79 meta-analysis/ or systematic review/ or meta-analysis as topic/ or "meta analysis (topic)"/ or "systematic review (topic)"/ or exp technology assessment, biomedical/ 763541

80 ((systematic* adj3 (review* or overview*)) or (methodologic* adj3 (review* or overview*))).ti,ab,kf,kw. 545532

81 ((quantitative adj3 (review* or overview* or synthes*)) or (research adj3 (integrati* or overview*))).ti,ab,kf,kw. 27991

82 ((integrative adj3 (review* or overview*)) or (collaborative adj3 (review* or overview*)) or (pool* adj3 analy*)).ti,ab,kf,kw. 77640

83 (data synthes* or data extraction* or data abstraction*).ti,ab,kf,kw. 72374

84 (handsearch* or hand search*).ti,ab,kf,kw. 22399

85 (mantel haenszel or peto or der simonian or dersimonian or fixed effect* or latin square*).ti,ab,kf,kw. 70264

86 (met analy* or metanaly* or technology assessment* or HTA or HTAs or technology overview* or technology appraisal*).ti,ab,kf,kw. 27626

87 (meta regression* or metaregression*).ti,ab,kf,kw. 25322

88 (meta-analy* or metaanaly* or systematic review* or biomedical technology assessment* or bio-medical technology assessment*).mp,hw. 964927

89 (medline or cochrane or pubmed or medlars or embase or cinahl).ti,ab,hw. 626294

90 (cochrane or (health adj2 technology assessment) or evidence report).jw. 49101

91 (meta-analysis or systematic review).mp. 905482

92 (comparative adj3 (efficacy or effectiveness)).ti,ab,kf,kw. 37493

93 (outcomes research or relative effectiveness).ti,ab,kf,kw. 24961

94 ((indirect or indirect treatment or mixed-treatment) adj comparison*).ti,ab,kf,kw. 7127

95 or/78-94 1367445

96 45 and (77 or 95) 1093

97 exp animals/ 52379076

98 exp animal experimentation/ or exp animal experiment/ 2763853

99 exp models animal/ 2135776

100 nonhuman/ 6690940

101 exp vertebrate/ or exp vertebrates/ 50991954

102 97 or 98 or 99 or 100 or 101 54307434

103 exp humans/ 42629983

104 exp human experimentation/ or exp human experiment/ 569961

105 103 or 104 42632622

106 102 not 105 11676550

107 96 not 106 1081

108 107 use ppez 408

109 107 use oemezd 673

110 remove duplicates from 107 744

**PICO 11- US screening**

**Embase <1974 to 2021 October 19>**

**OVID Medline Epub Ahead of Print, In-Process & Other Non-Indexed Citations, Ovid MEDLINE(R) Daily and Ovid MEDLINE(R) 1946 to Present**

1 exp Brain injuries, Traumatic/ 75096

2 ((head or crani* or cerebr* or capitis or brain* or forebrain* or fore-brain* or skull* or hemisphere or intra?cran* or inter?cran* or intracran* or intercran*) adj3 (injur* or trauma* or damag* or wound$ or fracture$ or contusion$ or concus*)).ti,ab,kw. 383435

3 (tbi or tbis).ti,ab,kw. 72760

4 traumatic encephalopath*.ti,ab,kw. 2274

5 exp Craniocerebral Trauma/ use ppez 167169

6 exp Head Trauma/ use oemezd 307973

7 exp Cerebrovascular Trauma/ use ppez 7623

8 or/1-7 662078

9 exp Venous Thrombosis/ use ppez 57099

10 exp Vein Thrombosis/ use oemezd 139295

11 exp Pulmonary Embolism/ use ppez 41002

12 exp Lung Embolism/ use oemezd 102069

13 ((vein* or ven*) and (thrombo* or embol*)).ti,ab,kw. 355855

14 ((blood or lung or lungs or pulmonary) adj3 (clot* or thrombo* or embol*)).ti,ab,kw. 169668

15 (VTE or DVT or PE or VTEs or DVTs or PEs).ti,ab,kw. 194862

16 (pulmonary and (embolism or embolisms or thromboembolism or thrombo-embolism or thromboembolisms or thrombo-embolisms)).ti,ab,kw. 112579

17 (thrombus* or thrombotic* or thrombolic* or thromboemboli* or thrombos* or embol*).ti,ab,kw. 896109

18 exp Embolism/ 216375

19 exp Thromboembolism/ 580485

20 exp Thrombosis/ 480059

21 Thrombophlebitis/ 33552

22 or/9-21 1313682

23 exp "Wounds and Injuries"/ use ppez 950785

24 exp Injury/ use oemezd 2341382

25 ((abdominal or abdomen or thorax or thoracic) adj3 (injur* or trauma* or perforat* or penetrat*)).ti,ab,kw. 48470

26 ((splenic or spleen) adj3 rupture*).ti,ab,kw. 7869

27 ((stomach or gastric) adj3 (rupture or perforation or injur* or burst*)).ti,ab,kw. 13760

28 ((stab* or gunshot or shot or penetrat* wound* or bullet?) adj3 (abdomen* or abdominal or stomach or splenic or spleen or thorax or thoracic)).ti,ab,kw. 4747

29 (asphyxia or burn* or drown or drowning or fracture* or frostbite or injur* or lacerat* or perforation or rupture or stab* or shot or shoot* or trauma* or wound*).ti,ab,kw. 6576386

30 23 or 24 or 25 or 26 or 27 or 28 or 29 7897678

31 exp Mass Screening/ 404226

32 mandatory testing/ 1765

33 multiphasic screening/ 1127

34 exp early diagnosis/ 174258

35 Diagnostic tests, Routine/ 95904

36 Risk Assessment/ 925427

37 (screen or screens or screening or surveil or surveillance or tested or testing).ti,ab,kw. 5397975

38 ((early or rapid* or routin*) adj6 (detect* or diagnos* or identif* or test*)).ti,ab,kf. 1118480

39 (drive adj6 (diagnos* or detect* or test* or identif*)).ti,ab,kf. 7305

40 ((point of care or poc) adj6 (detect* or diagnos* or identif* or test*)).ti,ab,kf. 34221

41 (risk? adj1 assess*).ti,ab,kf. 216483

42 or/31-41 7245494

43 (8 or 30) and 22 and 42 33571

44 exp Ultrasonography, Doppler, Duplex/ 30261

45 (doppler adj duplex adj ultrasonography).ti,ab,kw. 136

46 blood flow velocity.ti,ab,kw. 19115

47 or/44-46 48892

48 (8 or 30) and 22 and 42 and 47 287

49 (Randomized Controlled Trial or Controlled Clinical Trial or Pragmatic Clinical Trial or Equivalence Trial or Clinical Trial, Phase III).pt. 640429

50 Randomized Controlled Trial/ 1226869

51 exp Randomized Controlled Trials as Topic/ 365770

52 "Randomized Controlled Trial (topic)"/ 212918

53 Controlled Clinical Trial/ 558649

54 exp Controlled Clinical Trials as Topic/ 379650

55 "Controlled Clinical Trial (topic)"/ 11939

56 Randomization/ 198029

57 Random Allocation/ 194206

58 Double-Blind Method/ 331995

59 Double Blind Procedure/ 188756

60 Double-Blind Studies/ 315344

61 Single-Blind Method/ 73067

62 Single Blind Procedure/ 44067

63 Single-Blind Studies/ 75079

64 Placebos/ 352241

65 Placebo/ 372427

66 Control Groups/ 111513

67 Control Group/ 111513

68 (random* or sham or placebo*).ti,ab,hw,kf,kw. 3886466

69 ((singl* or doubl*) adj (blind* or dumm* or mask*)).ti,ab,hw,kf,kw. 579660

70 ((tripl* or trebl*) adj (blind* or dumm* or mask*)).ti,ab,hw,kf,kw. 2914

71 (control* adj3 (study or studies or trial* or group*)).ti,ab,kf,kw. 2600474

72 (Nonrandom* or non random* or non-random* or quasi-random* or quasirandom*).ti,ab,hw,kf,kw. 109922

73 allocated.ti,ab,hw. 168473

74 ((open label or open-label) adj5 (study or studies or trial*)).ti,ab,hw,kf,kw. 112051

75 ((equivalence or superiority or non-inferiority or noninferiority) adj3 (study or studies or trial*)).ti,ab,hw,kf,kw. 24517

76 (pragmatic study or pragmatic studies).ti,ab,hw,kf,kw. 1219

77 ((pragmatic or practical) adj3 trial*).ti,ab,hw,kf,kw. 13050

78 ((quasiexperimental or quasi-experimental) adj3 (study or studies or trial*)).ti,ab,hw,kf,kw. 24980

79 (phase adj3 (III or "3") adj3 (study or studies or trial*)).ti,hw,kf,kw. 137715

80 or/49-79 5692416

81 meta-analysis.pt. 143993

82 meta-analysis/ or systematic review/ or meta-analysis as topic/ or "meta analysis (topic)"/ or "systematic review (topic)"/ or exp technology assessment, biomedical/ 763541

83 ((systematic* adj3 (review* or overview*)) or (methodologic* adj3 (review* or overview*))).ti,ab,kf,kw. 545532

84 ((quantitative adj3 (review* or overview* or synthes*)) or (research adj3 (integrati* or overview*))).ti,ab,kf,kw. 27991

85 ((integrative adj3 (review* or overview*)) or (collaborative adj3 (review* or overview*)) or (pool* adj3 analy*)).ti,ab,kf,kw. 77640

86 (data synthes* or data extraction* or data abstraction*).ti,ab,kf,kw. 72374

87 (handsearch* or hand search*).ti,ab,kf,kw. 22399

88 (mantel haenszel or peto or der simonian or dersimonian or fixed effect* or latin square*).ti,ab,kf,kw. 70264

89 (met analy* or metanaly* or technology assessment* or HTA or HTAs or technology overview* or technology appraisal*).ti,ab,kf,kw. 27626

90 (meta regression* or metaregression*).ti,ab,kf,kw. 25322

91 (meta-analy* or metaanaly* or systematic review* or biomedical technology assessment* or bio-medical technology assessment*).mp,hw. 964927

92 (medline or cochrane or pubmed or medlars or embase or cinahl).ti,ab,hw. 626294

93 (cochrane or (health adj2 technology assessment) or evidence report).jw. 49101

94 (meta-analysis or systematic review).mp. 905482

95 (comparative adj3 (efficacy or effectiveness)).ti,ab,kf,kw. 37493

96 (outcomes research or relative effectiveness).ti,ab,kf,kw. 24961

97 ((indirect or indirect treatment or mixed-treatment) adj comparison*).ti,ab,kf,kw. 7127

98 or/81-97 1367445

99 48 and (80 or 98) 31

100 remove duplicates from 99 30

101 99 use ppez 17

102 99 use oemezd 14

**COCHRANE Controlled Clinical Trials Registry and Cochrane Database of Systematic Reviews (*The Cochrane Library* October 19, 2021)**

#1 MeSH descriptor: [Brain Injuries, Traumatic] explode all trees

#2 ((head or crani* or cerebr* or capitis or brain* or forebrain* or fore-brain* or skull* or hemisphere or intra-cran* or inter-cran* or intracran* or intercran*) Near (injur* or trauma* or damag* or wound$ or fracture$ or contusion$ or concus*)):ti,ab,kw

#3 (tbi or tbis):ti,ab,kw

#4 traumatic encephalopath*:ti,ab,kw

#5 MeSH descriptor: [Craniocerebral Trauma] explode all trees

#6 MeSH descriptor: [Cerebrovascular Trauma] explode all trees

#7 #1 or #2 or #3 or #4 or #5 or #6

#8 MeSH descriptor: [Venous Thromboembolism] explode all trees

#9 MeSH descriptor: [Pulmonary Embolism] explode all trees

#10 ((vein* or ven*) and (thrombo* or embol*)):ti,ab,kw

#11 ((blood or lung or lungs or pulmonary) near (clot* or thrombo* or

embol*)):ti,ab,kw

#12 (VTE or DVT or PE or VTEs or DVTs or PEs):ti,ab,kw

#13 (pulmonary and (embolism or embolisms or thromboembolism or thrombo-embolism or thromboembolisms or thrombo-embolisms)):ti,ab,kw

#14 (thrombus* or thrombotic* or thrombolic* or thromboemboli* or thrombos* or

embol*):ti,ab,kw

#15 MeSH descriptor: [Embolism] explode all trees

#16 MeSH descriptor: [Thromboembolism] explode all trees

#17 MeSH descriptor: [Thrombosis] explode all trees

#18 MeSH descriptor: [Thrombophlebitis] this term only

#19 #8 or #9 or #10 or #11 or #12 or #13 or #14 or #15 or #16 or #17 or #18

#20 [mh Thrombosis/PC]

#21 [mh Venous Thromboembolism/PC]

#22 [mh pulmonary embolism/PC]

#23 [mh post-exposure prophylaxis/]

#24 (chemoprevention or chemo-prevention or chemoprophylaxis or chemoprophylaxis or thromboprophylaxis or thrombo-prophylaxis):ti,ab,kw

#25 #20 or #21 or #22 or #23 or #24

#26 #19 or #24

#27 MeSH descriptor: [Time] explode all trees

#28 MeSH descriptor: [Early Medical Intervention] explode all trees

#29 MeSH descriptor: [Time-to-Treatment] explode all trees

#30 (immediate or immediately or earlier or early or soon or sooner or "fast-track" or

emergen* or expedit* or urgenc* or urgent* or after* or extend* or nonearlie* or non-earlie* or nonearly* or non-early* or post-pon* or postpon* or defer or deferred or

delay or delayed or postpone or postponed or late or later or time or timing):ti,ab,kw

#31 #27 or #28 or #29 or #30

#32 MeSH descriptor: [Spinal Cord Injuries] explode all trees

#33 MeSH descriptor: [Spinal Cord] explode all trees

#34 MeSH descriptor: [Central Cord Syndrome] explode all trees

#35 [mh Spine/SU]

#36 ((spinal or spine) NEAR (contusion* or damag* or fracture* or injury or injuries or laceration* or transection* or trauma* or wound*)):ti,ab,kw

#37 ((myelopathy or myelopathies) NEXT (traumatic or post?traumatic)):ti,ab,kw

#38 ("central cord injury syndrome" or "central cord syndrome" or "central spinal cord syndrome"):ti,ab,kw

#39 ((coccygeal or lumbar or sacral or spinal or thoracic) NEXT cord*):ti,ab,kw

#40 ((conus or spinali or spinalis) NEXT (medulla or medullari or medullaris or terminali or terminalis)):ti,ab,kw

#41 (myelon or myelons):ti,ab,kw

#42 (contusion* or damag* or fracture* or injury or injuries or laceration* or transection* or trauma* or wound*):ti,ab,kw

#43 (#39 or #40 or #41) and #42

#44 #32 or #33 or #34 or #35 or #36 or #37 or #38 or #43

#45 MeSH descriptor: [Wounds and Injuries] explode all trees

#46 ((abdominal or abdomen or thorax or thoracic) NEAR (injur* or trauma* or perforat* or penetrat*)):ti,ab,kw

#47 ((splenic or spleen) NEAR rupture*):ti,ab,kw

#48 ((stomach or gastric) NEAR (rupture or perforation or injur* or burst*)):ti,ab,kw

#49 ((stab* or gunshot or shot or penetrat* wound* or bullet?) NEAR (abdomen* or abdominal or stomach or splenic or spleen or thorax or thoracic)):ti,ab,kw

#50 (asphyxia or burn* or drown or drowning or fracture* or frostbite or injur* or lacerat* or perforation or rupture or stab* or shot or shoot* or trauma* or wound*):ti,ab,kw

#51 #45 or #46 or #47 or #48 or #49 or #50

#52 MeSH descriptor: [Heparin, Low-Molecular-Weight] explode all trees

#53 (LMWH or low?molecular?weight?heparin or ardeparin or adomiparin or

ardeparin or arteven or bemiparin or certoparin or clexane or clivarin or clivarine or

Danaparoid or dalteparin or Depo?Heparin or enoxaparin or Eparina or Fluxum or

Fragamin or Fraxiparin or Hed?Heparin or Heparina or Heparinate or Heparine or

"heparinic acid" or heparinum or Hepathrom or Lip?hepin or liquaemin or liquemin or

multiparin or nadroparin or nadroparine or novoheparin or octaparin or pabyrin or

parnaparin or pularin or reviparin or sandoparin or semuloparin or subeparin or

sublingula or thromboliquine or tinzaparin or triofiban or vetren or vitrum):ti,ab,kw

#54 #52 or #53

#55 MeSH descriptor: [Drug Dosage Calculations] explode all trees

#56 ((intermediate or high or higher or increased or adjusted or "goal directed" or "weight-based" or titrated or titrate or titrated) near (dose or dosing or dosage))

#57 (dose adjustment* or "alternative dosing")

#58 #55 or #56 or #57

#59 MeSH descriptor: [Intermittent Pneumatic Compression Devices] explode all trees

#60 ("pneumatic compression" next (device* or hose or stocking*)):ti,ab,kw

#61 ("intermittent pneumatic" near compression):ti,ab,kw

#62 ("pneumatic hose" or "pneumatic intermittent impulse device" or "pneumatic leg compression"):ti,ab,kw

#63 #59 or #60 or #61 or #62

#64 MeSH descriptor: [Mass Screening] explode all trees

#65 MeSH descriptor: [Mandatory Testing] explode all trees

#66 MeSH descriptor: [Multiphasic Screening] explode all trees

#67 MeSH descriptor: [Early Diagnosis] explode all trees

#68 MeSH descriptor: [Diagnostic Tests, Routine] explode all trees

#69 MeSH descriptor: [Risk Assessment] explode all trees

#70 (screen or screens or screening or surveil or surveillance or tested or testing):ti,ab,kw

#71 ((early or rapid* or routin*) near (detect* or diagnos* or identif* or test*)):ti,ab,kw

#72 (drive NEAR (diagnos* or detect* or test* or identif*)):ti,ab,kw

#73 ((point of care or poc) NEAR (detect* or diagnos* or identif* or test*)):ti,ab,kw

#74 (risk? NEAR assess*):ti,ab,kw

#75 #64 or #65 or #66 or #67 or #68 or #69 or #70 or #71 or #72 or #73 or #74

#76 MeSH descriptor: [Ultrasonography, Doppler, Duplex] explode all trees

#77 (doppler NEXT duplex NEXT ultrasonography):ti,ab,kw

#78 blood flow velocity:ti,ab,kw

#79 #76 or #77 or #78

#80 #7 and #26 and #31

#81 #26 and #31 and #44

#82 (#7 or #51) and #54 and #58

#83 (#7 OR #51) AND #26 AND #63

#84 (#7 OR #51) AND #19 AND #75 AND #79

#85 #80 OR #81 OR #82 OR #83 OR #84

50 Systematic reviews

843 clinical trials

PICOs 2 and 3: 25 systematic reviews, 497 trials (522 citations)

PICOs 5 and 6: 7 systematic reviews, 214 trials (221 citations)

PICO 8: 17 systematic reviews, 69 trials (86 citations)

PICOs 9 and 10: 4 systematic reviews, 68 trials (72 citations)

PICO 11: 35 trials (35 citations)

**Table S2:** **Implications of different recommendations to key stakeholders**

| **Recommendation** | **Meaning** | **Implication to patients** | **Implications to clinicians** | **Implications to policymakers** |
| --- | --- | --- | --- | --- |
| Strong recommendation | Must do or must avoid | Almost all individuals in this situation would want the recommended intervention, and only a small proportion would not want it | Most individuals should receive the recommended course of action | Can be adapted as policy in most situations, including the use as performance indicators |
| Conditional recommendation | Consider doing or Consider avoiding | The majority of individuals in this situation would want the recommended intervention, but many would not | Different choices are likely to be appropriate for different patients, and the recommendation should be tailored to the individual patient’s circumstances. Such as patients’, families, values and preferences | Policies will likely be variable |

**Table S3. PICO question: Recommendation 1**

In adults with blunt solid organ injury, should we recommend early pharmacologic VTE prophylaxis (24-48 hours) versus delayed pharmacologic VTE prophylaxis (> 48 hours)?

| Population | Intervention | Comparator | Outcomes |
| --- | --- | --- | --- |
| Adults with blunt solid organ injury to liver, spleen, or kidney managed non-operatively with low risk of bleeding | Early pharmacologic VTE prophylaxis ( 24-48 hours) | delayed pharmacologic VTE prophylaxis (> 48 hours) | 1. VTE 2. Major bleeding 3. Need for surgical intervention |

The American Association for the Surgery of Trauma (AAST) developed the Organ Injury Scaling system for solid organs based on the magnitude of the anatomic disruption. injuries are graded from minimal (grade 1) to lethal (grade 6) based on CT abdomen findings. <https://www.aast.org/resources-detail/injury-scoring-scale>

| **Table S4. Definition of bleeding risk in blunt solid organ injury** | | | |
| --- | --- | --- | --- |
|  | **Low risk for bleeding** | **Patients at risk of failure NOM ^b^** | **Possible high risk of bleeding with indication for urgent laparotomy (OM)** |
| Liver^[[1]](#footnote-1)^ | hemodynamically stable patients ^a^ without peritonitis    Can by managed non-operatively irrespective of the injury grade or patient age as long as hemodynamic stable | Hepatic injuries who require ongoing fluid resuscitation to maintain hemodynamic stability, have multiple solid-organ injuries, a higher Grade IV or V injury, large hemoperitoneum, or contrast extravasation on CT scan | Patients who are hemodynamically unstable with evidence of intra-abdominal hemorrhage (a positive FAST or DPL) ^c^  Patients who have peritonitis  Any suspicion of hollow viscous injury  Patients with clinical symptoms of systemic inflammatory response, sepsis, an elevation in serum bilirubin levels, or worsening abdominal pain |
| Spleen ^[[2]](#footnote-2)^ | hemodynamically stable patients without peritonitis | Patients with a vascular blush or pseudoaneurysm on CT scan, Grade III injuries with large hemoperitoneum or a Grade IV or V injury | patients who have diffuse peritonitis or who are hemodynamically unstable after blunt abdominal trauma with evidence of intraperitoneal hemorrhage (a positive FAST examination result or positive DPL) should undergo immediate exploratory laparotomy  Suspicion of hollow viscous injury or change in abdominal pain pattern indicates a need for operation |
| Kidney^[[3]](#footnote-3)^ | NOM should be the treatment of choice for all hemodynamical stable or stabilized minor (AAST I-II), moderate (AAST III) and severe (AAST IV-V) lesions | In one prospective study, failure of non-operative management due to delayed bleeding was more likely to occur in kidney injury grade ≥ IV | Hemodynamically unstable  severe injury with main renal vein injury without self-limiting bleeding  Presence of non-viable tissue (devascularized kidney) is not an indication to OM in the acute setting in the absence of other indications for laparotomy |

Abbreviations: DPL= Diagnostic peritoneal lavage, NOM= non-operative management, OM= operative management

^a^ hemodynamically stable if patient is maintaining SBP > 90 mmHg and does not have a base deficit, and is making > 50 ml of urine/hour.

^b^ should be approached with caution for VTEPX initiation time. 75% will fail NOM within 48 hours of injury. Of note, early VTEPX was not associated with increase the risk of NOM failure rate. Angioembolization maybe used as salvage or adjunct to limit persistent or delayed bleeding or other delayed complications.

^C^ hemodynamic instability defined as admission SBP < 90 mmHg with evidence of skin vasoconstriction (cool, clammy, decreased capillary refill), altered level of consciousness and/or shortness of breath, or > 90 mmHg but requiring bolus infusions/ transfusions and/or vasopressor drugs and/or admission base excess (BE) > − 5 mmol/l and/or shock index > 1 and/or transfusion requirement of at least 4–6 Units of packed red blood cells within the first 24 h

**Evidence Profile and Evidence to Decision Framework Recommendation 1: blunt solid organ injuries managed non-operatively**

<https://guidelines.gradepro.org/profile/FmzyU2rljqs>

**Meta-analysis for blunt solid organ injuries**

**VTE - low risk of bias studies**


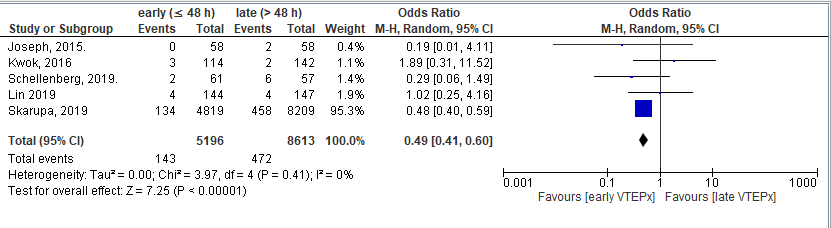


**Odds of requiring blood transfusion in SOI- low risk of bias studies**


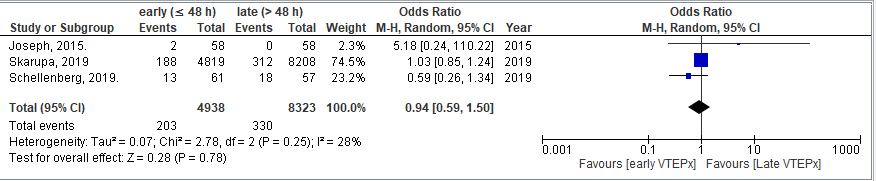


**Rate of Bleeding (from largest study by Skarupa et al) (assessed with: Rate of Postprophylaxis pRBC transfusions received)**


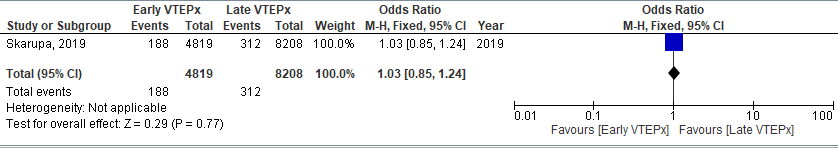


**Failure of Non-operative management - low risk of bias studies**


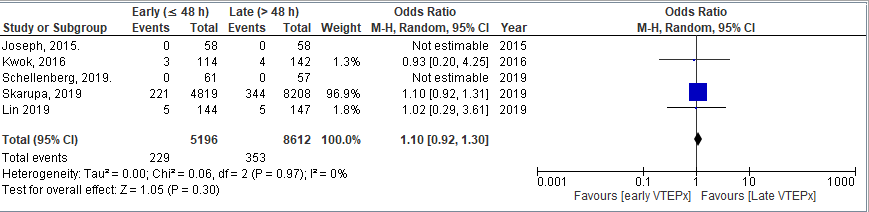


**Mortality (from largest study by Skarupa et al)**


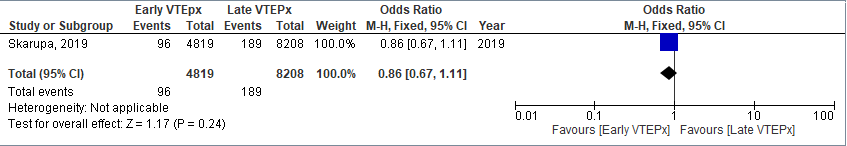


**Hospital LOS (from largest study by Skarupa et al)**


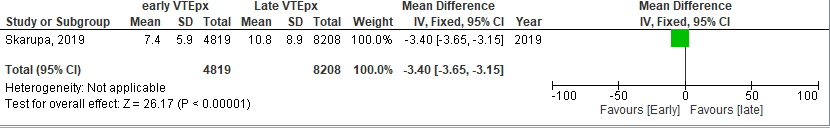


**ICU LOS** **(from largest study by Skarupa et al)**


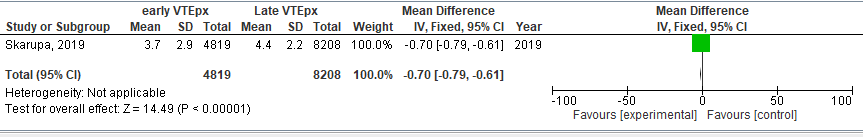


**Table S5. PICO question: Recommendation 2-4: traumatic brain injury**

**Recommendation 2: TBI with low risk of bleeding progression**

In adults with isolated blunt traumatic brain injury (TBI) with a low risk of bleeding progression, should we recommend early pharmacologic VTE prophylaxis (within 24-72 hours’ post-injury with stable brain imaging showing no bleeding progression prior to pharmacological VTE prophylaxis commencement) versus delayed pharmacologic VTE prophylaxis (>72 hours)?

| Population | Intervention | Comparator | Outcomes |
| --- | --- | --- | --- |
| Isolated blunt TBI patients with low risk of bleeding progression | Early pharmacologic VTE prophylaxis (within 24-72 hours post-injury with stable brain imaging showing no bleeding progression prior to pharmacological VTE prophylaxis commencement) | delayed pharmacologic VTE prophylaxis (> 72 hours) | 1. VTE 2. Intracranial bleeding or hematoma expansion |

**Recommendation 3: TBI with high risk of bleeding progression**

In adults with blunt TBI who are at high-risk of bleeding progression, should we recommend early pharmacologic VTE prophylaxis (within 72 hours’ post-injury with stable CT head that showed no bleeding progression) versus delayed pharmacologic VTE prophylaxis (> 72 hours)?

| Population | Intervention | Comparator | Outcomes |
| --- | --- | --- | --- |
| Isolated blunt TBI patients with a high risk of bleeding progression | Early pharmacologic VTE prophylaxis (72 hours post-injury with stable CT head that showed no bleeding progression) | delayed pharmacologic VTE prophylaxis (> 72 hours) | 1. VTE 2. Intracranial bleeding or hematoma expansion |

**Recommendation 4: TBI requiring invasive intervention**

In adults with TBI requiring intracranial pressure (ICP) monitoring or external ventricular drain (EVD) or craniotomy or craniectomy, should we recommend early pharmacologic VTE prophylaxis 24 hours from the procedure and follow-up stable CT head versus delayed pharmacologic VTE prophylaxis (>24 hours)?

| Population | Intervention | Comparator | Outcomes |
| --- | --- | --- | --- |
| Adults with severe TBI requiring invasive intervention (e.g., ICP monitor or EVD) or post craniotomy or craniectomy | Early pharmacologic VTE prophylaxis (24 hours from the procedure and follow-up stable CT head) | delayed pharmacologic VTE prophylaxis (> 24 hours) | 1. VTE 2. Intracranial bleeding or hematoma expansion |

**Evidence Profile and Evidence to Decision Framework Recommendation 2: isolated blunt TBI with a low risk of bleeding progression**

<https://guidelines.gradepro.org/profile/aXj7XJvkfm8>

**Meta-analysis:** **isolated blunt TBI with a low risk of bleeding progression**

1. **Mortality**


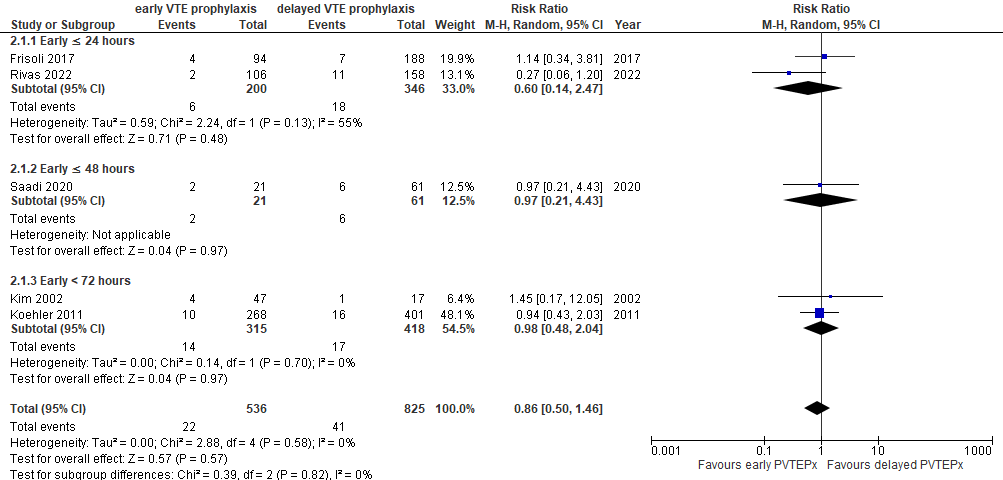


**Sensitivity analysis for mortality:**

Removed mixed population studies: Kim 2002 and Koehler 2011, therefore, no study in subgroup early <72 hours


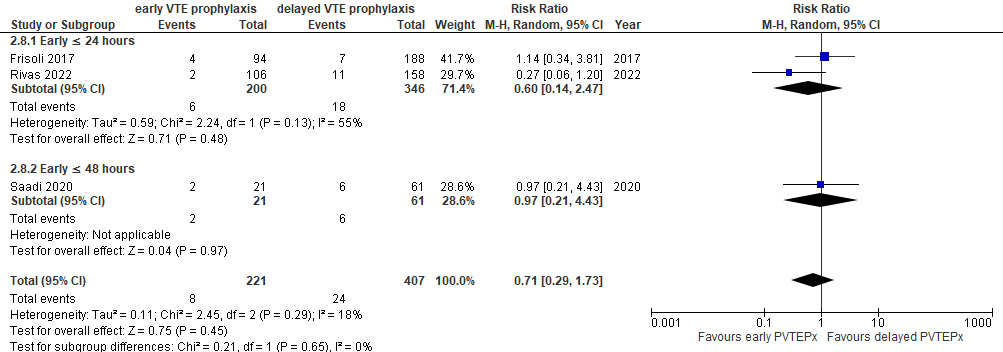


1. **VTE:**


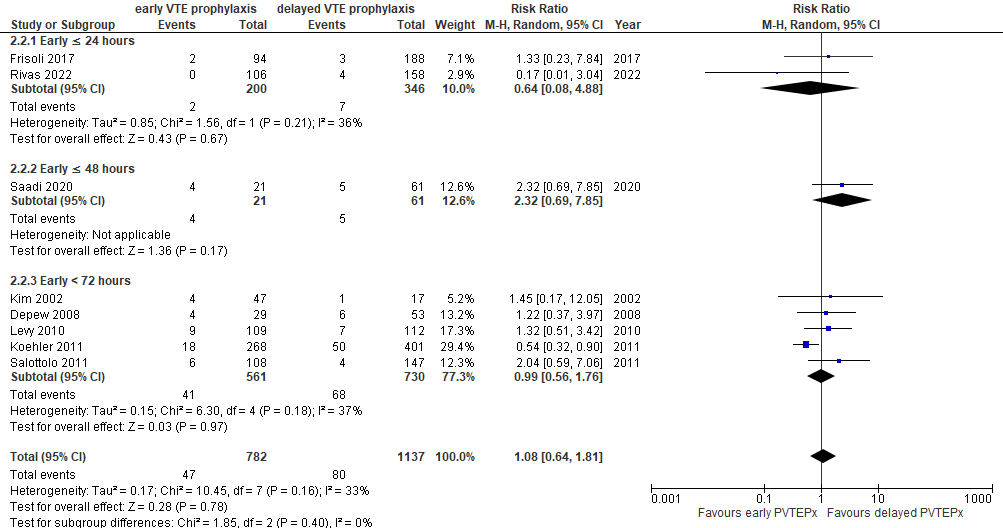


**Sensitivity analysis for VTE:**

Removed studies had a mixed patient population (blunt and penetrating TBI) , moderate and severe TBI (Saadi 2020), poly-trauma (Depew 2008, Koehler 2011, Kim 2002, Levy 2010) and interrupted VTEPx (Salottolo 2011) . Therefore, only studies left in subgroup early ≤24 hours
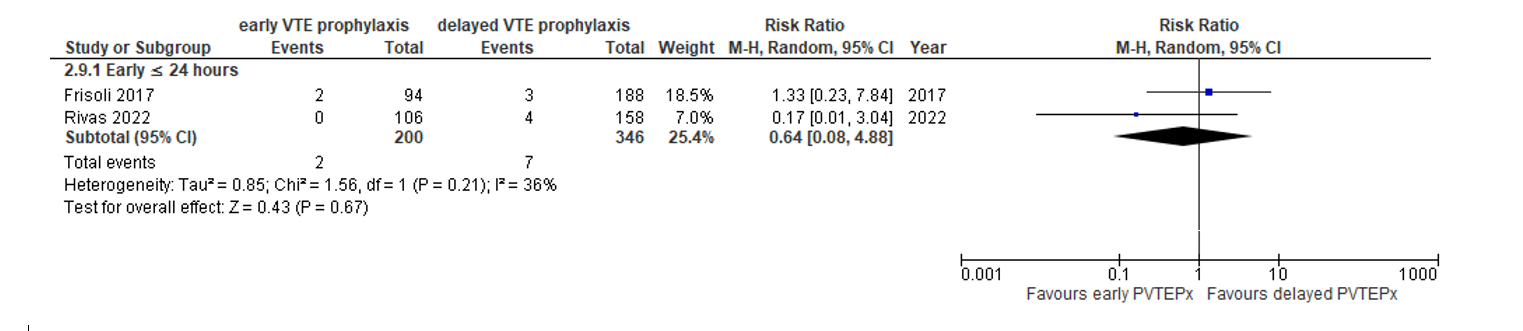


**DVT**


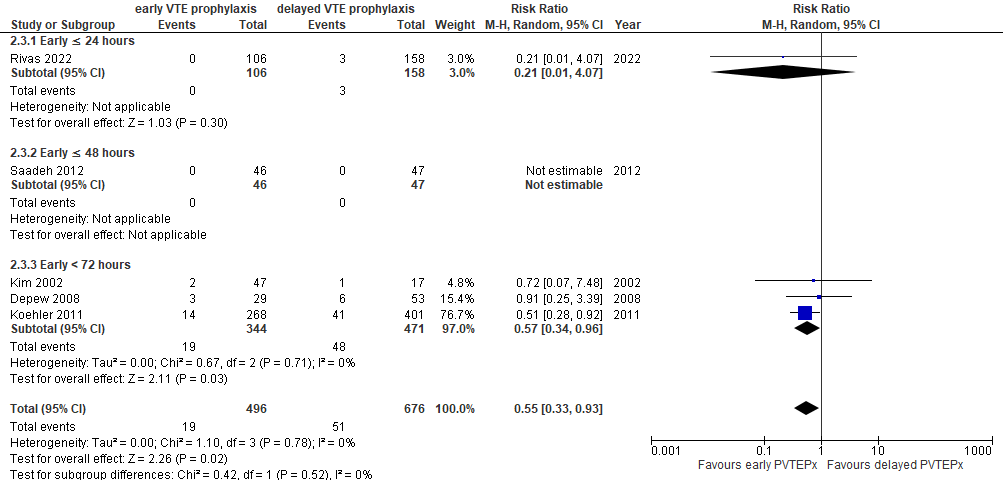


**Sensitivity analysis for DVT:**

Removed studies had a mixed patient population (blunt and penetrating TBI) , poly-trauma (Depew 2008, Koehler 2011, Kim 2002). Therefore, no study left in subgroup early <72 hours


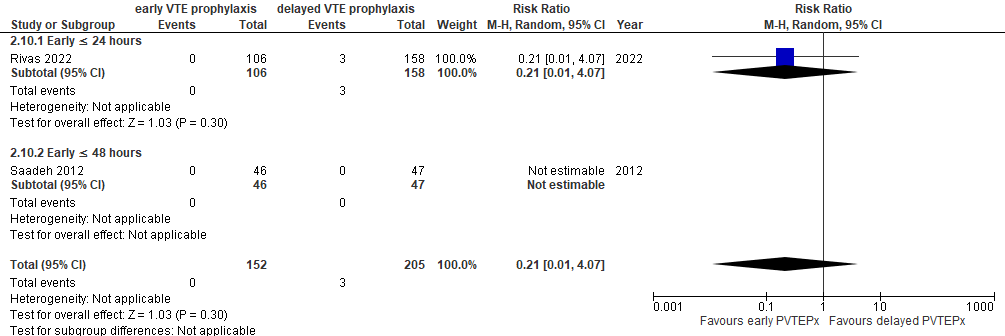


1. **PE:**


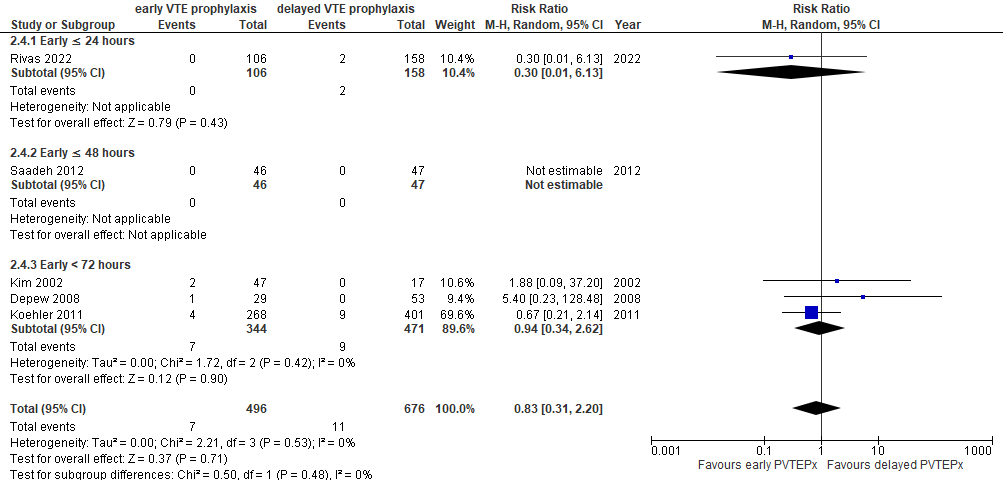


**Sensitivity analysis for PE:**

Removed studies had a mixed patient population (blunt and penetrating TBI) , poly-trauma (Depew 2008, Koehler 2011, Kim 2002). Therefore, no study left in subgroup early <72 hours


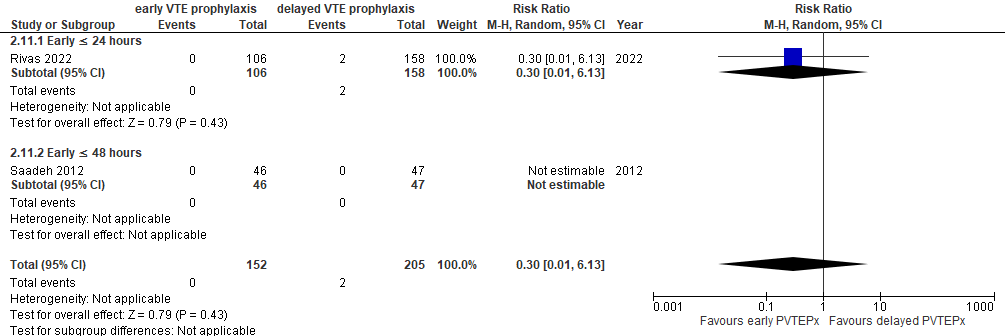


1. **Rates of ICH Progression after starting PVTEPx**


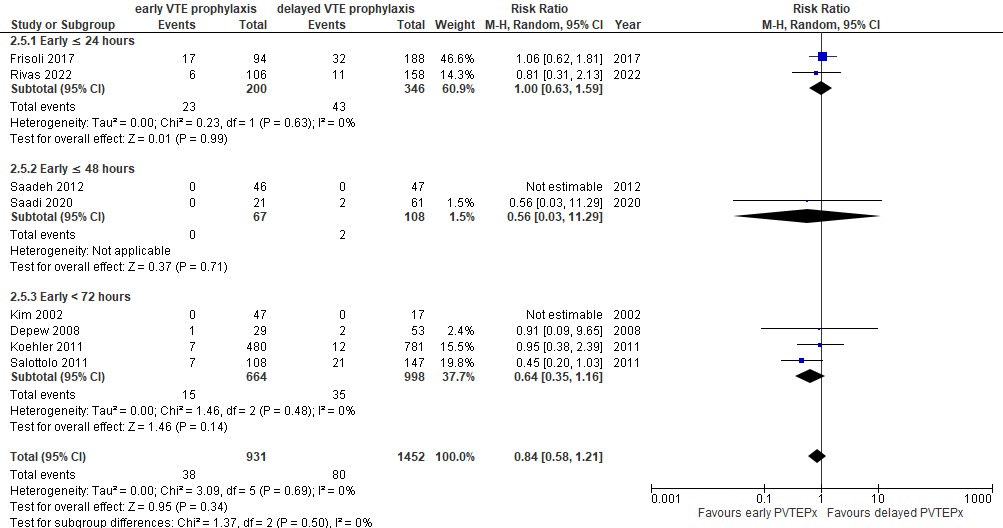


**Sensitivity analysis for ICH Progression after starting PVTEPx:**

Removed studies had a mixed patient population (blunt and penetrating TBI) , poly-trauma (Depew 2008, Koehler 2011, Kim 2002). Therefore, only one study left in subgroup early <72 hours


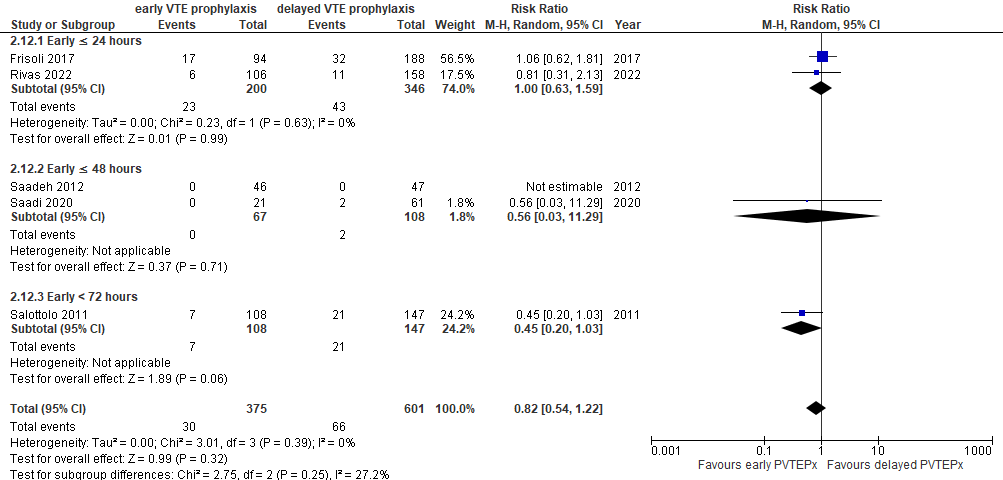


1. **Rate of acute surgical intervention [craniotomies/ craniectomies, EVD , ICP monitor ] after starting PVTEPx  to control hemorrhagic progression**


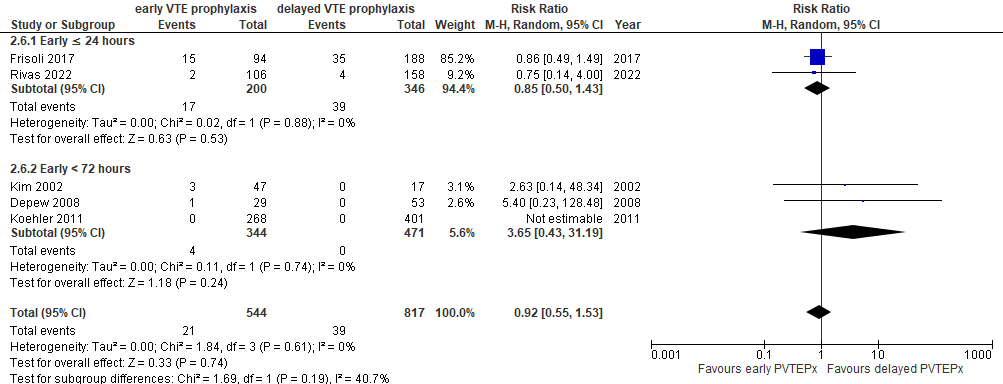


**Sensitivity analysis for acute surgical intervention:**

Removed studies had a mixed patient population (blunt and penetrating TBI) , poly-trauma (Depew 2008, Koehler 2011, Kim 2002). Therefore, no study left in subgroup early <72 hours


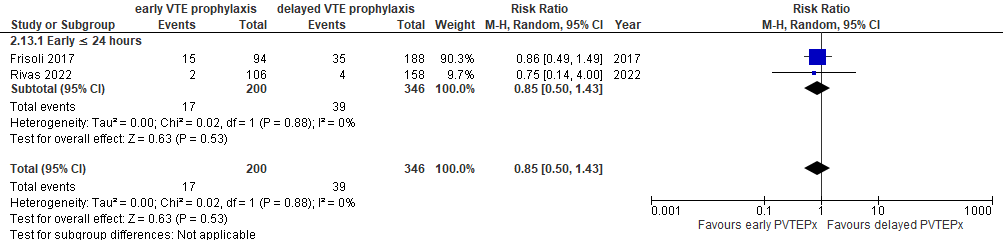


1. **Extra-cranial bleeding**

**
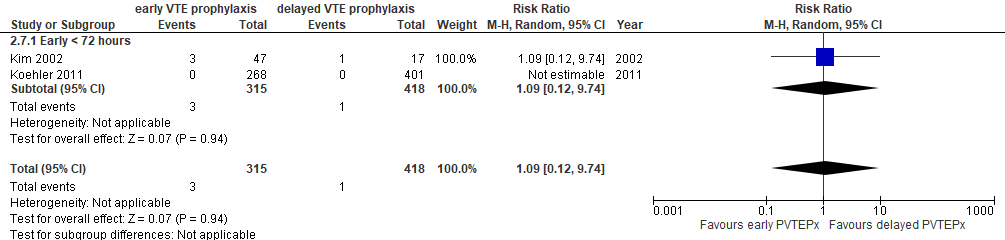
**

No sensitivity is possible, as all studies were mixed population studies

**Evidence Profile and Evidence to Decision Framework Recommendation 3: isolated blunt TBI with a high risk of bleeding progression**

<https://guidelines.gradepro.org/profile/7y-WPSqYQvw>

**Meta-analysis:** **isolated blunt TBI with a high risk of bleeding progression**

1. **Mortality**


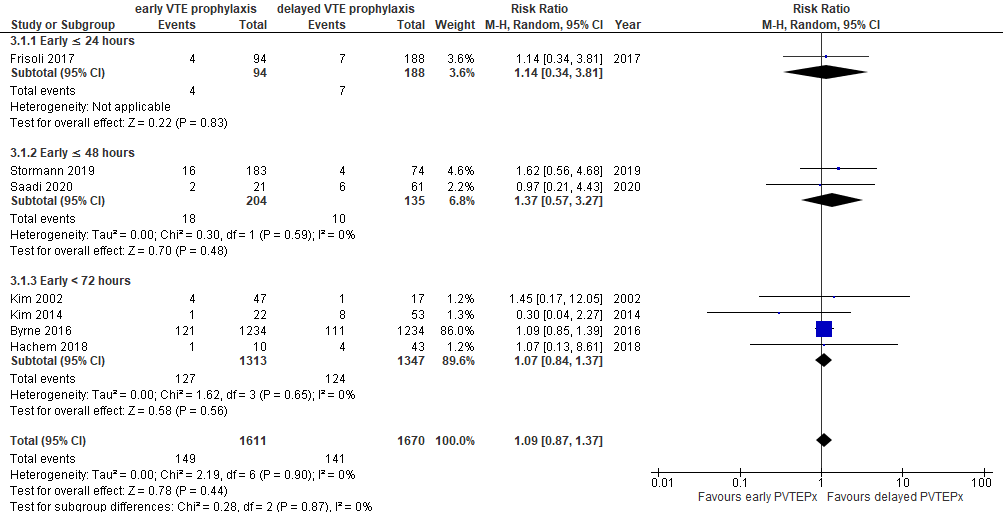


**Sensitivity analysis for mortality**

Removed studies had a mixed patient population, poly-trauma [Hachem 2018, Kim 2002], therefore, only two studies left in subgroup early <72 hours


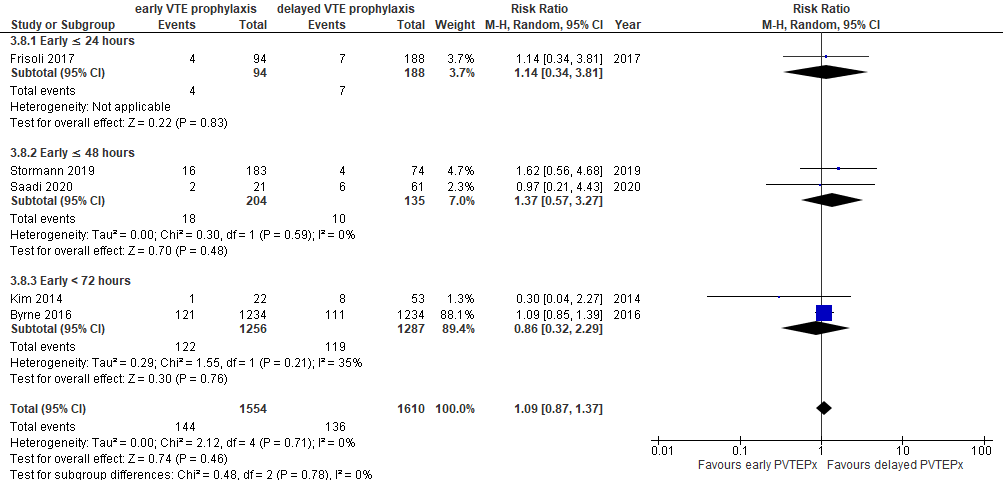


1. **DVT**


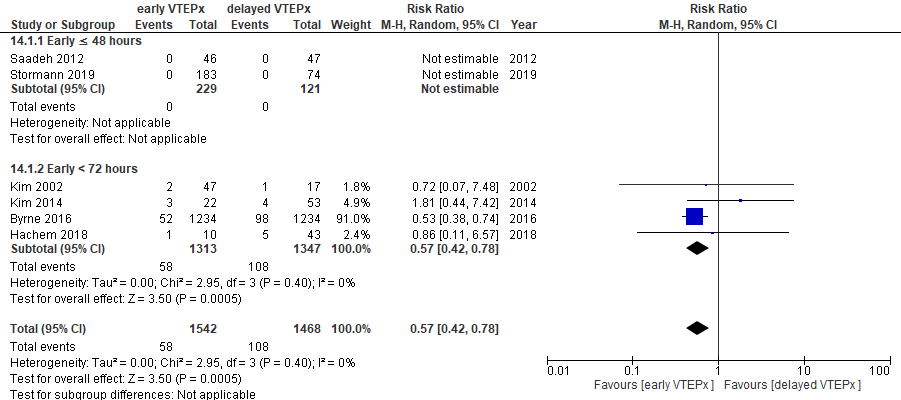


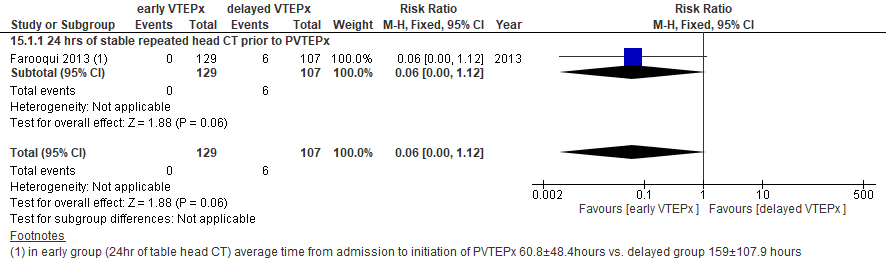


**Sensitivity analysis for DVT:**

Removed studies had a mixed patient population , poly-trauma [Hachem 2018, Kim 2002], therefore, only two studies left in subgroup early <72 hours.


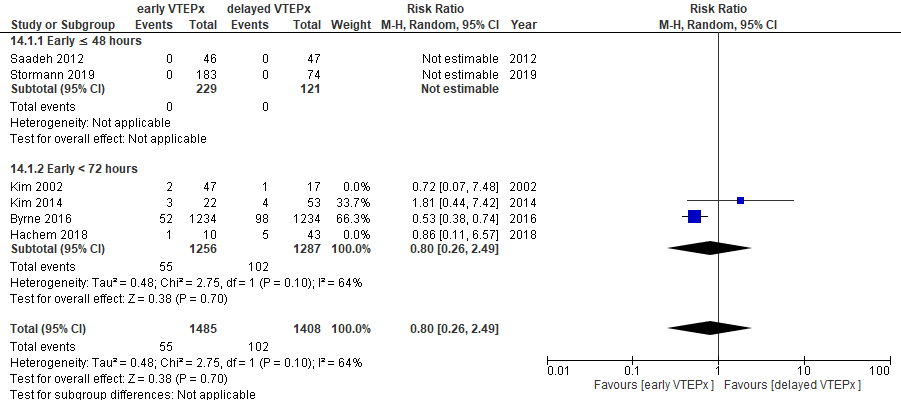


1. **PE:**

**
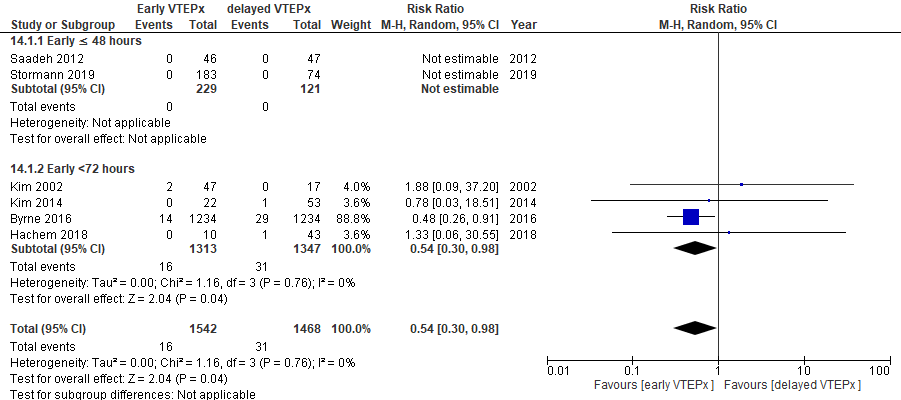
**

**
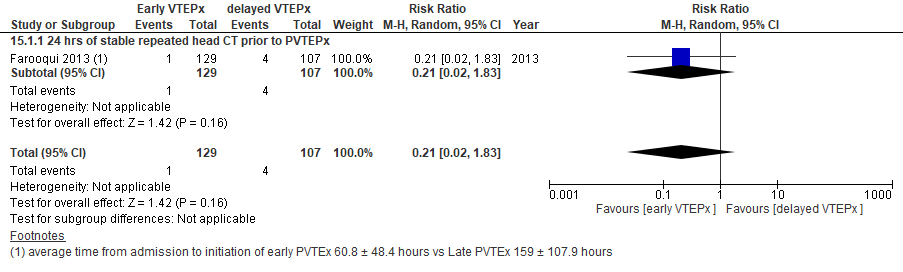
**

**Sensitivity analysis for PE:**

Removed studies had a mixed patient population, poly-trauma [Hachem 2018, Kim 2002], therefore, only two studies left in subgroup early <72 hours.


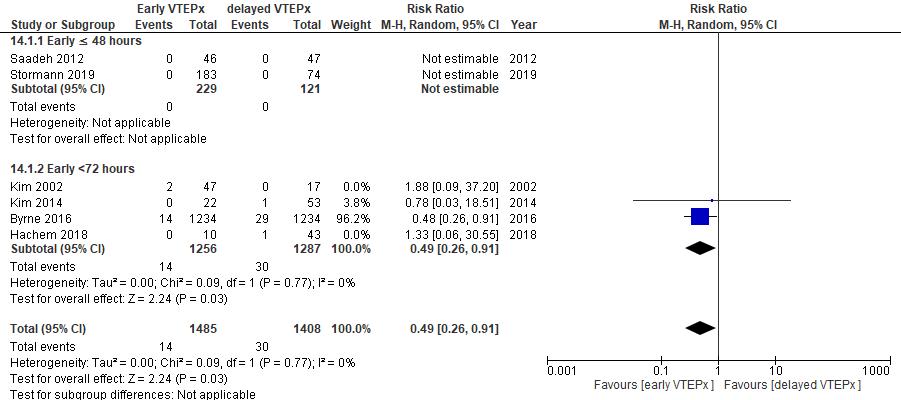


1. **Rates of ICH Progression after starting PVTEPx**


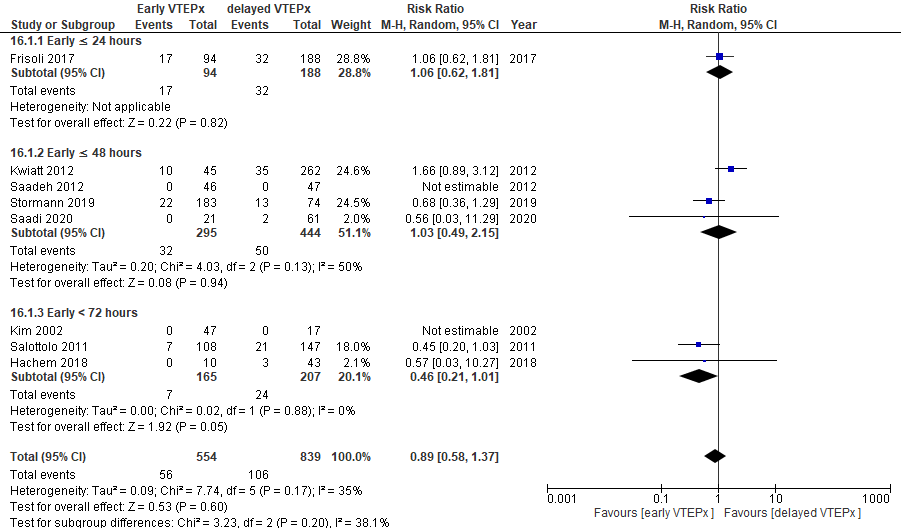


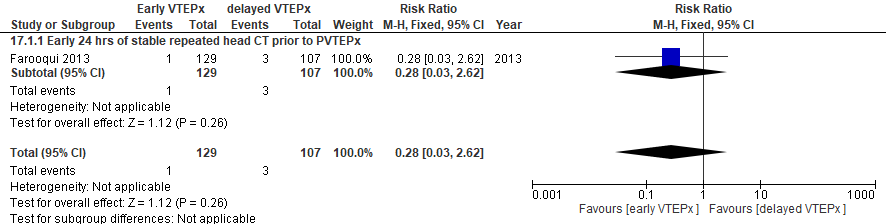


**Sensitivity analysis for ICH Progression after starting PVTEPx:**

Removed studies had a mixed patient population, poly-trauma [Hachem 2018, Kim 2002], therefore, only one study left in subgroup early <72 hours.


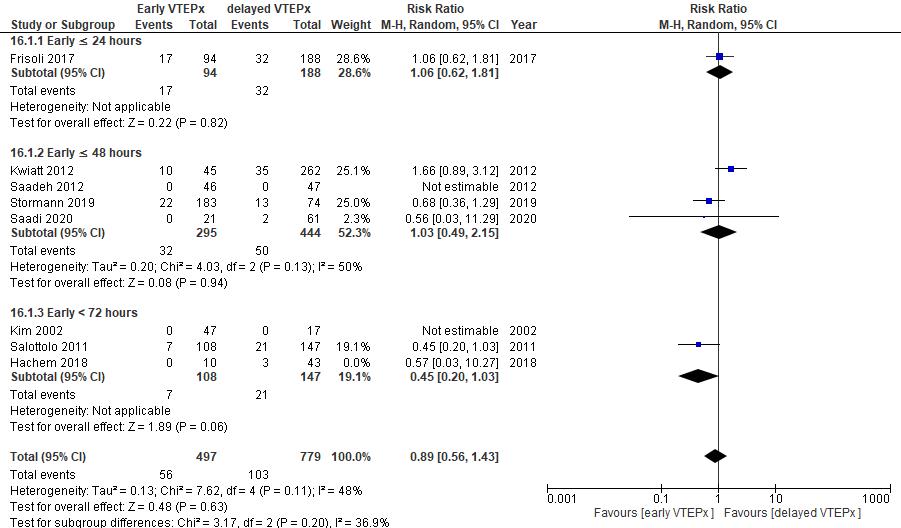


1. **Rate of acute surgical intervention [craniotomies/ craniectomies, EVD , ICP monitor ] after starting PVTEPx  to control hemorrhagic progression**

* Significant heterogeneity is seen between studies and between subgroups.


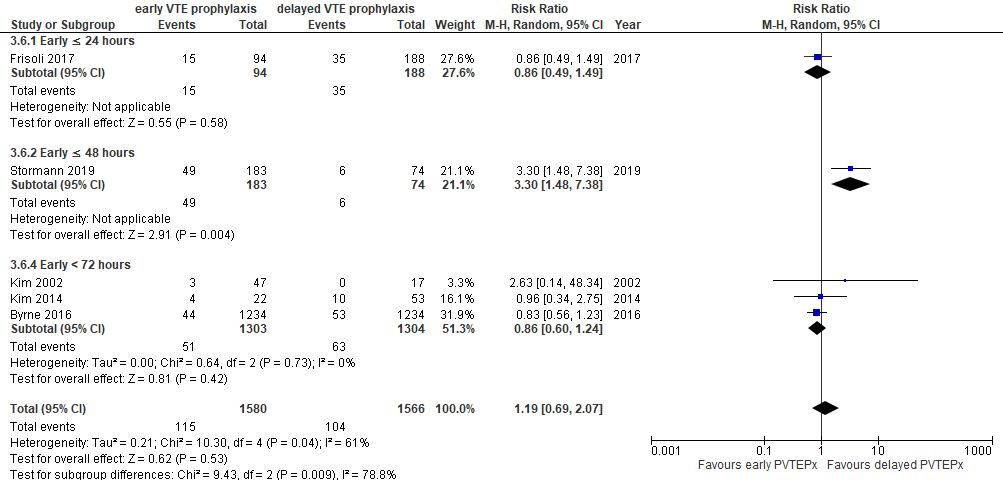


**Sensitivity analysis for acute surgical intervention:**

Removed a study had a mixed patient population, poly-trauma [Kim 2002], therefore, only two studies left in subgroup early <72 hours.

Did not resolve heterogeneity


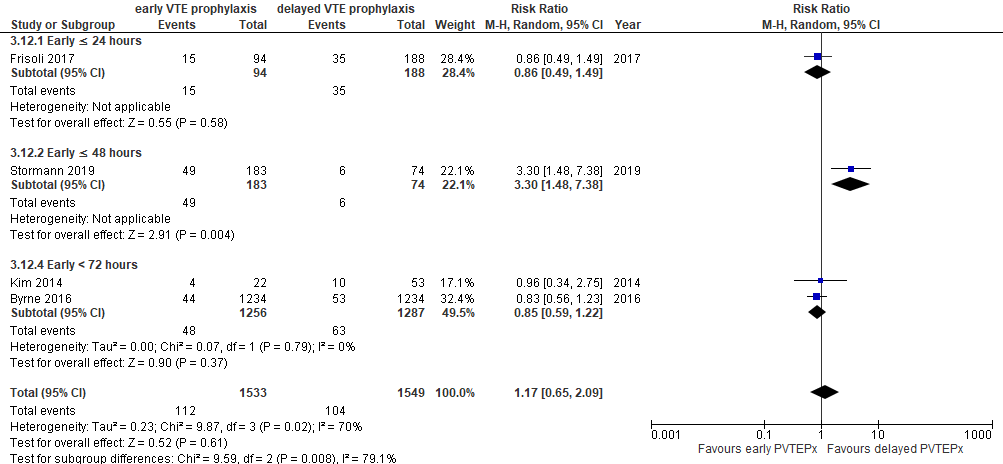


Excluding the studies that has intermediate category (Stormann 2019, Kim 2014 ) resolve heterogeneity in **acute surgical intervention outcome**


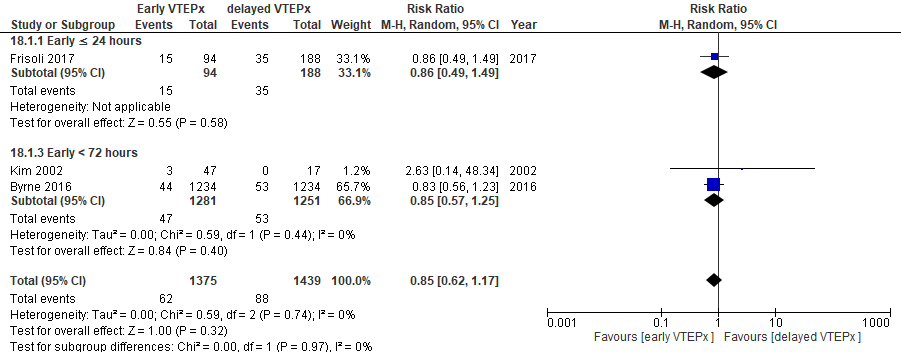


**Evidence Profile Recommendation 4: TBI requiring neurosurgical intervention such as** **intracranial pressure (ICP) monitoring or external ventricular drain (EVD) or craniotomy or craniectomy?**

<https://guidelines.gradepro.org/profile/v7SWl8qQGJs>

**Metanalysis for TBI requiring neurosurgical intervention**

1. **Mortality**

* Note, study Byrne 2021 was entered as two studies in meta-analyses: Byrne 2021 (Craniotomy/craniectomy subgroup), & Byrne 2021 (Intracranial monitor/drain subgroup), but counted as one study in summary table.


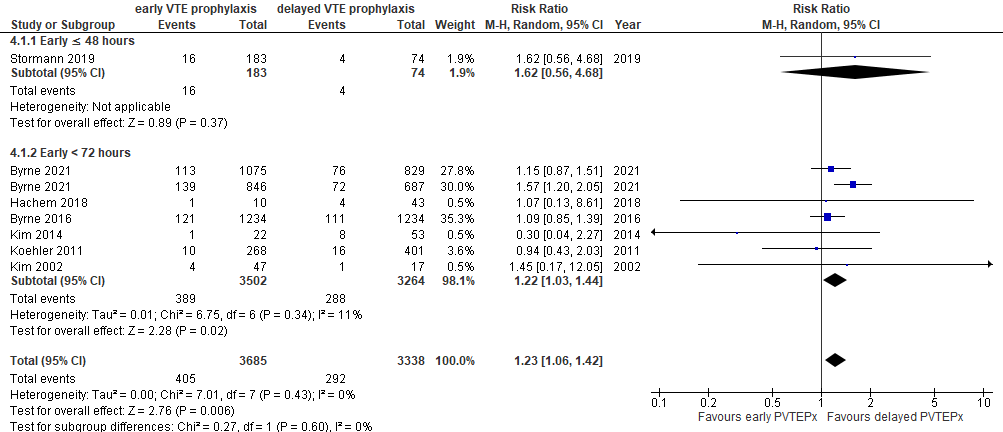


**Sensitivity analysis for mortality:**

Removed studies had a mixed patient population (blunt and penetrating TBI) , poly-trauma [Hachem 2018, Kim 2002, Koehler 2011], therefore, only four studies left in subgroup early <72 hours, this subgroup no longer shows significant difference between two arms. The overall effect size changed to border line significant: RR 1.24 (95%CI 1.00 to 1.54, p=0.05).


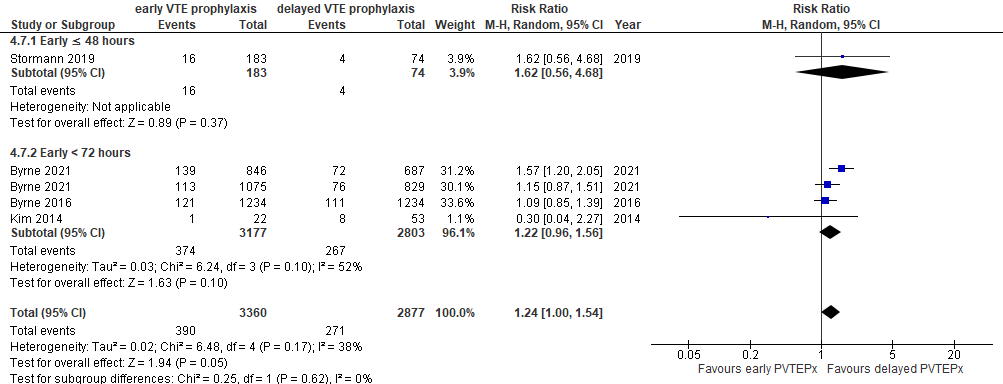


1. **VTE:**

All studies were in the subgroup of <72 hours

**
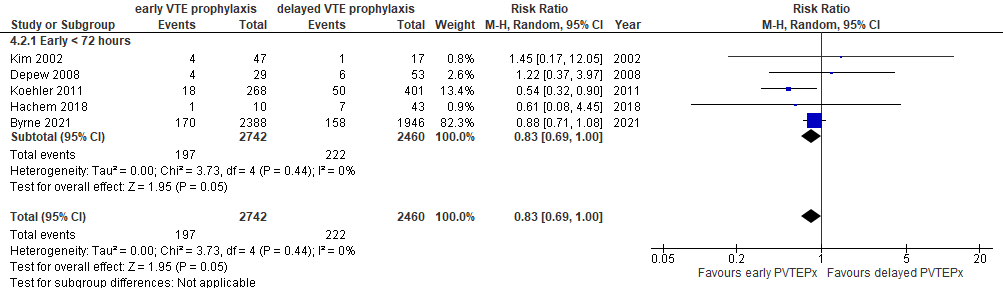
**

**Sensitivity analysis for VTE:**

Removed studies had a mixed patient population (blunt and penetrating TBI) , poly-trauma [Hachem 2018, Kim 2002, Depew 2008, Koehler 2011].

Therefore, only one study left in the whole analysis (early <72 hours)


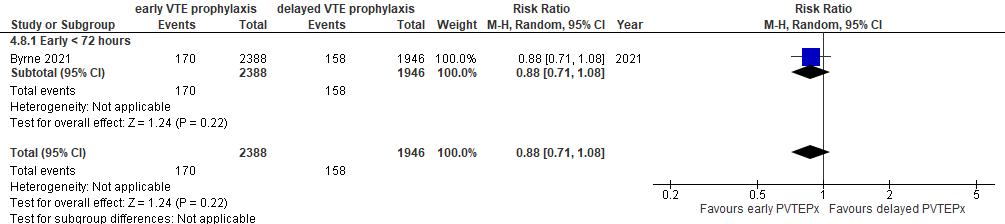


1. **DVT**


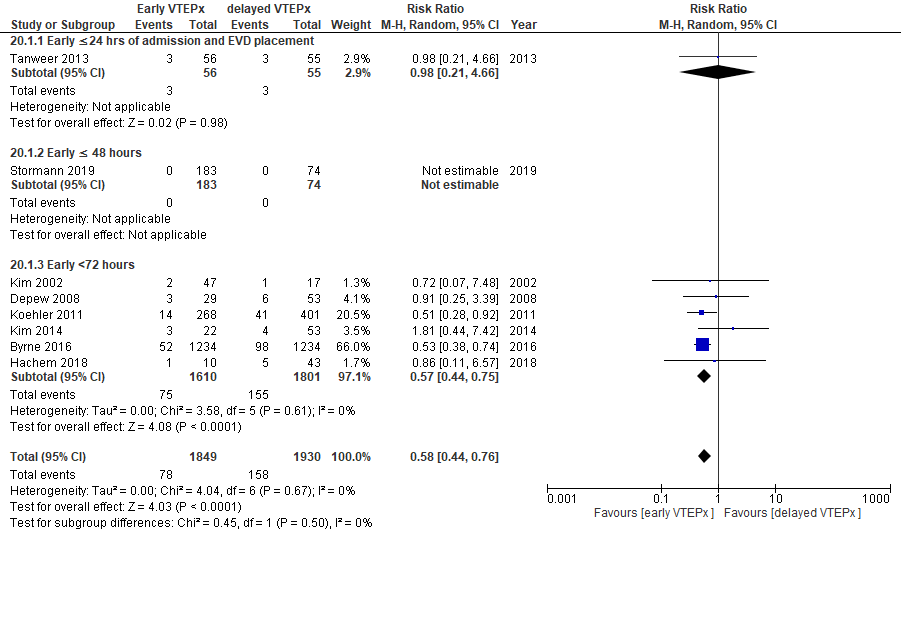


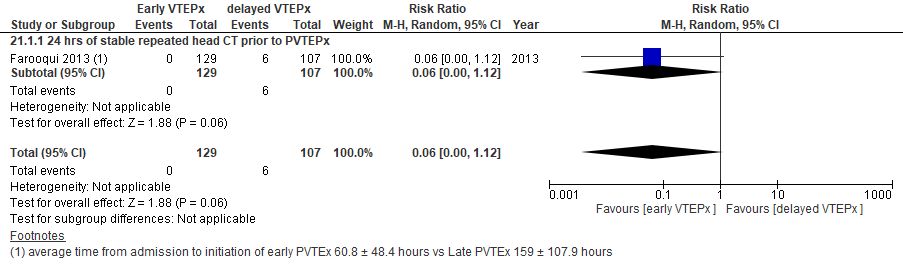


**Sensitivity analysis for DVT:**

Removed studies had a mixed patient population (blunt and penetrating TBI) , poly-trauma [Hachem 2018, Kim 2002, Depew 2008, Koehler 2011], therefore, only two studies left in subgroup early <72 hours.

Overall effect and subgroup of early <72 hours both are no longer significant.


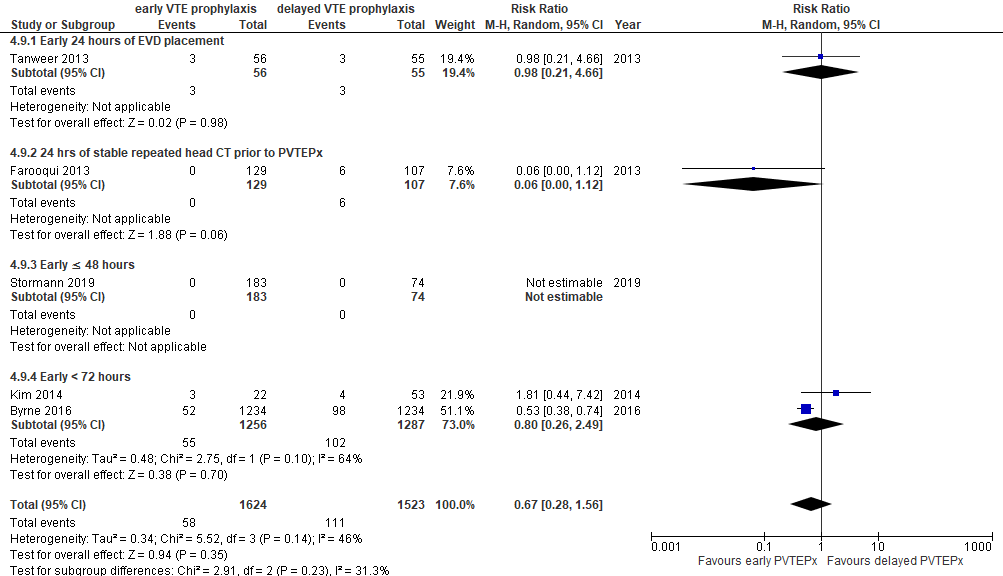


1. **PE:**

**
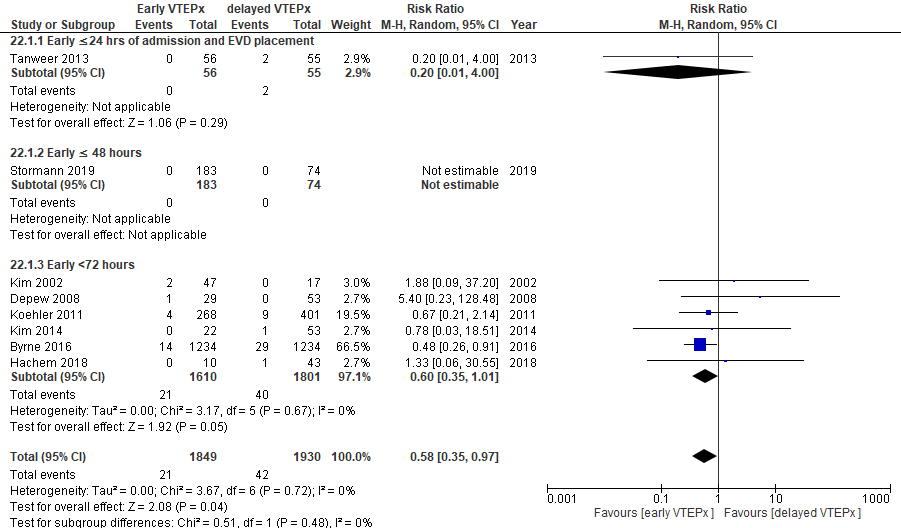
**

**
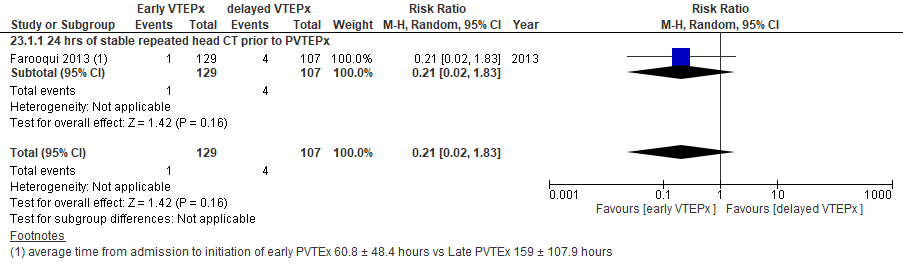
**

**Sensitivity analysis for PE:**

Removed studies had a mixed patient population (blunt and penetrating TBI), poly-trauma [Hachem 2018, Kim 2002, Depew 2008, Koehler 2011], therefore, only two studies left in subgroup early <72 hours.


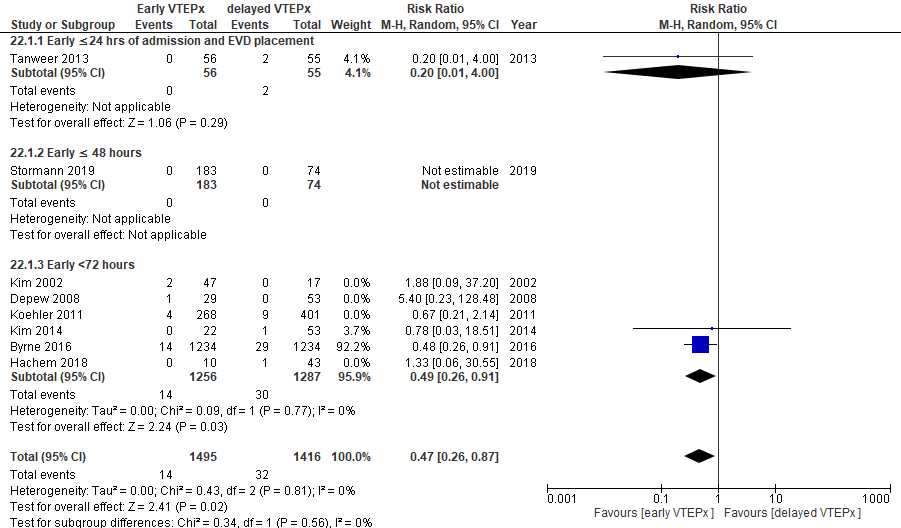


1. **Rates of ICH Progression after starting PVTEPx**

* Please note that for study Koehler 2011, based the number of events on expansion of individual intracranial lesions (480 vs 781) as opposed to the number of patients (268 vs 401).


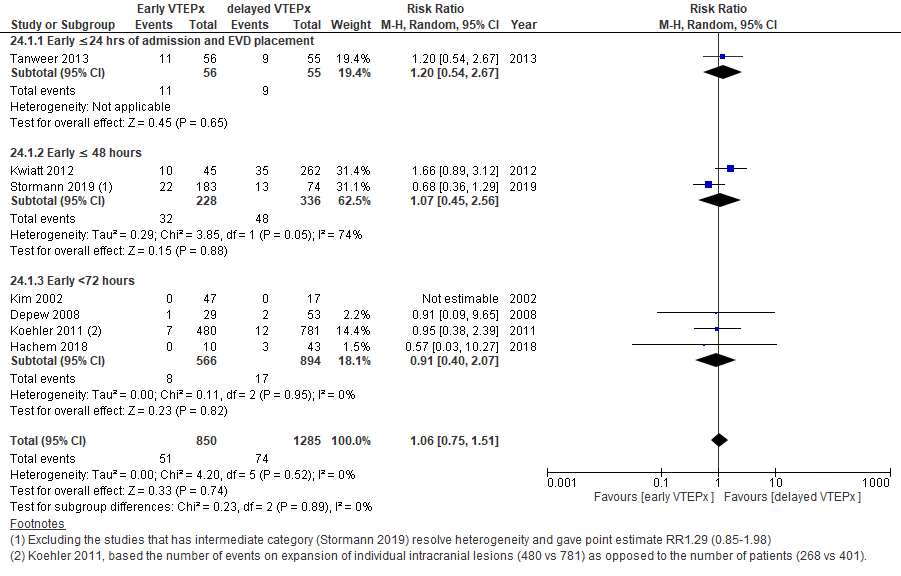


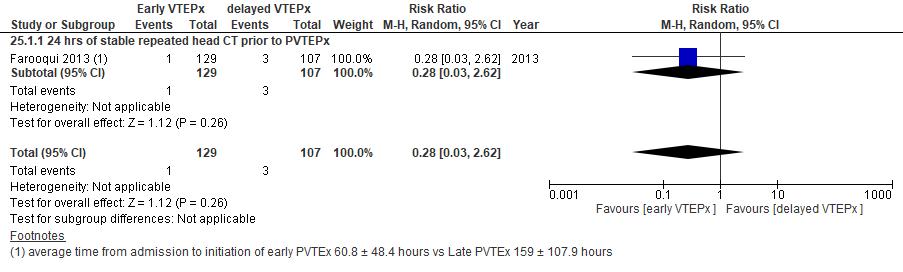


**Sensitivity analysis for ICH Progression after starting PVTEPx:**

Removed studies had a mixed patient population (blunt and penetrating TBI), poly-trauma [Hachem 2018, Kim 2002, Depew 2008, Koehler 2011], therefore, no study left in subgroup early <72 hours.


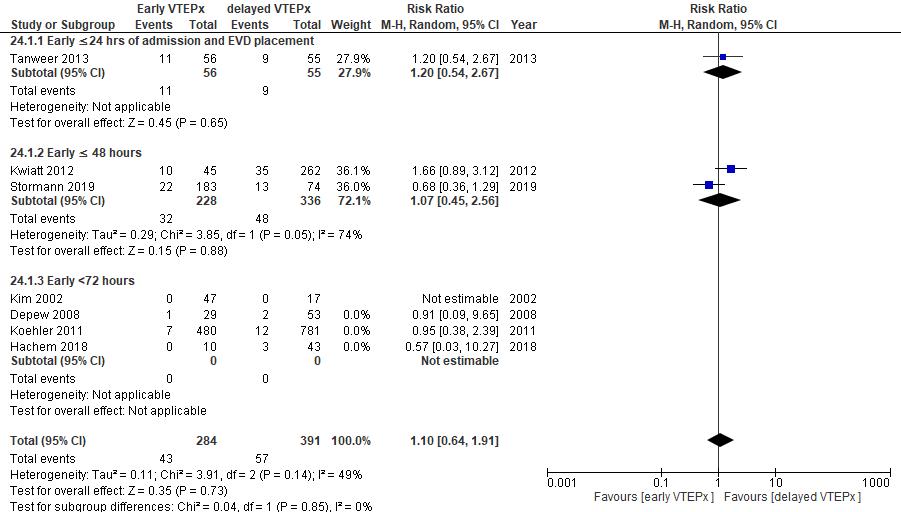


1. **Rate of acute surgical intervention [craniotomies/ craniectomies, EVD, ICP monitor] after starting pharmacologic VTE prophylaxis to control hemorrhagic progression**

* Significant heterogeneity is seen between studies and between subgroups.


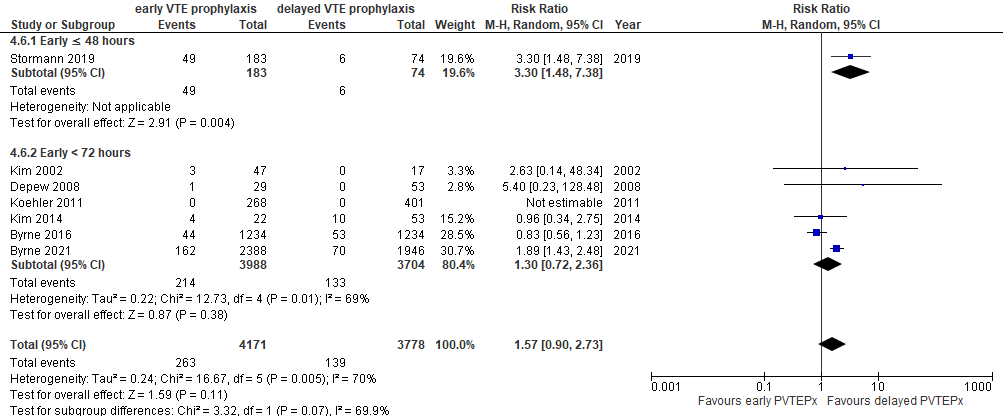


Excluding the studies that has intermediate category (Stormann 2019, Kim 2014) did not resolve heterogeneity (unexplained heterogeneity) and gave point estimate RR1.41 (0.69-2.87)

If unexplained heterogeneity, we considered that in grading the evidence and formulating recommendation

**Sensitivity analysis for acute surgical intervention:**

Removed studies had a mixed patient population (blunt and penetrating TBI), poly-trauma [Kim 2002, Depew 2008, Koehler 2011], therefore, only three studies left in subgroup early <72 hours.

Did not resolve heterogeneity


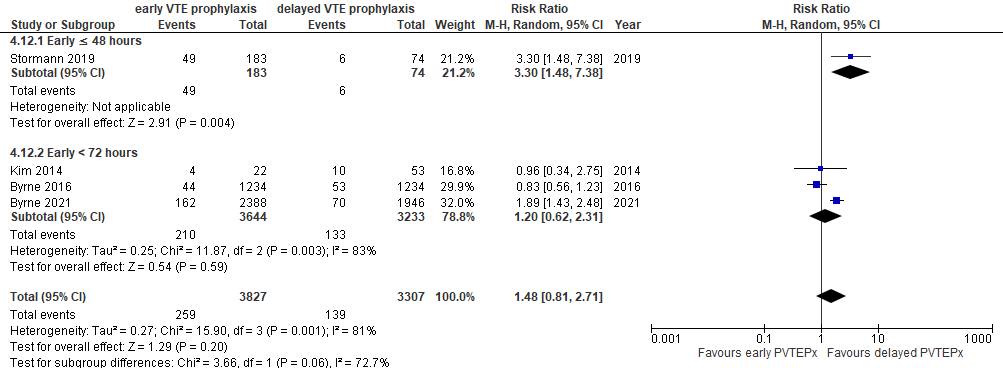


**Table S6.** Evidence to Decision Framework Recommendation 4: TBI ***requiring neurosurgical intervention*** [ intracranial pressure (ICP) monitoring or external ventricular drain (EVD) or craniotomy or craniectomy]

| Question | |
| --- | --- |
| **Should early pharmacologic VTE prophylaxis 24 hours from the procedure and follow-up stable brain imaging vs. delayed pharmacologic VTE prophylaxis (>24 hours) be used for adults with isolated blunt TBI requiring intracranial pressure (ICP) monitoring or external ventricular drain (EVD) or craniotomy or craniectomy?** | |
| **Population:** | adults with isolated blunt TBI requiring intracranial pressure (ICP) monitoring or external ventricular drain (EVD) or craniotomy or craniectomy |
| **Intervention:** | early pharmacologic VTE prophylaxis 24 hours from the procedure and follow-up stable brain imaging |
| **Comparison:** | delayed pharmacologic VTE prophylaxis (>24 hours) |
| **Main outcomes:** | **Critical outcomes**:   - Mortality - VTE - DVT - PE - Intracranial bleeding or Hemorrhagic progression after starting Pharmacological VTEPx   **Important outcomes:**   - Rate of **repeated** neurosurgical interventions (craniotomies/ craniectomies, EVD , ICP monitor) after starting Pharmacological VTEPx to control hemorrhagic progression. *Surrogate for Intracranial bleeding or hematoma expansion* - Worsening of neuro examination after starting Pharmacological VTEPx “Drop in baseline GCS “. *Surrogate for Intracranial bleeding or hematoma expansion* - clinically significant extracranial hemorrhage [GIB, bleeding with solid organ injuries] after starting Pharmacological VTEPx |
| **Setting:** | inpatient |
| **Perspective:** | clinical recommendation - population perspective |
| **Background:** | Patients who undergo urgent neurosurgical interventions such as decompressive craniotomy/craniectomy or intracranial monitor/drain insertion are at highest risk for ICH progression.  Extrapolation from elective craniotomy population and elective neurosurgeries is difficult given underlying different disease etiologies, different baseline VTE risk and ICH progression risk, elective setting, different mechanisms of injury to the brain parenchyma, and lack of concurrent injuries.  This EtD assess whether early pharmacologic VTE prophylaxis 24 hours from the procedure and follow-up stable brain imaging vs. delayed pharmacologic VTE prophylaxis (>24 hours) for adults with isolated **blunt TBI requiring intracranial pressure (ICP) monitoring or external ventricular drain (EVD) or craniotomy or craniectomy** |
| **Conflict of interests:** | None |

# Assessment

| Problem Is the problem a priority? | | |
| --- | --- | --- |
| Judgement | Research evidence | Additional considerations |
| ○ No ○ Probably no ○ Probably yes ● Yes ○ Varies ○ Don't know | TBI patients requiring emergent neurosurgical procedure craniotomy/ICP monitor have been described as a high risk for spontaneous hemorrhage expansion. About **64.2% demonstrated spontaneous progression of their TBI patterns,with 10.5% continuing to progress at 72 hours after injury** (1)    Yet it is well known that the rate of VTE in TBI patients with an ICP monitor is reported to be up to 25%. Allen et al 2020 reported that there was a nearly two-fold higher associated risk for VTE in patients with ICP monitor even when controlling for known VTE risk factors.(2)  WTA, AAST, neurocritical care guideline recommendations regarding the initiation of pharmacologic VTE prophylaxis after TBI lack specificity as a result of minimal data, particularly in TBI requiring intracranial pressure (ICP) monitoring or external ventricular drain (EVD) or craniotomy or craniectomy (3)(4)(5) |  |
| Desirable Effects How substantial are the desirable anticipated effects? | | |
| Judgement | Research evidence | Additional considerations |
| ○ Trivial ○ Small ● Moderate ○ Large ○ Varies ○ Don't know | **Byrne et al JAMA Surgery 2021** (6)  **Multivariable model for VTE in craniotomy/Craniectomy Subgroup:** Prophylaxis Delay (each additional day) associated with odd of VTE 1.07 ( CI 1.01 – 1.15)  **Multivariable model for VTE in intracranial Monitor/Drain Subgroup:** Prophylaxis Delay (each additional day) associated w odd of VTE 1.08 ( CI 1.02– 1.14)    **SRMA of observational studies**  **VTE- stratified by definition of early < 72 hours (5 studies)**   - Based on 5 observational studies (N= 5202), 75 out of 1000 patients in early VTEPx will have VTE and 925 would not vs. 90 out of 1000 patients in late VTEPx will have VTE and 910 would not. Difference 15 fewer per 1000 patients **(1.5 % reduction in VTE)** - This represents ~ 2.7% reduction assuming baseline VTE risk in moderate VTE risk population( 25%)   **DVT- stratified by definition of early ≤ 24 hours (1 study) ≤ 48 hours ( 1 study), < 72 hours (6 studies)**   - Based on 8 observational studies (N= 3779), 48 out of 1000 patients in early VTEPx will have DVT and 952 would not vs. 82 out of 1000 patients in late VTEPx will have DVT and 918 would not. Difference 34 fewer per 1000 patients **(3.4 % reduction in DVT)** - This represents ~ 3.3% reduction assuming baseline DVT risk in low DVT risk population( 15%) and 7.8% reduction from baseline VTE risk in high VTE risk population( 25%)   **PE- stratified by definition of early ≤ 24 hours (1 study) ≤ 48 hours ( 1 study), < 72 hours (6 studies)**   - Based on 8 observational studies (N= 3779 ), 13 out of 1000 patients in early VTEPx will have PE and 987 would not vs. 22 out of 1000 patients in late VTEPx will have PE and 978 would not. Difference 9 fewer per 1000 patients **(0.9 % reduction in PE)** . This represents ~ 0.4% reduction assuming baseline PE risk in this population is 3%   **from Farooqui 2013 et al 24 hrs of stable repeated brain CT following neurosurgical procedure and prior to PVTEPx (average time from admission to initiation 60.8 ± 48.4 hours)**  **DVT :** Based on 1 observational study (N= 236), 3 out of 1000 patients in early VTEPx will have DVT and 997 would not vs. 56 out of 1000 patients in late VTEPx will have DVT and 944 would not. Difference 53 fewer per 1000 patients **(5.3 % reduction in DVT)**  **PE:** Based on 1 observational study (N= 236), 8 out of 1000 patients in early VTEPx will have PE and 992 would not vs. 37 out of 1000 patients in late VTEPx will have PE and 963 would not. Difference 29 fewer per 1000 patients **(2.9% reduction in PE)**     \| **Outcomes** \| **With delayed pharmacologic VTE prophylaxis (>24 hours)** \| **With early pharmacologic VTE prophylaxis 24 hours from the procedure and follow-up stable CT brain** \| **Difference** \| **Relative effect (95% CI)** \| \| --- \| --- \| --- \| --- \| --- \| \| VTE- from SRMA of observational studies- stratified by definition of early < 72 hours (5 studies) \| 90 per 1,000 \| **75 per 1,000** (62 to 90) \| **15 fewer per 1,000** (28 fewer to 0 fewer) \| **RR 0.83** (0.69 to 1.00) \| \| DVT- from SRMA of observational studies- stratified by definition of early ≤ 24 hours (1 study) ≤ 48 hours ( 1 study), < 72 hours (6 studies) \| 82 per 1,000 \| **47 per 1,000** (36 to 62) \| **34 fewer per 1,000** (46 fewer to 20 fewer) \| **RR 0.58** (0.44 to 0.76) \| \| DVT- from Farooqui 2013 et al- 24 hrs of stable repeated brain CT post neurosurgical procedure and prior to PVTEPx (average time from admission to initiation 60.8 ± 48.4 hours) \| 56 per 1,000 \| **3 per 1,000** (0 to 63) \| **53 fewer per 1,000** (56 fewer to 7 more) \| **RR 0.06** (0.00 to 1.12) \| \| PE- from SRMA of observational studies- stratified by definition of early ≤ 24 hours (1 study) ≤ 48 hours ( 1 study), < 72 hours (6 studies) \| 22 per 1,000 \| **13 per 1,000** (8 to 21) \| **9 fewer per 1,000** (14 fewer to 1 fewer) \| **RR 0.58** (0.35 to 0.97) \| \| PE- from Farooqui 2013 et al- 24 hrs of stable repeated brain CT post neurosurgical procedure and prior to PVTEPx (average time from admission to initiation 60.8 ± 48.4 hours) \| 37 per 1,000 \| **8 per 1,000** (1 to 68) \| **30 fewer per 1,000** (37 fewer to 31 more) \| **RR 0.21** (0.02 to 1.83) \| |  |
| Undesirable Effects How substantial are the undesirable anticipated effects? | | |
| Judgement | Research evidence | Additional considerations |
| ○ Large ● Moderate ○ Small ○ Trivial ○ Varies ○ Don't know | **Byrne et al JAMA Surgery** **2021** (6)  **Multivariable model for Repeat NSx in Craniotomy/Craniectomy Subgroup :** Prophylaxis Delay (each additional day) associated w Odds of Repeat Neurosurgery 0.49 ( CI 0.27 – 0.90) in 0-2 days  **Multivariable model for Repeat NSx in Craniotomy/Craniectomy Subgroup** : Prophylaxis Delay (each additional day) associated w Odds of Repeat Neurosurgery 0.75 ( CI 0.75 – 0.92) for after 2 days  **Multivariable model for Repeat NSx in Intracranial Monitor/Drain Subgroup** : Prophylaxis Delay (each additional day) associated w Odds of Repeat Neurosurgery 0.82 ( CI 0.74 – 0.90)    **SRMA of observational studies**  **Rates of ICH Progression after starting PVTEPx- stratified by definition of early ≤ 24 hours (1 study) ≤ 48 hours ( 2 studies), < 72 hours (4 studies)**   - Based on 8 observational studies (N= 2135 ), 61 out of 1000 patients in early VTEPx will have ICH progression and 939 would not vs. 58 out of 1000 patients in late VTEPx will have ICH progression and 942 would not. Difference 3 more per 1000 patients **(0.3% increase in ICH progression**)   **Rate of acute surgical interventions [craniotomies, craniectomies, EVD, ICP] after PVTEPx - stratified by definition of early ≤ 48 hours ( 1 studies), < 72 hours (6 studies)**   - Based on 7 observational studies (N= 7949 ), 58 out of 1000 patients in early VTEPx will need acute neurosurgical intervention and 942 would not vs. 37 out of 1000 patients in late VTEPx will need acute neurosurgical intervention and 963 would not. Difference 21 more per 1000 patients (**2.1 % increase in acute neurosurgical intervention** )   **All-cause mortality- stratified by definition of early ≤ 48 hours (1 study), < 72 hours (6 studies)**   - Based on 7 observational studies (N= 7023 ), 107 out of 1000 patients in early VTEPx will die and 893 would not vs. 87 out of 1000 patients in late VTEPx will die and 913 would not. Difference 20 more per 1000 patients (**2 % increase in death rate** )   **from Farooqui 2013 et al 24 hrs of stable repeated brain CT following neurosurgical procedure and prior to PVTEPx (average time from admission to initiation 60.8 ± 48.4 hours)**  **Rate of ICH progression after starting PVTEPx :** Based on 1 observational study (N= 236), 8 out of 1000 patients in early VTEPx will have ICH progression and 992 would not vs. 28 out of 1000 patients in late VTEPx will have ICH progression and 972 would not. Difference 20 fewer per 1000 patients **(2 % reduction in ICH progression)**   \| **Outcomes** \| **With delayed pharmacologic VTE prophylaxis (>24 hours)** \| **With early pharmacologic VTE prophylaxis 24 hours from the procedure and follow-up stable CT brain** \| **Difference** \| **Relative effect (95% CI)** \| \| --- \| --- \| --- \| --- \| --- \| \| Rates of ICH Progression after starting PVTEPx- from SRMA of observational studies- stratified by definition of early ≤ 24 hours (1 study) ≤ 48 hours ( 2 studies), < 72 hours (4 studies) \| 58 per 1,000 \| **61 per 1,000** (43 to 87) \| **3 more per 1,000** (14 fewer to 29 more) \| **RR 1.06** (0.75 to 1.51) \| \| Rate of ICH progression after starting PVTEPx- from Farooqui 2013 et al- 24 hrs of stable repeated brain CT post neurosurgical procedure and prior to PVTEPx (average time from admission to initiation 60.8 ± 48.4 hours) \| 28 per 1,000 \| **8 per 1,000** (1 to 73) \| **20 fewer per 1,000** (27 fewer to 45 more) \| **RR 0.28** (0.03 to 2.62) \| \| Rate of acute surgical interventions [craniotomies, craniectomies, EVD, ICP] after PVTEPx - from SRMA observational studies- stratified by definition of early ≤ 48 hours ( 1 studies), < 72 hours (6 studies) \| 37 per 1,000 \| **58 per 1,000** (33 to 100) \| **21 more per 1,000** (4 fewer to 64 more) \| **RR 1.57** (0.90 to 2.73) \| \| All-cause mortality- from SRMA of observational studies- stratified by definition of early ≤ 48 hours (1 study), < 72 hours (6 studies) \| 87 per 1,000 \| **108 per 1,000** (93 to 124) \| **20 more per 1,000** (5 more to 37 more) \| **RR 1.23** (1.06 to 1.42) \| \| Worsening of neuro examination after starting PVTEPx “Drop in baseline GCS" [Surrogate for Intracranial bleeding or hematoma expansion ] \| **no study identified addressing this outcome** \| \| \| \| \| Clinically significant extracranial hemorrhage \| **no study identified addressing this outcome** Kim et al., 2002 Extra-cranial bleeding (hematuria-nonsignificant) 3/47 (6.4%) in early vs. 1/17 (5.8%) in late \| \| \| \| | Panel comments:  Based on the SR, mortality is higher with early prophylaxis. This would be a serious outcome, in patients with low mortality rate    One panel member stated that not all ICH progression required surgical intervention |
| Certainty of evidence What is the overall certainty of the evidence of effects? | | |
| Judgement | Research evidence | Additional considerations |
| ● Very low ○ Low ○ Moderate ○ High ○ No included studies | \| **Outcomes** \| **Importance** \| **Certainty of the evidence (GRADE)** \| \| --- \| --- \| --- \| \| VTE- from SRMA of observational studies- stratified by definition of early < 72 hours (5 studies) \| CRITICAL \| ⨁◯◯◯ Very low^a,b,c^ \| \| DVT- from SRMA of observational studies- stratified by definition of early ≤ 24 hours (1 study) ≤ 48 hours ( 1 study), < 72 hours (6 studies) \| CRITICAL \| ⨁◯◯◯ Very low^c,d^ \| \| DVT- from Farooqui 2013 et al- 24 hrs of stable repeated brain CT post neurosurgical procedure and prior to PVTEPx (average time from admission to initiation 60.8 ± 48.4 hours) \| CRITICAL \| ⨁◯◯◯ Very low^e,f^ \| \| PE- from SRMA of observational studies- stratified by definition of early ≤ 24 hours (1 study) ≤ 48 hours ( 1 study), < 72 hours (6 studies) \| CRITICAL \| ⨁◯◯◯ Very low^c,g,h^ \| \| PE- from Farooqui 2013 et al- 24 hrs of stable repeated brain CT post neurosurgical procedure and prior to PVTEPx (average time from admission to initiation 60.8 ± 48.4 hours) \| CRITICAL \| ⨁◯◯◯ Very low^f^ \| \| Rates of ICH Progression after starting PVTEPx- from SRMA of observational studies- stratified by definition of early ≤ 24 hours (1 study) ≤ 48 hours ( 2 studies), < 72 hours (4 studies) \| CRITICAL \| ⨁◯◯◯ Very low^f,i,j^ \| \| Rate of ICH progression after starting PVTEPx- from Farooqui 2013 et al- 24 hrs of stable repeated brain CT post neurosurgical procedure and prior to PVTEPx (average time from admission to initiation 60.8 ± 48.4 hours) \| CRITICAL \| ⨁◯◯◯ Very low^f^ \| \| Rate of acute surgical interventions [craniotomies, craniectomies, EVD, ICP] after PVTEPx - from SRMA observational studies- stratified by definition of early ≤ 48 hours ( 1 studies), < 72 hours (6 studies) \| CRITICAL \| ⨁◯◯◯ Very low^c,k,l,m^ \| \| All-cause mortality- from SRMA of observational studies- stratified by definition of early ≤ 48 hours (1 study), < 72 hours (6 studies) \| CRITICAL \| ⨁◯◯◯ Very low^c,n^ \|  1. We downgraded by one level for indirectness. Some studies included polytrauma patients (not isolated blunt TBI). sensitivity analysis after excluding polytrauma patients (Hachem 2018, Kim 2002, Depew 2008, Koehler 2011) gave estimate RR 0.88 (95% CI 0.71-1.08) 2. Downgraded by 1 level due to wide CI - crosses line of no effect 3. we did not downgrade for ROB as the effect estimate comes from the largest study Byrne 2021 ( Early within 3 days after urgent neurosurgical intervention) vs. late (median > 3 days after urgent neurosurgical intervention) which is low ROB study 4. We downgraded by one level for indirectness. Some studies included polytrauma patients (not isolated blunt TBI). sensitivity analysis after excluding polytrauma patients (Hachem 2018, Kim 2002, Depew 2008, Koehler 2011) gave estimate RR 0.67 (95% CI 0.28-1.56) 5. Only 1 study reporting data - unable to compare consistency across studies 6. Downgraded by 2 level as the total numbers of events was small and confidence interval is wide to reliably estimate an effect 7. We downgraded by one level for indirectness. Some studies included polytrauma patients (not isolated blunt TBI). Sensitivity analysis after excluding polytrauma patients (Hachem 2018, Kim 2002, Depew 2008, Koehler 2011) gave similar estimate RR 0.47 (95% CI 0.26- 0.87) 8. downgraded by 1 level due to few events to reliably estimate an effect 9. We downgraded by one level for indirectness. Some studies included polytrauma patients (not isolated blunt TBI). sensitivity analysis after excluding polytrauma patients (Hachem 2018, Kim 2002, Depew 2008, Koehler 2011) gave similar estimate RR 1.10 (95% CI 0.64- 1.91) 10. We downgraded by one level for serious risk of bias due to bias in selection of participants into the study (more severe form of TBI and lower GCS are likely getting late PVTEPx). Also some studies reported Follow-up CT scans in ~80% (bias due to missing data). 11. downgraded by 1 level due to significant heterogeneity I2 70 % (unexplained heterogeneity) . Excluding the studies that has intermediate category (Stormann 2019, Kim 2014 ) did not resolve heterogeneity and gave point estimate RR1.41 (0.69-2.87) 12. We downgraded by one level for indirectness. Some studies included polytrauma patients. Sensitivity analysis excluding studies with poly-trauma (Kim 2002, Depew 2008, Koehler 2011) gave RR 1.48 (95% CI 0.81- 2.71) 13. We did not downgrade for imprecision. Although 95% CI crosses 1, the absolute difference range from almost 0 to 6.4 increase in harm 14. We downgraded by one level for indirectness. Some studies included polytrauma patients. Sensitivity analysis excluding studies with poly-trauma (Hachem 2018, Kim 2002, Koehler 2011) gave RR 1.24 (95% CI 1- 1.54) |  |
| Values Is there important uncertainty about or variability in how much people value the main outcomes? | | |
| Judgement | Research evidence | Additional considerations |
| ● Important uncertainty or variability ○ Possibly important uncertainty or variability ○ Probably no important uncertainty or variability ○ No important uncertainty or variability | It is likely that Important uncertainty or variability exists for how most patients would value the benefits of VTE, DVT, PE risk reduction while there is likely increase in undesirable effect ( increase in acute and repeated neurosurgical intervention, **i**ncrease in ICH progression , and increase in mortality)    Some studies reported the need for acute neurosurgical intervention or in-hospital mortality as a surrogate marker for clinically significant worsening of intracranial hemorrhage (indirect outcome for ICH progression which possibly speculative)  **Saudi patient perspective:**  patient valued 1.5 % reduction in VTE, 3.4% reduction in DVT, and about 1 % reduction in PE associated with early use of pharmacological VTE prophylaxis in TBI patient requiring emergent neurosurgical intervention. This time, the patient express hesitation and was a bit concerned about moderate undesirable effect including possible increase in ICH progression by 0.3% , increase in OR returns by 2% and increase in death rate by 2 %. Patient understood that 64.2% of head injuries requiring emergent neurosurgical procedure demonstrated spontaneous progression of their TBI patterns. The patient indicated his/her preference to have a CT brain prior to pharmacological VTEPx initiation and if head hemorrhage demonstrated in repeated brain CT, he/she would prefer to avoid early pharmacological VTEPx | Panel comments:  one panel member stated that Mortality in TBI is multifactorial and cannot be swayed toward a singular factor like ICH progression after DVT prophylaxis |
| Balance of effects Does the balance between desirable and undesirable effects favor the intervention or the comparison? | | |
| Judgement | Research evidence | Additional considerations |
| ○ Favors the comparison ○ Probably favors the comparison ○ Does not favor either the intervention or the comparison ○ Probably favors the intervention ○ Favors the intervention ○ Varies ● Don't know | - There is a possible **reduction** in VTE 1.5% with imprecise magnitude - There is a possible **reduction** in DVT **~** 3.4% - There is a possible **reduction** in PE**~** 0.9% but of imprecise due to few events to reliably estimate the effect - There is an **unclear effect in ICH progression** 0.3% increase from SRMA while 2% reduction in ICH progression from Farooqui 2013 et al 24 hrs of stable repeated brain CT following neurosurgical procedure and prior to PVTEPx with serious imprecise magnitude - There is **increase** effect on rate of acute neurosurgical intervention with imprecise magnitude - There is **increase** in all-cause mortality |  |
| Resources required How large are the resource requirements (costs)? | | |
| Judgement | Research evidence | Additional considerations |
| ○ Large costs ○ Moderate costs ○ Negligible costs and savings ○ Moderate savings ○ Large savings ● Varies ○ Don't know | Cost varies depends on the types of pharmacological VTE prophylaxis and resource needed for disease burden and treatment of complications  **Cost for intervention below is based on Saudi FDA cost**    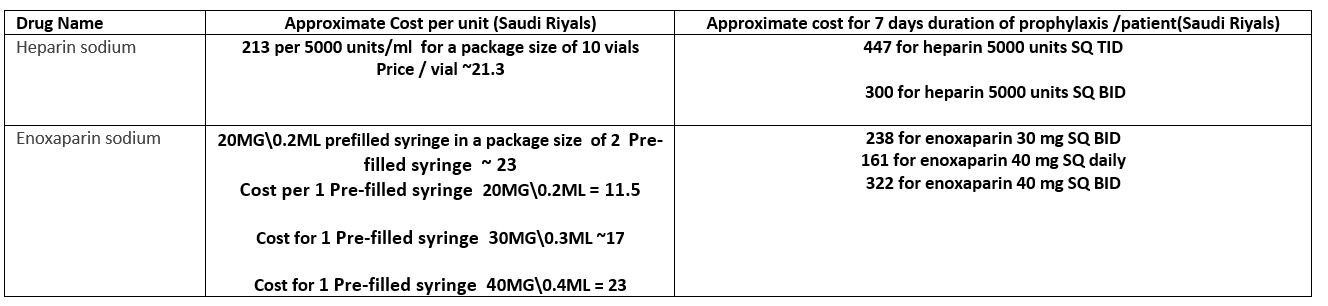 | One panel member voted for Negligible costs and savings |
| Certainty of evidence of required resources What is the certainty of the evidence of resource requirements (costs)? | | |
| Judgement | Research evidence | Additional considerations |
| ○ Very low ○ Low ○ Moderate ○ High ● No included studies | No research evidence identified. |  |
| Cost effectiveness Does the cost-effectiveness of the intervention favor the intervention or the comparison? | | |
| Judgement | Research evidence | Additional considerations |
| ○ Favors the comparison ○ Probably favors the comparison ○ Does not favor either the intervention or the comparison ○ Probably favors the intervention ○ Favors the intervention ● Varies ○ No included studies | **No evidence directly** addresses the cost-effectiveness of early pharmacological VTEPx compared with late pharmacological VTEPx in TBI requiring neurosurgical procedure. Cost-effectiveness also depends on the types of pharmacological VTEPx and resource needed for disease burden and treatment of complications    **INDIRECT EVIDENCE from Patients Undergoing Craniotomy for Brain Tumor** (7)  systematic review with cost-effectiveness analysis (CEA) included 15 studies of various prophylaxis strategies in tumor patients undergoing craniotomy to determine the safest and most cost-effective prophylaxis regimen.  **The estimated cost of treatment (** All costs were reported in 2016 US dollars **)**  $127.47 for mechanical prophylaxis,  $142.20 for mechanical prophylaxis+UFH,  $169.40 for mechanical prophylaxis+LMWH.  **The average cost per quality-adjusted life-year** ( All costs were reported in 2016 US dollars )  $338.39 for mechanical prophylaxis  $284.14 for mechanical prophylaxis +UFH  $722.87 for mechanical prophylaxis+LMWH (However, If VTE probabilities > 5%, MP+LMWH becomes more effective)    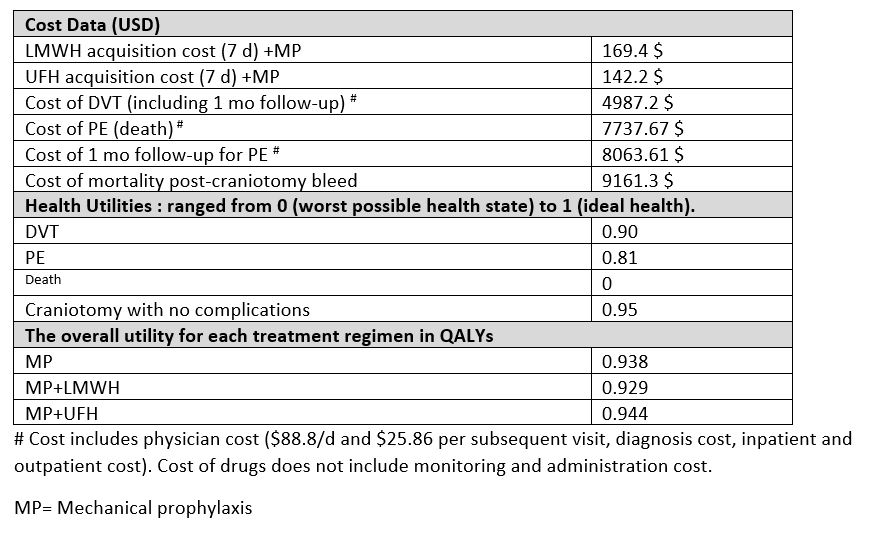 |  |
| Equity What would be the impact on health equity? | | |
| Judgement | Research evidence | Additional considerations |
| ○ Reduced ○ Probably reduced ● Probably no impact ○ Probably increased ○ Increased ○ Varies ○ Don't know | Probably no impact on equity, assuming that prophylaxis would typically be short-term for this population. |  |
| Acceptability Is the intervention acceptable to key stakeholders? | | |
| Judgement | Research evidence | Additional considerations |
| ○ No ○ Probably no ○ Probably yes ○ Yes ● Varies ○ Don't know | Acceptability to key stakeholders probably varies  **In Byrne JAMA Surgery** **2021** (6)   - Delay in initiating pharmacologic VTE prophylaxis was associated with increased risk of thromboembolic complications. - Howeve**r, earlier initiation of prophylaxis was associated with increased risk of repeated neurosurgerical intervention raises concern that the benefit of reduced thromboembolic risk might also come at the cost of increased risk for hemorrhagic complication. This risk appeared to be greatest during the first 3 days after the index procedure** - CT findings or acute neurologic changes are not captured in this study from TQIP registry . Instead, it reported the **need for acute neurosurgical intervention or in-hospital mortality as a surrogate marker for clinically significant worsening of intracranial hemorrhage (indirect outcome for ICH progression )** |  |
| Feasibility Is the intervention feasible to implement? | | |
| Judgement | Research evidence | Additional considerations |
| ○ No ○ Probably no ● Probably yes ○ Yes ○ Varies ○ Don't know | The intervention is judged to be likely feasible  **important barriers:**  survey of 391 Eastern Association for the surgery of Trauma (EAST) trauma surgeons, approximately half reported no standardized protocol at their institution and almost half of responders believe that their institution’s practice is too conservative. In a case-based question, approximately 80% responded that they would initiate VTE prophylaxis within 48 hours with evidence of a stable CT scan, but it was noted that surgeons credentialed for <10 years were more likely to start prophylaxis earlier than those credentialed >10 years (8) |  |

# Summary of judgements

|  | **Judgement** | | | | | | |
| --- | --- | --- | --- | --- | --- | --- | --- |
| **Problem** | No | Probably no | Probably yes | **Yes** |  | Varies | Don't know |
| **Desirable Effects** | Trivial | Small | **Moderate** | Large |  | Varies | Don't know |
| **Undesirable Effects** | Large | **Moderate** | Small | Trivial |  | Varies | Don't know |
| **Certainty of evidence** | **Very low** | Low | Moderate | High |  |  | No included studies |
| **Values** | **Important uncertainty or variability** | Possibly important uncertainty or variability | Probably no important uncertainty or variability | No important uncertainty or variability |  |  |  |
| **Balance of effects** | Favors the comparison | Probably favors the comparison | Does not favor either the intervention or the comparison | Probably favors the intervention | Favors the intervention | Varies | **Don't know** |
| **Resources required** | Large costs | Moderate costs | Negligible costs and savings | Moderate savings | Large savings | **Varies** | Don't know |
| **Certainty of evidence of required resources** | Very low | Low | Moderate | High |  |  | **No included studies** |
| **Cost effectiveness** | Favors the comparison | Probably favors the comparison | Does not favor either the intervention or the comparison | Probably favors the intervention | Favors the intervention | **Varies** | No included studies |
| **Equity** | Reduced | Probably reduced | **Probably no impact** | Probably increased | Increased | Varies | Don't know |
| **Acceptability** | No | Probably no | Probably yes | Yes |  | **Varies** | Don't know |
| **Feasibility** | No | Probably no | **Probably yes** | Yes |  | Varies | Don't know |

# Conclusions

| **Statement 4:** There is insufficient evidence to issue a recommendation on the use of early pharmacologic VTE prophylaxis in adults with isolated blunt TBI requiring neurosurgical intervention (including craniectomy, craniotomy, EVD, or ICP monitoring).  **Best practice statement (BPS)**  The SCCS guideline panel agrees that best practice includes withholding early pharmacologic VTE prophylaxis until follow-up brain imaging (e.g., brain CT) demonstrates no progression. If progression is demonstrated, we agree that best practice includes continuation of mechanical VTE prophylaxis (if no contradictions) and prophylactic IVCF and/or US screening to be considered (BPS).  The SCCS guideline panel agrees that best practice includes evaluation of timely initiation of pharmacologic VTE prophylaxis by multidisciplinary teams (trauma, neuro/neurosurgical, critical care, and clinical pharmacist) (BPS). |
| --- |
| Related recommendation(s) **1. Should mechanical prophylaxis with intermittent pneumatic compression (IPC) vs. no mechanical prophylaxis be used for adults with trauma who are not candidate pharmacologic VTE prophylaxis?**  In adults with trauma who are not candidate pharmacologic VTE prophylaxis, the SCCS guideline panel recommends using mechanical prophylaxis with Intermittent pneumatic compression over no mechanical prophylaxis when not contraindicated by lower extremity injury (strong recommendation, very low certainty in the evidence).  **Remarks:**  T**his is the practice of majority of SCCS panel when there is insufficient evidence to support a recommendation**  Pharmacological prophylaxis should be delayed in patients with clinically active bleeding or coagulopathy, until these conditions have been controlled or reversed. Mechanical prophylaxis should be employed in these patients.  **2. Should we recommend routine VTE ultrasonography (US) screening vs. No routine surveillance be used for adults with trauma who are not candidate pharmacologic VTE prophylaxis?**  In adults with trauma who are at high-risk of VTE and are not candidate for pharmacologic VTE prophylaxis, the SCCS guideline panel ***suggests*** routine bilateral lower-extremity ultrasonography to screen for asymptomatic DVT over no routine screening (***conditional recommendation, very low certainty of evidence***).  **Remarks:**   - Studies were inconsistent about frequency but reasonable frequency based on large studies is twice weekly (from PROTECT trial) - This recommendation is inapplicable to trauma patients who are ambulating, those at low VTE risk, and patients with signs or symptoms of DVT in whom diagnostic imaging is indicated.   **3. Should we recommend prophylactic inferior vena-cava (IVC) filters vs. no prophylactic IVC filters be used for adults with trauma who are not candidate pharmacologic VTE prophylaxis?**  In adults with trauma who are not candidates for pharmacologic VTE prophylaxis, the SCCS guideline panel ***suggests against*** the routine placement of prophylactic IVC filters (***conditional recommendation, very low certainty evidence***).  **Remark:**  Clinicians may consider using temporary retrievable IVC filters in patients who are expected to be off pharmacologic VTE prophylaxis for > 7 days (e.g. severely injured patients with ongoing bleeding risk). |
| Justification |
| There was insufficient evidence to make a recommendation for or against early pharmacological VTEPx in TBI patients requiring acute neurosurgical intervention. The current evidence is very low certainty leading to an unclear effect whether the balance between desirable and undesirable effects favor the intervention or the comparison.    Earlier initiation of prophylaxis was associated with **increased risk of repeated neurosurgerical intervention** raises concern that the benefit of reduced thromboembolic risk might also come at the cost of increased risk for hemorrhagic complication. This risk appeared to be greatest during the first 3 days after the index procedure. Uncertainty remains for earlier prophylaxis association with **increased mortality** in patients who underwent ICP monitor/drain insertion.  Mortality and repeated neurosurgerical intervention outcomes were used as surrogate marker for clinically significant worsening of intracranial hemorrhage -indirect outcome for ICH progression)  Probably have no impact on equity, probably acceptable, likely feasible, and cost varies according to agents used and the cost needed for repeated neurosurgical procedures  More evidence is need in TBI patients requiring acute neurosurgical intervention before a recommendation for or against early pharmacological VTEPx can be made  4 panel members voted for Strong recommendation against the intervention  4 voted for Conditional recommendation against the intervention  9 voted for Conditional recommendation for either the intervention or the comparison  3 voted Conditional recommendation for the intervention and acknowledging very weak data, low certainty  2 panel stated Case by case, multidisiplinary teams decisions and should be individualized |

| Subgroup considerations |
| --- |
| - Earlier prophylaxis was associated with increased mortality among patients who underwent ICP monitor/drain insertion as the index procedure. This association was not observed in patients who underwent craniotomy/ craniectomy. These data may reflect a greater potential harm of hemorrhage progression in patients selected for ICP monitor/drain procedures possibly owing to injuries that are typically more diffuse, intraparenchymal, or not amenable to cranial decompression. |
| Implementation considerations |
| **For clinician:**  -Patient-specific assessment of the risk and benefit for VTE and bleeding outcomes  **for policymaker :**  Integration with electronic health record systems (order sets) transforming guideline recommendations into actionable items.  Dissemination of guideline recommendations |

| Monitoring and evaluation |
| --- |
| We suggest to periodically reevaluate bleeding risk. Once bleeding is stabilized and the patient is no longer considered at high risk for major bleeding, the use of early pharmacological VTE prophylaxis should be  reconsidered as soon as feasible  Monitoring of the agent pursue was included under PICO question 7 (agent) |
| Research priorities |
| · Further high-quality RCT with adequate power comparing early pharmacological VTE prophylaxis vs late prophylaxis in patients with TBI requiring intracranial pressure (ICP) monitoring or external ventricular drain (EVD) or craniotomy or craniectomy are important to provide greater certainty**. Currently, no ongoing RCTs were identified focusing on TBI requiring neurosurgical intervention**  References Summary 1. Phelan, Herb A., Eastman, Alexander L., Madden, Christopher J., Aldy, Kim, Berne, John D., Norwood, Scott H., Scott, William W., Bernstein, Ira H., Pruitt, Jeffrey, Butler, Gordon, Rogers, Lowery, Minei, Joseph P.. TBI risk stratification at presentation: A prospective study of the incidence and timing of radiographic worsening in the Parkland Protocol. Journal of Trauma and Acute Care Surgery; 08/2012.  2. A, Allen, A, Grigorian, A, Christian, Sd, Schubl, C, Barrios, M, Lekawa, B, Borazjani, V, Joe, J, Nahmias. Intracranial pressure monitors associated with increased venous thromboembolism in severe traumatic brain injury. European journal of trauma and emergency surgery : official publication of the European Trauma Society; 2021 Oct.  3. Ley, Eric J., Brown, Carlos V.R., Moore, Ernest E., Sava, Jack A., Peck, Kimberly, Ciesla, David J., Sperry, Jason L., Rizzo, Anne G., Rosen, Nelson G., Brasel, Karen J., Kozar, Rosemary, Inaba, Kenji, Martin, Matthew J.. Updated guidelines to reduce venous thromboembolism in trauma patients: A Western Trauma Association critical decisions algorithm. Journal of Trauma and Acute Care Surgery; 11/2020.  4. Rappold, Joseph F, Sheppard, Forest R, Carmichael II, Samuel P, Cuschieri, Joseph, Ley, Eric, Rangel, Erika, Seshadri, Anupamaa J, Michetti, Christopher P. Venous thromboembolism prophylaxis in the trauma intensive care unit: an American Association for the Surgery of Trauma Critical Care Committee Clinical Consensus Document. Trauma Surgery & Acute Care Open; 02/2021.  5. Nyquist, Paul, Bautista, Cynthia, Jichici, Draga, Burns, Joseph, Chhangani, Sanjeev, DeFilippis, Michele, Goldenberg, Fernando D., Kim, Keri, Liu-DeRyke, Xi, Mack, William, Meyer, Kim. Prophylaxis of Venous Thrombosis in Neurocritical Care Patients: An Evidence-Based Guideline: A Statement for Healthcare Professionals from the Neurocritical Care Society. Neurocritical Care; 2016-02.  6. Byrne, James P., Witiw, Christopher D., Schuster, James M., Pascual, Jose L., Cannon, Jeremy W., Martin, Niels D., Reilly, Patrick M., Nathens, Avery B., Seamon, Mark J.. Association of Venous Thromboembolism Prophylaxis After Neurosurgical Intervention for Traumatic Brain Injury With Thromboembolic Complications, Repeated Neurosurgery, and Mortality. JAMA Surgery; 2022-03-09.  7. Algattas, Hanna, Damania, Dushyant, DeAndrea-Lazarus, Ian, Kimmell, Kristopher T., Marko, Nicholas F., Walter, Kevin A., Vates, G. Edward, Jahromi, Babak S.. Systematic Review of Safety and Cost-Effectiveness of Venous Thromboembolism Prophylaxis Strategies in Patients Undergoing Craniotomy for Brain Tumor. Neurosurgery; 2018-02-01.  8. Strollo, Brian P., Bennett, Gregory J., Chopko, Michael S., Guo, Weidun Alan. Timing of venous thromboembolism chemoprophylaxis after traumatic brain injury. Journal of Critical Care; 02/2018. |

**Table S7. PICO question: Recommendation 5-6: spine trauma or fracture and/or SCI**

**Recommendation 5: spine trauma or fracture and/or SCI managed non-operatively**

In adults with isolated spine trauma or fracture and/or SCI managed non-operatively, should we recommend early pharmacologic VTE prophylaxis (within 24-48 hours post-injury) versus delayed pharmacologic VTE prophylaxis (>48 hours)?

| Population | Intervention | Comparator | Outcomes |
| --- | --- | --- | --- |
| Adults with isolated spine trauma or fracture and/or SCI, low risk of bleeding and managed non-operatively | Early pharmacologic VTE prophylaxis (within 24-48 hours post-injury) | delayed pharmacologic VTE prophylaxis (> 48 hours) | 1. VTE 2. Intraspinal epidural hematoma development or expansion 3. Need for delayed surgery decompressive laminectomy |

**Recommendation 6: spine trauma or fracture and/or SCI managed operatively**

In adults with spine trauma or fracture and/or SCI managed operatively, should we recommend early pharmacologic VTE prophylaxis (within 48 hours post spinal fixation) versus delayed pharmacologic VTE prophylaxis (> 48hr)?

| Population | Intervention | Comparator | Outcomes |
| --- | --- | --- | --- |
| Adults with spine trauma or fracture and/or SCI, and managed operatively | Early pharmacologic VTE prophylaxis (≤ 48 hours post-operative fixation/ post spine surgery) | delayed pharmacologic VTE prophylaxis (> 48 hours) | 1. VTE 2. Intraspinal or epidural hematoma development or expansion |

**Evidence Profile and Evidence to Decision Framework Recommendation 5-6: spine trauma or fracture and/or SCI**

**PICO 5: spine trauma or fracture and/or SCI managed non-operatively**

<https://guidelines.gradepro.org/profile/HaApoQ153kU>

**PICO 6: spine trauma or fracture and/or SCI managed operatively**

<https://guidelines.gradepro.org/profile/XexGjIWaWJU>

**Metanalysis for spine trauma or fracture and/or SCI managed non-operatively**

1. **Mortality**


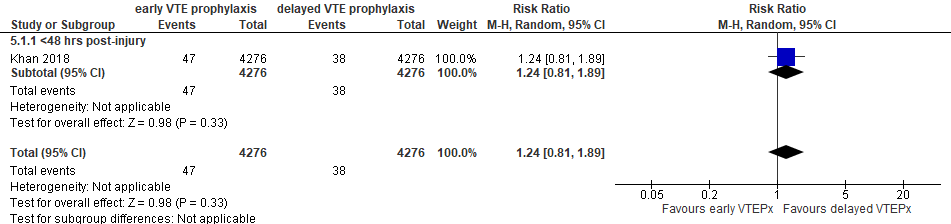


1. **VTE:**

**
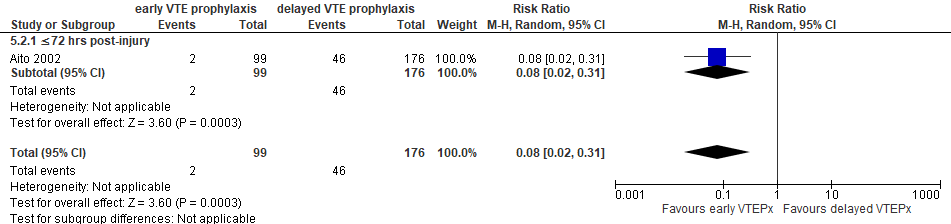
**

1. **DVT**


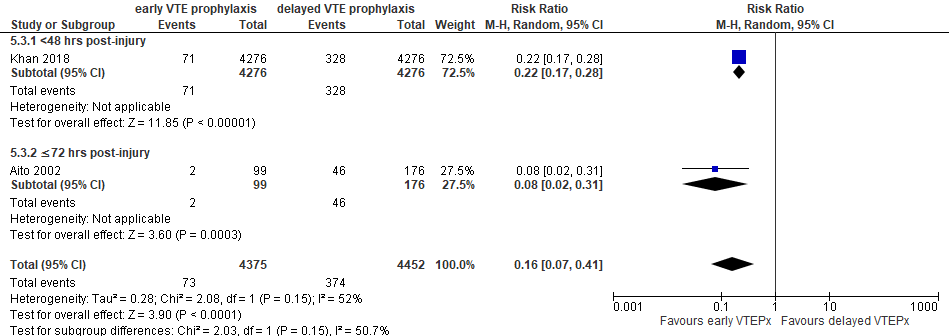


1. **PE:**


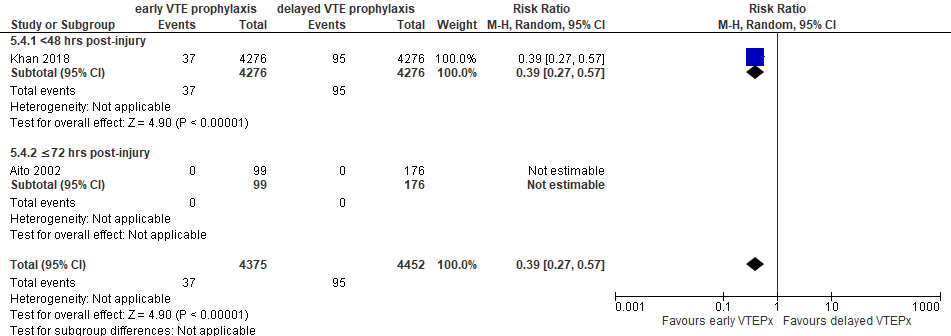


1. **Rate of/ need of Post VTE prophylaxis decompressive laminectomy**

(any intervention for decompression of the spinal canal after initiation of thromboprophylaxis): surrogate for intra-spinal hematoma


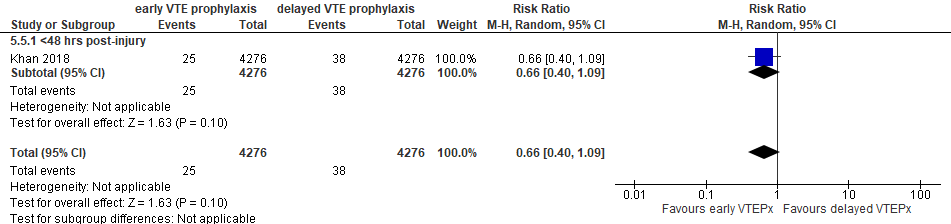


1. **Surrogate marker for any bleeding complications: Post-prophylaxis packed red blood cells (pRBCs) transfusion**


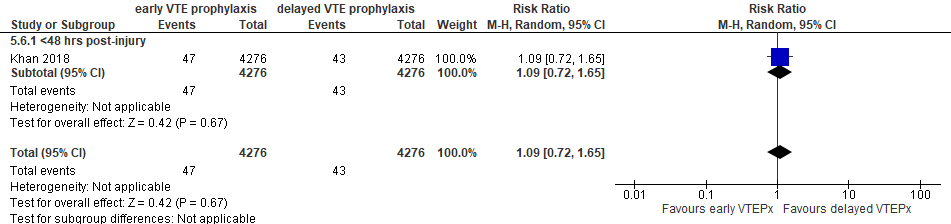


1. **Subgroup analysis for spine injury level for DVT from Khan 2018**


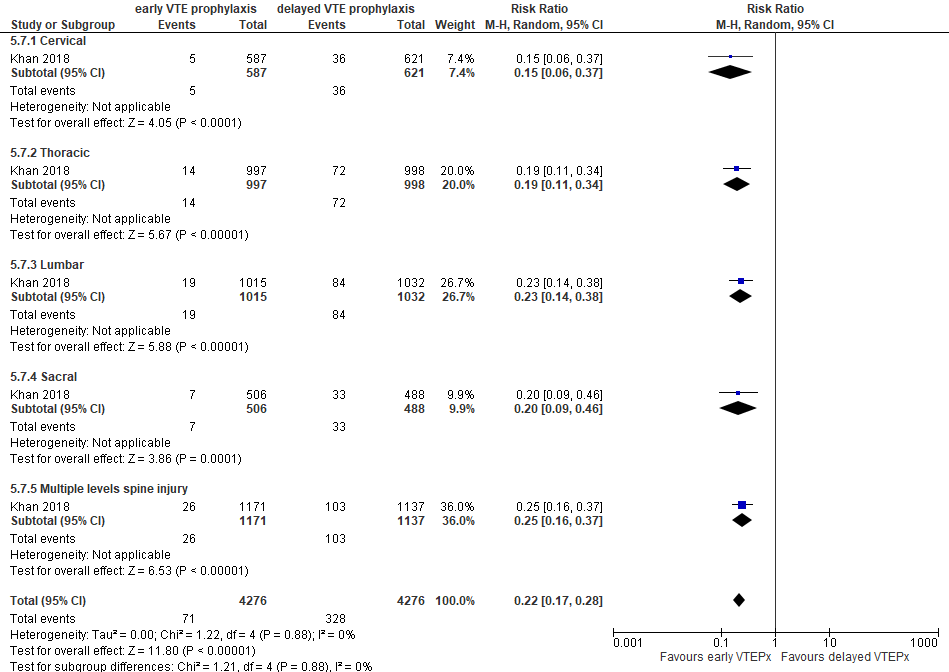


1. **Subgroup analysis for spine injury level for PE from Khan 2018**


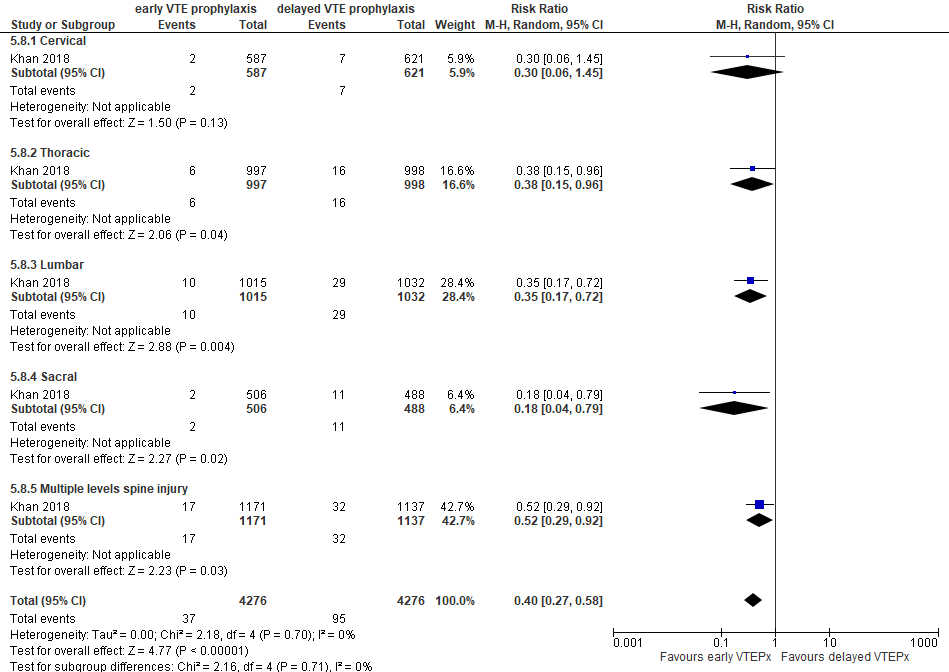


**Metanalysis for spine trauma or fracture and/or SCI managed operatively**

1. **Mortality**


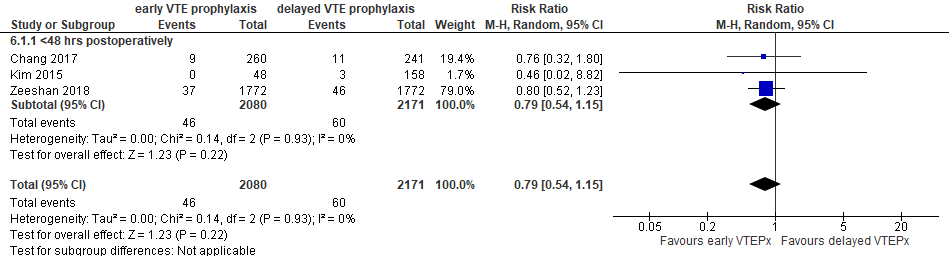


1. **VTE:**

**
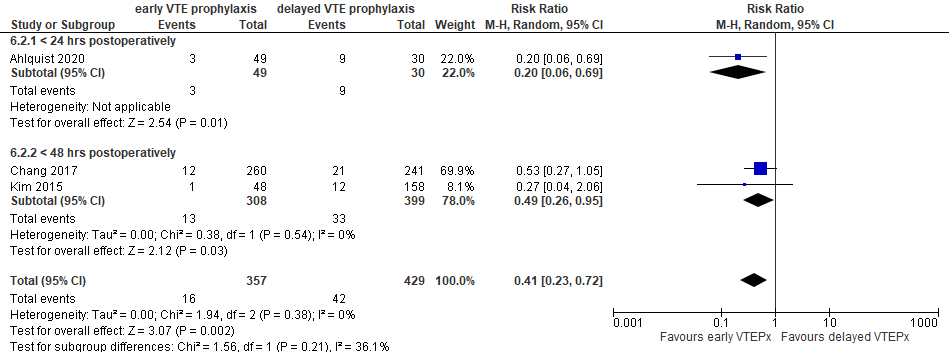
**

1. **DVT**


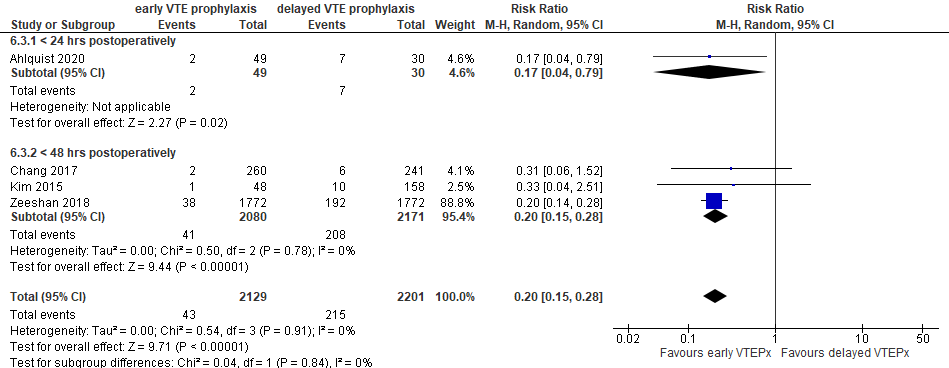


1. **PE:**


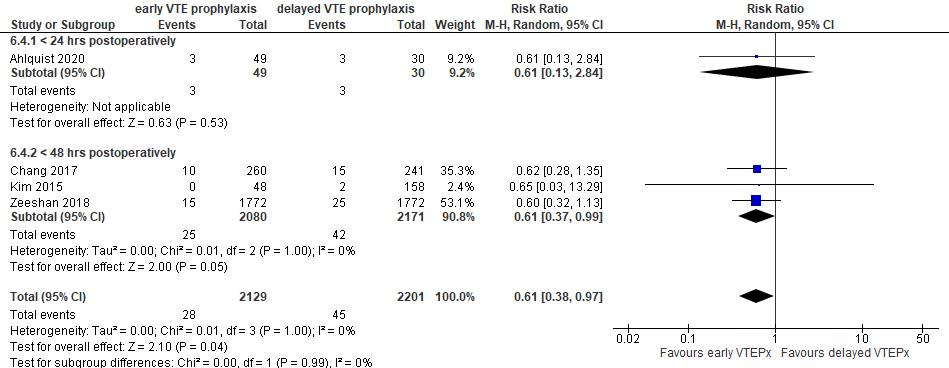


1. **Rate of/ need of Post VTE prophylaxis repeated decompressive laminectomy**

(any**repeated** intervention for decompression of the spinal canal after initiation of thromboprophylaxis): surrogate for intra-spinal hematoma)


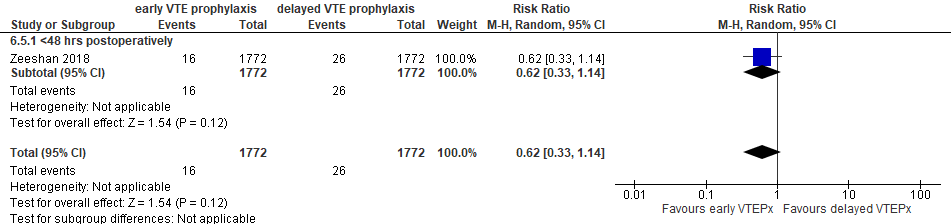


1. **Surrogate marker for any bleeding complications: Post-prophylaxis packed red blood cells (pRBCs) transfusion**


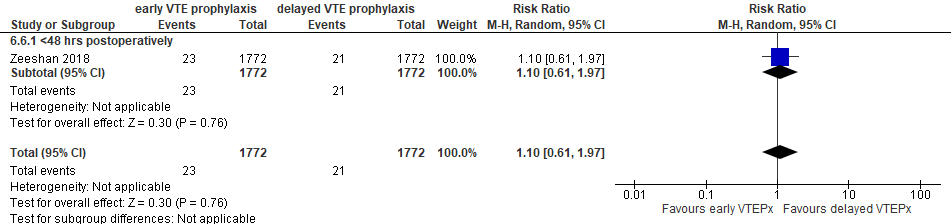


1. **Subgroup analysis for spine injury level for DVT from Zeeshan 2018**


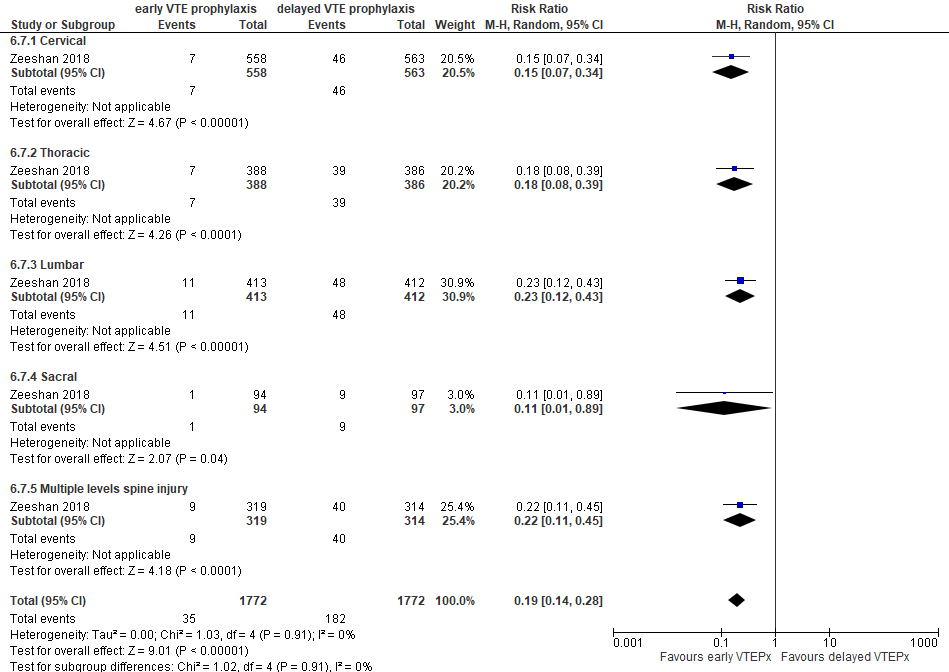


Please note numbers in subgroups were calculated based on percentages provided in table 4. The total number of DVT events = 35 vs 182, which is smaller than that in the primary analysis 38 vs 192. For some percentages we are not able to get the precise number of patients only approximate (Eg, sacral spine injury and multiple levels of spine injury.

1. **Subgroup analysis for spine injury level for PE from Zeeshan 2018**


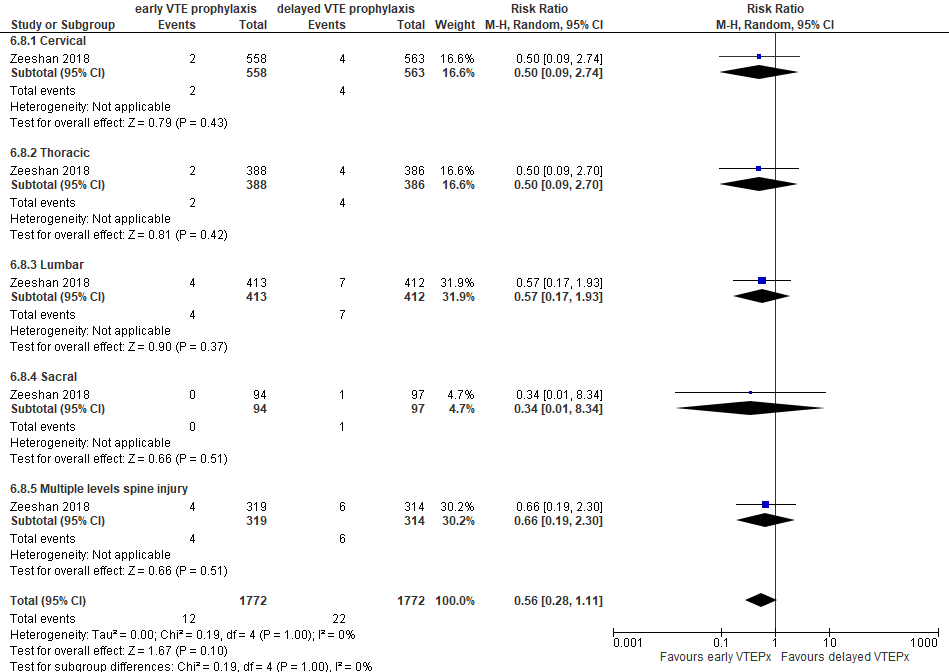


Please note numbers in subgroups were calculated based on percentages provided in table 4. The total number of PE events = 12 vs 22, which is smaller than that in the primary analysis 15 vs 25. For some percentages we are not able to get the precise number of patients only approximate (eg, thoracic spine injury, and lumbar spine injury.

**Table S8. PICO question: Recommendation 7: agent**

In adults with trauma who are prescribed VTE prophylaxis, should we recommend using low molecular weight heparin (LMWH) versus unfractionated heparin (UFH)?

| Population | Intervention | Comparator | Outcomes |
| --- | --- | --- | --- |
| Adults with  trauma in the ICU | LMWH | UFH | 1. VTE 2. Major bleeding |

- **Major bleeding is defined as** bleeding that results in hemodynamic instability, a hemoglobin drop of > 2 g/dL requiring transfusion of at least 2 units of whole blood or red cells, with temporal association within 12-24 h to the bleeding or bleeding in sensitive areas: brain, spine, or retroperitoneal bleeding; and need for surgical intervention.^[[4]](#footnote-4)^

**Evidence Profile and** **evidence to decision framework Recommendation 7: agent**

<https://guidelines.gradepro.org/profile/xphSP0xqeg4>

**Table S9. PICO question: Recommendation 8: Dose**

In adults with trauma who are prescribed LMWH, should we recommend using intermediate-high dose versus conventional dosing?

| Population | Intervention | Comparator | Outcomes |
| --- | --- | --- | --- |
| Adults with trauma who are prescribed LMWH | Intermediate-high dose LMWH | conventional dosing LMWH | 1. VTE 2. Major bleeding |

**Evidence Profile and** **evidence to decision framework Recommendation 8: Dose**

<https://guidelines.gradepro.org/profile/BWf_VYx4hqc>

**Meta-analysis for LMWH (enoxaparin) dose in trauma**

**VTE**


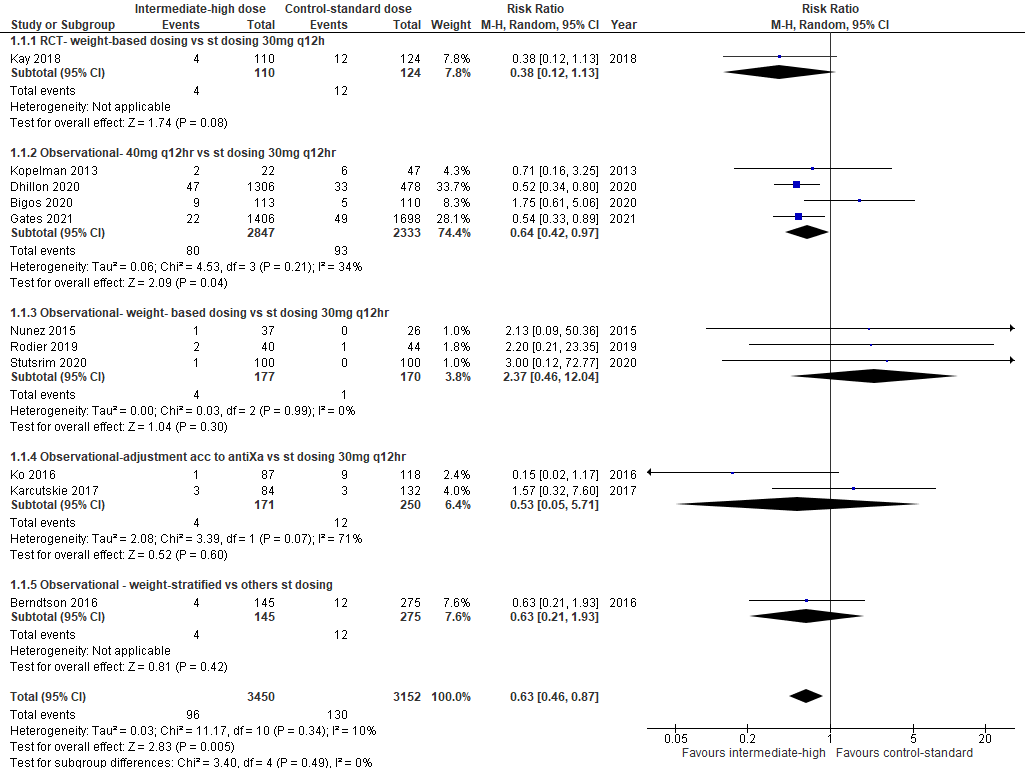


**DVT**


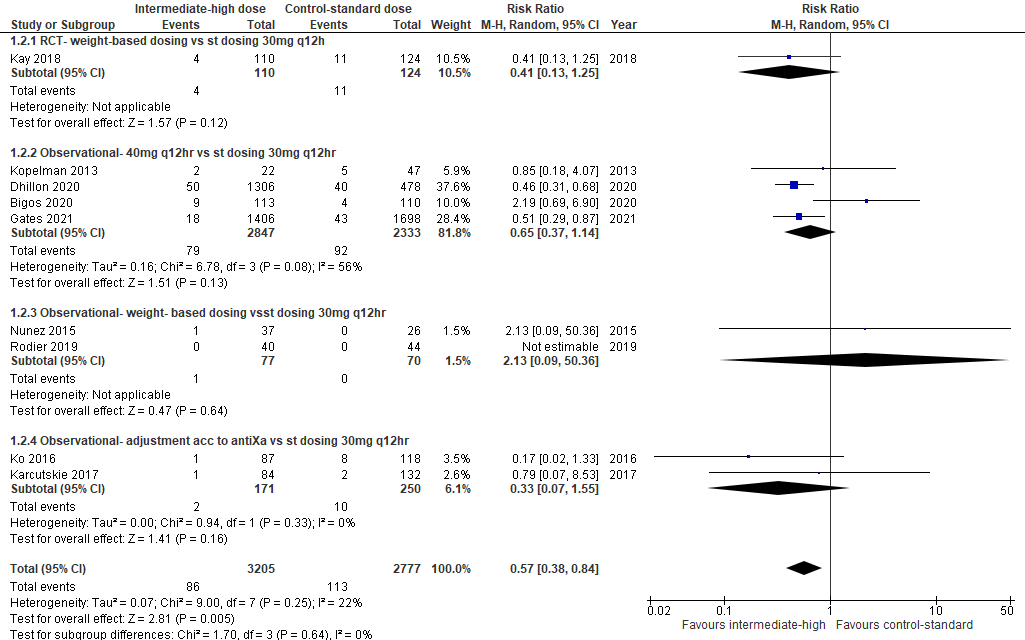


**PE**


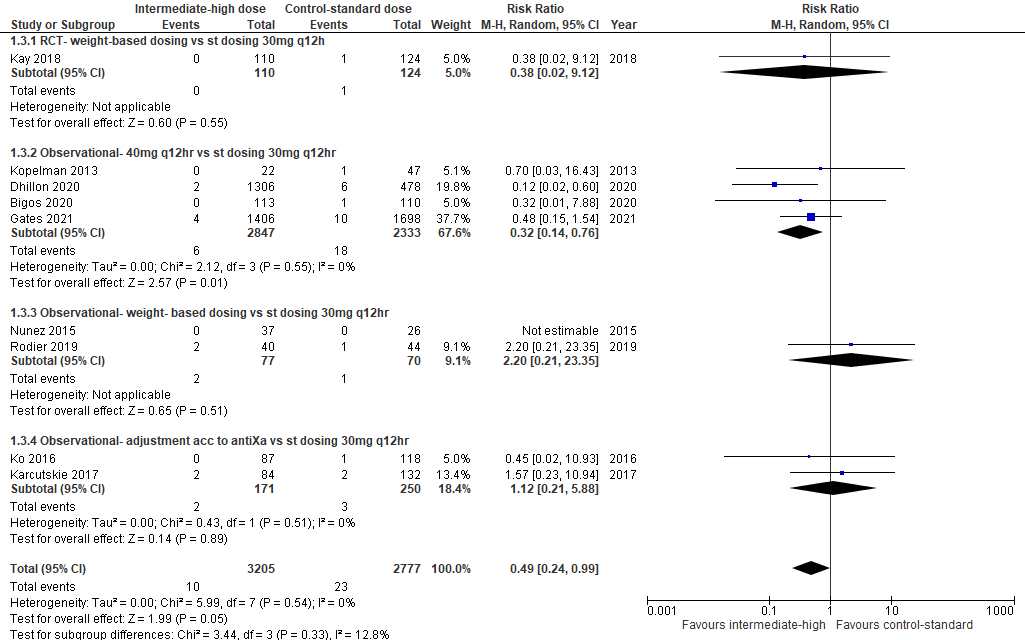


**Mortality**


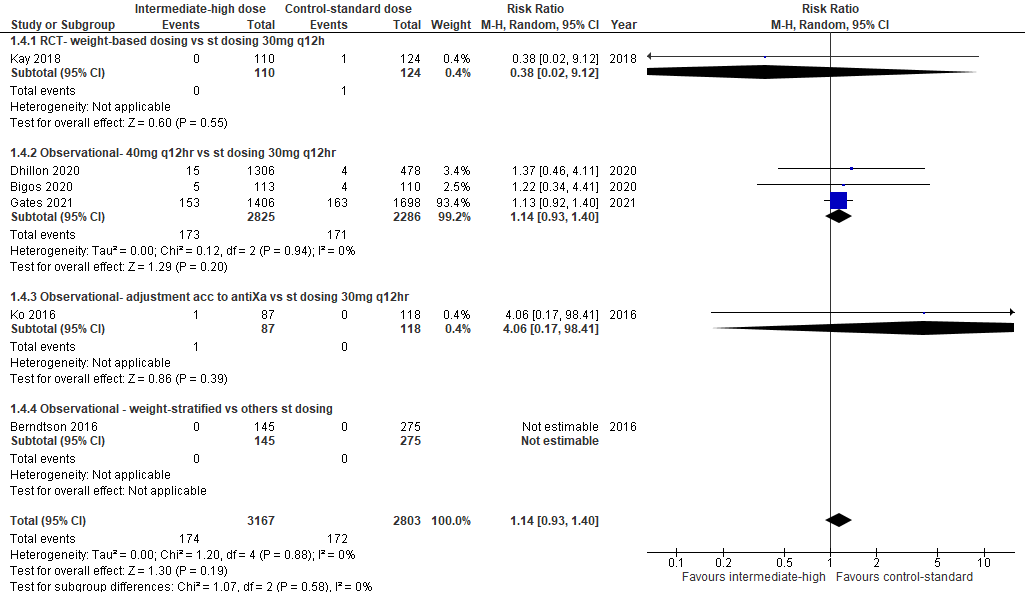


**On Target peak anti-Xa Level**


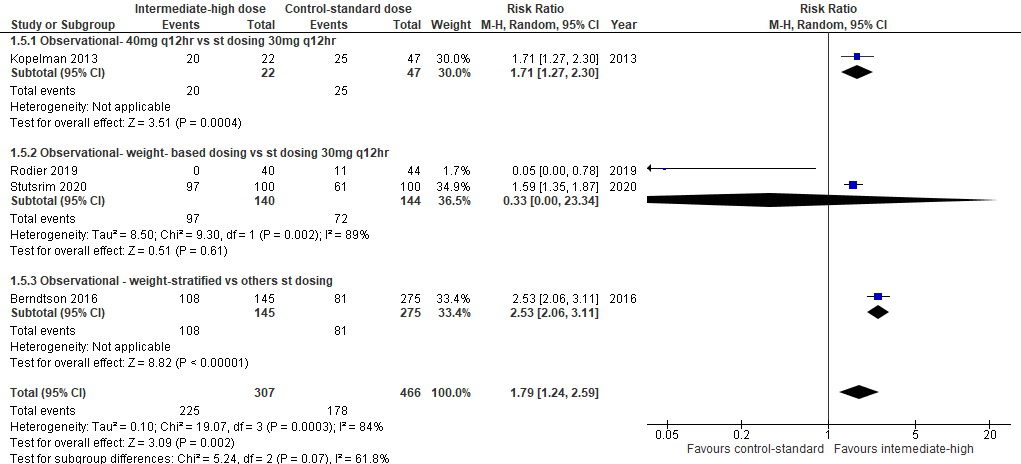


**On Target trough anti-Xa levels**


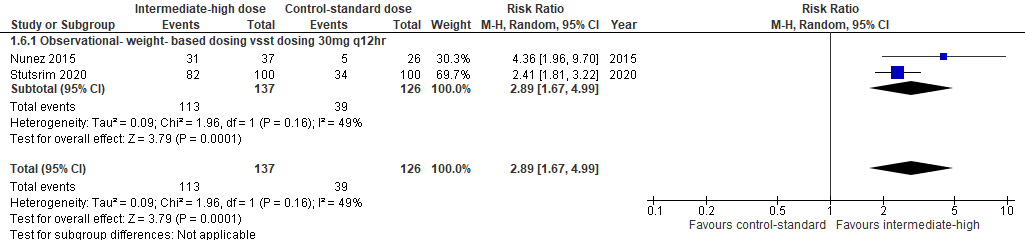


**Bleeding**


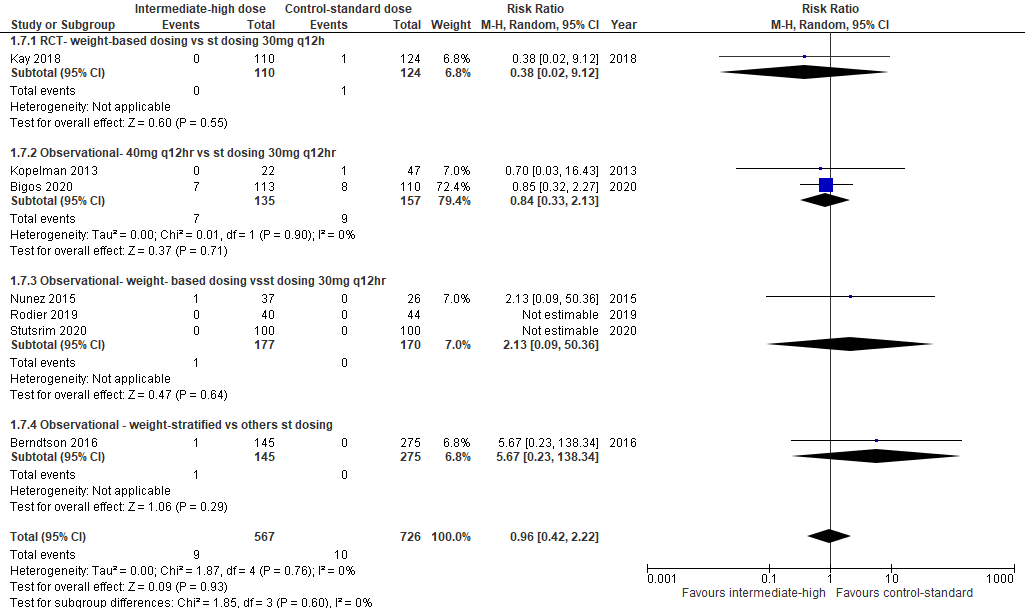


**RBC transfusion "surrogate"**

Pooled estimates for those studies showed significant heterogeneity preclude meta-analyzing them. We used Gates et al 20201 to base our decision in GDT evidence profile


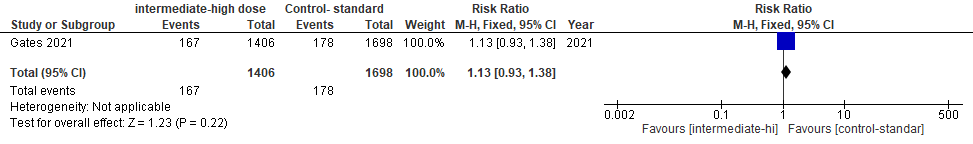


**Table S10. PICO question: Recommendation 9-10: Mechanical VTE prophylaxis**

**Recommendation 9:** In adults with trauma who are not candidates for pharmacologic VTE prophylaxis, should we recommend mechanical prophylaxis with intermittent pneumatic compression (IPC) versus no mechanical prophylaxis ?

| Population | Intervention | Comparator | Outcomes |
| --- | --- | --- | --- |
| Adults with trauma who are not candidate pharmacologic VTE prophylaxis | Mechanical VTE prophylaxis (IPC) | No mechanical prophylaxis | 1. VTE 2. Patient harm “ adverse events of mechanical prophylaxis e.g., skin breakdown, limb ischemia” |

**Recommendation 10:** In adults with trauma on pharmacologic prophylaxis, should we recommend adding mechanical VTE prophylaxis with intermittent pneumatic compression (IPC) versus pharmacologic prophylaxis alone?

| Population | Intervention | Comparator | Outcomes |
| --- | --- | --- | --- |
| Adults with trauma on pharmacologic VTE prophylaxis | Adding mechanical VTE prophylaxis (IPC) | Pharmacologic VTE prophylaxis alone | 1. VTE 2. Patient harm “adverse events of mechanical prophylaxis e.g., skin breakdown, limb ischemia” |

**Evidence Profile and Evidence to Decision Framework Recommendation 9-10: Mechanical VTE prophylaxis**

**PICO 9**

<https://guidelines.gradepro.org/profile/8om8BWmPK-s>

**PICO 10**

<https://guidelines.gradepro.org/profile/ICFXyvu8Og=>

**Meta-analyses for PICO 9: mechanical prophylaxis vs no prophylaxis**

1. **Mortality**
   1. **Mortality. Knudson 1994 Group I and Group III were combined.**

**
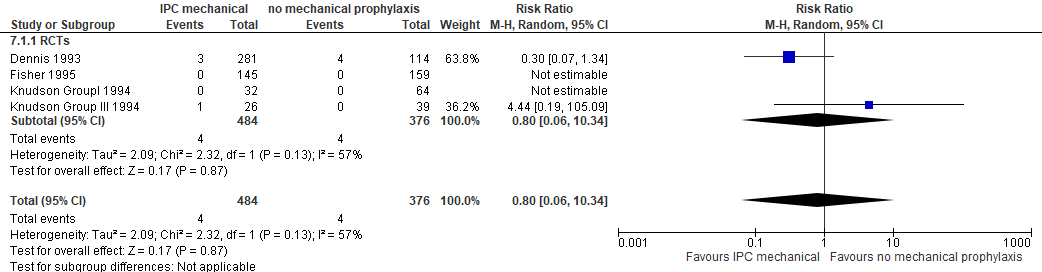
**

1. **VTE:**

**
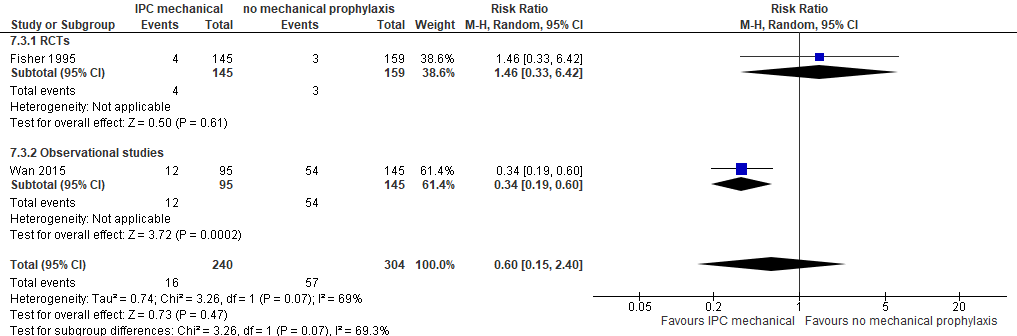
**

1. **DVT**
   1. **DVT. Knudson 1994 Group I and Group III were combined.**


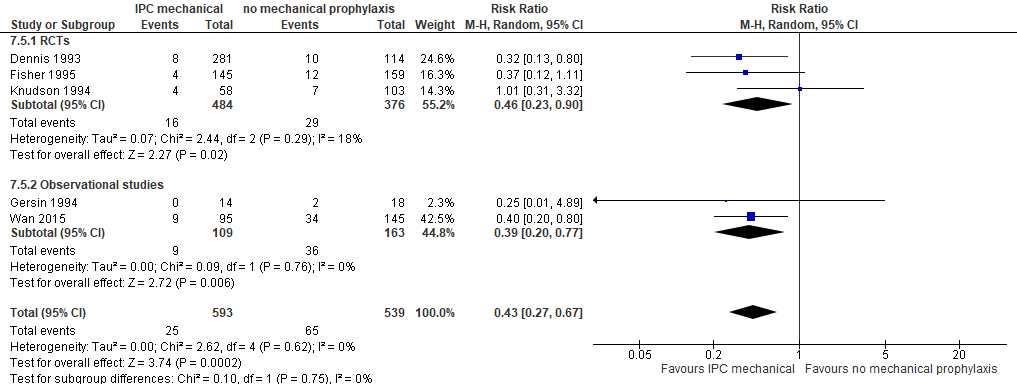


1. **PE:**
   1. **PE. Knudson 1994 Group I and Group III combined.**


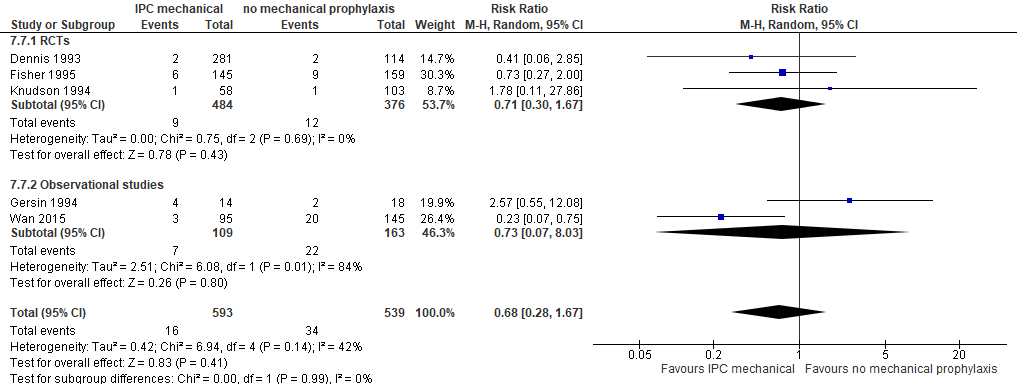


1. **Adverse events- Bleeding**
   1. **Adverse events- Bleeding. Knudson 1994 Group I and Group III were combined.**


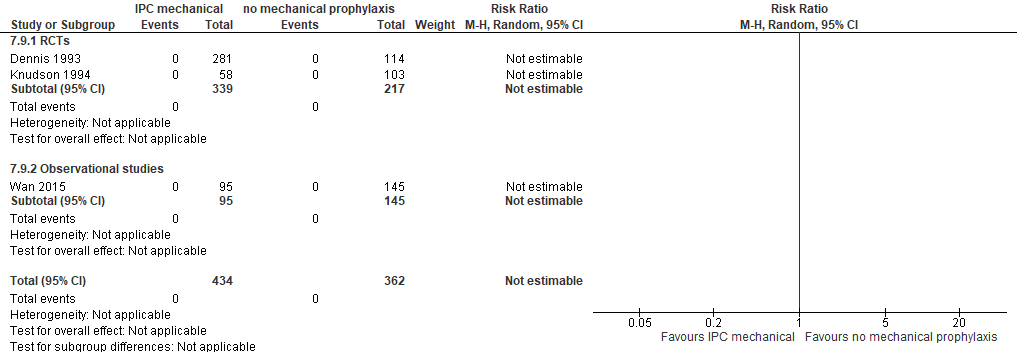


**Meta-analyses for PICO 10: adding mechanical VTE prophylaxis with intermittent pneumatic compression (IPC) compared to pharmacologic prophylaxis alone**

1. **Mortality**

Note: Arabi, data reported for 90 days and 28 days. As suggested, data at 28 days are included. In this trial, data regarding 28-day mortality were not available for two patients in the trial cohort who were discharged from the hospital before day 28 and were lost to follow‑up. There are two zero-event studies.


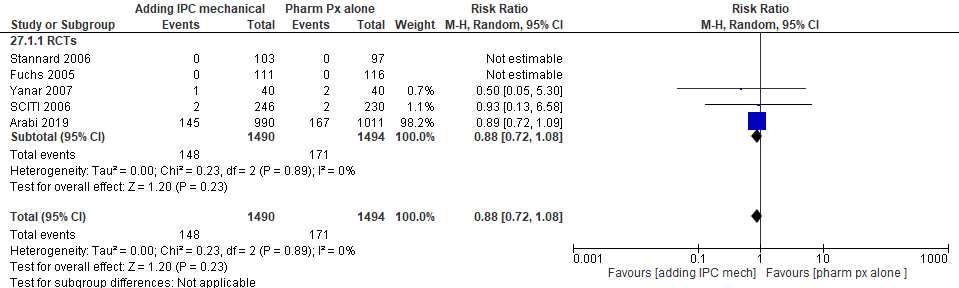


1. **VTE:**


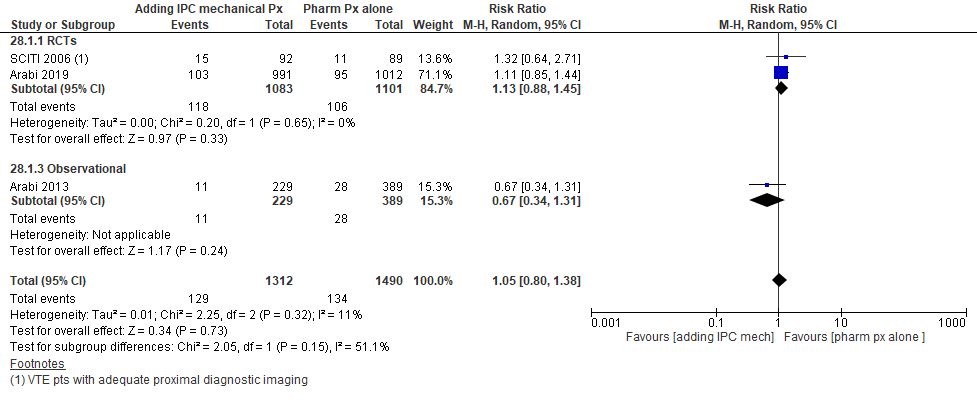


1. **DVT**


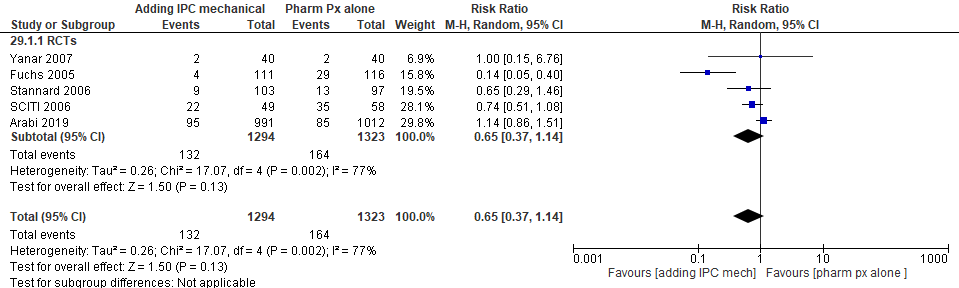


1. **PE:**


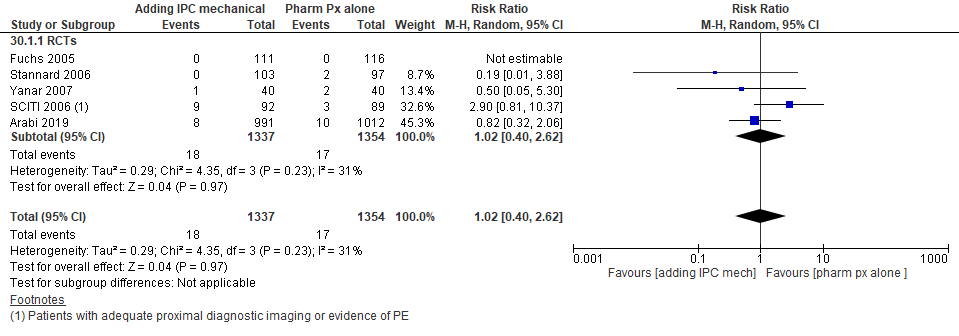


1. **Adverse events of mechanical prophylaxis: Leg skin injury**


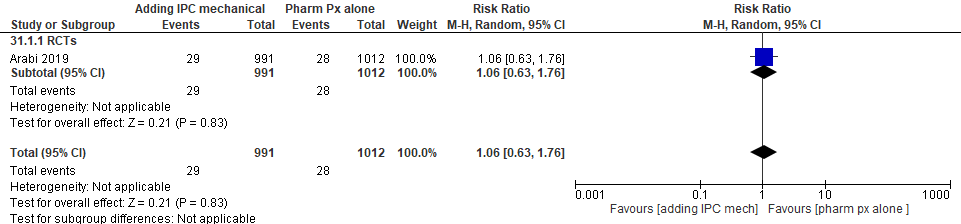


1. **Adverse events of mechanical prophylaxis: Limb ischemia**


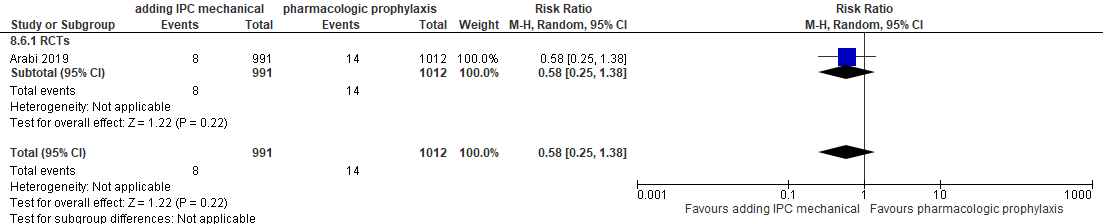


1. **Adverse events of mechanical prophylaxis: Bleeding**
   1. **Bleeding, SCITI 2003 used major bleeding.**


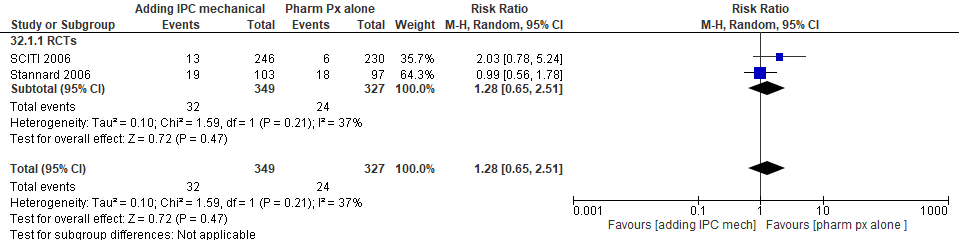


- 1. **Bleeding, SCITI 2003 used minor bleeding.**


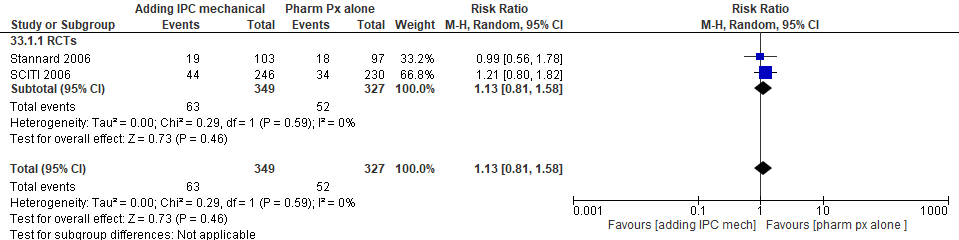


**Table S11. PICO question: Recommendation 11: ultrasonography (US) Surveillance**

In adults with trauma who are not candidates for pharmacologic VTE prophylaxis, should we recommend routine VTE US screening versus no routine screening?

| Population | Intervention | Comparator | Outcomes |
| --- | --- | --- | --- |
| Adults with trauma who are not candidate pharmacologic VTE prophylaxis | Routine surveillance duplex ultrasound | No routine surveillance | Lower limb DVT, or proximal LL DVT |

**Evidence Profile and Evidence to Decision Framework Recommendation 11: Surveillance ultrasound (US)**

<https://guidelines.gradepro.org/profile/b1Vj16rY8u0>

**Meta-analysis for Surveillance ultrasound (US)**

**Mortality: Critical outcome**

**RCT:**


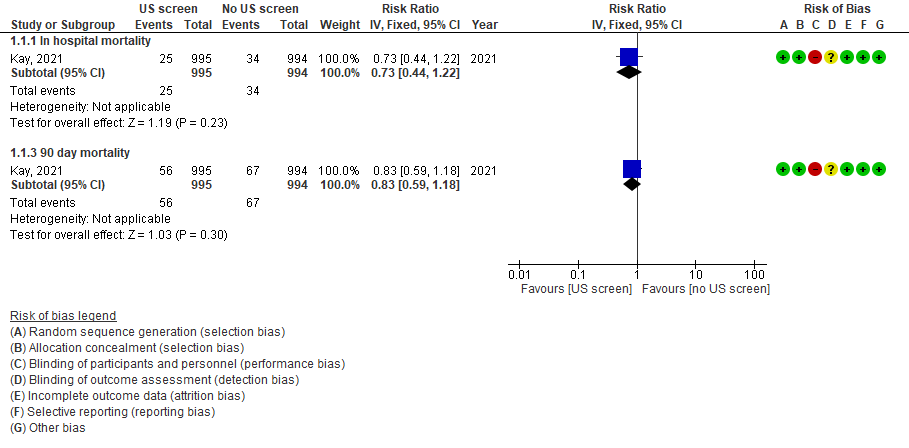


**Observational studies [conducted by IV fixed effect, presented OR]**


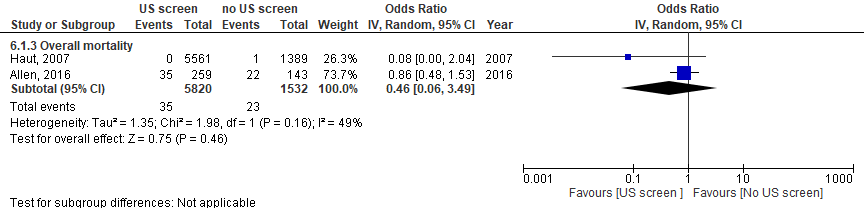


**Sensitivity analysis for low-moderate ROB studies ( excluding Haut 2007 mortality outcome)**


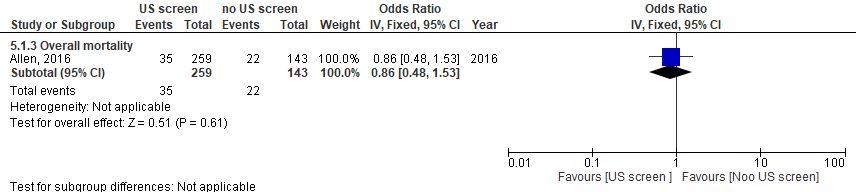


**DVT proximal and distal: Critical outcome**

**RCT:**


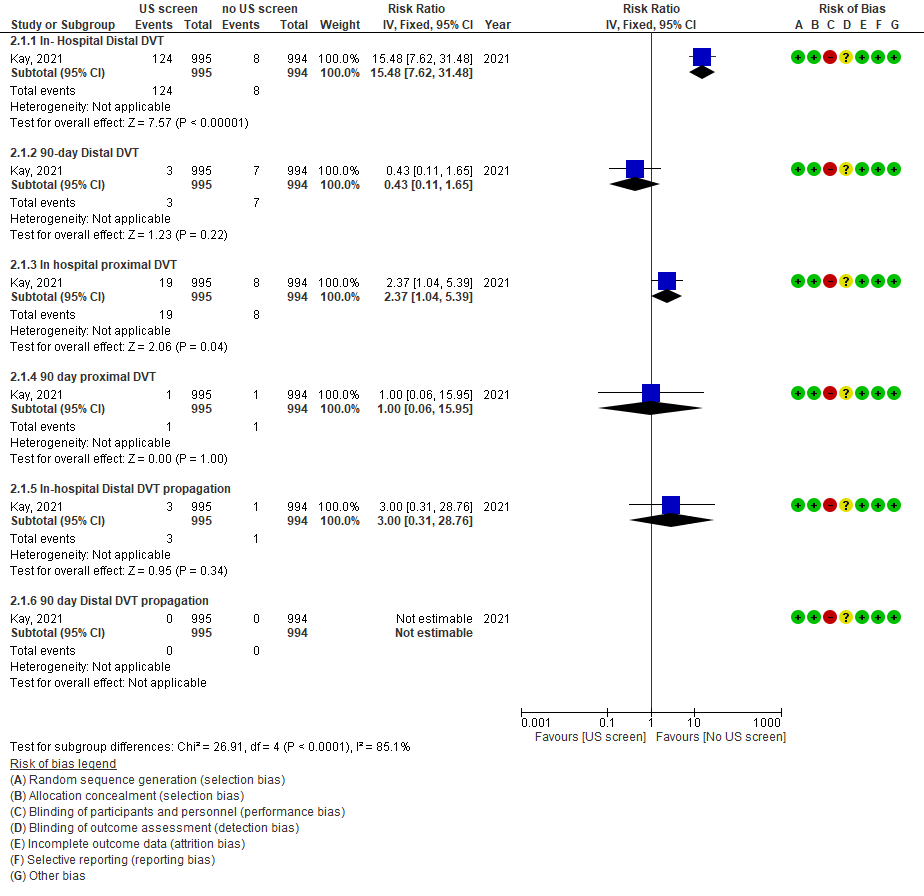


**DVT**

**Observational studies conducted by IV random effect, presented OR**

**
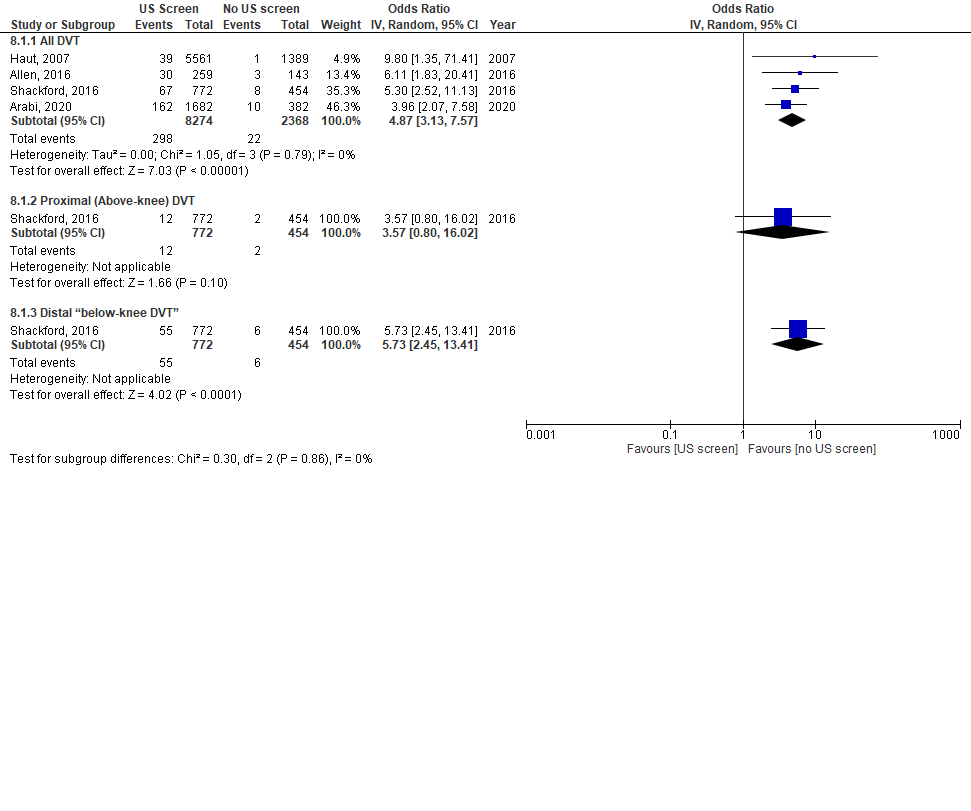
**

**Sensitivity analysis for low-mod ROB studies ( excluding Haut 2007 from all DVT outcome) showed similar results**

**
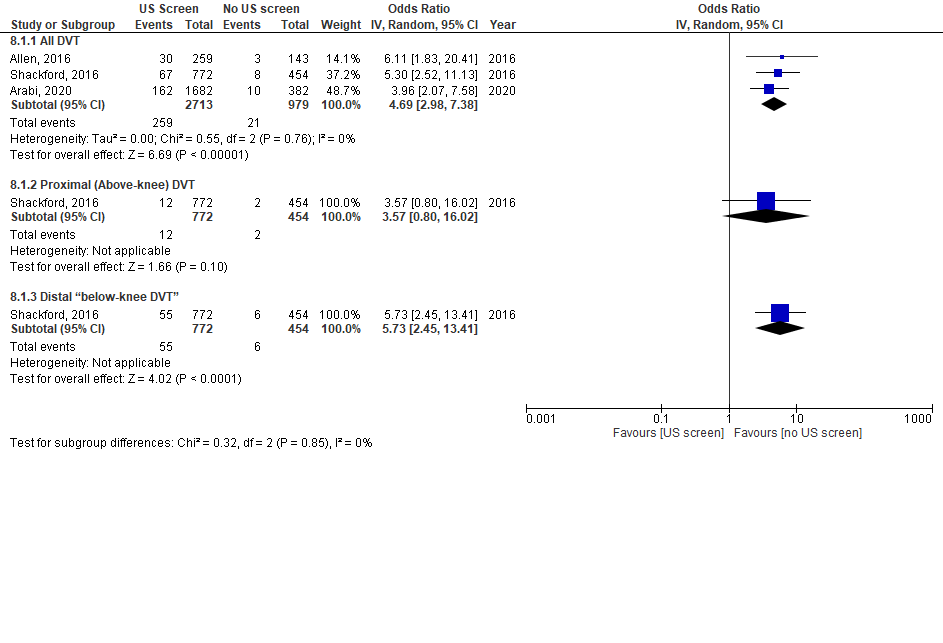
**

**PE: Critical outcome**

**RCT**

**
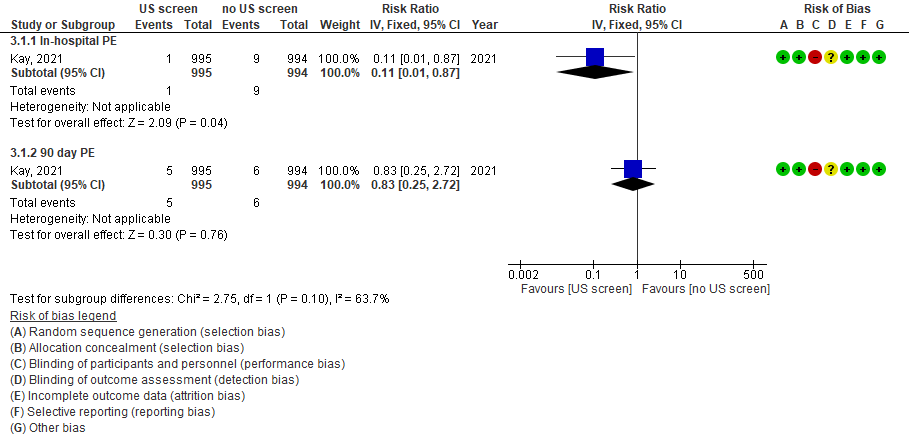
**

**Observational studies [conducted by IV random effect, presented OR]**

**
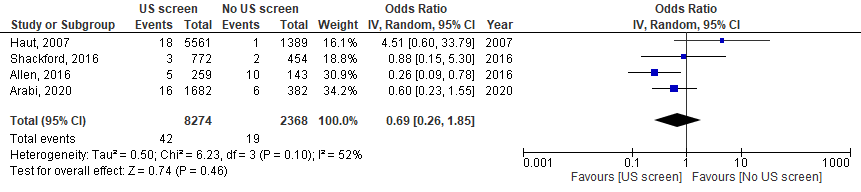
**

**Sensitivity analysis for low-moderate ROB studies ( excluding Haut 2007)**

**
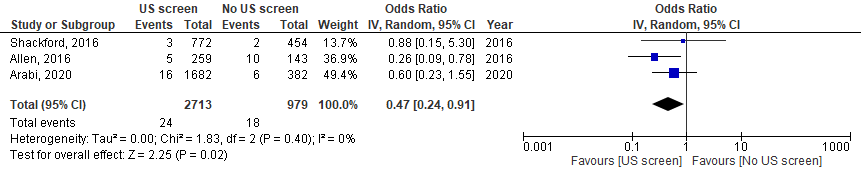
**

**Time to VTE Dx -RCT, Important outcome**

**
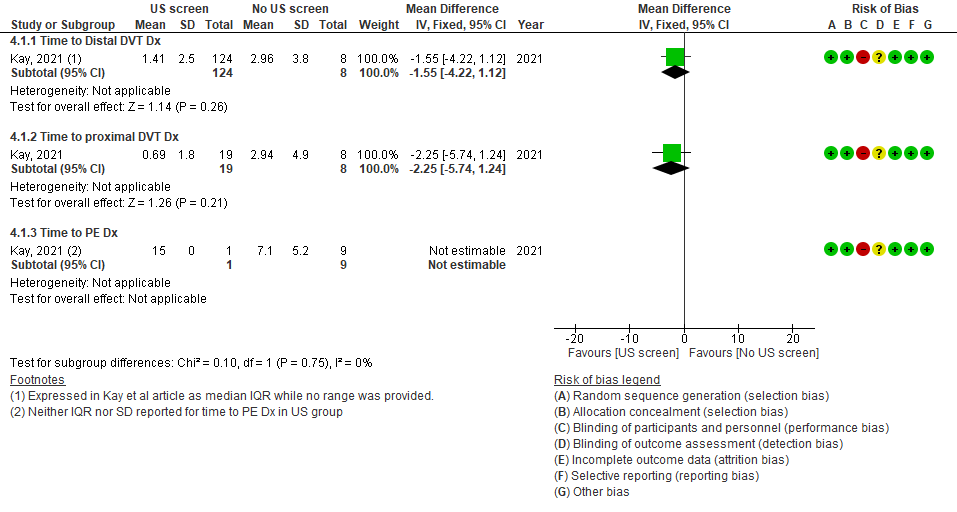
**

**Table S12. PICO question: Recommendation 12: Prophylactic IVC filter**

In adults with trauma who are not candidates for pharmacologic VTE prophylaxis, should we recommend prophylactic inferior vena-cava (IVC) filters versus no IVC filters?

| Population | Intervention | Comparator | Outcomes |
| --- | --- | --- | --- |
| Adults with trauma who are not candidate pharmacologic VTE prophylaxis | Prophylactic IVC filters | No prophylactic IVC filters | 1. VTE 2. Mortality 3. Filter insertion complications |

**Evidence Profile and Evidence to Decision Framework Recommendation 12: Prophylactic IVC filter**

<https://guidelines.gradepro.org/profile/BHbLqHtu0Gc>

**Meta-analysis for Prophylactic IVC filter**

**Mortality**

**RCT**


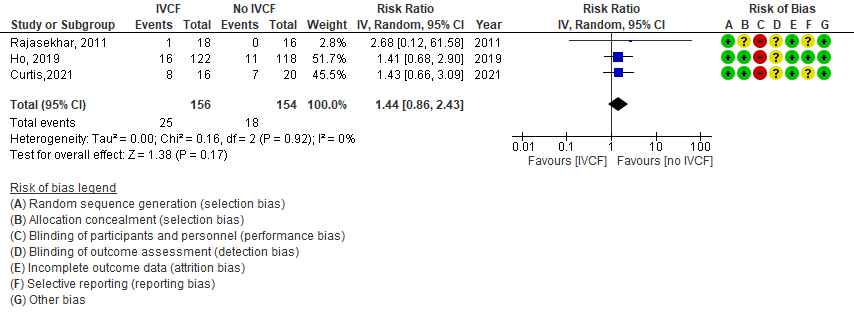


**Observational studies [conducted by IV random effect, presented RR]**


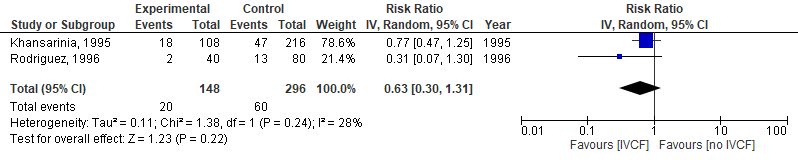


**PE**

**RCT**

Note RR here is slightly different than Ho KM et al reported RR. Ho KM et al used RR were calculated with the use of the Miettinen and Nurminen method and CI not adjusted for multiplicity.

**PE Observational studies [conducted by IV random effect, presented RR]**

**Fatal PE**

**RCT**

**Observational studies** No new study was added to 2014 SRMA. Batty et al was removed as it is unclear whether 2 fatal PEs reported in this study happened in the IVC group or the control group

**DVT**

**RCT**

**Observational studies [conducted by IV random effect, presented RR]**

**Table S13.** **Results for studies identified after conclusion of SRMAs and guidelines panel recommendations**

| **Guidelines domain** | **# of recent articles** |
| --- | --- |
| **PICO 1**: Timing of pharmacologic VTE prophylaxis in non-operative blunt solid organ injuries | 4 |
| **PICO 2,3**: Timing of pharmacologic VTE prophylaxis in blunt TBI | 6 |
| **PICO 5,6:** Timing of pharmacologic VTE prophylaxis in non-operative and operative spine trauma or fracture and/or SCI | 2 |
| **PICO 7:** Type of pharmacologic VTE prophylaxis (LMWH versus UFH) | 2 |
| **PICO 8:** Dose of LMWH (enoxaparin) in trauma | 5 |
| **PICO 10:** Adding mechanical VTE prophylaxis (IPC) versus pharmacologic VTE prophylaxis alone | 3 |
| **PICO 12:** Prophylactic placement of IVCF versus no IVCF | 2 |

**Table S14. Summary findings for studies identified after conclusion of SRMAs and guidelines panel recommendations**

| **Author/Year** | **Study Type and data source** | **Patients (N)** | **Study arms** | **Outcomes** | **Comments** |
| --- | --- | --- | --- | --- | --- |
| **PICO 1: Timing of pharmacologic VTE prophylaxis in non-operative blunt solid organ injuries** | | | | | |
| ***Gaitanidis, et al. (2021)^[[5]](#footnote-5)^*** | retrospective analysis of the Trauma Quality Improvement Program database was performed between 2013 and 2016 | 3,22 isolated Blunt Solid organ injuries (liver, spleen, pancreas, or kidney, Abbreviated Injury Scale score, <3 in other regions) who underwent  initial nonoperative management | Divided into three groups (early, <48 hours; intermediate,  48–72 hours; and late, >72 hours) | **Late initiation** was independently associated with a higher likelihood of both DVT (OR, 3.15; 95% CI, 1.68–5.91, p < 0.001) and PE (OR, 4.29; 95% CI, 1.95–9.42; p < 0.001).  **Intermediate initiation** was independently associated with a higher likelihood of DVT (OR, 2.38; 95% CI, 1.20–4.74; p = 0.013), but not PE  (p = 0.960) compared with early initiation.  **Early initiation** was associated with a higher likelihood of bleeding (OR, 2.05; 95% CI, 1.11–2.18; p = 0.023), along with a history of diabetes mellitus, splenic, and high-grade liver injuries. | Early pharmacological VTE prophylaxis should be considered in patients with ***low likelihood of bleeding.***  ***An intermediate delay (48–72 hours) should be considered for patients with diabetes, splenic injuries, and Grades 3 to 5 liver injuries***  Detailed information regarding  Dosing of thromboprophylaxis is also not available in the TQIP database |
| ***Moore, et al. (2022)^[[6]](#footnote-6)^*** | single-center, retrospective study between January 2010 and October 2017 | 207 **high-grade** blunt liver and splenic lacerations (AAST grades ≥3) who survived 24 h post-admission and did not require massive  transfusion | early, defined as ≤72 h  after presentation; vs. late, >72 h after presentation | Early administration of LMWH was not associated with a statistically  significant increased risk of failure of non-operative management (p = 0.054). Rates of VTE and in-hospital mortality were similar. | Distribution of grades 3, 4, and 5 liver and splenic injuries were similar across all groups.  55.5% received LMWH  Underpowered to detect a difference among groups due to small sample size |
| ***Anteby R et al (2023)^[[7]](#footnote-7)^*** | systematic review and meta-analysis | blunt abdominal solid organ injuries N= 21,909 (6375 included on adjusted outcomes)  12 observational studies | Late (>48 h) versus early (<48 h) thromboprophylaxis | No difference in failure of nonoperative management (OR 0.92, 95% CI:0.4-2.14).  In adjusted analysis for VTE events, patients receiving late thromboprophylaxis had a higher risk of VTE compared with those who received early (OR 1.89, 95% CI:1.15-3.12) | Used adjusted analysis |
| ***Lamb T et al (2022)^[[8]](#footnote-8)^*** | systematic review and meta-analysis | 4642 patients  10 cohort studies | Late (>48 h) versus early (<48 h) thromboprophylaxis | significant increase in the risk of failure of nonoperative management in early group (OR 1.76, 95% CI 1.01–3.05, p = 0.05). There was no significant difference in risk of transfusion. Odds of DVT were significantly lower in the  early group (OR 0.36, 95% CI 0.22–0.59, p < 0.0001). There was no difference in mortality (OR 1.50, 95% CI 0.82–2.75, p = 0.19) | Used un-adjusted analysis |
| **PICO 2,3: Timing of pharmacologic VTE prophylaxis in blunt TBI** | | | | | |
| ***Al‑Dorzi, et al. (2022)^[[9]](#footnote-9)^*** | Retrospective cohort study at King Abdulaziz  Medical City, Riyadh, Saudi Arabia between February 1, 2016, and December 31, 2019. | 322 Moderate TBI GCS of 9–12 and severe TBI GCS of <9 | early (≤72 h from hospital admission) versus late (>72 h)  pharmacologic prophylaxis. | VTE occurred in 6.6% of the early prophylaxis group and 26.6% of the late group (P < 0.001). On multivariable logistic regression analysis, the predictors of VTE were APACHE II score, subarachnoid hemorrhage, and late versus early pharmacologic prophylaxis (OR, 3.858; 95% CI, 1.687–8.825). | UFH was more commonly used than LMWH  Single institution experience |
| ***Jakob, et al. (2021)^[[10]](#footnote-10)^*** | Matched cohort study using ACS-TQIP database (2013-2016) | Severe blunt head trauma with acute subdural hematomas (ASDH).  severe TBI was defined as head Abbreviated Injury Scale ≥3  1660 matched cases | early prophylaxis (≤48 h) and late prophylaxis (>48 h) | VTE complications (3.1% vs 0.5%, p < 0.001) were more common in the late prophylaxis  compared to the early group.  **Multivariable regression analysis**: early prophylaxis is protective  factor for VTE complications (OR 0.169, p < 0.001) but not mortality (p = 0.260) or delayed craniectomy risk (p = 0.095).  LMWH was independently associated with a lower mortality (OR 0.480, p = 0.008) compared to UH. | There is a possibility of overlapping cohort with Byrne et al. 2016 (included in our SRMA): used ACS-TQIP database for severe TBI from January 1, 2012 to December 31, 2014  TBI characteristics was deemed to be low risk of bleeding progression (only 8% had a GCS<8, excluded patients on pre-injury anticoagulants). Size of ASDH was not reported |
| ***Jakob, et al. (2021)^[[11]](#footnote-11)^*** | Matched cohort study using ACS-TQIP database (2013–2017) | blunt head trauma with combined ASDH and subarachnoid  hemorrhage (SAH)  2152 matched cases | early prophylaxis (≤48 h) and late prophylaxis (>48 h) | **Multivariable regression analysis**: early prophylaxis is protective  factor for VTE complications (OR 0.468, CI 0.293–0.748) but not mortality (p = 0.485) or delayed craniectomy risk (p = 0.283). | Possibility of overlapping cohort with Byrne et al. 2016 (included in our SRMA): used ACS-TQIP database for severe TBI from January 1, 2012 -December 31, 2014  ASDH and SAH sizes were not reported |
| ***Shulkosky, et al. (2022) ^[[12]](#footnote-12)^*** | cohort study using Michigan Trauma Quality  Improvement Program (MTQIP) from 2008 to 2018. | TBI with head Abbreviated Injury Scale ≥3, n= 5,589 | <24 hour group vs. 24-48 hour group vs. 48-72 hour group vs. >72 hour group | VTE were significantly greater in patients initiated within 48-72 hours (adjusted OR 2.861, 95% CI 1.271-6.439) and >72 hours (aOR 3.963, 95% CI 1.824-8.612) compared to <24 hours.  Patients that received VTE prophylaxis within 24 hours had similar  rates of serious in-hospital complication as patients initiated within 24-48 hours (aOR .956, 95% CI .637-1.434) and 48- 72 hour (aOR 1.132, 95% CI .757-1.692) but less than the >72 hour group (aOR 1.662, 95% CI 1.154-2.393) | MTQIP includes TBI patients from 32 American College of Surgeons (ACS) verified level 1 or 2 trauma centers. Notably, all patients enrolled in this program are also enrolled in the ACS-TQIP. There is a possibility of patients overlap with Jakob’s and Byrne’s studies  Included penetrating and blunt TBI |
| ***Störmann, et al. (2022)^[[13]](#footnote-13)^*** | Retrospective review of a single center in Germany between January 2015 and December 2018. | Severe TBI with head abbreviated injury scale ≥3 and at least one further AIS ≥ 3 in any other body region. N=79 | Early (≤24 h) chemoprophylaxis (n = 35) vs. late (>24 h)chemoprophylaxis (n = 29) vs. no therapy (n = 15). | Intracranial hemorrhage progression after VTEp was reported in 6 patients in the early group (N=29) and 9 patients in the late group (N=35), with no statistically significant difference (adjusted P = 0.8; adjusted OR, 1.23; 95% CI, 0.24-6.21).  The VTE rate was low; there was only 1 VTE event in the early group and none in the late group (P=0.53).  In-hospital mortality did not demonstrate statistically significant difference between the early and late group (2.9% vs. 10.3%; adjusted p = 0.24; adjusted OR, 0.06; 95% CI, 0-5.9). | Unpowered, and single-center experience. High risk of selection bias due to lack of standardized protocol.  Included multiple trauma combined with severe TBI (not isolated TBI). Patients are severely injured with a mean ISS of 36.7 ± 12.7, which is markedly higher than existing TBI literature. Significant differences exist in baseline characteristics including age, GCS,and cerebral herniation  No routine US surveillance (raises concerns for missed DVTs).  ICP monitoring and craniotomies are unknown whether performed before or after VTEp. |
| ***Darwin Ang, et al (2022)^[[14]](#footnote-14)^*** | retrospective cohort study of 87 trauma centers in USA | 7977 moderate to severe TBI / 23,548 | **control group:**  no prophylaxis. Other groups received LMWH, UFH, and combined LMWH and UFH chemoprophylaxis. | patients with subdural  hematoma and LMWH between 6 and 24 h (N = 62), as well as patients with ‡35 BMI and LMWH between 6 and 24 h (N = 65) or >24–48 h (N = 54), had no VTE events. | VTE chemoprophylaxis timing may have prevented VTE in certain subgroups of isolated TBI patients.  LMWH VTE chemoprophylaxis was associated with reduced mortality |
| **PICO 5,6: Timing of pharmacologic VTE prophylaxis in spine trauma or fracture and/or SCI** | | | | | |
| ***Taghlabi, et al (2022)^[[15]](#footnote-15)^*** | retrospective review between May 2015 and June 2019. | Patients with spinal fracture operative intervention N= 88 | <72h versus late >72h chemoprophylaxis  timing | Chemoprophylactic anticoagulation at 72 hours in surgically treated spinal fracture patients demonstrates a lower VTE rate (4.4% vs. 30%) without increasing bleeding complications  Timing of anticoagulation were not significant in binary logistic regression | small sample size and underpowered  Between groups  significant difference in number of transfers (p = 0.018), surgical service (p = 0.008), GCS (p = 0.03) |
| ***Godat LN, et al (2022)^[[16]](#footnote-16)^*** | secondary analysis of the 2018-2020 prospective, observational, cohort CLOTT study | 343 patients with SCI | Early Pharmacologic VTE prophylaxis ≤ 48 h vs late >48 h | After adjusting for differences in risk factors between cohorts starting ≤48 hours was independently associated with fewer VTEs (OR 0.45, CI 0.101-0.978, p = 0.044). | _ |
| **PICO 7: Type of pharmacologic VTE prophylaxis (LMWH versus UFH)** | | | | | |
| ***Maragkos, et al (2022)^[[17]](#footnote-17)^*** | retrospective review of National Trauma Data Bank of the American College of Surgeons (ACS) for 2017 and 2018 | TBI patients with head  Abbreviated Injury Score (AIS) <6.  61,998 matched cases / 218,594 | LMWH vs. UFH | logistic regression showed lower rates of  post-prophylaxis cranial decompression for the LMWH group (OR, 0.13; 95% CI, 0.11-0.16; P < 0.001) | This finding questions the notion of UH being safer for patients  with TBI because it can be readily reversed. RCTs are needed to elucidate causality. |
| ***Danford, et al (2022)^[[18]](#footnote-18)^*** | retrospective cohort study of Trauma Data Bank (NTDB) using data from the year 2013 | operatively treated closed femoral shaft fracture treated with open reduction and internal fixation N= 2,058 | LMWH vs. UFH | LMWH had lower odds of inpatient mortality compared to patients who received VTE prophylaxis with UFH (OR 0.19; 95% CI 0.05 to 0.68, p = 0.011). | dosing of VTE prophylaxis is not reported |
| **PICO 8: Dose of LMWH (enoxaparin) in trauma** | | | | | |
| ***Bellfi, et al (2022)^[[19]](#footnote-19)^*** | Retrospective study from January 2020 to June 2021 at a Level I trauma center | N=204 trauma patients | standard dosing 30 mg twice daily vs. increased dose 40 mg twice daily | Sub-therapeutic anti-Xa levels were higher with standard dosing compared to the increased dose (44 vs 22%, P = 0.003).  VTE rates were ***non-significant*** with standard dosing compared to higher dosing (6.2 vs 3.3%, P= 0.52) and incidence of major bleed is similar (1.8 vs 4.4%, P= 0.41) | Anti-Xa levels is surrogate endpoint (not patient-centered outcomes)  No adjustments of confounders were made |
| ***Verhoeff K. et al (2022) ^[[20]](#footnote-20)^*** | systematic review and meta-analysis | N= 4302  24 observational studies | Standard fixed dosing vs. anti-Xa guided dosing, | dose adjustment, including anti-Xa guided dosing, may **not reduce VTE**.  Anti-Xa guided dosing achieves prophylactic anti-Xa more frequently (OR, 4.05; p = 0.007) but without VTE (OR, 0.72; p = 0.15) or  PE (OR, 0.48; p = 0.10) differences. | Primary outcome attainment of anti-Xa level (surrogate outcome) |
| ***Ebeid, A et al (2022) ^[[21]](#footnote-21)^*** | systematic review and meta-analysis | N= 764  4 observational studies | Wight based dosing vs. standard dosing | Weight-based enoxaparin prophylaxis dosing was associated with increased odds of prophylactic AFXa levels (odds ratio, 5.85; 95% CI, 3.02–11.30; p < 0.00001). There was **no statistical difference in incidence VTE** between the two groups (RD, 0.01; 95% CI, −0.02 to 0.03; p = 0.64). | Did not include the RCT by Kay et al 2018 which compared wight based dosing vs. standard dosing |
| ***Grange L et al (2022)^[[22]](#footnote-22)^*** | systematic review and meta-analysis | 9 studies | adjustment of LMWH thromboprophylaxis dose (according to body weight or changes in anti-factor Xa level) versus no adjustment | **no significant reduction in the risk of VTE** was observed with adjusted doses of LMWH compared with fixed doses when considering only randomised control trials (OR 1.02 [95% CI, 0.09 to 11.6]) or all trials (OR 0.70 [95% CI, 0.34 to 1.42]). There was no significant difference in bleeding risk (OR 1.36, 95% CI 0.59 to 3.10). | no evidence to justify adjusting LMWH doses |
| ***Tran A et al (2022)^[[23]](#footnote-23)^*** | systematic review and meta-analysis | N= 10,348  15 observational studies | fixed LMWH dosing vs. anti-Xa guided dosing | anti-Xa guided dosing may reduce  DVT (aOR 0.52, 95% CI 0.40 to 0.69), PE (aOR 0.48, 95% CI 0.30 to 0.78) or any VTE (aOR  0.54, 95% CI 0.42 to 0.69), uncertain effect on mortality (aOR 1.06, 95% CI 0.85 to 1.32) and bleeding events (aOR 0.84, 95% CI 0.50 to 1.39) | limited by clinical heterogeneity with regards to anti-Xa targets, comparison regimen, anti-Xa lab assays, timing of peak and trough concentrations  Findings are inconsistent with previous SRMAs that showed no significant difference in VTE risk with anti-Xa guided dosing |
| **PICO 10: Adding mechanical VTE prophylaxis (IPC) versus pharmacologic VTE prophylaxis alone** | | | | | |
| ***Kakkos, et al (2022)^[[24]](#footnote-24)^*** | Updated systematic review and meta-analysis | 34 studies involving 14,931 participants, participants undergoing surgery and trauma and ICU patients | Combined IPC and pharmacological VTE prophylaxis compared to single one | IPC and pharma VTE prophylaxis vs. pharma VTE prophylaxis alone reduced symptomatic PE (OR 0.46, 95% CI 0.3 to 0.71; 15 studies, N=6737, low-certainty evidence).  incidence of DVT reduced in favor of the combined group (OR 0.38, 95% CI 0.21 to 0.70; 17 studies, 6151 participants, high-certainty evidence)  Increased risk of major bleeding in combined group (OR 1.21, 95% CI 0.35 to 4.18; 5 studies, 908 participants, very low-certainty evidence). | included heterogonous population [14 studies orthopaedic surgery, 3 studies urology, 2 studies cardiothoracic, 1 study neurosurgery, 1 study trauma, 12 studies general surgery and gynaecology]  Risk for performance bias was high in most studies, and risk for selection and detection bias was mostly unclear or high. |
| ***Guo, et al (2022)^[[25]](#footnote-25)^*** | Retrospective single-center study from January 2016 to February 2020. | Patients with mild craniocerebral injury and clavicular fractures; N=252 (126 patients on each arm). | Combined IPC and pharmacological VTE prophylaxis compared to pharmacological VTE prophylaxis alone | The incidence of DVT was significantly lower in the combined IPC and pharmacological VTE prophylaxis than pharmacological VTE alone (5.6% [7/126] vs. 15.1% [19/126], respectively; P<0.05).  IPC (OR, 5.833; 95% CI 1.807 to 18.828). | Small sample size and single-center experience.  Reporting of the multivariable logistic regression is questionable. The text description seems to contradict the results presented in the table in relation to the pneumatic compression. |
| ***Duval C, et al (2022)^[[26]](#footnote-26)^*** | systematic review and meta-analysis of RCTs | 17 trials enrolling 8,796 participants | adjunctive IPC and pharmacological VTE prophylaxis compared to pharmacological VTE prophylaxis alone | Adjunctive IPC was associated with a decreased risk of VTE (15 trials, RR = 0.53; 95% CI [0.35–0.81]) and DVT (14 trials, RR = 0.52; 95% CI [0.33–0.81]) but not PE (seven trials, RR = 0.73; 95% CI [0.32–1.68]).  Adjunctive IPC is unlikely to change the risk of all-cause mortality or adverse events | VTE events were rare, generating wide confidence Limits. low-quality evidence underpinning the additional use of IPC to pharmacological thromboprophylaxis |
| **PICO 12: Prophylactic placement of IVCF versus no IVCF** | | | | | |
| ***Ho, et al (2022)^[[27]](#footnote-27)^*** | Priori sub-study of da Vinci RCT; their median follow-up time after enrollment was 65 months (interquartile range 59–73). | Severely injured patients with mix trauma (N=198 patients who survived 90 days follow-up). | IVCF group (N=99) versus control group (N=99) | 10 patients (5.1%) died after day-90 follow-up, and none were related to VTE.  4 patients developed VTE, all allocated to the control group (0 vs 4%, p = 0.043). 2 of them were symptomatic PE at 10 and 62 months after insertion. 1 developed despite being on pharmacologic VTEpx and 1 developed secondary to readmission for femur fracture.  Symptomatic PE since enrollment with follow-up until March 4, 2022 (N = 223) was significantly lower in the IVCF group (1 [0.5%] vs. 8 [7.3%]; p =0.014). Patients allocated to the filter group experienced a significantly reduced risk of developing symptomatic PE (Log-rank test: p = 0.018).  Symptomatic PE from enrollment to March 4, 2022 for those who could not be anti- coagulated within 7 days of injury, was significantly higher among the control group (N = 72: 0/41 for IVCF group vs. 6/31 for the control group; p = 0.005).  Inferior vena cava injuries/complications were not recorded in any patients after 90-days. | This is a sub-study of da Vinci trial, which is included in our meta-analysis (Ho et al, 2019); however, this study reported long-term outcomes beyond 90-day follow-up of the original trial report.  It is possible that some patients could have long-term complications attributable to the use of the filters managed completely in a private healthcare setting and not recorded in this study. |
| ***Lee, et al (2022)^[[28]](#footnote-28)^*** | Retrospective of national trauma data bank from 2008 to 2015. | Patients with severe traumatic pelvic/lower extremity, intracranial, and spinal cord injuries (N=462,838). | 11,938 (2.6%) patients received a prophylactic IVCF versus 450,900 (97.4%) who did not. | Prophylactic IVCF showed higher incidence of PE (aOR, 5.25; 95% CI, 4.31-6.39; P <.001) and DVT (aOR, 5.55; 95% CI, 4.96-6.20; P <.001) compared to control group.  All-cause in-hospital mortality was lower among patients with prophylactic IVCF (aOR, 0.46; 95% CI, 0.42-0.50; P <.001) compared to control group. | Retrospective nature is a major limitation.  Serious risk of classification bias.  Unknown timing of VTE is a major limitation leading to potential misclassification, in which VTE could occurred before IVCF placement (therapeutic IVCF). This could raise a serious potential for overestimation of VTE events in the IVCF arm. |

1. Stassen NA, et al; Eastern Association for the Surgery of Trauma. Nonoperative management of blunt hepatic injury: an Eastern Association for the Surgery of Trauma practice management guideline. J Trauma Acute Care Surg. 2012 Nov;73(5 Suppl 4):S288-93. doi: 10.1097/TA.0b013e318270160d. **PMID: 23114483.** [↑](#footnote-ref-1)
2. Stassen NA, et al; Eastern Association for the Surgery of Trauma. Selective nonoperative management of blunt splenic injury: an Eastern Association for the Surgery of Trauma practice management guideline. J Trauma Acute Care Surg. 2012 Nov;73(5 Suppl 4):S294-300. doi: 10.1097/TA.0b013e3182702afc. **PMID: 23114484** [↑](#footnote-ref-2)
3. Coccolini F, et al; WSES-AAST Expert Panel. Kidney and uro-trauma: WSES-AAST guidelines. World J Emerg Surg. 2019 Dec 2;14:54. doi: 10.1186/s13017-019-0274-x. **PMID: 31827593** [↑](#footnote-ref-3)
4. Schulman S, et al. Definition of major bleeding in clinical investigations of antihemostatic medicinal products in non-surgical patients. J Thromb Haemost. 2010 Jan;8(1):202-4. **PMID: 15842354.** [↑](#footnote-ref-4)
5. Gaitanidis, et al. Timing of thromboprophylaxis in patients with blunt abdominal solid organ injuries undergoing nonoperative management. The Journal of Trauma and Acute Care Surgery; 2021-01-01 [↑](#footnote-ref-5)
6. Moore K, Barton CA, Wang Y, et al. Early initiation of thromboembolic prophylaxis in critically ill trauma patients with high-grade blunt liver and splenic lacerations is not associated with increased rates of failure of non-operative management. Trauma. February 2022. doi:10.1177/14604086211046099 [↑](#footnote-ref-6)
7. Anteby R, Allar BG, Broekhuis JM, et al. Thromboprophylaxis Timing After Blunt Solid Organ Injury: A Systematic Review and Meta-analysis. J Surg Res. 2023 [↑](#footnote-ref-7)
8. Lamb T, Lenet T, Zahrai A, et al. Timing of pharmacologic venous thromboembolism prophylaxis initiation for trauma patients with nonoperatively managed blunt abdominal solid organ injury: a systematic review and meta-analysis. World J Emerg Surg. 2022 Apr 25;17(1):19 [↑](#footnote-ref-8)
9. Dorzi HM, Al-Yami G, Al-Daker F, Alqirnas MQ, Alhamadh MS, Khan R. The association of timing of pharmacological prophylaxis and venous thromboembolism in patients with moderate-to-severe traumatic brain injury: A retrospective cohort study. Ann Thorac Med 2022;17:102-9. [↑](#footnote-ref-9)
10. Jakob DA, et al. Venous thromboembolic pharmacological prophylaxis in severe traumatic acute subdural hematomas: Early prophylaxis is effective and safe. Am J Surg. 2022 May;223(5):1004-1009. [↑](#footnote-ref-10)
11. Jakob DA, et al. Timing of venous thromboembolic pharmacological prophylaxis in traumatic combined subdural and subarachnoid hemorrhage. Am J Surg. 2022 Jun;223(6):1194-1199. [↑](#footnote-ref-11)
12. Shulkosky MM, Han EJ, Wahl WL, Hecht JP. Effects of Early Chemoprophylaxis in Traumatic Brain Injury and Risk of Venous Thromboembolism. Am Surg. 2022 May 15:31348221102604 [↑](#footnote-ref-12)
13. Störmann P, et al. Early start of thromboprophylaxis does not increase risk of intracranial hematoma progression in multiply injured patients with traumatic brain injury. Brain Injury. 2022 Aug 3. Ahead of print. DOI: 10.1080/02699052.2022.2105951 [↑](#footnote-ref-13)
14. Darwin Ang, Kevin Pierre, John Armstrong. Timing and Type of Venous Thromboembolic Chemoprophylaxis Is Associated with Acute Traumatic Brain Injury Outcomes. Neurotrauma Reports 2022 3:1, 511-521 [↑](#footnote-ref-14)
15. Taghlabi et al, Chemoprophylactic Anticoagulation 72 Hours After Spinal Fracture Surgical Treatment Decreases Venous Thromboembolic Events Without Increasing Surgical Complications, North American Spine Society Journal (NASSJ) (2022) [↑](#footnote-ref-15)
16. Godat LN, Haut ER, Moore EE, et al. venous thromboembolism risk after spinal cord injury: a secondary analysis of the CLOTT study. J Trauma Acute Care Surg. 2022 Oct 7. [↑](#footnote-ref-16)
17. Maragkos GA, et al. Delayed Cranial Decompression Rates After Initiation of Unfractionated Heparin versus Low-Molecular-Weight Heparin in Traumatic Brain Injury. World Neurosurg. 2022 Jun 9:S1878-8750(22)00791-4. [↑](#footnote-ref-17)
18. Danford NC, et al. Venous thromboembolism prophylaxis with low molecular weight heparin versus unfractionated heparin for patients undergoing operative treatment of closed femoral shaft fractures, Journal of Clinical Orthopaedics and Trauma (2022). [↑](#footnote-ref-18)
19. Bellfi LT, Zimmerman SA, Boudreau R, et al. Impact of Increased Enoxaparin Dosing on Anti-Xa Levels for Venous Thromboembolism Prophylaxis in Trauma Patients. The American Surgeon. July 2022. [↑](#footnote-ref-19)
20. Verhoeff K, Raffael K, Connell M, Kung JY, Strickland M, Parker A, Anantha RV. Relationship between anti-Xa level achieved with prophylactic low-molecular weight heparin and venous thromboembolism in trauma patients: A systematic review and meta-analysis. J Trauma Acute Care Surg. 2022 Aug 1;93(2):e61-e70. [↑](#footnote-ref-20)
21. Ebeid A, Cole E, Stallwood-Hall C. The efficacy of weight-based enoxaparin dosing for venous thromboembolism prophylaxis in trauma patients: A systematic review and meta-analysis. J Trauma Acute Care Surg. 2022 Aug 1;93(2):e71-e79. [↑](#footnote-ref-21)
22. Grange L, Chapelle C, Ollier E, et al. Adjusted versus fixed doses of LMWHs in trauma patients: A systematic review and meta-analysis. Anaesth Crit Care Pain Med. 2022 Dec;41(6):101155. [↑](#footnote-ref-22)
23. Tran A, Fernando SM, Gates RS, et al. Efficacy and Safety of Anti-Xa Guided versus Fixed Dosing of Low Molecular Weight Heparin for Prevention of Venous Thromboembolism in Trauma Patients - A Systematic Review and Meta-Analysis. Ann Surg. 2022 [↑](#footnote-ref-23)
24. Kakkos S, Kirkilesis G, Caprini JA, et al. Combined intermittent pneumatic leg compression and pharmacological prophylaxis for prevention of venous thromboembolism. Cochrane Database Syst Rev. 2022 Jan 28;1(1):CD005258. [↑](#footnote-ref-24)
25. Guo PC, Li N, Zhong HM, Zhao GF. Clinical effectiveness of a pneumatic compression device combined with low-molecular-weight heparin for the prevention of deep vein thrombosis in trauma patients: A single-center retrospective cohort study. World Journal of Emergency Medicine. 2022;13(3):189. [↑](#footnote-ref-25)
26. Duval C, Sirois C, Savoie-White FH, et al. Effect of Intermittent Pneumatic Compression in Addition to Pharmacologic Prophylaxis for Thromboprophylaxis in Hospitalized Adult Patients: A Systematic Review and Meta-Analysis. Crit Care Explor. 2022 Oct 3;4(10):e0769. [↑](#footnote-ref-26)
27. Ho KM, Patel P, Chamberlain J, Nasim S, Rogers FB. Long-term outcomes after using retrievable vena cava filters in major trauma patients with contraindications to prophylactic anticoagulation. European Journal of Trauma and Emergency Surgery. 2022 Aug 27:1-7. [↑](#footnote-ref-27)
28. Lee SJ, Fan S, Guo M, Majdalany BS, Newsome J, Duszak Jr R, Gichoya J, Benjamin ER, Kokabi N. Prophylactic IVC filter placement in patients with severe intracranial, spinal cord, and orthopedic injuries at high thromboembolic event risk: A utilization and outcomes analysis of the National Trauma Data Bank. Clinical Imaging. 2022 Aug 5. [↑](#footnote-ref-28)
