# Supplementary material for: Saudi Critical Care Society clinical practice guidelines on the prevention of venous thromboembolism in adults with trauma: reviewed for evidence-based integrity and endorsed by the Scandinavian Society of Anaesthesiology and Intensive Care Medicine
Source: Ann Intensive Care. 2023 May 11;13:41. doi: 10.1186/s13613-023-01135-8 (PMC10172441; doi:10.1186/s13613-023-01135-8)
Supplement: Supplementary file 3 — Additional file 3: Appendix 3. Management of conflict of interests [file 13613_2023_1135_MOESM3_ESM.docx]

### **Management of conflict of interests (COI)**

Guidelines chair (MA) and co-chairs (WA, MSA) have no identified COI related to the topic of the proposed guidelines. All panel members completed a COI form prior to participation (see table below). The panel members were also asked to disclose relevant COI before voting on recommendations and at the start of manuscript writing.

To ensure the integrity of the guidelines, guidelines chair (MA) and co-chairs (WA, MSA) reviewed all disclosures and adjudicated any potential conflicts prior to assigning panel members to different subgroups according to guidelines questions. These included financial, intellectual, and personal COI. Direct financial and industry-related COI were not permitted. We defined intellectual COI as leading clinical research that is directly relevant to a given recommendation/topic. Panel members with possible intellectual COI were not permitted to vote on corresponding recommendations. All reported/adjudicated COIs were secondary and were managed in accordance with the Saudi Critical Care Society COI policy and guiding principles for disclosure of COI (1-5).

**Pertinent COI and resolution of COI:** YA is a principal investigator for US Screening trial (DETECT-NCT05112705), PREVENT trial (NCT02040103) and PREVENT sub-study. This was considered as intellectual COI and resolved by abstaining from voting on a matter related to the conflict.

The remaining panel members declare that they have no competing interests. All panelists without COI have the opportunity to provide input on the final recommendations. We defined consensus as ≥ 80% agreement rate and ≥ 75% response rate. Voters could provide feedback for consideration in revising statements that did not receive consensus in up to three rounds of voting. However, we achieved approval and agreement on all recommendations after a single round of voting.

| **Panel Member** | **CoI link** |
| --- | --- |
| Faisal Al-Suwaidan | <https://gdt.gradepro.org/forms/#who-coi-preview/p_wzk505_40dca86f-6cb6-47bb-9233-73763ee4982a_7b780f12-134b-4b43-ac31-7f1d13793421_79eba7dc-5718-489d-868a-c3baaf8a28a0/sections> |
| Ali Alaklabi | <https://gdt.gradepro.org/forms/#who-coi-preview/p_wzk505_40dca86f-6cb6-47bb-9233-73763ee4982a_844acf86-0895-4afe-9f92-f6ff67b2838a_951f729e-63af-41f4-a14d-1832fca24adc/sections> |
| Muneerah Albugami | <https://gdt.gradepro.org/forms/#who-coi-preview/p_wzk505_40dca86f-6cb6-47bb-9233-73763ee4982a_d5c9ea79-a9de-4136-9546-4c0c6e529b0d_21f20e5e-a7d2-47f5-bbe6-691ebed40d5a/sections> |
| Ahmed Aljedai | <https://gdt.gradepro.org/forms/#who-coi-preview/p_wzk505_40dca86f-6cb6-47bb-9233-73763ee4982a_2c30f0d2-c722-473c-a0db-707b7a57987c_cbedb776-3496-4819-863e-da931d9cc0a9/sections> |
| Hosam Aljehani | <https://gdt.gradepro.org/forms/#who-coi-preview/p_wzk505_40dca86f-6cb6-47bb-9233-73763ee4982a_727e4ea9-27d9-4acf-bdc6-da877e2bd4f5_facc0449-8e97-4806-b65a-09cce0d1fe95/sections> |
| Maha Aljuaid | <https://gdt.gradepro.org/forms/#who-coi-preview/p_wzk505_40dca86f-6cb6-47bb-9233-73763ee4982a_a6282bf2-4d31-43c8-8d92-0a60023ce862_93fcc38f-1076-4b62-8dea-b84c4bf7bb93/sections> |
| Mohammad Alsenani | <https://gdt.gradepro.org/forms/#who-coi-preview/p_wzk505_40dca86f-6cb6-47bb-9233-73763ee4982a_66cdca38-8cb5-4be6-8ea2-c7f8695726ee_7cd9c91e-4f41-4605-a01c-60d43072320e/sections> |
| Mohammed Alshahrani | <https://gdt.gradepro.org/forms/#who-coi-preview/p_wzk505_40dca86f-6cb6-47bb-9233-73763ee4982a_a44eec89-c959-4ce1-a6be-2055e9d3072d_47e533ec-0db6-4195-aa0f-ce4583edfd11/sections> |
| Thamer Alshammary | <https://gdt.gradepro.org/forms/#who-coi-preview/p_wzk505_40dca86f-6cb6-47bb-9233-73763ee4982a_5cf07279-d127-43d1-a33b-64f6be1913a9_096a7f3e-a5ac-45bb-a1ca-eab31813b265/sections> |
| Abdulaziz Alsharydah | <https://gdt.gradepro.org/forms/#who-coi-preview/p_wzk505_40dca86f-6cb6-47bb-9233-73763ee4982a_4dea45cf-0ccb-47df-8c89-7239af23f64d_d214ecc1-2d62-494f-a228-dd00b954f42c/sections> |
| Norah Alsubaie | <https://gdt.gradepro.org/forms/#who-coi-preview/p_wzk505_40dca86f-6cb6-47bb-9233-73763ee4982a_2acdf406-3649-4543-aa42-e3fc2a8292dc_ea792c42-666e-40cf-8321-783caeae2b1a/sections> |
| Marwa Amer | <https://gdt.gradepro.org/forms/#who-coi-preview/p_wzk505_40dca86f-6cb6-47bb-9233-73763ee4982a_8fea6bee-d4c2-4ed4-865b-95be35021593_5b2dd861-cc60-4df4-aad0-249efb77e97c/sections> |
| Yaseen Arabi | <https://gdt.gradepro.org/forms/#who-coi-preview/p_wzk505_40dca86f-6cb6-47bb-9233-73763ee4982a_6a7456c1-bf5f-4594-9e59-3998aba98273_1ba9d166-3caa-4b65-8456-b954afcf43ca/sections> |
| Samaher Hashim | <https://gdt.gradepro.org/forms/#who-coi-preview/p_wzk505_40dca86f-6cb6-47bb-9233-73763ee4982a_ee75e123-c4a6-4b2f-881c-791845519e63_61cf95fd-1ad4-4bef-a3ad-df6e77c8f5b8/sections> |
| Nadia Ismail | <https://gdt.gradepro.org/forms/#who-coi-preview/p_wzk505_40dca86f-6cb6-47bb-9233-73763ee4982a_93ec9432-9258-4dd8-88bf-bf920ed566bf_46899f52-fc84-4f4e-a7c6-f912aa312a20/sections> |
| Hassan Mashbari | <https://gdt.gradepro.org/forms/#who-coi-preview/p_wzk505_40dca86f-6cb6-47bb-9233-73763ee4982a_4d472406-e1cb-46f4-be8e-f6c8873e5087_dc8e1181-b34f-421a-98aa-fb490217743e/sections> |
| Thamer Nouh | <https://gdt.gradepro.org/forms/#who-coi-preview/p_wzk505_40dca86f-6cb6-47bb-9233-73763ee4982a_e6b6a081-8bd5-407c-a1e0-7c3a48cf289c_6a9980dc-5f8a-4362-8470-d9db91ffde19/sections> |
| Alqahtani Saad | <https://gdt.gradepro.org/forms/#who-coi-preview/p_wzk505_40dca86f-6cb6-47bb-9233-73763ee4982a_028f4938-d15d-4694-8844-54571c741fd1_714d7a07-cbc8-49b2-be76-75dc23cf8a0b/sections> |
| Wail Tashkandi | <https://gdt.gradepro.org/forms/#who-coi-preview/p_wzk505_40dca86f-6cb6-47bb-9233-73763ee4982a_0add1bc7-7dda-4a78-9aca-651349f1dec3_248c049f-f3af-4e9a-bf12-ab1af9351007/sections> |
| Haifa algethamy | <https://gdt.gradepro.org/forms/#who-coi-preview/p_wzk505_40dca86f-6cb6-47bb-9233-73763ee4982a_16d2c875-4b5b-4945-8ec6-43e81d75e973_fee4c389-7eea-455e-ac5f-6909914ae2fa/sections> |
| Hassan alshaqaq | <https://gdt.gradepro.org/forms/#who-coi-preview/p_wzk505_40dca86f-6cb6-47bb-9233-73763ee4982a_eef6e93a-22a6-4bd7-a26e-a347b16c85f1_5100e2cd-d7ce-40af-be53-036368a51f57/sections> |
| Alyaa elhazmi | <https://gdt.gradepro.org/forms/#who-coi-preview/p_wzk505_40dca86f-6cb6-47bb-9233-73763ee4982a_c276ec17-4453-4370-8d6b-b52d40562b49_3de29dcd-c243-4c6c-ba9e-d485b74e0b77/sections> |
| Khalid maghrabi | <https://gdt.gradepro.org/forms/#who-coi-preview/p_wzk505_40dca86f-6cb6-47bb-9233-73763ee4982a_6e030270-7268-4bdc-88bc-465b9011b7fa_37038b6d-3c81-41fd-9c37-95f185e18e50/sections> |
| ahmed nahhas | <https://gdt.gradepro.org/forms/#who-coi-preview/p_wzk505_40dca86f-6cb6-47bb-9233-73763ee4982a_dbd9df0c-b154-44c2-9b49-ba851c39eaac_5b56a642-024c-4e0f-9b9c-dac5ce857e2d/sections> |
| Esraa taweel | <https://gdt.gradepro.org/forms/#who-coi-preview/p_wzk505_40dca86f-6cb6-47bb-9233-73763ee4982a_63d11c5f-75c2-4a91-b845-296ff00bf147_b1bc3291-7607-48ab-88b0-64cc6e923f59/sections> |
| waleed.al-hazzani | <https://gdt.gradepro.org/forms/#who-coi-preview/p_wzk505_40dca86f-6cb6-47bb-9233-73763ee4982a_1ce16e0f-3080-4bac-95f3-1331cb1b047d_4ce236b2-63dd-477e-961e-1a4a6fe05197/sections> |

**References:**

1. Traversy G, Barnieh L, Akl EA, et al. Managing conflicts of interest in the development of health guidelines. CMAJ 2021;193:E49-54.
2. Schünemann H, Al-Ansary LA, Forland F, et al. Guidelines International Network: Principles for disclosure of interests and management of conflicts in guidelines. Ann Int Med 2015; 163:548-553.
3. Alhazzani W, Lewis K, Jaeschke R, Rochwerg B, Moller MH, Evans L, et al. Conflicts of interest disclosure forms and management in critical care clinical practice guidelines. Intensive Care Med. 2018;44:1691–8.
4. Agoritsas T, Neumann I, Mendoza C, Guyatt GH. Guideline conflict of interest management and methodology heavily impacts on the strength of recommendations: comparison between two iterations of the American College of Chest Physicians Antithrombotic Guidelines. J Clin Epidemiol 2017;81:141-143.
5. Schünemann H, Osborne M, Moss J, et al. An official American Society policy statement: Managing Conflict of Interest in professional societies. Am J Respir Crit Care Med 2009;180:564-580.
